# FRA1 (*FOSL1*) suppresses neoplastic transformation and modulates radiation responses via transcriptional control of mitogenic and stress-responsive networks

**DOI:** 10.3389/fcell.2025.1659986

**Published:** 2025-09-22

**Authors:** Wuroud Al-Khayyat, Taylor Laframboise, Jessica Dougherty, Marc S. Mendonca, Douglas R. Boreham, T. C. Tai, Christopher Thome, Sujeenthar Tharmalingam

**Affiliations:** ^1^ Biomolecular Sciences Program, School of Natural Sciences, Laurentian University, Sudbury, ON, Canada; ^2^ Medical Sciences Division, NOSM University, Sudbury, ON, Canada; ^3^ Radiation and Cancer Biology Laboratories, Department of Radiation Oncology, and Department of Medical & Molecular Genetics, Indiana University School of Medicine, Indianapolis, IN, United States; ^4^ Health Sciences North Research Institute, Sudbury, ON, Canada

**Keywords:** FOSL1, FRA1, AP-1 transcription factor, neoplastic transformation, radiation-induced carcinogenesis, CRISPR, gene expression, CGL1 hybrid cell line

## Abstract

**Purpose:**

FOS-like antigen 1 (FRA1), encoded by *FOSL1*, is an inducible subunit of the AP-1 transcription factor complex and regulates gene expression in response to proliferative and environmental cues. Although FRA1 has been linked to cancer progression, its role in early transformation and radiation responses remains unclear.

**Methods:**

CRISPR-engineered human CGL1 cells—a hybrid of HeLa and normal fibroblasts—were used to evaluate the impact of FRA1 overexpression and knockout on neoplastic transformation. Transformation frequency, clonogenic survival, DNA damage recognition and repair, and cell cycle distribution were assessed following irradiation. Transcriptomic profiling was performed under baseline and serum-stimulated conditions.

**Results:**

FRA1 loss markedly increased both spontaneous and radiation-induced transformation frequency, while overexpression suppressed transformation under both conditions. FRA1-deficient cells were sensitized to radiation-induced cell killing, despite intact DNA damage recognition and repair. In contrast, FRA1 overexpression promoted G2/M accumulation post-irradiation, suggesting enhanced checkpoint activation. Transcriptomic profiling revealed that FRA1 remodels AP-1 complex composition and functions as a transcriptional repressor of mitogen- and stress-responsive genes. FRA1-mediated repression was observed across gene networks involved in extracellular matrix remodeling, hypoxia signaling, inflammation, and proliferation, under both baseline and serum-stimulated conditions.

**Conclusion:**

These findings establish FRA1 as a key modulator of neoplastic transformation and radiation response, acting primarily through transcriptional repression of pro-tumorigenic signaling pathways.

## 1 Introduction

The Activator Protein-1 (AP-1) transcription factor complex regulates gene expression in response to mitogenic and environmental stimuli, influencing cell proliferation, differentiation, and stress responses (Wu et al., 2021; Song et al., 2023; Li et al., 2024). FRA1 (FOS-like antigen 1), encoded by *FOSL1*, is a member of the FOS family that dimerizes with JUN proteins to form functional AP-1 complexes (He et al., 2022; Casalino et al., 2023; He et al., 2024). Unlike cFOS, which is transiently expressed, FRA1 is stabilized under persistent MAPK/ERK signaling and exhibits context-dependent roles in cancer (Yue and Lopez, 2020; He et al., 2022). While FRA1 overexpression is linked to invasion, metastasis, and therapy resistance in late-stage malignancies (Casalino et al., 2022; Zeng et al., 2022; de Visser and Joyce, 2023), emerging evidence suggests that FRA1 may also serve tumor-suppressive functions in early oncogenesis by modulating AP-1 activity and transcriptional homeostasis under baseline and mitogenic stimulation (He et al., 2022; He et al., 2023; Ahn et al., 2024).

Neoplastic transformation is the process by which non-malignant or pre-malignant cells acquire the capacity for uncontrolled growth and tumor formation (Yin et al., 2021; Zeng et al., 2022; Gimla and Herman-Antosiewicz, 2024), typically driven by cumulative genetic and epigenetic alterations that disrupt pathways governing proliferation, survival, DNA repair, and apoptosis (Liu et al., 2014; Lu et al., 2020; Damiescu et al., 2024; Zhang S. et al., 2024). Ionizing radiation (IR) plays a dual role in this context: it is both a mainstay of cancer therapy and a known mutagen (Gilbert, 2009; Ali et al., 2020; Li et al., 2021; Mzizi et al., 2024). IR induces DNA double-strand breaks (DSBs), oxidative stress, and chromosomal instability, all of which promote oncogenic transformation when improperly repaired (Cannan and Pederson, 2016; Ali et al., 2020; Helm and Rudel, 2020; Li et al., 2020; Mzizi et al., 2024). This underscores the need to better understand how cells respond to radiation-induced damage during early oncogenic progression.

Several transcription factors, including the AP-1 complex, mediate cellular responses to radiation (Lee et al., 1995; Hellweg et al., 2016; Song et al., 2023; Xue et al., 2025). AP-1 subunits are rapidly induced following radiation and regulate genes involved in DNA damage response, cell cycle control, and apoptosis (Lee et al., 1995; Christmann and Kaina, 2013; Xue et al., 2025). Elevated cFOS and cJUN levels are associated with increased neoplastic transformation frequency in irradiated breast epithelial cells and the developing rat cerebellum (Deng et al., 2012; Cheng et al., 2013; Aravindan et al., 2016; Tyagi et al., 2017). While cFOS and cJUN are well characterized in radiation-induced transformation, the specific role of FRA1 remains less defined. Given its unique regulation and functional versatility, FRA1 may serve a distinct role in modulating transcriptional responses to radiation and shaping early oncogenic outcomes.

Although FRA1 has been widely studied in advanced cancers for its role in metastasis and therapy resistance (Zeng et al., 2022; Casalino et al., 2023; Liu et al., 2025; Zhao et al., 2025), its function during early transformation is poorly understood. FRA1 activity through the AP-1 complex is highly context-dependent (Liu et al., 2025; Zhao et al., 2025), and its influence on radiation-induced transformation has not been systematically investigated. Most mechanistic studies have focused on cFOS and cJUN, leaving a critical gap in understanding how FRA1 modulates early oncogenic stress responses. Key questions remain regarding which downstream pathways are regulated by FRA1, how it influences AP-1 complex composition, and whether its expression alters the balance between regulated growth and malignant progression. Addressing these gaps is essential to understanding FRA1’s role in early tumorigenesis and may identify new strategies to either harness its tumor-suppressive functions or counteract its pro-oncogenic activity.

The overarching objective of this study was to clarify the functional role of FRA1 in neoplastic transformation and radiation response. To investigate this, we utilized the CGL1 human hybrid cell line—a pre-malignant model derived from fusion of HeLa cervical carcinoma cells and normal fibroblasts—which is widely used to study radiation-induced transformation and early neoplastic events (Lewis et al., 2001; Mendonca et al., 2005; Pirkkanen et al., 2017; Pirkkanen et al., 2019; Peterson et al., 2022). In prior studies, we identified that *FOSL1* was frequently deleted or epigenetically silenced in neoplastically transformed CGL1 clones following irradiation, and that reintroducing *FRA1* suppressed tumor formation *in vivo* (Pirkkanen et al., 2023). We also demonstrated that FRA1 overexpression led to widespread transcriptional changes, reduced cell adhesion, and altered cell cycle progression—supporting a regulatory role for FRA1 in phenotype and stress adaptation (Pirkkanen et al., 2019; Al-Khayyat et al., 2023; Pirkkanen et al., 2023). Together, these findings implicate FRA1 as a potentially critical determinant of early oncogenic behavior. Importantly, prior studies have established FRA1 as a tumor suppressor in cervical cancer, where loss or mutation of *FOSL1* is associated with increased tumor progression (Soto et al., 2000; Prusty and Das, 2005; Xiao et al., 2015; Jiang et al., 2020; Zhang et al., 2020; Jiang et al., 2021). Given that the CGL1 hybrid cell line is derived in part from HeLa cervical carcinoma cells, this model provides a relevant system to investigate FRA1’s tumor-suppressive functions in the context of cervical cancer biology.

Based on this rationale, we hypothesized that FRA1 functions as a tumor suppressor in the CGL1 system by regulating AP-1 complex activity and maintaining transcriptional control over key gene networks involved in homeostasis, proliferation, and stress adaptation. Specifically, we proposed that FRA1 overexpression would suppress, and FRA1 knockout would enhance, neoplastic transformation under both basal conditions and following ionizing radiation exposure. To test this, we established stable FRA1-overexpressing and FRA1 knockout CGL1 cell lines and assessed transformation frequency with and without radiation. We also evaluated how FRA1 modulates radiation sensitivity and DNA damage responses, including clonogenic survival, cell cycle dynamics, and γH2AX accumulation. Finally, we characterized AP-1 subunit expression and FRA1-regulated gene expression programs using RT-qPCR, Western blotting, and RNA sequencing. Through this multifaceted approach, our goal was to define the mechanistic role of FRA1 in early tumorigenesis and stress-responsive transcription.

## 2 Materials and methods

### 2.1 Cell culture

The CGL1 wild-type and CGL1 CRISPR genome edited variants including CGL1^dCas9^, CGL1^FRA1Act^, CGL1^Cas9^, and CGL1^FRA1KO^, were cultured in Minimum Essential Medium (Corning, 10-010CV; Corning, New York, NY, United States) supplemented with 5% calf serum (Sigma-Aldrich, C8056; St. Louis, MO, United States) and 100 U/mL penicillin-streptomycin (Corning, 30001CI). The generation and characterization of the CGL1^dCas9^ and CGL1^FRA1Act^ lines were previously described (Al-Khayyat et al., 2023). The human embryonic kidney 293-T (HEK293T) cells were purchased from ATCC (CRL-3216) and cultured in Dulbecco’s Modified Eagle Medium (HyClone, SH3028501; Marlborough, MD, United States), supplemented with 10% fetal bovine serum (HyClone, SH3039603HI) and 100 U/mL penicillin-streptomycin. All cell lines were incubated in a humidified atmosphere at 37 °C with 5% CO_2_.

### 2.2 Design and cloning of CRISPR knockout gRNA sequences into a lentiviral transfer plasmid

Target-specific guide RNA (gRNA) sequences for the *FOSL1* gene were designed using the Sigma-Aldrich Advanced Genomics bioinformatics platform. gRNA selection was restricted to 20-nucleotide guide sequences immediately upstream of a 5′-NGG protospacer adjacent motif (PAM), required for *Streptococcus pyogenes* Cas9 activity, to ensure high on-target efficiency while minimizing off-target effects. The top three gRNA sequences [#1: 5′-TTCGACGTACCCCTGGAGG-3′ (exon #2; NC_000011.10: 65896823–65896841); #2: 5′-TGGTACAGCCTCATTTCCTG-3′ (exon #2; NC_000011.10: 65896929–65896948); and #3: 5′-TCCGCTCGCGCCTTACTCGG-3′ (exon #3; NC_000011.10: 65894076–65894095)] were synthesized as complementary oligonucleotides and cloned into a third generation lentiviral gRNA vector (Addgene, 52963; Watertown, MD, United States). This plasmid contains BsmBI sites for scarless insertion of the gRNA target sequences. Therefore, the BsmBI overhang sequences were appended to the gRNA target sequences and ordered as single-stranded oligonucleotides from IDT. The forward and reverse oligonucleotides were phosphorylated and annealed in-house at a 10 μL reaction volume. This reaction mixture contained 10 μM of each oligonucleotide, 1 mM ATP (NEB), 1X Kinase Reaction Buffer A (NEB), and 5 units of T4 polynucleotide kinase (NEB). The phosphorylation and annealing process was carried out in a thermocycler with the following parameters: an initial incubation at 37 °C for 30 min to allow for phosphorylation, followed by a denaturation step at 95 °C for 5 min. Subsequently, the temperature was gradually decreased to 25 °C at a rate of 0.1 °C per second to enable annealing of the oligonucleotides. After annealing, the reaction mixture was diluted 250-fold by adding 2 μL of the annealed oligonucleotides to 498 μL of molecular grade water (HyClone, SH30538.LS). This dilution step is crucial to achieve the appropriate concentration for subsequent cloning steps. Single-step digestion and ligation of the vector and annealed oligonucleotides was completed in a 20 μL reaction volume consisting of 100 ng lentiviral gRNA transfer plasmid, 2 μL of diluted and annealed oligonucleotides, 1X Tango Buffer (ThermoScientific), 1 mM DTT (NEB), 1 mM ATP (NEB), 10 units BsmBI (ThermoScientific), and 1 μL T4 Quick Ligase (NEB). The reaction was completed in a thermocycler using the following parameters: (1) 37 °C for 5 min, (2) 23 °C for 5 min, and (3) steps 1 and 2 repeated for 6 cycles. 2 μL of the final ligation product was transformed into NEB Stable Competent *Escherichia coli* cells according to the manufacturer’s instructions and selected for ampicillin resistance (100 μg/mL). Select clones were amplified and subjected to plasmid purification using the GeneJet Plasmid Miniprep Kit (ThermoFisher, K0502), according to the manufacturer’s instructions. Successful insertion of the gRNA target sequences into the lentiviral backbone vector was verified by Sanger sequencing performed by The Centre for Applied Genomics (TCAG, SickKids, Toronto, ON, Canada).

### 2.3 Preparation of CRISPR lentiviral particles via calcium phosphate transfection

HEK293T cells were plated into three 10 cm dishes at 1.0 × 10^6^ cells per dish 48 h before transfection. Cells were ∼50–60% confluent on the day of transfection. Two hours prior to the transfection, the media was replaced with 9.0 mL/dish of prewarmed complete media without antibiotics. In a 15 mL tube, the following amounts of third generation lentiviral plasmids were mixed to a final volume of 1.35 mL with molecular grade water: 10 μg pMD2.G (Addgene, 12259), 30 μg psPAX2 (Addgene, 12260), and 40 μg transfer plasmid (Addgene, 52962-Cas9 or cloned CRISPR gRNA plasmids described above); 150 μL of 2.5 M CaCl_2_ were added to the plasmid mixture and mixed by vortex. Next, 1.5 mL of 2× HEPES-buffered saline (0.05 M HEPES, 0.28 M NaCl, 1.5 mM Na_2_HPO_4_, pH 7.0) were added dropwise while the solution was continuously vortexed. After incubating the transfection solution for 15 min at room temperature, 1 mL of solution was added dropwise into each 10 cm plate. After 20 h post-transfection, the media was replaced with 7 mL of complete media per 10 cm dish. The supernatant containing the lentiviral particles was collected 8 h after the media change. The supernatant was centrifuged at 500 × g for 5 min at 4 °C to remove cellular debris and cleared using a 0.45 μm syringe filter. The viral solution was aliquoted into 1 mL fractions and stored at −80 °C.

### 2.4 CGL1 lentiviral infection for producing CRISPR/Cas9 mediated FRA1 knockout

CGL1 cells were seeded into a six-well plate in complete media at 100,000 cells per well 24 h before the lentiviral infection. Cells were ∼40–50% confluent on the day of the infections. Two hours prior to the infection, the media was replaced with 0.9 mL/well of complete media. The infection was initiated via the addition of 1 mL of lentiviral solution and 100 μL of 20X polybrene solution (8 μg/mL final concentration). After 16 h, the virus solution was removed, and each well was incubated with 2 mL of complete media. After 48 h, the cells were passaged and transferred to T25 flasks under antibiotic selection (0.8 μg/mL blasticidin for Cas9 selection; 8 μg/mL puromycin for gRNA selection). The antibiotic selection was maintained for four rounds of passage, at which point the cells were maintained without the selection antibiotic. FRA1-knockout CGL1 cells were generated via sequential viral infections. First, Cas9-expressing CGL1 cells were generated (CGL1^Cas9^) and verified via immunohistochemical analysis using a Cas9 antibody (ThermoScientific, 10C11-A12). Next, the CGL1^Cas9^ cells were subsequently infected with three unique lentiviral preparations containing varying gRNA sequences designed for FRA1 knockout (CGL1^FRA1KO−#1/#2/#3^). All assays were performed within 4–7 passages from the initial CRISPR lentiviral infections.

### 2.5 RNA extraction

Total RNA was extracted from cells using the TRIzol reagent (Invitrogen, Waltham, MA, United States), as described previously (Liu et al., 2015; Liu et al., 2014). Briefly, cells were seeded in six-well plates with complete media and collected at ∼70–80% confluency. Cells were washed with phosphate-buffered saline (PBS) and harvested using 500 μL of TRIzol per well. The samples were transferred into a 1.5 mL tube and mixed with 100 μL of chloroform. The mixture was incubated at room temperature for 15 min and centrifuged at 12,000 × g for 20 min at 4 °C. After centrifugation, the top aqueous layer containing the total RNA fraction was transferred to a new 1.5 mL tube. The RNA was precipitated with 125 μL of isopropanol. The mixture was vortexed for 15 s, incubated for 10 min at room temperature, and centrifuged at 12,000 × g for 8 min at 4 °C. The supernatant was discarded, and the RNA pellet was washed with 1 mL of 70% ethanol. The solution was centrifuged at 7,500 × g for 5 min. The supernatant was removed, and the RNA pellet was air-dried for 10 min. The RNA pellet was dissolved in 20 μL of molecular grade water in a thermomixer for 10 min at 37 °C at 1,000 rpm. The RNA samples were incubated on ice for 20 min, followed by RNA analysis on the NanoDrop spectrophotometer (ThermoScientific, ND-1000). Absorbance ratios of 260/280 nm > 1.8 were considered suitable for downstream applications.

### 2.6 cDNA synthesis

Complementary DNA (cDNA) synthesis was performed using the SuperScript IV First-Strand Synthesis (ThermoScientific, LS18090050) kit according to the manufacturer’s instructions, with minor modifications. Briefly, 1 μg of RNA was subjected to DNase treatment and converted to cDNA using random hexamers and the reverse transcriptase enzyme. The reaction volume was adjusted to 25 μL to obtain a final cDNA concentration of 40 ng/μL and stored at −80 °C.

### 2.7 RT-qPCR

Forward and reverse primer pairs, for SYBR-Green-based RT-qPCR analysis, were designed in-house and validated under stringent conditions (efficiency between 90% and 110% and R^2^ > 0.99), as described previously (Hunt et al., 2023; Liu et al., 2023; Liu et al., 2025; Livak and Schmittgen, 2001). Table 1 lists the validated RT-qPCR primer sequences utilized in this study. RT-qPCR reactions were performed using the QuantStudio 5 Real-Time PCR instrument (ThermoScientific) in 96-well plates. The RT-qPCR reaction mix was completed in 15 μL reaction volume consisting of 5 ng cDNA, 600 nm forward and reverse primers, and 7.5 μL 2× Luna Universal qPCR Master Mix (NEB, M3003). The RT-qPCR cycling parameters consisted of 95 °C for 1 min followed by a two-step denaturation and extension cycle of 95 °C for 15 s and 60 °C for 30 s for a total of 40 cycles. Plate reading was performed at the end of the extension phase. A DNA melt curve was performed at the completion of each RT-qPCR experiment to assess the amplification specificity. The RT-qPCR data was analyzed using the 2^−ΔΔCT^ analysis method (Livak and Schmittgen, 2001). All samples were normalized to two independent housekeeping genes (*RSP18* and *RPL4*). The relative mRNA expression of each gene was reported as the mRNA fold change ±standard error of means (SEM) relative to the control cells (CGL1^dCas9^ or CGL1^Cas9^).

**TABLE 1:** RT-qPCR primer sequences. Forward and reverse primer pairs for SYBR-Green based RT-qPCR analysis designed using Primer-BLAST and validated under stringent conditions (efficiency between 90% and 110% and R^2^ > 0.99). The optimal annealing temperature for all primer sets was 60 °C.

Gene name	Gene ID	Primer seqeunce (5′→ 3′)
*53BP1*	NM_005657	Forward: AGCAGAACAGTCCAGCAAGG
		Reverse: ACCTTGCAGGTGGTGGATTT
*BCL2*	NM_000633.3	Forward: GATAACGGAGGCTGGGATGC
		Reverse: TCACTTGTGGCCCAGATAGG
*CCNB1*	NM_031966.4	Forward: ACCTGTGTCAGGCTTTCTCTG
		Reverse: CTGACTGCTTGCTCTTCCTCA
*CCND1*	NM_053056.3	Forward: ATCAAGTGTGACCCGGACTG
		Reverse: CTTGGGGTCCATGTTCTGCT
*CDK1*	NM_001786.5	Forward: CTTGGCTTCAAAGCTGGCTC
		Reverse: GGGTATGGTAGATCCCGGCT
*CDKN1A*	NM_001291549.3	Forward: TGTTTTCAGGTGAGGAAGGGG
		Reverse: TGCTTGTCATCCTTTATTTCTGG
*CHK1*	NM_001114121.2	Forward: TCTGCTCCTCTAGCTCTGCT
		Reverse: GCCCCTTTCTTGAGGGGTTT
*FOS*	NM_005252.4	Forward: GGGGCAAGGTGGAACAGTTA
		Reverse: AGTTGGTCTGTCTCCGCTTG
*FOSL1*	NM_005438.5	Forward: GCCTTGTGAACAGATCAGCC
		Reverse: AGTTTGTCAGTCTCCGCCTG
*JUN*	NM_002228.4	Forward: CTTTTCAAAGCCGGGTAGCG
		Reverse: TTTCTCTAAGAGCGCACGCA
*PCNA*	NM_002592.2	Forward: GCTCTTCCCTTACGCAAGTCT
		Reverse: AGTCTAGCTGGTTTCGGCTT
*RAD51*	NM_133487.4	Forward: GAAGTGGAGCGTAAGCCAGG
		Reverse: GCATTGCCATTACTCGGTCC
*RPL4*	NM_000968.4	Forward: CACGCAAGAAGATCCATCGC
		Reverse: CCGGAGCTTGTGATTCCTGG
*RPS18*	NM_022551.2	Forward: ATTAAGGGTGTGGGCCGAAG
		Reverse: GGTGATCACACGTTCCACCT
*TNFRSF1A*	NM_001065.4	Forward: CTGCTGCCACTGGTGCTC
		Reverse: AGGTGAGGGACCAGTCCAAT

### 2.8 Protein extraction and Western blot analysis

Cells were harvested in 10 cm culture dishes at ∼70–80% confluency for protein extraction. Briefly, cells were washed with PBS, trypsinized, neutralized with media, and centrifuged. The cell pellet was washed with ice-cold PBS, followed by resuspension of the pellet with 100 μL of fresh RIPA lysis buffer (150 mM NaCl, 5 mM EDTA, 50 mM Tris, 1% NP-40, 0.5% sodium deoxycholate, and 0.1% SDS; pH 7.5) supplemented with a protease inhibitor mix (ThermoScientific, A32955). The lysis mixture was agitated for 30 min at 4 °C with intermittent vortex, followed by centrifugation at 20,000 × g for 20 min at 4 °C. The soluble protein fraction was transferred to a 1.5 mL tube and stored at −80 °C. Protein concentration was determined using the Pierce BCA Protein Assay Kit (ThermoScientific, PI23225) according to the manufacturer’s instructions. The protein samples were analyzed via gel electrophoresis using the BOLT Bis-Tris Plus gel system (ThermoScientific). Briefly, samples were prepared for gel electrophoresis in 50 μL of reaction volumes consisting of 25 μg of RIPA solubilized protein sample, 1X Bolt LDS Sample Buffer (LSB0007), and 1X Bolt Reducing Agent (LSB0004). The samples were sonicated at 10 Hz for 20 s, followed by 70 °C for 10 min. The protein ladder (ThermoScientific, 26619) and samples were loaded onto a Blot Bis-Tris Plus Mini Gel (ThermoScientific, NW04120BOX) and electrophoresed for 22 min at a constant 200 V. The contents of the gel were transferred to a nitrocellulose membrane (PALL, 66593) using the Mini Bolt Module (B1000) according to the manufacturer’s instructions. The blots were blocked with 5% bovine serum albumin (BSA) in TBS-T buffer (Tris-buffered saline with 0.1% Tween-20) for 60 min at room temperature. The blots were washed three times with TBS-T for 5 min each at room temperature, followed by incubation with the following primary rabbit monoclonal antibodies overnight at 4 °C: FRA1 (1:1,000; Cell Signaling #5281T) and cFOS (1:500; Cell Signaling #2250S). The blots were washed four times with TBS-T and incubated with HRP-conjugated goat antirabbit secondary antibody (ThermoScientific, G21234) at 1:10,000 dilution for 1 h at room temperature. After four washes with TBS-T, the blots were incubated with 2 mL of ECL mixture (Medi-Res Corp. #Bi2M-CK-ECL) for 5 min followed by signal imaging using the ChemiDoc Imaging System (Bio-Rad, Hercules, CA, United States). The antibodies were stripped from the blots using a mild stripping buffer (0.2 M glycine, 0.5% SDS, and 1% Tween-20; pH 2.2) for 10 min at room temperature. The blocking, washing, and antibody incubation procedure described above were repeated for the mouse monoclonal α-Tubulin housekeeping antibody (1:2,000; Medi-Res Corp. #Bi2M-GB15200) or mouse monoclonal Gapdh (1:20,000; ThermoScientific, MA515738), followed by the HRP-conjugated goat anti-mouse secondary antibody (1:10,000; ThermoScientific, LSG21234). The band intensities were quantified using the ImageJ analysis software.

### 2.9 Growth curve

In T25 flasks, 100,000 cells were seeded in complete media and incubated in a 37 °C humified incubator at 5% CO_2_. Cell counts were performed every 24 h over a period of 7 days, with duplicates for each time point. Media changes were performed on day 3 and day 5. For cell collection, the cells were first washed with PBS, followed by the addition of 0.05% trypsin. After trypsinization, cells were neutralized using complete media. The total cell count per T25 flask was determined using a hemocytometer. Statistical analysis was performed using GraphPad Prism. The mean cell count ±SEM for three independent experiments is provided for each cell line.

### 2.10 Radiation exposure

Irradiations were performed using an X-Rad320 irradiation cabinet (Precision X-Ray). Cells were exposed to an x-ray dose of 0.5–7 Gy, depending on the endpoint (described below). The irradiator was operated at 320 kV and 12.5 mA with a 2 mm Al filter, resulting in a dose rate of approximately 3.5 Gy/min. Sham irradiated (0 Gy) cells were handled in parallel and were placed in the X-Rad320 unit without turning the beam on. Dose rates were verified using thermoluminescent dosimeters.

### 2.11 Neoplastic transformation assay

To evaluate the frequency of neoplastic transformation, CGL1 cells with FRA1 overexpression (CGL1^FRA1Act^), FRA1 knockout (CGL1^FRA1KO^), and their respective control cell lines (CGL1^dCas9^ and CGL1^Cas9^) were subjected to X-radiation (0 Gy or 7 Gy) and seeded for long-term colony outgrowth. Cells were seeded in T75 flasks 6 h post irradiation. Transformation assays were performed across 5–7 independent experiments, with each condition comprising approximately 50–70 replicate T75 flasks in total. For each condition, the number of cells plated per flask was calculated based on prior plating efficiency and radiation survival measurements to ensure a viable surviving density of 50 cells/cm^2^. Accordingly, CGL1^Cas9^ cells were seeded at 5,100 cells per flask (0Gy) and 87,500 cells per flask (7 Gy). CGL1^dCas9^ cells were seeded at 4,800 cells per flask (0 Gy) and 88,900 cells per flask (7 Gy). CGL1^FRA1Act^ cells were seeded at 4,400 cells per flask (0 Gy) and 75,800 cells per flask (7 Gy). CGL1^FRA1KO^ cells were seeded at 6,300 cells per flask (0 Gy) and 223,900 cells per flask (7 Gy).

Following irradiation and seeding, T75 flasks were incubated at 37 °C in a humidified incubator with 5% CO_2_ for 21 days. To maintain optimal growth conditions, warm fresh media was added on days 7, 11, 14, and 18 of the incubation period. On day 21, cultures were gently washed three times with 5 mL of PBS, fixed with 10 mL of 2% paraformaldehyde for 20 min at room temperature, and then rinsed twice with PBS. Neoplastically transformed colonies were visualized by staining with 2 mL of Western Blue alkaline phosphatase substrate (Promega #S3841) for 20 min. After staining, flasks were washed three times with PBS, and blue-stained intestinal alkaline (IAP)-positive foci were manually counted under a stereomicroscope.

A second set of flasks was seeded to calculate the number of cells at risk of transformation (i.e., the number of cells that survived plating and the radiation exposure) based on clonogenic survival. Cells were seeded into T25 flasks at a density of 200 cells per flask for the sham (0 Gy) condition and 2,000 cells per flask for the 7 Gy condition. Following seeding, cells were incubated for 8 h, exposed to x-rays, and incubated at 37 °C in a humidified atmosphere with 5% CO_2_ for 7 days. On day 7, flasks were stained with 1 mL of 0.3% crystal violet for 20 min to visualize colony formation. Colonies containing >30 and <200 cells were counted manually.

Transformation frequency was determined by calculating the number of neoplastically transformed foci per surviving cell for each condition. To account for variability in foci distribution and potential satellite colony formation, the average number of transformed foci per flask was estimated using the method described by Elkind and Han (1979), which utilizes the proportion of flasks without visible foci. This statistical approach improves accuracy and minimizes bias in transformation frequency estimates. Average transformation frequency using the following formula, where PE is the plating efficiency.

$$TA=\frac{Total\;\;Foci\;\#}{\#\;cell\;\;seeded\times PE}$$

### 2.12 Clonogenic survival assay

Clonogenic survival was assessed in CGL1 parental and FRA1-modified cell lines following X-radiation. Cells were seeded in triplicate at densities optimized for colony formation at varying radiation doses: 200 cells per flask for 0, 0.5, 1, and 2 Gy; 2,000 cells for 4 Gy; and 3,000 cells for 6 and 7 Gy. After an 8-h attachment period, cells were irradiated and subsequently incubated under standard culture conditions for 9 days. At the end of the incubation period, flasks were gently washed three times with PBS, fixed, and stained with 0.03% crystal violet. Colonies were allowed to dry overnight and counted manually the following day. Only colonies containing at least 50 cells were included in the survival analysis. 0 Gy flasks were used to calculate the plating efficiency (PE). Survival fraction (SF) at each radiation dose was then calculated using the following formula:

$$SF=\frac{\#\;colonies}{\#\;cell\;\;seeded\times PE}$$

### 2.13 Cell cycle and DNA damage flow cytometry analysis

In T25 flasks containing complete media, 200,000 cells were seeded and incubated for 48 h, after which the media was replaced with fresh complete media. The flasks were X-ray irradiated (2 Gy), and samples were collected at various time points, including 0, 2, 4, 8, and 24 h post-irradiation. At each time point, cells were washed with PBS, trypsinized, neutralized, and counted using a hemocytometer. 250,000 cells per flask were transferred to assay tubes for analysis. Cells were fixed overnight at −20 °C with 70% ethanol, then washed with PBS and blocked by adding 1 mL of cold blocking buffer (1% BSA and 0.1% Triton-X 100 in PBS), followed by incubation at room temperature (RT) for 30 min on a rocker. Cells were centrifuged at 300 × g for 5 min, after which the supernatant was decanted, and cells were resuspended by vortexing. Next, 200 μL of γH2AX primary antibody (0.5 μg/mL in blocking buffer, ThermoFisher Scientific, Waltham, MA, United States) was added, and cells were incubated at RT for 120 min on a rocker. Following this the cells were washed with 3 mL of cold blocking buffer, centrifuged, and resuspended in 200 μL of Alexa Fluor 488 secondary antibody (5 μg/mL, Thermo Fisher Scientific, Waltham, MA, United States) for 1 h at RT for 60 min in the dark on a rocker. Finally, cells were stained with propidium iodide (PI) for cell cycle analysis. Cells were washed with 3 mL of cold wash buffer, centrifuged, and resuspended, followed by the addition of 300 μL of PI (25 μg/mL) and incubated on ice for 10 min in the dark. Samples were analyzed using a Sony SA3800 flow cytometer, and the data were processed with Kaluza software. Following initial gating on the cell population, the percentage of cells in each cell cycle stage (G0/G1, S, G2) was determined based on PI fluorescence. The average γH2AX fluorescence was then determined separately on G0/G1 and G2 gated cells. A minimum of 10,000 cells was analyzed for each treatment.

### 2.14 Gene expression analysis following radiation exposure

A total of 100,000 cells were seeded in T25 flasks containing complete media and incubated at 37 °C with 5% CO_2_. After 48 h, cells were exposed to 7 Gy X-radiation (or sham). At 1 h and 8 h post-irradiation, cell pellets were collected and processed for RNA extraction, cDNA synthesis, and RT-qPCR as described previously.

### 2.15 Serum stimulation assay

100,000 cells were seeded in a 10 cm cell culture dish containing complete 1X MEM media and incubated at 37 °C in a 5% CO_2_ incubator for 48 h. Serum stimulation was initiated by replacing the media with fresh complete MEM. Cells were harvested at 0, 2, 4, and 8 h post-stimulation. Total RNA and protein were extracted at each time point for RT-qPCR and Western blot analyses to assess AP-1 family gene and protein expression, as well as for downstream RNA-sequencing analysis.

### 2.16 Whole transcriptome RNA sequencing

Total RNA was extracted using the TRIzol reagent and further purified with the NEB Monarch® RNA Cleanup Kit (T2040L, NEB). High-quality RNA (A260/280 ∼2.0 ± 0.1) was used to generate strand-specific libraries using the NEBNext® Ultra™ II Directional RNA Library Prep Kit (E7765S, NEB), which enriches for poly-A mRNA and incorporates the dUTP method for strand specificity. Libraries were quantified using the NEBNext® Library Quant Kit (E7630S, NEB), pooled at equimolar concentrations, and sequenced on the Illumina NovaSeq X platform at The Centre for Applied Genomics (TCAG), generating approximately 50 million 150 bp paired-end reads per sample. The sequencing data was processed in-house using various bioinformatics toolkits available through the Illumina Sequence Hub (San Diego, CA, USA), powered by DRAGEN Inc. Briefly, *DRAGEN FASTQ* was used to assess sequencing quality and perform read trimming. *DRAGEN RNA* was employed to align reads to the human reference genome (GRCh38.p14; hg38) and quantify transcript abundance. Differential expression analysis was conducted using the *DRAGEN* Differential Expression pipeline, which incorporates the *DESeq2* algorithm. Genes were considered differentially expressed if they exhibited a fold change <−1.5 or >1.5, an FDR-adjusted p-value <0.05, and an average transcript abundance of at least 30 transcripts per million (TPM). Selected targets were validated by RT-qPCR.

### 2.17 Quantification and statistical analysis

All data represent the mean ± standard error of the mean (SEM) from a minimum of three independent experiments. Statistical analyses were performed using GraphPad Prism software. One-way or two-way ANOVA and unpaired t-tests were used, as appropriate, to assess differences between experimental groups. A p-value <0.05 was considered statistically significant.

## 3 Results

### 3.1 Establishing FRA1-knockout and overexpressing CGL1 cells

**FIGURE 1:**
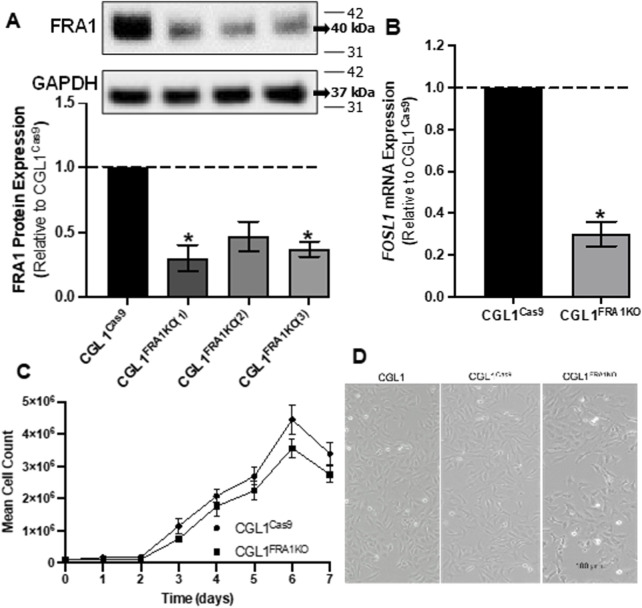
Analysis of FRA1 knockout in CGL1 cells and its effects on cell growth and morphology. **(A)** Western blot analysis of FRA1 protein expression in CGL1^Cas9^ cell line and CGL1^FRA1KO^ clones. FRA1 protein levels were significantly reduced by approximately 70% and 60% (p < 0.05, n = 4) in the knockout clones 1 and 3 respectively compared to controls. Since CGL1^FRA1KO^ clone 1 showed the greatest FRA1 reduction, it was used for subsequent experiments and will be referred to as CGL1^FRA1KO^. Molecular weight markers (kDa) are shown on the right; FRA1 protein was detected at ∼42 kDa and GAPDH at ∼37 kDa. Full blot images, including all three biological replicates used for quantification, are provided in Supplementary Figure S1. **(B)** RT-qPCR analysis of *FOSL1* mRNA expression in CGL1^Cas9^ and CGL1^FRA1KO^ cells, indicating a statically significant decrease by 70% (p < 0.05, n = 3) of *FOSL1* mRNA levels. Data are presented as the mean ± standard error of the mean (SEM) from 3 independent experiments, with error bars indicating variability among replicates. **(C)** Growth curve of CGL1^Cas9^ and CGL1^FRA1KO^ cells measured over 7 days. Cell counts were normalized to the initial seeding density, and no significant differences were observed between groups. Results are shown as the mean ± SEM, with error bars representing variability across 3 independent experiments. **(D)** Representative phase-contrast images of CGL1, CGL1^Cas9^, and CGL1^FRA1KO^ cells during exponential growth. No significant changes in cell morphology were observed among the groups. Scale bar = 100 μm.

To investigate the functional role of FRA1 in neoplastic transformation and mitogenic signaling, we first generated CGL1 cell models with targeted deletion of the *FOSL1* gene, which encodes the FRA1 protein. FRA1 knockout cells were created using the CRISPR/Cas9 gene editing system, which consists of the CRISPR-associated protein 9 (Cas9) endonuclease enzyme and a single guide RNA (gRNA) sequence complementary to *FOSL1*. The CGL1^FRA1KO^ cell line was generated through a two-step lentiviral infection process. First, CGL1 cells were infected with a Cas9-expressing lentivirus, creating CGL1^Cas9^ control cells. Subsequently, CGL1^Cas9^ cells were transduced with a *FOSL1*-targeting gRNA lentivirus, leading to the establishment of the CGL1^FRA1KO^ knockout cell line. To optimize the knockout efficiency, we used three distinct *FOSL1* gRNA sequence variants to generate CGL1^FRA1KO−#1, #2, and #3^ cell lines. These variants allowed us to assess the effectiveness of different gRNA sequences in targeting and deleting *FOSL1* expression. To validate FRA1 protein expression, Western blot analysis was conducted. Among the knockout variants, CGL1^FRA1KO#1^ and CGL1^FRA1KO#3^ demonstrated statistically significant FRA1 protein knockdown in two of the three clones (Figure 1A). FRA1 levels were significantly reduced by approximately 70% in CGL1^FRA1KO#1^ and 60% in CGL1^FRA1KO#3^ compared to control cells (p < 0.05, n = 3), while CGL1^FRA1KO#2^ showed a reduction that did not reach statistical significance (Figure 1A). Based on these results, CGL1^FRA1KO#1^, which exhibited the most robust FRA1 protein reduction, was selected for further experiments and will be referred to as CGL1^FRA1KO^. Next, we examined whether *FOSL1* mRNA levels aligned with FRA1 protein expression. Consistent with protein-level data, RT-qPCR revealed a significant 75% reduction in *FOSL1* mRNA expression in CGL1^FRA1KO^ cells relative to controls (Figure 1B), confirming successful gene disruption at both transcript and protein levels. Although residual *FOSL1* mRNA and FRA1 protein expression were detected, the marked reductions observed at both the transcript and protein levels—together with the robust phenotypic consequences described in later sections—suggest that FRA1 activity is functionally impaired. This residual expression is likely due to in-frame indels or partially functional hypomorphic alleles, which are common outcomes in CRISPR/Cas9-edited cell lines (Hunt et al., 2023).

To evaluate whether FRA1 knockout impacted cell proliferation, a growth-curve analysis was conducted using CGL1^Cas9^ and CGL1^FRA1KO^ cell lines (Figure 1C). Cell growth was measured at 24-h intervals over a period of 7 days. No statically significant differences were observed in the growth patterns among the two cell lines (n = 3), indicating that loss of FRA1 does not affect cell doubling under standard culture conditions. Likewise, phase-contrast imaging of exponentially growing CGL1, CGL1^Cas9^, and CGL1^FRA1KO^ cells revealed no discernible differences in cell morphology among the three lines (Figure 1D). All cell lines retained fibroblast-like characteristics similar to wildtype CGL1 cells. Taken together, these findings suggest that FRA1 knockout does not affect the growth rate or morphological features of CGL1 cells.

The generation and characterization of CGL1 cells stably overexpressing FRA1 (CGL1^FRA1Act^) and their dCas9-expressing control line (CGL1^dCas9^) have been described previously (Al-Khayyat et al., 2023). In brief, cells were engineered using CRISPR activation (CRISPRa) technology via dCas9-VP64 and a *FOSL1*-targeting guide RNA to induce sustained overexpression of endogenous FRA1. The CGL1^FRA1Act^ cell line demonstrated 2–3 fold increase in *FOSL1* mRNA and FRA1 protein expression and were shown to exhibit no baseline growth or morphological differences relative to controls. Together, these validated FRA1-modified CGL1 cell lines provide a robust experimental platform to investigate the functional consequences of FRA1 loss or overexpression in the context of radiation response and mitogen-induced gene regulation.

### 3.2 FRA1 suppresses neoplastic transformation in CGL1 cells

**FIGURE 2:**
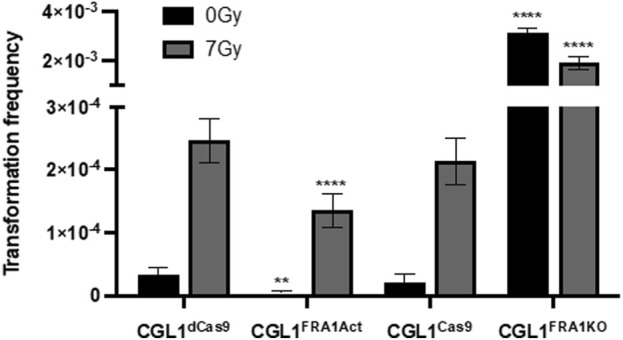
Neoplastic transformation frequency in CGL1 cells with FRA1 overexpression or knockout following sham (0 Gy) or 7 Gy X-radiation. Transformation assays were conducted in CGL1^dCas9^, CGL1^FRA1Act^, CGL1^Cas9^, and CGL1^FRA1KO^ cell lines under 0 Gy (black bars) and 7 Gy (grey bars) conditions. Baseline transformation frequency in both control cell lines (CGL1^dCas9^ and CGL1^Cas9^) ranged between 0.15–0.2 × 10^−5^, and increased to 2.2–2.4 × 10^−4^ following 7 Gy radiation. FRA1 overexpression significantly suppressed baseline transformation by 90% (p < 0.0001, **, n = 70) and radiation-induced transformation by 45% (**p < 0.0001, n = 70; Two-Way ANOVA). Conversely, FRA1 knockout increased baseline transformation by 154-fold (**p < 0.0001, n = 50) and radiation-induced transformation by 8-fold (**p < 0.0001, n = 50; Two-Way ANOVA). Data represent mean ± SEM from 5 (FRA1 overexpression) and 6 (FRA1 knockout) independent experiments, each consisting of 10–2s0 replicate flasks, for a total of 50–70 flasks per condition.

To investigate the impact of FRA1 on early tumorigenic events, we performed neoplastic transformation assays using the CGL1 cell system, a well-established model for studying radiation-induced transformation and oncogenic potential (Lewis et al., 2001; Mendonca et al., 2005; Pirkkanen et al., 2017). This assay quantifies the frequency of neoplastically transformed cells based on their ability to form foci with elevated alkaline phosphatase activity, which serves as a surrogate marker of early tumorigenic transformation *in vitro*. Experiments were conducted in FRA1 knockout (CGL1^FRA1KO^), FRA1 overexpressing (CGL1^FRA1Act^), and control cell lines following sham (0 Gy) or 7 Gy X-irradiation. In control cells (CGL1^Cas9^ and CGL1^dCas9^), baseline transformation frequencies ranged from 0.15 to 0.2 × 10^−5^, and exposure to 7 Gy X-radiation increased transformation rates approximately 100-fold, to 2.2–2.4 × 10^−4^. FRA1 overexpression significantly suppressed transformation frequency under both sham and irradiated conditions. At baseline, CGL1^FRA1Act^ cells exhibited a 90% reduction in transformation relative to CGL1^dCas9^ controls (0.02 × 10^−5^ vs. 0.2 × 10^−5^; p = 0.0019, n = 70). Following 7 Gy irradiation, FRA1 overexpression reduced transformation by approximately 45%, from 2.4 × 10^−4^ to 1.3 × 10^−4^ (p < 0.0001, n = 70; Two-Way ANOVA). Conversely, FRA1 knockout markedly increased neoplastic transformation. Under sham conditions, transformation frequency rose from 0.2 × 10^−5^ in CGL1^Cas9^ cells to 30.8 × 10^−5^ in CGL1^FRA1KO^ cells—representing a 154-fold increase (p < 0.0001, n = 50). Following radiation, transformation increased 8-fold, from 2.2 × 10^−4^ in controls to 17.6 × 10^−4^ in FRA1 knockout cells (p = 0.0018, n = 50; Two-Way ANOVA) (Figure 2). Together, these results demonstrate that FRA1 exerts a potent suppressive effect on both spontaneous and radiation-induced neoplastic transformation in CGL1 cells, supporting a tumor-suppressive role for FRA1 in this cellular context.

### 3.3 Impact of FRA1 on radiation-induced clonogenic survival

**FIGURE 3:**
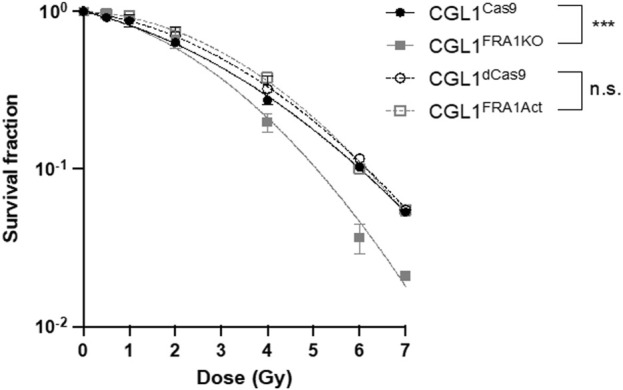
Clonogenic survival of CGL1 cells with FRA1 overexpression or knockout following X-radiation. CGL1^dCas9^, CGL1^FRA1Act^, CGL1^Cas9^, and CGL1^FRA1KO^ cell lines were exposed to increasing doses of X-radiation (0.5, 1, 2, 4, 6, and 7 Gy). Cells were incubated for 9 days post-irradiation and stained with crystal violet to assess colony formation. Clonogenic survival was significantly reduced in FRA1 knockout cells compared to CGL1^Cas9^ controls (p < 0.0001, ***; Two-Way ANOVA), whereas FRA1 overexpression did not significantly affect survival compared to CGL1^dCas9^ controls. Data represent the mean ± SEM from 3 independent experiments. Data were fit with a linear quadratic relationship.

Given the opposing effects of FRA1 overexpression and knockout on neoplastic transformation, we next evaluated whether these phenotypes were associated with altered radiosensitivity using clonogenic survival assays. This assay assesses the ability of single cells to undergo sustained proliferation following ionizing radiation exposure and reflects intrinsic differences in DNA repair capacity, cell cycle regulation, and apoptosis susceptibility (Pirkkanen et al., 2017; Pirkkanen et al., 2019; Peterson et al., 2022). CGL1^FRA1Act^, CGL1^FRA1KO^, and their respective controls (CGL1^dCas9^ and CGL1^Cas9^) were irradiated with 0.5–7 Gy X-rays and incubated for 9 days to allow colony formation (Figure 3). FRA1 knockout significantly impaired clonogenic survival compared to CGL1^Cas9^ controls, with reductions of 64% at 6 Gy and 61% at 7 Gy (p < 0.0001; Two-Way ANOVA, n = 3). In contrast, FRA1 overexpression did not significantly alter survival relative to CGL1^dCas9^ controls at any dose. These data suggest that while FRA1 loss enhances transformation potential, it also disrupts the cellular stress response to ionizing radiation—likely through impaired DNA damage tolerance or reduced proliferative recovery—highlighting a complex, context-dependent role for FRA1 in tumor progression.

### 3.4 FRA1 influences cell cycle response to radiation

**FIGURE 4:**
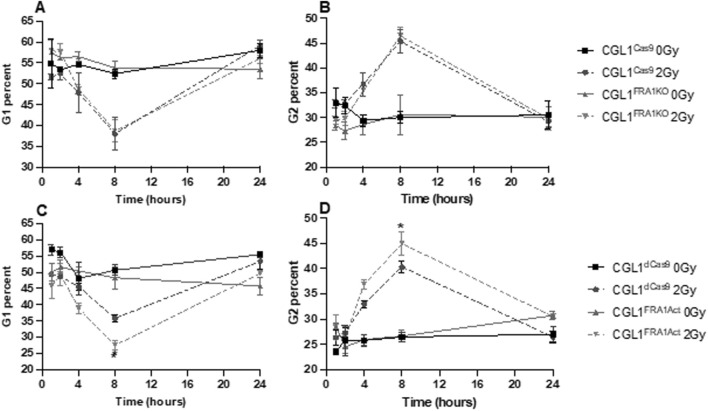
Cell cycle analysis of CGL1 cells with FRA1 overexpression or knockout following sham (0 Gy) or 2 Gy X-radiation. Panels **(A,B)** show the percentage of cells in G1 and G2 phases respectively in CGL1^Cas9^ and CGL1^FRA1KO^ cell lines. Panels **(C,D)** show G1 and G2 phase distributions respectively in CGL1^dCas9^ and CGL1^FRA1Act^ cells. Cells were collected at 1, 2, 4, 8, and 24 h post-radiation and analyzed by flow cytometry. FRA1 overexpression significantly reduced the proportion of cells in G1 phase by 23% and increased the proportion in G2 phase by 11% at 8 h post 2 Gy radiation compared to dCas9 controls (p < 0.05; *; Two-Way ANOVA). No significant differences in G1 or G2 phase distribution were observed between FRA1 knockout and CGL1^Cas9^ control cells. Data represent the mean ± SEM from 3 independent experiments.

Neoplastic transformation is closely associated with impaired cell cycle checkpoint control following genotoxic stress. Given that FRA1 modulation altered transformation frequency, we hypothesized that it may also regulate the cell cycle response to ionizing radiation. To investigate this, we performed flow cytometry–based cell cycle analysis in CGL1^Cas9^, CGL1^FRA1KO^, CGL1^dCas9^, and CGL1^FRA1Act^ cells following exposure to 2 Gy X-radiation. Cells were collected at 1, 2, 4, 8, and 24 h post-irradiation and analyzed for G0/G1, S, and G2 phase distributions using propidium iodide staining (Figure 4).

Across all cell lines, radiation exposure induced a characteristic cell cycle shift: a reduction in the proportion of cells in G1 phase and a concomitant accumulation in G2 phase. These changes peaked at 8 h post-irradiation and returned to baseline by 24 h, consistent with transient G2/M checkpoint activation in response to DNA damage. FRA1 overexpression was found to significantly alter this cell cycle response to radiation. At 8 h post-irradiation, CGL1^FRA1Act^ cells exhibited a 23% decrease in the G1 population and an 11% increase in the G2 population relative to CGL1^dCas9^ controls (p < 0.05; Two-Way ANOVA), consistent with enhanced G2/M checkpoint activation. In contrast, FRA1 knockout did not significantly alter G1 or G2 phase distribution at any time point compared to CGL1^Cas9^ controls, suggesting that loss of FRA1 does not impair checkpoint activation in this context. Across both cell lines, no significant differences were seen in the proportion of cells in S phase (data not shown). Overall, these findings suggest that FRA1 overexpression augments radiation-induced G2/M arrest, potentially improving DNA repair efficiency and contributing to its tumor-suppressive activity. Meanwhile, the absence of cell cycle perturbation in FRA1 knockout cells—despite increased transformation—suggests that their oncogenic phenotype may arise through checkpoint-independent mechanisms.

### 3.5 FRA1 modulation does not alter DNA damage recognition or canonical repair gene expression

**FIGURE 5:**
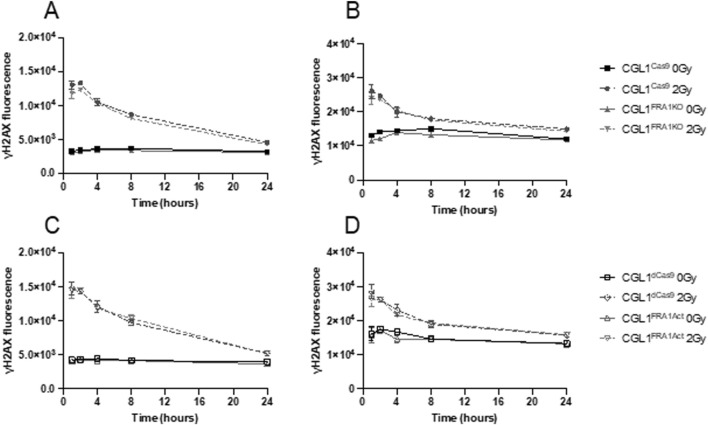
DNA damage response in CGL1 cells with FRA1 overexpression or knockout following sham (0 Gy) or 2 Gy X-radiation. γH2AX expression was measured by flow cytometry to assess DNA double-strand break formation and repair in CGL1^Cas9^ and CGL1^FRA1KO^ cells (Panels **A**,**B**), and in CGL1^dCas9^ and CGL1^FRA1Act^ cells (Panels **C,D**). Cells were exposed to sham or 2 Gy radiation and collected at 1, 2, 4, 8, and 24 h post-treatment. Mean fluorescence was measured in G0/G1 gated cells (Panels **A,C**) and G2 gated cells (Panels **B,D**). As expected, all irradiated cell lines showed a marked increase in γH2AX fluorescence intensity at early time points (1–2 h), followed by a gradual decline to near-baseline levels by 24 h. No statistically significant differences in γH2AX expression were observed between FRA1-modified and corresponding control cell lines, indicating that FRA1 manipulation does not alter the kinetics of DNA double-strand break recognition or repair. Data represent mean ± SEM from 3 independent experiments. Statistical analysis was performed using Two-Way ANOVA (p > 0.05, not significant).

**FIGURE 6:**
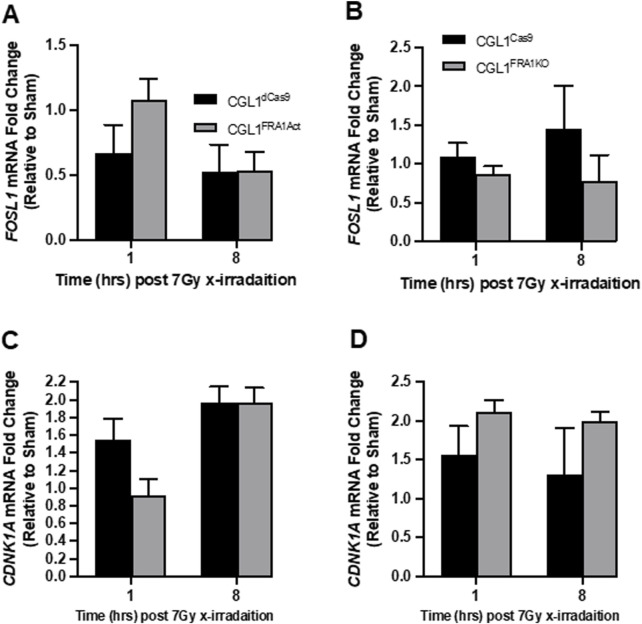
Expression analysis of radiation-responsive genes in CGL1 cells with FRA1 overexpression or knockout. CGL1^FRA1Act^, CGL1^FRA1KO^, and their respective control cell lines (CGL1^dCas9^, CGL1^Cas9^) were treated with 7 Gy X-radiation or sham exposure, and total RNA was collected at 1- and 8-h post-treatment. RT-qPCR was performed to assess the expression of FOSL1 and select DNA damage response genes including CDKN1A, CCNB1, RAD51, 53BP1, PCNA, CCND1, CHK1, CDK1, BCL2, and TNFRSF1A. No significant differences in gene expression were observed between FRA1-modified cells and their controls at either time point. For illustration, gene expression results for FOSL1 and CDKN1A are shown. **(A,B)** FOSL1 expression dynamics in overexpression and knockout contexts showed expected modulation without additional radiation-specific effects. **(C,D)** CDKN1A expression was upregulated following radiation in all lines but was not significantly altered by FRA1 status. These findings suggest that FRA1 does not broadly influence classical radiation-induced gene expression programs, and its role in radiation sensitivity and transformation may involve alternative regulatory mechanisms. Data represent mean ± SD from 3 independent experiments. Statistical significance was assessed using Two-Way ANOVA (p < 0.05).

Given the reduced clonogenic survival observed in CGL1^FRA1KO^ cells following radiation, we examined whether FRA1 influences the DNA damage response, particularly the recognition and repair of double-strand breaks (DSBs). To assess this, we first measured γH2AX expression—a well-established marker of DSB formation and repair—via flow cytometry at 1, 2, 4, 8, and 24 h after 2 Gy X-irradiation (Figure 5). Across all cell lines, radiation exposure triggered a sharp increase in γH2AX fluorescence within 1–2 h, followed by a gradual decline to near-baseline levels by 24 h, indicating efficient DSB repair over time. This kinetic profile was consistent across both FRA1-modified (CGL1^FRA1Act^ and CGL1^FRA1KO^) and corresponding control cells (CGL1^dCas9^ and CGL1^Cas9^), with no statistically significant differences observed at any time point (p > 0.05; Two-Way ANOVA). To confirm these results at the transcriptional level, we analyzed the expression of key DNA damage response genes following exposure to 7 Gy radiation. RT-qPCR was performed at 1- and 8-h post-irradiation for *FOSL1* and ten classical DNA repair and cell cycle regulatory genes, including *CDKN1A*, *CCNB1*, *RAD51*, *53BP1*, *PCNA*, *CCND1*, *CHK1*, *CDK1*, *BCL2*, and *TNFRSF1A*. No significant differences in expression were observed between FRA1-modified cell lines and their respective controls across any of the genes tested. *FOSL1* and *CDKN1A* are shown as representative examples (Figure 6). As expected, *FOSL1* expression levels reflected the overexpression and knockout contexts but were not additionally modulated by radiation exposure. *CDKN1A* was strongly induced by radiation across all cell lines, consistent with a canonical p53-mediated response, but this induction was not significantly altered by FRA1 status. Expression patterns for the remaining genes mirrored these findings, with no FRA1-dependent differences observed. Collectively, these findings suggest that although FRA1 loss sensitizes cells to radiation at the functional level, this effect is not mediated by impaired DNA damage recognition or classical DNA repair gene expression, implying that alternative regulatory pathways underlie the observed phenotypes.

### 3.6 FRA1 reprograms AP-1 complex gene expression in response to serum stimulation

**FIGURE 7:**
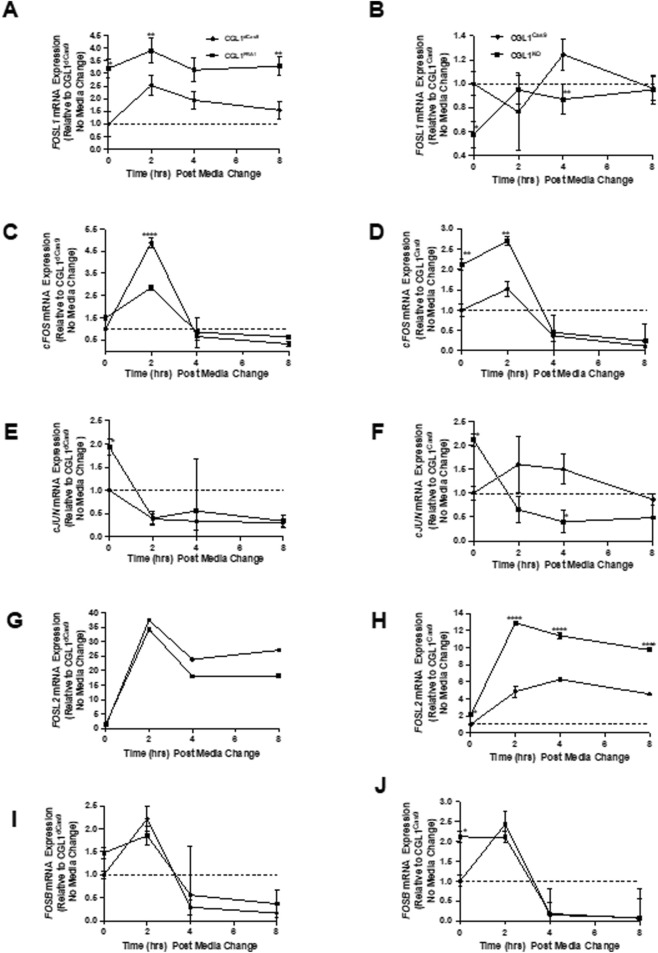
FRA1 modulation alters mRNA expression dynamics of AP-1 complex genes following serum stimulation. RT-qPCR was used to measure mRNA levels of FOSL1, cFOS, cJUN, FOSL2, and FOSB in CGL1^dCas9^, CGL1^FRA1Act^, CGL1^Cas9^, and CGL1^FRA1KO^ cell lines at 0, 2, 4, and 8 h after serum stimulation. **(A)** FOSL1 expression was induced by serum in CGL1^dCas9^ controls, peaking at 2 h and remaining elevated through 8 h, with FRA1-overexpressing cells exhibiting consistently higher expression across all time points. **(B)** FRA1 knockout cells showed a similar induction profile to CGL1^Cas9^ controls. **(C)** cFOS expression was rapidly and transiently induced at 2 h in CGL1^dCas9^ cells, returning to baseline by 4 h; this induction was markedly blunted in FRA1-overexpressing cells. **(D)** In contrast, FRA1 knockout cells displayed elevated cFOS expression at baseline and exaggerated induction following serum stimulation. **(E)** cJUN expression decreased progressively after serum stimulation in all lines; however, FRA1-overexpressing cells started with higher baseline cJUN levels. **(F)** A similar suppressive trend in cJUN expression was seen in FRA1 knockout and CGL1^Cas9^ cells. **(G)** FOSL2 expression was robustly upregulated by serum at 2 h and remained elevated at 4 and 8 h in all cells, with no significant difference in FRA1-overexpressing cells. **(H)** FRA1 knockout, however, led to a markedly exaggerated FOSL2 response throughout the time course. **(I)** FOSB expression followed an early-response pattern, peaking at 2 h and falling below baseline by 4 and 8 h in all cell types. **(J)** FRA1 knockout cells exhibited approximately 2-fold higher FOSB expression at baseline compared to CGL1^Cas9^ controls, though the overall temporal pattern remained similar. Data represent mean ± SD from 3 independent experiments. Statistical significance was determined using Two-Way ANOVA (p < 0.05).

To explore the molecular mechanisms underlying FRA1-mediated suppression of neoplastic transformation, we focused on gene expression dynamics under non-irradiated conditions. This approach was informed by prior findings showing that FRA1 modulation strongly influenced transformation frequency even in the absence of radiation, suggesting that baseline signaling alterations may drive this phenotype. Serum stimulation—achieved through the addition of fresh complete media—mimics the mitogenic conditions used in the transformation assay, enabling interrogation of early transcriptional changes relevant to transformation onset. Given that FRA1 and other AP-1 complex members are key regulators of mitogen-responsive gene expression, we profiled their transcriptional and protein expression patterns following serum stimulation to determine whether altered AP-1 dynamics contribute to the pro- or anti-transformative phenotypes observed. To this end, we first conducted a time-course RT-qPCR analysis of five AP-1 subunits—*FOSL1*, *cFOS*, *cJUN*, *FOSL2*, and *FOSB*—in FRA1-modified CGL1 cells and their respective controls at 0, 2, 4, and 8 h post-serum stimulation (Figure 7).

*FOSL1* mRNA levels were rapidly induced by serum in both control groups (CGL1^dCas9^ and CGL1^Cas9^), peaking at 2 h and remaining elevated. As expected, this response was significantly amplified in CGL1^FRA1Act^ cells, which showed consistently higher expression across all time points (p < 0.0001, Two-Way ANOVA; Figure 7A). In CGL1^FRA1KO^ cells, serum-induced *FOSL1* mRNA expression was abolished, with no significant changes over time relative to CGL1^Cas9^ controls (Figure 7B), confirming effective knockout.

Next, *cFOS* mRNA exhibited a strong, transient pulse in both control cell lines (CGL1^dCas9^ and CGL1^Cas9^), peaking at 2 h and declining thereafter. This early induction was significantly blunted in CGL1^FRA1Act^ cells (p = 0.0007; p < 0.0001 at 2 h, Tukey’s test; Figure 7C), indicating that FRA1 overexpression suppresses *cFOS* activation in response to mitogenic signals. In contrast, CGL1^FRA1KO^ cells exhibited elevated baseline *cFOS* expression and a heightened transcriptional response following serum stimulation, with significantly higher expression than CGL1^Cas9^ controls across the time course (p = 0.0029; Figure 7D). These findings suggest that FRA1 plays a critical role in limiting *cFOS* expression under both basal and stimulated conditions, and that its loss leads to enhanced *cFOS* activation during the early mitogenic response.

*cJUN* mRNA levels declined progressively following serum stimulation across all cell lines. Although no significant interaction or main effect of cell line was observed in the overexpression analysis (p = 0.67 and p = 0.32, respectively), CGL1^FRA1Act^ cells displayed higher baseline *cJUN* expression relative to CGL1^dCas9^ controls, but the serum-induced expression trajectory was similar between groups (Figure 7E). In contrast, FRA1 knockout significantly reduced *cJUN* expression, with CGL1^FRA1KO^ cells exhibiting lower transcript levels compared to CGL1^Cas9^ controls (p = 0.0157), particularly at 4 h post-stimulation (Figure 7F). These results suggest that FRA1 contributes to the maintenance of *cJUN* expression, particularly under basal conditions and during the early serum response.

*FOSL2* mRNA was robustly upregulated in response to serum stimulation in all cell lines. However, FRA1 overexpression significantly suppressed *FOSL2* induction, with CGL1^FRA1Act^ cells showing reduced expression at 2, 4, and 8 h compared to CGL1^dCas9^ controls (p < 0.0001; Figure 7G). In contrast, FRA1 knockout strongly enhanced *FOSL2* expression, with CGL1^FRA1KO^ cells exhibiting significantly elevated levels across all time points relative to CGL1^Cas9^ controls (p < 0.0001; Figure 7H). These findings suggest that FRA1 dampens *FOSL2* activation in response to mitogenic signals, potentially acting as a transcriptional brake on this AP-1 component.

*FOSB* mRNA followed a typical early-response profile, peaking at 2 h post-serum stimulation and declining by 4 and 8 h across all cell lines (p = 0.0024, Figures 7I,J). FRA1 overexpression or knockout had no significant impact on this overall expression pattern (p = 0.63). However, CGL1^FRA1KO^ cells exhibited modestly elevated baseline *FOSB* expression relative to CGL1^Cas9^ controls, though this difference did not persist post-stimulation. These results suggest that *FOSB* is largely unaffected by FRA1 modulation during the mitogenic response.

**FIGURE 8:**
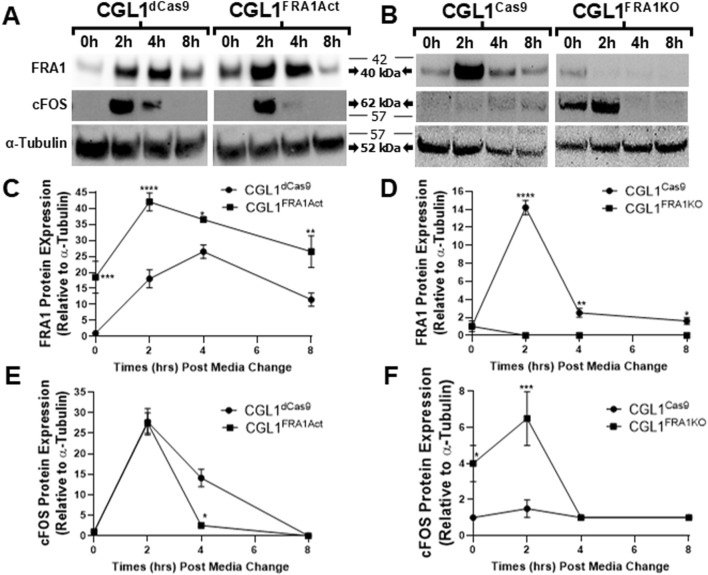
Western blot validation of FRA1 and cFOS protein expression following serum stimulation in CGL1 cells with FRA1 overexpression or knockout. CGL1^FRA1Act^, CGL1^FRA1KO^, and their respective control cell lines (CGL1^dCas9^, CGL1^Cas9^) were serum-stimulated, and protein samples were collected at 0, 2, 4, and 8 h post-treatment. **(A)** Representative Western blot images show time-dependent protein expression of FRA1 and cFOS in FRA1-overexpressing and CGL1^dCas9^ control cells. Three independent biological replicates of these blots are provided in Supplementary Figure S2. **(B)** Corresponding blots for FRA1 knockout and CGL1^Cas9^ control cells. Three independent biological replicates of these blots are provided in Supplementary Figure S3. α-Tubulin was used as a loading control. Molecular weight markers (kDa) are shown on the right; FRA1 protein was detected at ∼42 kDa, cFOS at ∼62 kDa, and α-Tubulin at ∼52 kDa. Full unprocessed blot images for all panels are provided in Supplementary Figures S3–S7. **(C–F)** Quantification of band intensities normalized to α-Tubulin is presented for each protein. **(C)** FRA1 expression increased with serum stimulation in both CGL1^dCas9^ and FRA1-overexpressing cells, with markedly higher levels in the overexpression line across all time points. **(D)** CGL1^Cas9^ control cells showed serum-induced FRA1 expression, while no detectable FRA1 protein was observed in the knockout line. **(E)** cFOS was induced at 2 h in both CGL1^dCas9^ and FRA1-overexpressing cells; however, FRA1 overexpression accelerated resolution back to baseline by 4 h, whereas controls remained elevated until 8 h **(F)** FRA1 knockout cells showed elevated cFOS levels at baseline and exaggerated responses to serum compared to CGL1^Cas9^ controls. Quantified data represent mean ± SD from 3 independent experiments. Statistical significance was determined using ANOVA (p < 0.05).

To determine whether these transcriptional trends were reflected at the protein level, we performed Western blot analysis for FRA1 and cFOS in all four cell lines at matching time points to confirm the gene expression results (Figure 8). In control cells, FRA1 protein levels increased progressively following serum stimulation. This induction was markedly enhanced in CGL1^FRA1Act^ cells, which exhibited 18.5- to 24-fold higher expression across all time points compared to CGL1^dCas9^ controls (p = 0.0001; Figures 8A,C), consistent with elevated *FOSL1* mRNA levels. In contrast, FRA1 protein levels in CGL1^FRA1KO^ cells were minimal and did not change following stimulation, consistent with successful gene disruption (Figures 8B,D). Next, cFOS protein was strongly induced at 2 h after serum stimulation in CGL1^dCas9^ control cells and remained elevated at 4 h before returning to baseline by 8 h. In contrast, CGL1^FRA1Act^ cells displayed an early induction at 2 h but failed to sustain the response, with levels already returning to baseline by 4 h (p = 0.0239; Figures 8A,E). This temporal shift is consistent with the attenuated cFOS mRNA induction observed in FRA1-overexpressing cells (Figure 7C), demonstrating that FRA1 overexpression dampens the serum-induced cFOS response. Conversely, FRA1 knockout elevated cFOS protein expression by 3-fold at baseline and 5.1-fold at 2 h relative to CGL1^Cas9^ controls (p < 0.05; Figures 8B,F), consistent with the exaggerated mRNA response seen in the knockout line (Figure 7D).

Taken together, these results demonstrate that FRA1 reprograms AP-1 complex activity at both the transcriptional and protein levels in response to mitogenic stimulation. By modulating the amplitude and timing of AP-1 subunit expression, FRA1 likely fine-tunes early transcriptional responses to external cues. This regulatory control may represent a key mechanism through which FRA1 suppresses neoplastic transformation in the CGL1 cell model.

### 3.7 Inhibition of cFOS activity does not rescue the elevated transformation phenotype in FRA1 knockout cells

Among the AP-1 complex subunits analyzed, cFOS was the most strongly dysregulated in response to FRA1 manipulation under baseline and serum-stimulated conditions, with FRA1 overexpression blunting serum-induced cFOS induction and FRA1 knockout resulting in elevated baseline and exaggerated serum-induced expression. Given prior evidence linking cFOS overexpression to cellular transformation (Muhammad et al., 2017; Wang et al., 2017), we investigated whether increased cFOS activity contributes to the heightened transformation phenotype observed in CGL1^FRA1KO^ cells. To test this, we conducted serum stimulation and assessed downstream effects on AP-1 gene expression and transformation frequency using the selective cFOS inhibitor T-5224 (Ishida et al., 2015).

**FIGURE 9:**
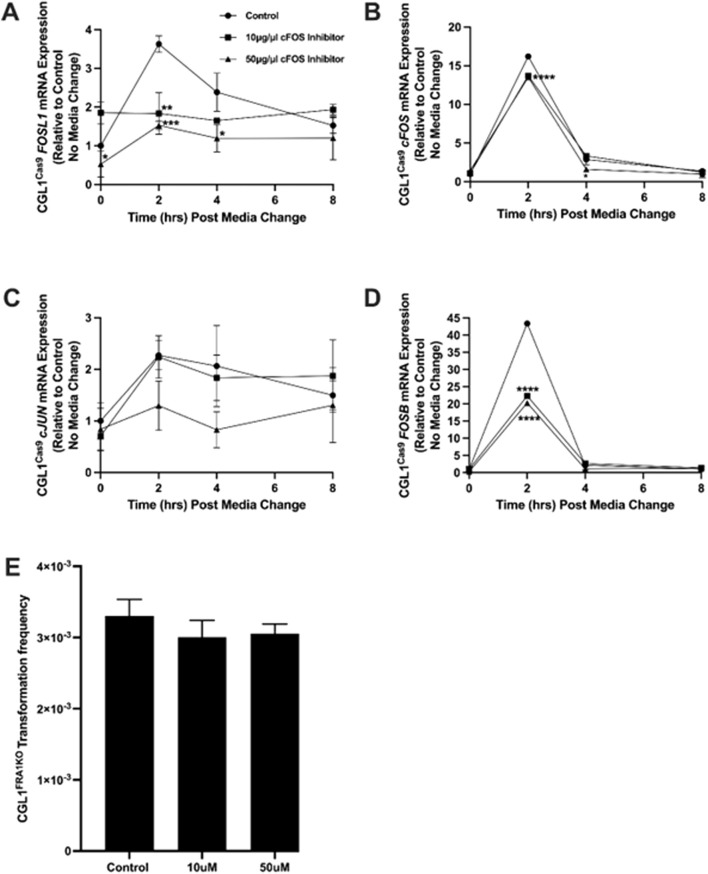
Effect of cFOS inhibition on AP-1 gene expression and neoplastic transformation in FRA1 knockout cells. CGL1^Cas9^ cells were treated with vehicle control (DMSO), 10 μg/mL, or 50 μg/mL of the cFOS inhibitor T-5224 and subjected to serum stimulation. Cells were collected at 0, 2, 4, and 8 h post-stimulation for gene expression analysis. **(A)** *FOSL1* mRNA levels were upregulated by serum at 2 h in vehicle-treated cells, but this response was significantly suppressed by both concentrations of the cFOS inhibitor, with stronger inhibition observed at 50 μg/mL. **(B)** *cFOS* expression followed a typical early-response pattern with serum but was not significantly affected by either concentration of the inhibitor. **(C)** *cJUN* mRNA levels were not notably altered by treatment, although a modest reduction was observed at 50 μg/mL. **(D)** *FOSB* expression was strongly induced by serum at 2 h and declined thereafter; this response was markedly inhibited—by more than 2-fold—at both inhibitor concentrations. **(E)** To determine if cFOS inhibition could reverse the elevated baseline transformation phenotype in FRA1 knockout cells, neoplastic transformation assays were performed in the presence of vehicle control, 10 μg/mL, or 50 μg/mL of cFOS inhibitor. No significant changes in transformation frequency were observed, suggesting that elevated cFOS activity is not the driver of enhanced neoplastic transformation in FRA1-deficient cells. Data represent mean ± SD from 3 independent experiments. Statistical significance was assessed using Two-Way ANOVA (*p* < 0.05).

To determine whether the cFOS inhibitor was functionally active under our experimental conditions, CGL1^Cas9^ control cells were pretreated with vehicle control (DMSO), 10 μg/mL, or 50 μg/mL T-5224 before serum stimulation. Cells were then collected at 0, 2, 4, and 8 h post-treatment for gene expression analysis. RT-qPCR analysis confirmed that cFOS inhibition modulated AP-1 gene expression in a dose-dependent manner (Figures 9A–D). *FOSL1* mRNA, normally induced by serum at 2 h, was significantly reduced in inhibitor-treated cells (p = 0.0007; Figure 9A), with stronger suppression observed at 50 μg/mL. As expected, *cFOS* expression itself was not significantly altered by the inhibitor (Figure 9B), consistent with the fact that its transcription is regulated by upstream mitogenic signals rather than autoregulation. Next, *cJUN* mRNA levels showed a modest reduction at 50 μg/mL, but this was not statistically significant (Figure 9C). Notably, *FOSB* mRNA—a known downstream target of cFOS—was markedly downregulated by both concentrations of the inhibitor, with more than 2-fold suppression at 2 h post-serum (p < 0.0001; Figure 9D), confirming effective inhibition of cFOS transcriptional activity.

To evaluate whether cFOS activity is required for neoplastic transformation in FRA1-deficient cells, we performed transformation assays in CGL1^FRA1KO^ cells treated with the same concentrations of T-5224 (10 or 50 μg/mL) or vehicle control. Baseline transformation frequency remained elevated in the vehicle group, as previously observed (Figure 2). However, cFOS inhibition did not reduce transformation frequency at either dose (p > 0.05, One-Way ANOVA; Figure 9E), indicating that elevated cFOS activity is not solely responsible for the oncogenic phenotype in FRA1 knockout cells. These findings suggest that while cFOS regulates transcriptional dynamics within the AP-1 network, additional or alternative mechanisms likely mediate transformation downstream of FRA1 loss.

### 3.8 FRA1 regulates growth, structural, and immune gene networks under baseline conditions

Given the lack of impact of cFOS inhibition on neoplastic transformation in FRA1-deficient cells, we sought to explore additional gene networks modulated by FRA1 that may contribute to its tumor-suppressive function. To this end, we performed baseline RNA-sequencing analysis in CGL1 cells with FRA1 knockout or overexpression, each compared to their respective CRISPR control lines (CGL1^dCas9^ and CGL1^Cas9^). This approach aimed to identify transcriptional programs regulated by FRA1 under non-irradiated, baseline conditions—paralleling the resting conditions used in our transformation assays. By focusing on the constitutive transcriptomic landscape shaped by FRA1, we aimed to uncover molecular pathways that might underpin its role in maintaining cellular homeostasis and preventing oncogenic progression. To accomplish this, we examined differentially expressed genes (DEGs) in FRA1 knockout cells relative to CGL1^Cas9^ controls (Supplementary Table S1), followed by DEG analysis in FRA1 overexpressing cells relative to CGL1^dCas9^ controls (Supplementary Table S2). Finally, we identified genes showing reciprocal regulation across both datasets, offering higher-confidence gene candidates likely to be directly or indirectly regulated by FRA1, given their consistent directional changes in both knockout and overexpression models (Table 2).

**TABLE 2:** List of genes showing reciprocal expression patterns in FRA1 knockout and overexpression CGL1 cells at baseline. To identify genes regulated by FRA1, we cross-compared the differentially expressed gene (DEG) lists from RNA-sequencing analyses of FRA1 knockout vs. versus CGL1^Cas9^ (Supplementary Table S1) and FRA1 overexpression vs. CGL1^dCas9^ (Supplementary Table S2). Genes that were significantly upregulated in one condition and downregulated in the other were identified as reciprocally expressed relative to FRA1 levels. A total of 39 genes met the inclusion criteria (fold change ≥1.5 and adjusted p-value ≤0.05) and are presented in alphabetical order. Of these, 37 genes were upregulated in FRA1 knockout cells and downregulated in FRA1 overexpressing cells, while only 2 showed the opposite trend. This strong directional bias highlights FRA1 as a key transcriptional modulator that suppresses expression of a defined gene set under baseline, non-irradiated conditions. Gene names, fold changes (F.C), and adjusted p-values (p-adj) for each comparison are provided.

Gene	CGL1^FRA1KO^ vs CGL1^Cas9^		CGL1^FRA1Act^ vs CGL1^dCas9^	
	F.C.	p-adj	F.C.	p-adj
*ACTA2*	2.84	4.41E-17	−1.80	1.26E-02
*CCL5*	8.88	4.31E-57	−9.27	8.67E-10
*CD24*	2.00	3.95E-04	−2.08	4.55E-03
*EPAS1*	3.22	6.25E-10	−2.26	2.29E-02
*FN1*	1.93	9.31E-13	−1.83	3.99E-02
*GBP1*	3.21	7.64E-19	−1.90	1.35E-03
*HERC5*	2.94	8.05E-17	−2.26	2.82E-04
*HHAT*	2.09	1.23E-03	−2.24	6.27E-03
*HLA-B*	1.91	9.09E-69	−1.76	4.82E-40
*ICAM1*	2.21	2.08E-19	−1.53	1.68E-03
*IFI44*	1.74	3.10E-16	−1.53	2.61E-05
*IFI6*	2.50	1.99E-18	−1.57	1.84E-02
*IFIH1*	2.47	3.57E-10	−2.23	9.57E-04
*IFIT1*	1.80	4.67E-29	−2.39	1.37E-42
*IFIT3*	2.22	4.89E-46	−2.24	8.49E-31
*IGFBP4*	4.36	1.88E-30	−2.18	2.01E-03
*IGFBP6*	2.67	1.92E-25	−1.68	5.20E-04
*IGFBP7*	1.65	7.56E-13	−2.07	1.50E-19
*IL32*	2.66	7.20E-18	−2.76	1.63E-04
*ISG15*	2.07	1.53E-26	−2.45	4.82E-27
*KRT17*	6.27	8.20E-79	−4.08	5.28E-21
*L1CAM*	1.84	1.20E-21	−2.02	4.42E-12
*LIMS2*	4.10	1.04E-02	−12.21	5.00E-03
*LOXL1*	1.69	7.72E-12	−1.75	5.44E-11
*MMP2*	2.55	2.61E-20	−1.76	9.00E-05
*MMP24*	3.36	1.46E-19	−1.87	3.87E-03
*NNMT*	1.57	7.22E-07	−1.94	1.59E-06
*PALM*	2.27	1.41E-11	−1.86	3.72E-03
*PAPPA*	6.52	3.62E-20	−3.05	2.42E-02
*PLAAT4*	3.44	9.81E-25	−3.53	3.56E-08
*PLAU*	2.15	4.93E-13	−1.69	1.61E-03
*POSTN*	1.79	2.23E-17	−1.78	3.46E-17
*PSMB9*	2.37	9.71E-10	−1.92	1.65E-02
*RNF24*	1.78	3.87E-04	−1.52	3.23E-02
*STRA6*	6.74	3.40E-39	−4.05	2.72E-07
*TMPRSS15*	−3.76	5.32E-04	2.12	1.73E-02
*TRIM47*	1.57	4.23E-03	−1.66	1.14E-02
*UBE2L6*	1.77	6.60E-05	−1.68	3.12E-02
*ZNF385B*	−1.62	7.51E-04	1.53	5.20E-04

In CGL1^FRA1KO^ cells, a total of 387 DEGs were identified relative to CGL1^Cas9^ controls relative to CGL1^Cas9^ controls (Supplementary Table S1), with 339 upregulated and 48 downregulated transcripts. The transcriptional profile of FRA1 knockout cells revealed significant upregulation of genes involved in extracellular matrix remodeling and cellular adhesion, including *MMP2*, *MMP24*, several collagen isoforms (*COL1A1*, *COL4A1*, *COL5A1*), fibronectin (*FN1*), *ICAM1*, and *CD24*. This pattern suggests enhanced structural and adhesive alterations in the absence of FRA1. FRA1 knockout also led to increased expression of hypoxia-associated signaling components such as *EPAS1* (HIF-2α) and prostaglandin pathway genes including *PTGS1* and *PTGER4*. Developmental and growth-regulatory genes such as *FGFR2*, *FGFR4*, *WNT5A*, *SOX9*, *FZD1*, and *FZD4* were also upregulated, indicating a potential shift toward proliferative and morphogenic programs. In addition to structural and growth-related genes, numerous immune response genes were strongly induced in the FRA1 knockout cell line. Among these were several interferon-stimulated genes and innate immune regulators, including *IFI27*, *IFI30*, *IFI44*, *IFI44L*, *IFI6*, *IFIH1*, *IFIT1*, *IFIT3*, *ISG15*, *IL6*, and *IL32*. This transcriptional signature suggests that FRA1 normally restrains basal activation of antiviral and inflammatory gene networks. Concurrently, downregulation of multiple genes associated with the TGFβ signaling pathway was observed, including *TGFB2*, *TGFBI*, and *TGFBR1*, alongside decreased expression of several semaphorin family members such as *SEMA3A*, *SEMA4B*, and *SEMA5A*. These changes may reflect disruptions in cellular differentiation, migration, or immunomodulatory signaling following FRA1 loss.

In contrast, FRA1 overexpression resulted in a more targeted transcriptional program, with only 216 DEGs identified relative to CGL1^Cas9^ controls, of which 210 were downregulated (Supplementary Table S2). This bias toward transcriptional repression reinforces the role of FRA1 as a predominantly suppressive regulator under steady-state conditions. Notably, many of the genes downregulated by FRA1 overexpression overlapped with those upregulated in FRA1 knockout cells, including *FN1*, *MMP2*, *MMP24*, *CD24*, *EPAS1*, *PTGS1*, *IL32*, *IFI44*, *IFIT1*, and *ISG15*, reinforcing the idea that FRA1 actively limits the expression of genes implicated in adhesion, hypoxia, inflammation, prostaglandin signaling and growth.

To identify genes most tightly regulated by FRA1, we cross-referenced the DEG datasets from the knockout and overexpression conditions. This analysis revealed 39 genes that were reciprocally expressed—meaning significantly upregulated in FRA1 knockout cells and downregulated in FRA1 overexpressing cells, or *vice versa* (Table 2). Of these, 37 genes were upregulated in the absence of FRA1 and repressed when FRA1 was overexpressed, underscoring a clear directional bias in FRA1-dependent gene regulation. Among these were key interferon response genes such as *IFI44*, *IFI6*, *IFIH1*, *IFIT1*, *IFIT3*, *ISG15*, and *IL32*. In addition, genes associated with extracellular matrix remodeling and cellular adhesion, including *MMP2*, *MMP24*, *FN1*, *CD24*, and *ICAM1*, were also reciprocally regulated, as were growth and hypoxia-associated transcripts such as *EPAS1* and *IGFBP4*. Several other genes involved in cell communication and remodeling, such as *POSTN*, *PLAUR*, and *STRA6*, and regulators of TGF signaling like *TGFBI* and *TGFB2*, further contributed to this signature.

Taken together, these transcriptomic findings reveal that FRA1 plays a central role in maintaining the basal transcriptional equilibrium of CGL1 cells by repressing a broad network of genes involved in ECM organization, hypoxia signaling, cellular proliferation and immune activation.

### 3.9 FRA1 modulates the early serum-induced transcriptional response in CGL1 cells

In order to better understand the cellular dynamics contributing to neoplastic transformation in our assay system, we examined not only baseline transcriptional programs, but also how CGL1 cells respond to mitogenic stimulation. During the transformation assay, the cells were regularly replenished with fresh media to support cell survival and proliferation over the 3-week assay period. This recurring exposure to growth factors likely plays an important role in modulating transformation potential, particularly in the context of FRA1 perturbation. As such, we sought to determine whether FRA1 influences the transcriptional response to serum stimulation, which could reveal early regulatory pathways that either promote or suppress transformation. To this end, we performed RNA-sequencing in CGL1^FRA1KO^, CGL1^FRA1Act^, and their respective control cell lines (CGL1^Cas9^ and CGL1^dCas9^) 2 h following serum stimulation, and compared these profiles to their matched baseline expression states. This analysis allowed us to identify serum-responsive transcriptional programs that are differentially modulated by FRA1, thereby providing mechanistic insight into how FRA1 may influence oncogenic susceptibility in this system.

To characterize the early mitogenic response in wild-type CGL1 cells, we first examined the transcriptional profile of pooled control lines (CGL1^Cas9^ and CGL1^dCas9^) following serum stimulation. As no significant transcriptional differences were observed between these control lines under baseline conditions, their serum-stimulated datasets were merged to generate a unified reference. RNA-sequencing performed 2 h post-serum stimulation revealed a total of 639 DEGs, consisting of 337 upregulated and 302 downregulated transcripts (Supplementary Table S3). This dataset represents the early serum-induced gene expression program in CGL1 cells and provides a baseline against which the effects of FRA1 manipulation on serum responsiveness were compared.

Building on this reference dataset, we next assessed how FRA1 modulates the transcriptional response to mitogenic stimulation by examining the serum-induced gene expression profiles of CGL1^FRA1KO^ and CGL1^FRA1Act^ cells relative to their respective baseline states. In FRA1-deficient cells, serum stimulation elicited a robust transcriptional response, with 480 DEGs identified, including 364 upregulated and 116 downregulated transcripts (Supplementary Table S4). FRA1-overexpressing cells also exhibited a pronounced mitogenic response, with 550 DEGs identified, comprising 352 upregulated and 198 downregulated transcripts (Supplementary Table S5). These datasets indicate that FRA1 manipulation does not eliminate the capacity of CGL1 cells to respond to mitogenic cues but significantly alters the composition and magnitude of the serum-induced gene expression program.

To directly assess how FRA1 alters the transcriptional response to mitogenic stimulation, we compared RNA-sequencing profiles from CGL1^FRA1KO^ and CGL1^FRA1Act^ cells to their respective control lines (CGL1^Cas9^ and CGL1^dCas9^) following 2 h of serum exposure. This analysis revealed that FRA1 knockout markedly intensified the early serum response, with 223 DEGs (172 upregulated and 51 downregulated; Supplementary Table S6), whereas FRA1 overexpression led to a more constrained transcriptional output, with only 107 DEGs (75 downregulated and 32 upregulated; Supplementary Table S7). To further refine this analysis and highlight the most robust FRA1-responsive targets, we cross-referenced these datasets to identify genes that were reciprocally expressed—those significantly altered in opposite directions across both models. While reciprocal changes were assessed in both directions, all 24 genes that met the criteria were consistently upregulated in FRA1 knockout cells and downregulated in FRA1-overexpressing cells, underscoring a strong directional effect. This set of genes, presented in Table 3, represents a core serum-responsive signature that is tightly suppressed by FRA1 during early mitogenic stimulation. Among the 24 reciprocally expressed genes identified in Table 3, many were directly relevant to oncogenic processes and exhibited clear FRA1-dependent regulation during early serum stimulation. Structural and matrix remodeling genes such as *POSTN*, *PLAUR*, *ICAM1*, and *CD24* were upregulated in FRA1 knockout cells and suppressed with FRA1 overexpression, suggesting that FRA1 constrains adhesive and migratory programs that may facilitate transformation. Similarly, growth and developmental regulators, including *EPAS1*, *IGFBP4*, *PLAU*, and *ZC3HAV1*, followed this same directional pattern, reinforcing the role of FRA1 in restraining early proliferative and morphogenic transcriptional outputs.

**TABLE 3:** List of genes showing reciprocal expression patterns in FRA1 knockout and overexpression CGL1 cells 2 h post serum stimulation. To identify serum-responsive genes regulated by FRA1, we cross-compared DEGs from CGL1^FRA1KO^ vs. CGL1^Cas9^ and CGL1^FRA1Act^ vs. CGL1^dCas9^ cells 2 h post serum stimulation. Genes that were significantly upregulated in one condition and downregulated in the other (fold change ≥1.5, adjusted p-value ≤0.05) were defined as reciprocally expressed. A total of 24 genes met these criteria, all showing upregulation in FRA1 knockout cells and downregulation in FRA1-overexpressing cells. This consistent directional pattern highlights FRA1 as a suppressor of a defined gene set during the early serum response. Gene names, fold changes (F.C.), and adjusted p-values (p-adj) for each comparison are provided.

Gene	CGL1^FRA1KO^ vs CGL1^Cas9^		CGL1^FRA1Act^ vs CGL1^dCas9^	
	F.C.	p-adj	F.C.	p-adj
*ANPEP*	1.83	6.23E-08	−2.16	1.28E-02
*APOL6*	2.18	8.84E-13	−1.97	1.40E-02
*CCL5*	16.16	7.92E-51	−7.22	2.13E-03
*CPA4*	1.70	2.69E-14	−1.73	1.75E-04
*CYP1B1*	1.71	9.55E-22	−1.54	3.59E-03
*DUSP4*	2.73	9.09E-49	−2.25	9.74E-05
*FOXC2*	1.63	4.61E-02	−2.74	1.24E-03
*HERC5*	5.36	1.89E-37	−2.25	3.10E-02
*HLA-B*	1.93	1.83E-45	−1.68	2.93E-06
*ICAM1*	1.70	1.15E-06	−2.14	1.40E-02
*IFIT1*	3.32	1.82E-38	−2.03	1.76E-05
*IFIT3*	3.74	4.49E-110	−3.02	3.95E-02
*IGFBP7*	1.78	1.17E-08	−1.84	1.02E-03
*IL6*	8.04	8.17E-28	−5.79	2.76E-02
*KRT17*	4.43	9.67E-105	−4.44	7.70E-24
*L1CAM*	1.90	4.30E-06	−2.81	1.51E-06
*MVP*	1.67	6.04E-03	−2.03	4.06E-03
*NTN4*	1.61	1.63E-04	−1.89	2.13E-03
*PAPPA*	4.07	7.19E-07	−12.42	3.11E-03
*SAMD9L*	4.05	1.44E-03	−123.59	5.37E-03
*SVIL*	1.83	1.97E-04	−1.90	3.90E-02
*TRIM22*	3.64	1.08E-02	−94.90	2.00E-02
*ZC3HAV1*	1.97	5.77E-28	−1.53	9.47E-03
*ZNFX1*	1.57	3.70E-13	−1.58	2.51E-02

Although several of these targets overlapped with baseline FRA1-regulated genes, their continued modulation following serum exposure underscores FRA1’s persistent influence in suppressing transformation-relevant pathways, both constitutively and in response to mitogenic cues. Notably, immune and inflammatory mediators such as *CCL5*, *IFI6*, *IFIH1*, *IFI44*, and *ISG15* were also induced in the knockout and repressed in the overexpression model. These findings support a broader role for FRA1 in attenuating innate immune activation during early growth stimulation, consistent with its previously observed immunomodulatory functions under baseline conditions.

Collectively, these findings indicate that FRA1 serves as a fine-tuning regulator of mitogen-induced gene networks, selectively repressing transcriptional programs linked to growth, matrix remodeling, hypoxia, and inflammation. The coordinated upregulation of these pathways in FRA1-deficient cells, and their suppression upon FRA1 overexpression, aligns with the observed increase and decrease in transformation frequency, respectively, reinforcing FRA1’s role in modulating oncogenic susceptibility.

## 4 Discussion

This study demonstrates that FRA1 plays a critical tumor-suppressive role in the CGL1 human hybrid cell model by modulating neoplastic transformation and radiation responses through transcriptional regulation of stress and mitogenic pathways. FRA1 overexpression significantly suppressed both spontaneous and radiation-induced transformation, while FRA1 knockout markedly enhanced transformation frequency under both conditions. These phenotypic changes occurred without impacting baseline cell growth or morphology. Clonogenic assays revealed that FRA1 knockout sensitized cells to ionizing radiation. Notably, FRA1 did not influence DNA double-strand break recognition or classical repair gene expression, as evidenced by comparable γH2AX kinetics and transcriptional profiles of DNA repair genes across all lines. Instead, FRA1 enhanced G2/M checkpoint activation post-radiation, suggesting that its protective effect is mediated via cell cycle regulation. Transcriptomic profiling further revealed that FRA1 loss and overexpression result in reciprocal gene expression changes, particularly affecting AP-1 subunit composition and downstream pathways linked to proliferation, extracellular matrix remodeling, hypoxia, and immune signaling. These findings support a model in which FRA1 suppresses transformation by fine-tuning stress-responsive transcriptional networks and maintaining AP-1 complex balance.

### 4.1 FRA1 in neoplastic transformation

The findings from this study highlight FRA1 as a critical tumor suppressor regulating neoplastic transformation in the CGL1 hybrid cell model. FRA1 overexpression significantly reduced both spontaneous and radiation-induced transformation, whereas FRA1 knockout markedly enhanced transformation frequencies under similar conditions. These results corroborate previous observations from our group, which identified frequent loss or epigenetic silencing of *FOSL1* in radiation-induced neoplastic transformants derived from CGL1 cells (Pirkkanen et al., 2019; Pirkkanen et al., 2023). Importantly, re-expression of FRA1 effectively suppressed tumor formation *in vivo* (Pirkkanen et al., 2023). These findings emphasize FRA1’s protective role during early stages of carcinogenesis, contrasting its established association with tumor progression, metastasis, and therapy resistance in advanced malignancies.

These findings are particularly relevant given that the CGL1 system is derived in part from HeLa cervical carcinoma cells, making it a suitable model for cervical cancer biology. Multiple independent studies have shown that FRA1 acts as a tumor suppressor in cervical cancer, where its loss or mutation is associated with increased tumor progression (Soto et al., 2000; Prusty and Das, 2005; Mendoza et al., 2011; Xiao et al., 2015; Jiang et al., 2020; Jiang et al., 2021). Thus, the increased transformation frequency observed in FRA1-deficient CGL1 cells is consistent with the established tumor-suppressive role of FRA1 in cervical cancer models.

The dual roles of FRA1 in carcinogenesis—shaped by cellular context and environmental factors—are well established (Ramos-Nino et al., 2007; Liu et al., 2015; Sedef Iskit, 2015; Zhang et al., 2016; Song et al., 2021; Casalino et al., 2022; Schneeweis et al., 2022; Zeng et al., 2022; Casalino et al., 2023). While FRA1 is typically associated with aggressive behavior in advanced cancers such as breast, lung, and colorectal malignancies (Adiseshaiah et al., 2007; Adiseshaiah et al., 2008; Lu et al., 2012; Vaz et al., 2012; Desmet et al., 2013; Motrich et al., 2013; Yang et al., 2013; Moquet-Torcy et al., 2014; Borrego-Soto et al., 2015; Qiao et al., 2016; Zhong et al., 2016; Vallejo et al., 2017; Annis et al., 2018; Franco et al., 2018; Helm and Rudel, 2020; Song et al., 2021; Casalino et al., 2023; Hu et al., 2023; Manios et al., 2025), accumulating evidence suggests a distinct tumor-suppressive function in pre-malignant or early-stage cancer contexts (Grotsch et al., 2014; Zhang et al., 2020). Our current findings support this nuanced role, demonstrating that FRA1 restrains the initial stages of transformation, possibly by regulating transcriptional networks that influence cellular proliferation, extracellular matrix remodeling, inflammation, and stress responses (Young et al., 2002; Casalino et al., 2007; Reuter et al., 2010; Hannemann et al., 2019; Xiao et al., 2019; Cao et al., 2021; Bhosale et al., 2022; Chen et al., 2023; Qian et al., 2024).

Notably, FRA1 knockout cells exhibited significantly elevated transformation frequencies even without external stressors like radiation, highlighting FRA1’s intrinsic role in preventing spontaneous neoplastic progression. Conversely, FRA1 overexpression effectively limited transformation under both baseline and radiation-induced scenarios, reinforcing its active role in maintaining cellular homeostasis and genomic stability. Collectively, this study places FRA1 at the center of regulatory mechanisms that safeguard against early oncogenic events. These insights deepen our understanding of FRA1’s context-dependent functionality and underline the importance of precisely dissecting AP-1 complex roles throughout tumorigenesis, ultimately guiding potential therapeutic interventions targeting early cancer progression.

### 4.2 Cell cycle and DNA damage responses: implications for neoplastic transformation

The interplay between cell cycle regulation and DNA damage responses critically influences neoplastic transformation, particularly following genotoxic stressors such as ionizing radiation. Our study provides mechanistic insights into how FRA1 modulates these processes distinctly through knockout and overexpression contexts, influencing both radiation sensitivity and cell cycle dynamics without affecting canonical DNA repair pathways. Notably, FRA1 knockout enhanced cellular radiosensitivity, as evidenced by significantly impaired clonogenic survival post-irradiation (Figure 3) yet showed no significant alteration in DNA DSB recognition or repair kinetics as indicated by comparable γH2AX resolution across experimental conditions (Figure 5). In contrast, FRA1 overexpression did not significantly affect survival outcomes but markedly increased G2/M checkpoint activation post-radiation, characterized by increased G2 phase arrest and reduced G1 phase populations (Figure 4). These findings indicate that FRA1-mediated effects on neoplastic transformation and radiation responses predominantly involve alterations in cell cycle checkpoint regulation rather than direct modulation of DNA repair machinery.

The distinct outcomes observed between FRA1 knockout and overexpression models imply divergent mechanisms underlying their impact on cellular responses to genotoxic stress. Cell cycle checkpoints, particularly the G2/M checkpoint, play a pivotal role in ensuring genomic integrity by delaying cell cycle progression, allowing time for DNA repair or triggering apoptosis in severely damaged cells (Hamdi et al., 2008; Christmann and Kaina, 2013; Borrego-Soto et al., 2015; Srivastava and Raghavan, 2015; Chatterjee and Walker, 2017; Helm and Rudel, 2020; Huang and Zhou, 2020; Yang et al., 2020). FRA1-overexpressing cells exhibited enhanced G2/M checkpoint activation (Figure 4), a phenotype likely facilitating more efficient repair or apoptotic clearance of damaged cells, thus protecting against radiation-induced transformation. Enhanced G2 checkpoint enforcement is known to limit genomic instability and mutation accumulation, processes intrinsically linked to reduced transformation potential (Gabrielli et al., 2012; Fernando et al., 2021). In contrast, FRA1 knockout cells exhibited radiosensitivity not attributable to impaired canonical DNA repair (Figure 5), suggesting alternative molecular mechanisms contributing to increased transformation potential. Analysis of baseline RNA-seq data presented in Supplementary Tables S1, S2 provides clues to these alternative pathways. Specifically, FRA1 knockout led to significant upregulation of genes implicated in cellular stress responses, extracellular matrix remodeling, inflammatory signaling, and hypoxia pathways, all factors contributing to enhanced cellular proliferation, migration, and survival under genotoxic stress. Notably, extracellular matrix remodeling and inflammatory signaling have been extensively associated with genomic instability and neoplastic progression through the induction of proliferative and anti-apoptotic signaling cascades (Mendonca et al., 2005; Casalino et al., 2007; Srivastava and Raghavan, 2015; Helm and Rudel, 2020; Li et al., 2020; Fernando et al., 2021; Yoshioka et al., 2021; Waters and Spratt, 2024).

Furthermore, despite FRA1 knockout not altering cell cycle distributions post-radiation exposure (Figure 4), the transcriptional activation of proliferative and inflammatory gene networks may facilitate survival of genomically unstable cells, promoting neoplastic transformation. Previous studies suggest that elevated expression of pro-inflammatory and growth-regulatory genes, such as those identified in our FRA1 knockout cells (e.g., *FN1*, *MMP2*, *EPAS1*, and multiple interferon-stimulated genes), can potentiate neoplastic transformation independently of direct DNA repair impairment (Kay et al., 2019; Helm and Rudel, 2020). This transcriptional reprogramming likely contributes to a heightened oncogenic environment, driving transformation despite adequate DNA damage resolution.

Conversely, FRA1 overexpression consistently repressed these proliferative, inflammatory, and hypoxia-driven transcriptional programs at baseline, further supporting a protective role mediated by transcriptional control over critical oncogenic and stress-responsive gene networks. FRA1-driven alterations in cell cycle dynamics, specifically enhanced checkpoint arrest, may synergize with these transcriptional suppressive effects to robustly limit neoplastic progression following genotoxic stress.

Collectively, these observations delineate FRA1’s role as a critical regulator of cell cycle checkpoint dynamics rather than canonical DNA repair pathways, with transcriptional regulation underpinning its distinct impact on radiosensitivity and neoplastic transformation. By modulating gene networks linked to proliferation, inflammatory signaling, and extracellular matrix dynamics, FRA1 influences the cellular threshold for genomic instability and oncogenic progression. Future research should focus on further elucidating how FRA1 interacts with checkpoint regulators and transcriptionally controlled oncogenic pathways, potentially identifying novel therapeutic targets to mitigate radiation-induced transformation and improve cancer treatment outcomes.

### 4.3 FRA1 modulates AP-1 complex composition and activity

The AP-1 transcription factor complex is a dynamic regulator of gene expression, influencing diverse cellular processes, including proliferation, differentiation, and stress responses (Bakiri et al., 2007; Hamdi et al., 2008; Baan et al., 2010; Diesch et al., 2014; Godde et al., 2014; Grotsch et al., 2014; Bakiri et al., 2015; Zhang et al., 2016; Comandante-Lou et al., 2022; Schneeweis et al., 2022; Sobolev et al., 2022; Al-Khayyat et al., 2023; Guneri-Sozeri et al., 2023). Our study demonstrates that FRA1 significantly alters AP-1 complex composition and activity, specifically modulating the expression dynamics of key subunits such as cFOS, cJUN, FOSL2, and FOSB, with notable consequences for neoplastic transformation (Figures 7, 8). This complex modulation may underlie FRA1’s observed tumor-suppressive function.

The AP-1 complex is predominantly composed of FOS (cFOS, FRA1, FRA2, FOSB) and JUN family proteins (cJUN, JUNB, JUND), whose composition dictates target gene specificity and transcriptional activity (Lee et al., 1995; Bakiri et al., 2002; Verde et al., 2007; Baan et al., 2010; Bozec et al., 2010; Grotsch et al., 2014; Moquet-Torcy et al., 2014; Ye et al., 2014; Grotsch et al., 2014; Qiao et al., 2016; de Francisco et al., 2018; Garces de Los Fayos Alonso et al., 2018; Atsaves et al., 2019; Tolza et al., 2019). Our analysis revealed that FRA1 overexpression consistently suppressed cFOS induction following mitogenic stimulation at both mRNA and protein levels, whereas FRA1 knockout markedly increased cFOS basal expression and serum responsiveness (Figures 7C,D, 8E,F). This reciprocal regulation suggested that elevated cFOS might contribute significantly to the increased neoplastic transformation observed upon FRA1 loss, given cFOS’s established pro-oncogenic role in cellular proliferation, transformation, and tumor progression (Mahner et al., 2008; Muhammad et al., 2017; Yan et al., 2025). Indeed, previous studies have documented cFOS as a potent oncogenic driver implicated in the initiation and progression of multiple cancer types, including breast, colorectal, and ovarian cancers (Mahner et al., 2008; Racca et al., 2019; Gui et al., 2024). Increased cFOS expression is frequently associated with poor prognosis, enhanced proliferation, and aggressive tumor phenotypes (Muhammad et al., 2017). Therefore, we hypothesized that heightened cFOS activity in FRA1 knockout cells might directly mediate their enhanced neoplastic transformation potential.

To directly test this hypothesis, we employed the selective cFOS inhibitor T-5224, known to disrupt cFOS DNA binding and downstream target gene activation (Makino et al., 2017). Although treatment with T-5224 effectively attenuated cFOS-driven transcription, as confirmed by reduced expression of validated downstream targets such as *FOSB* and *FOSL1* (Figures 9A–D), we observed no corresponding reduction in transformation frequency in FRA1 knockout cells (Figure 9E). These results indicate that elevated cFOS alone is insufficient to drive the enhanced transformation observed upon FRA1 loss, implying involvement of other compensatory or parallel oncogenic pathways.

However, potential pitfalls exist regarding our reliance on cFOS inhibition to elucidate its functional role. First, pharmacological inhibition using T-5224 may not fully ablate cFOS transcriptional activity, as residual cFOS activity might remain sufficient to sustain the transformed phenotype (Makino et al., 2017). Furthermore, T-5224 specifically targets the DNA-binding activity of cFOS-containing AP-1 complexes, but may not effectively inhibit non-transcriptional or indirect oncogenic effects of cFOS (Perrotti et al., 2004; Tang et al., 2023). Consequently, genetic approaches such as targeted cFOS knockdown or knockout might yield more definitive conclusions regarding cFOS’s functional necessity in the transformation phenotype observed in FRA1 knockout cells.

Additionally, our data suggested alternative AP-1 subunits could mediate compensatory mechanisms, maintaining the transformed phenotype independent of cFOS. FOSB, another member of the FOS family robustly induced during early mitogenic response, represents an alternative candidate potentially involved in FRA1-dependent transformation mechanisms (Ramachandran et al., 2011). Indeed, FOSB has been implicated in regulating proliferation, differentiation, and survival, with emerging evidence indicating a context-dependent oncogenic or tumor-suppressive role similar to FRA1 itself (Haasper et al., 2008; Sugita et al., 2016; Zhang H. et al., 2024). Therefore, investigating FOSB activity following FRA1 modulation may provide additional mechanistic insights into AP-1 dynamics contributing to neoplastic transformation.

Further complexity arises from observed changes in cJUN and FOSL2 dynamics following FRA1 modulation. FRA1-overexpressing cells displayed sustained cJUN expression and suppressed FOSL2 induction, whereas FRA1 knockout cells showed reduced cJUN and increased FOSL2 responsiveness (Figures 7E–H, 8G,H). Considering that cJUN is involved in both tumor-suppressive (via apoptosis induction and differentiation) and oncogenic roles (via proliferation and migration), and FOSL2 is often implicated in tumorigenesis and invasion, these reciprocal changes may contribute substantially to the transformation phenotype independently or synergistically with cFOS (Bozec et al., 2010; Shetty et al., 2022).

Collectively, our findings underscore that FRA1 extensively reprograms AP-1 complex composition and activity beyond cFOS alone, influencing multiple subunits with complex, potentially context-dependent functional consequences. Although cFOS initially appeared a prominent mechanistic candidate, pharmacological inhibitor studies revealed limitations, highlighting the necessity for genetic validation and broader consideration of alternative subunits such as FOSB, FOSL2, and cJUN. Future studies employing targeted genetic manipulation, such as CRISPR-mediated knockdown or knockout of individual AP-1 subunits, combined with comprehensive transcriptional and functional analyses, are warranted to fully delineate the complex interplay among AP-1 family members and their collective influence on FRA1-mediated suppression of neoplastic transformation.

### 4.4 FRA1 regulates baseline and mitogen-induced transcriptional networks

Our transcriptomic analyses provide comprehensive insight into the extensive regulatory role of FRA1 in both baseline and serum-induced transcriptional responses, highlighting its function as a pivotal transcriptional fine-tuner in the context of early neoplastic progression. FRA1 manipulation resulted in significant reciprocal gene expression alterations affecting diverse cellular pathways, including growth regulation, extracellular matrix (ECM) remodeling, immune signaling, epithelial-mesenchymal transition (EMT), and hypoxia response networks.

At baseline, FRA1 knockout induced robust transcriptional activation of genes prominently linked to cell proliferation, adhesion, and extracellular matrix dynamics, including *FN1*, *MMP2*, *MMP24*, *CD24*, *ICAM1*, and *POSTN* (Supplementary Table S1). These genes are strongly associated with tumor invasiveness, metastatic potential, and enhanced cellular survival under stress (Jaggupilli and Elkord, 2012; Cooper and Giancotti, 2019; Almutairi et al., 2023). Conversely, FRA1 overexpression robustly repressed these same genes, underscoring FRA1’s active role in restraining the expression of genes critical for tumor progression. Previous studies have extensively documented that elevated expression of ECM remodeling factors such as FN1 and MMP2 facilitates metastatic dissemination by promoting cellular adhesion, migration, and invasion (Lou et al., 2013; Murray, 2024). Our current findings supported and provided a mechanistic understanding of our previous *in vivo* observations, where reintroduction of FRA1 into genomically unstable CGL1 transformants effectively suppressed tumor formation (Pirkkanen et al., 2023). Thus, FRA1 mediated suppression of these pathways may represent a crucial early tumor-suppressive mechanism limiting pro-invasive cellular behaviors.

EMT-associated genes such as *POSTN*, *EPAS1* (HIF-2α), *IGFBP4*, and various hypoxia and angiogenesis regulators were notably upregulated upon FRA1 knockout and consistently repressed upon FRA1 overexpression (Tables 2 and 3). The activation of EMT-related signaling pathways is central to enhanced invasiveness, therapeutic resistance, and increased metastatic potential (Diesch et al., 2014; Bakiri et al., 2015; Tripathi and Garg, 2018; Lu and Kang, 2019; Usman et al., 2021; Casalino et al., 2022; Casalino et al., 2023; Liu et al., 2023; Youssef et al., 2024). *EPAS1*, in particular, has been strongly associated with hypoxia-induced EMT and tumor aggressiveness across various malignancies (Song et al., 2020). FRA1-mediated repression of *EPAS1* and related hypoxic signaling networks likely represents another crucial mechanism through which FRA1 exerts tumor-suppressive effects by preventing cellular adaptation to pro-tumorigenic hypoxic stress conditions.

Notably, immune and inflammatory signaling pathways, including interferon-stimulated genes (e.g., *IFIT1*, *IFIT3*, *ISG15*, *IL32*, and *IFIH1*), were significantly activated in FRA1 knockout cells and reciprocally repressed upon FRA1 overexpression (Tables 2, 3). Elevated inflammatory and interferon signaling is increasingly recognized as a critical driver of tumorigenesis, promoting genomic instability, chronic proliferative signaling, and resistance to apoptosis (Reuter et al., 2010; Hannemann et al., 2019; Cao et al., 2021; Bhosale et al., 2022; Chen et al., 2023; Youssef et al., 2024). FRA1’s ability to restrain inflammatory signaling pathways, therefore, may represent a protective mechanism against inflammation-driven tumor initiation and progression. Recent studies have indeed begun highlighting FRA1’s emerging role in immune regulation, illustrating its ability to modulate cytokine expression, inflammatory responses, and immune cell recruitment (Atsaves et al., 2019; He et al., 2022; Karakaslar et al., 2023). For example, FRA1 has been reported to negatively regulate inflammatory responses in macrophages and modulate cytokine production to control immune homeostasis (He et al., 2022). Additionally, FRA1 influences the differentiation and function of T-cells by modulating IL-17 and IL-22 expression, thereby playing a pivotal role in shaping adaptive immune responses (Atsaves et al., 2019). Our previous study further supports this immunomodulatory role, demonstrating that FRA1 overexpression suppressed interferon-related inflammatory gene networks in CGL1 cells (Al-Khayyat et al., 2023). Collectively, these findings position FRA1 as a potentially critical regulator of immune signaling, where its absence facilitates pro-inflammatory gene expression linked to tumor-promoting inflammation. Future research aimed at delineating FRA1-mediated immune regulatory mechanisms could reveal novel therapeutic targets for inflammatory diseases and cancer prevention.

The transcriptional profiles elicited by serum stimulation further underscore FRA1’s role in fine-tuning early mitogenic responses. Serum-induced gene expression programs in FRA1 knockout cells were markedly amplified, encompassing genes associated with proliferation, ECM remodeling, inflammation, and EMT (Supplementary Table S6). In contrast, FRA1 overexpression constrained this mitogenic response, significantly dampening gene induction across these pathways (Supplementary Table S7). The reciprocal and finely balanced control exerted by FRA1 suggests that it actively modulates cellular responsiveness to external growth stimuli, maintaining transcriptional homeostasis and preventing excessive proliferative signaling that could initiate neoplastic transformation (Table 3). These collective findings position FRA1 as a critical transcriptional gatekeeper that fine-tunes basal and stimulus-responsive gene expression, safeguarding cellular homeostasis by tightly repressing the expression of pro-tumorigenic genes. Without FRA1, cells experience unchecked overexpression of oncogenic and pro-inflammatory genes, thereby creating a cellular environment primed for neoplastic transformation. Conversely, FRA1 overexpression reinforces transcriptional restraint, maintaining cellular integrity and resistance to oncogenic transformation.

### 4.5 Limitations and future directions

While this study establishes FRA1 as a key suppressor of neoplastic transformation in the CGL1 model, several limitations and avenues for future research remain. First, although the CGL1 hybrid cell line provides a unique and well-characterized system to quantify transformation frequency, validation of these findings in additional cervical cancer models would further strengthen the generalizability of our results. Second, while our CRISPR-based knockout achieved ∼70% reduction of FRA1 expression, this partial suppression is a recognized limitation of CRISPR/Cas9 editing (Hunt et al., 2023), and future work could employ complementary strategies to achieve more complete gene silencing. Importantly, the reciprocal patterns observed in the knockout and overexpression systems strongly support the robustness of our conclusions despite this partial reduction. Third, although we identified broad transcriptional networks regulated by FRA1, the direct interactors and mechanisms of transcriptional repression remain to be defined. Future work should employ ChIP-seq (chromatin immunoprecipitation sequencing), ATAC-seq (assay for transposase-accessible chromatin using sequencing), or proteomic approaches to map FRA1 binding partners and regulatory elements that control AP-1 complex activity and downstream signaling pathways. Finally, integration of FRA1-dependent transcriptional signatures with large-scale cancer genomics datasets will be valuable for establishing the clinical relevance of FRA1-mediated tumor suppression in cervical cancer and potentially in other malignancies.

## 5 Conclusion

This study demonstrated that FRA1 (*FOSL1*) functioned as a tumor suppressor by limiting neoplastic transformation and modulating radiation responses in the CGL1 hybrid cell model. FRA1 overexpression suppressed both spontaneous and radiation-induced transformation, whereas FRA1 knockout enhanced transformation frequency—effects that occurred independently of classical DNA repair mechanisms but were linked to altered G2/M checkpoint activation, shifts in AP-1 subunit composition, and broad transcriptional reprogramming. Mechanistically, FRA1 repressed gene networks associated with proliferation, extracellular matrix remodeling, inflammation, and hypoxia—processes commonly implicated in early tumor development. These regulatory effects were observed under both baseline and mitogen-stimulated conditions, supporting a model in which FRA1 fine-tuned stress-responsive gene expression to preserve transcriptional and cellular homeostasis. Although cFOS was strongly dysregulated in FRA1-deficient cells, cFOS inhibition did not reverse the transformation phenotype, indicating that FRA1 governed a broader oncogenic regulatory axis.

Future studies should investigate direct genomic targets of FRA1 using chromatin immunoprecipitation followed by sequencing and define its transcriptional interactome within the AP-1 complex. Candidate downstream genes such as *FN1*, *MMP2*, *EPAS1*, *ISG15*, *IL32*, and *ICAM1*—identified in this study as reciprocally regulated by FRA1—represent key nodes in proliferation, immune signaling, and matrix remodeling pathways and merit further functional interrogation. In addition, exploring whether FRA1 modulates tumor-suppressive factors such as *CDKN1A*, *GADD45*, or interferon regulators like *IRF1* may yield mechanistic insights into its role in early stress adaptation. Broadening these analyses across additional cell types and tumorigenic stages could clarify FRA1’s evolving function in cancer biology. Ultimately, pharmacologic strategies aimed at stabilizing FRA1 or mimicking its transcriptional output could provide a novel framework for cancer prevention and radiosensitization therapies.

## Data Availability

The data presented in the study are deposited in the Gene Expression Omnibus (GEO) repository, accession number GSE307991.
