# Supplementary material for: FRA1 (FOSL1) suppresses neoplastic transformation and modulates radiation responses via transcriptional control of mitogenic and stress-responsive networks
Source: Front Cell Dev Biol. 2025 Sep 22;13:1659986. doi: 10.3389/fcell.2025.1659986 (PMC12497744; doi:10.3389/fcell.2025.1659986)
Supplement: Supplementary file 1 [file Table1.docx]

**Supplementary Tables**

**Supplementary Table 1. Differentially expressed genes in CGL1^FRA1KO^ cells relative to CGL1^Cas9^ control cells under baseline conditions.**

Genes were considered differentially expressed if they exhibited a fold change (F.C.) >1.5 or <–1.5 with an FDR-adjusted *p*-value (p-adj) < 0.05. This table includes gene names, fold changes, p-adj values, and transcript abundance (TPM, transcripts per million). A total of 387 DEGs were identified under serum-starved, non-irradiated (baseline) conditions, consisting of 339 upregulated and 48 downregulated genes. Genes are listed in alphabetical order.

| **Gene ID** | **Gene Name** | **F.C.** | **p-adj.** | **CGL1^Cas9^** | **CGL1^FRA1KO^** |
| --- | --- | --- | --- | --- | --- |
| ENSG00000108846.16 | *ABCC3* | 28.8 | 4.24E-07 | 56.4 | 144.3 |
| ENSG00000173210.19 | *ABLIM3* | 18.9 | 1.53E-02 | 68.9 | 105.8 |
| ENSG00000205325.3 | *AC005863.1* | 15.2 | 4.59E-03 | 81.7 | 149.4 |
| ENSG00000107796.13 | *ACTA2* | 11.6 | 4.41E-17 | 118.3 | 335.5 |
| ENSG00000154736.6 | *ADAMTS5* | 10.8 | 4.37E-06 | 162.0 | 345.0 |
| ENSG00000178031.17 | *ADAMTSL1* | 9.1 | 3.88E-15 | 369.1 | 162.5 |
| ENSG00000272734.1 | *ADIRF-AS1* | 8.9 | 3.81E-02 | 41.6 | 67.6 |
| ENSG00000163485.17 | *ADORA1* | 8.9 | 7.90E-03 | 33.3 | 62.9 |
| ENSG00000185567.7 | *AHNAK2* | 8.8 | 3.08E-06 | 1439.4 | 2400.0 |
| ENSG00000226919.4 | *AL365184.1* | 8.7 | 2.02E-05 | 9.3 | 35.3 |
| ENSG00000286231.1 | *AL445423.3* | 8.7 | 5.65E-06 | 36.6 | 2.4 |
| ENSG00000108602.18 | *ALDH3A1* | 8.3 | 9.95E-03 | 17.3 | 40.9 |
| ENSG00000136859.10 | *ANGPTL2* | 8.2 | 3.63E-03 | 51.7 | 97.7 |
| ENSG00000167772.12 | *ANGPTL4* | 7.3 | 2.14E-03 | 20.1 | 56.4 |
| ENSG00000165887.11 | *ANKRD2* | 7.3 | 3.39E-02 | 12.9 | 34.4 |
| ENSG00000264230.9 | *ANXA8L1* | 7.1 | 1.06E-02 | 2.3 | 19.7 |
| ENSG00000138356.14 | *AOX1* | 7.0 | 1.10E-05 | 166.8 | 61.2 |
| ENSG00000128335.14 | *APOL2* | 6.7 | 3.18E-06 | 227.2 | 377.3 |
| ENSG00000221963.6 | *APOL6* | 6.7 | 5.35E-13 | 228.0 | 427.7 |
| ENSG00000165272.16 | *AQP3* | 6.5 | 8.19E-18 | 28.5 | 135.9 |
| ENSG00000165801.10 | *ARHGEF40* | 6.5 | 6.82E-03 | 292.5 | 445.7 |
| ENSG00000116017.11 | *ARID3A* | 6.3 | 4.04E-04 | 66.9 | 145.5 |
| ENSG00000122644.13 | *ARL4A* | 6.2 | 5.27E-06 | 420.5 | 270.3 |
| ENSG00000188042.8 | *ARL4C* | 6.0 | 1.43E-05 | 24.1 | 63.7 |
| ENSG00000125962.14 | *ARMCX5* | 5.3 | 2.85E-02 | 7.2 | 34.4 |
| ENSG00000099889.14 | *ARVCF* | 5.3 | 9.77E-04 | 139.5 | 238.2 |
| ENSG00000162772.17 | *ATF3* | 5.2 | 7.05E-04 | 230.4 | 353.8 |
| ENSG00000159363.18 | *ATP13A2* | 5.2 | 1.22E-05 | 257.4 | 516.7 |
| ENSG00000112182.15 | *BACH2* | 5.1 | 7.05E-03 | 11.6 | 37.5 |
| ENSG00000069399.15 | *BCL3* | 5.0 | 2.01E-04 | 61.7 | 119.6 |
| ENSG00000134107.5 | *BHLHE40* | 4.8 | 3.12E-09 | 110.7 | 252.3 |
| ENSG00000168487.19 | *BMP1* | 4.7 | 4.20E-10 | 431.8 | 697.8 |
| ENSG00000164619.10 | *BMPER* | 4.6 | 3.37E-02 | 21.4 | 4.8 |
| ENSG00000159388.6 | *BTG2* | 4.5 | 1.23E-02 | 91.8 | 145.7 |
| ENSG00000026950.17 | *BTN3A1* | 4.5 | 4.12E-05 | 98.1 | 185.6 |
| ENSG00000119280.16 | *C1orf198* | 4.5 | 1.65E-02 | 13.6 | 34.2 |
| ENSG00000116667.14 | *C1orf21* | 4.5 | 1.70E-02 | 90.1 | 45.0 |
| ENSG00000159403.18 | *C1R* | 4.4 | 3.68E-02 | 21.8 | 46.2 |
| ENSG00000125730.17 | *C3* | 4.4 | 3.57E-05 | 12.1 | 85.0 |
| ENSG00000244731.8 | *C4A* | 4.3 | 1.77E-02 | 3.6 | 32.2 |
| ENSG00000075461.6 | *CACNG4* | 4.3 | 1.78E-02 | 42.1 | 72.2 |
| ENSG00000178033.6 | *CALHM5* | 4.2 | 4.63E-02 | 7.1 | 19.2 |
| ENSG00000168497.5 | *CAVIN2* | 4.2 | 6.47E-03 | 125.5 | 209.2 |
| ENSG00000183287.14 | *CCBE1* | 4.1 | 4.96E-02 | 44.3 | 19.8 |
| ENSG00000108691.9 | *CCL2* | 4.0 | 7.10E-06 | 70.0 | 207.9 |
| ENSG00000271503.6 | *CCL5* | 3.8 | 4.31E-57 | 39.7 | 352.1 |
| ENSG00000118523.6 | *CCN2* | 3.7 | 4.55E-08 | 1446.6 | 2297.0 |
| ENSG00000146574.15 | *CCZ1B* | 3.6 | 2.14E-03 | 52.5 | 11.3 |
| ENSG00000272398.6 | *CD24* | 3.6 | 3.95E-04 | 43.9 | 87.7 |
| ENSG00000196352.15 | *CD55* | 3.5 | 4.99E-02 | 579.4 | 367.3 |
| ENSG00000125726.11 | *CD70* | 3.5 | 1.73E-02 | 20.2 | 41.5 |
| ENSG00000124762.13 | *CDKN1A* | 3.4 | 2.41E-02 | 22.8 | 51.1 |
| ENSG00000147883.12 | *CDKN2B* | 3.4 | 1.17E-16 | 439.2 | 708.9 |
| ENSG00000163347.6 | *CLDN1* | 3.4 | 3.24E-23 | 15.9 | 103.1 |
| ENSG00000169583.13 | *CLIC3* | 3.3 | 1.51E-10 | 210.1 | 371.3 |
| ENSG00000120885.22 | *CLU* | 3.3 | 1.69E-06 | 1643.7 | 2499.1 |
| ENSG00000162368.13 | *CMPK1* | 3.3 | 1.54E-02 | 41.8 | 8.9 |
| ENSG00000140931.20 | *CMTM3* | 3.3 | 4.40E-03 | 106.0 | 172.0 |
| ENSG00000100473.18 | *COCH* | 3.2 | 5.01E-06 | 81.3 | 31.7 |
| ENSG00000111799.21 | *COL12A1* | 3.2 | 1.12E-10 | 13258.5 | 20440.9 |
| ENSG00000187955.12 | *COL14A1* | 3.2 | 2.25E-04 | 7.0 | 31.2 |
| ENSG00000084636.18 | *COL16A1* | 3.2 | 2.12E-06 | 922.9 | 1554.5 |
| ENSG00000187498.16 | *COL4A1* | 3.2 | 2.14E-09 | 1321.8 | 2436.5 |
| ENSG00000130635.15 | *COL5A1* | 3.1 | 2.93E-02 | 11.2 | 27.0 |
| ENSG00000080573.7 | *COL5A3* | 3.0 | 1.19E-02 | 34.8 | 64.0 |
| ENSG00000158270.12 | *COLEC12* | 3.0 | 4.07E-07 | 3.1 | 33.1 |
| ENSG00000128510.12 | *CPA4* | 3.0 | 1.96E-93 | 1851.3 | 4045.4 |
| ENSG00000153002.12 | *CPB1* | 3.0 | 2.03E-04 | 4.2 | 25.7 |
| ENSG00000143320.9 | *CRABP2* | 3.0 | 1.86E-35 | 355.9 | 802.9 |
| ENSG00000005339.14 | *CREBBP* | 3.0 | 5.97E-06 | 381.5 | 638.8 |
| ENSG00000213145.10 | *CRIP1* | 3.0 | 2.91E-05 | 125.2 | 288.6 |
| ENSG00000006016.11 | *CRLF1* | 3.0 | 1.06E-03 | 5.2 | 26.6 |
| ENSG00000109846.9 | *CRYAB* | 2.9 | 2.54E-05 | 11.9 | 41.9 |
| ENSG00000184371.14 | *CSF1* | 2.9 | 6.82E-03 | 205.9 | 327.0 |
| ENSG00000204435.13 | *CSNK2B* | 2.9 | 3.18E-02 | 52.0 | 84.5 |
| ENSG00000159176.14 | *CSRP1* | 2.8 | 3.49E-30 | 1425.7 | 2687.4 |
| ENSG00000018610.14 | *CXorf56* | 2.8 | 2.60E-04 | 1.5 | 22.8 |
| ENSG00000138061.12 | *CYP1B1* | 2.8 | 2.18E-31 | 1256.6 | 1975.6 |
| ENSG00000011465.17 | *DCN* | 2.7 | 3.43E-06 | 594.2 | 983.5 |
| ENSG00000107201.10 | *DDX58* | 2.7 | 1.14E-06 | 151.7 | 366.5 |
| ENSG00000137628.17 | *DDX60* | 2.7 | 3.77E-07 | 247.1 | 534.8 |
| ENSG00000146966.13 | *DENND2A* | 2.7 | 2.01E-02 | 30.7 | 53.8 |
| ENSG00000162496.9 | *DHRS3* | 2.7 | 1.51E-02 | 36.0 | 74.5 |
| ENSG00000108771.13 | *DHX58* | 2.7 | 1.90E-04 | 21.9 | 65.4 |
| ENSG00000198947.15 | *DMD* | 2.7 | 1.39E-03 | 126.4 | 239.2 |
| ENSG00000104936.17 | *DMPK* | 2.7 | 2.03E-02 | 64.3 | 113.2 |
| ENSG00000187957.8 | *DNER* | 2.6 | 7.77E-10 | 10.3 | 51.4 |
| ENSG00000147251.15 | *DOCK11* | 2.6 | 2.16E-03 | 162.7 | 356.2 |
| ENSG00000136048.14 | *DRAM1* | 2.6 | 2.98E-20 | 388.4 | 703.8 |
| ENSG00000163840.10 | *DTX3L* | 2.6 | 2.65E-10 | 276.9 | 477.9 |
| ENSG00000120875.9 | *DUSP4* | 2.6 | 1.74E-40 | 36.2 | 243.4 |
| ENSG00000164176.13 | *EDIL3* | 2.6 | 7.02E-17 | 273.7 | 527.2 |
| ENSG00000078401.7 | *EDN1* | 2.6 | 1.01E-11 | 136.2 | 299.0 |
| ENSG00000127129.10 | *EDN2* | 2.6 | 3.14E-03 | 7.5 | 23.9 |
| ENSG00000115380.20 | *EFEMP1* | 2.6 | 1.46E-68 | 2479.5 | 5525.9 |
| ENSG00000115468.12 | *EFHD1* | 2.5 | 1.40E-02 | 23.6 | 47.2 |
| ENSG00000120738.8 | *EGR1* | 2.5 | 2.08E-10 | 123.9 | 261.6 |
| ENSG00000165521.15 | *EML5* | 2.5 | 1.30E-02 | 70.5 | 36.3 |
| ENSG00000116016.14 | *EPAS1* | 2.5 | 6.25E-10 | 57.1 | 184.2 |
| ENSG00000163161.13 | *ERCC3* | 2.5 | 9.71E-03 | 78.1 | 132.2 |
| ENSG00000006468.14 | *ETV1* | 2.5 | 4.90E-02 | 33.3 | 82.4 |
| ENSG00000175832.13 | *ETV4* | 2.5 | 2.94E-04 | 73.3 | 140.5 |
| ENSG00000244405.8 | *ETV5* | 2.5 | 7.80E-03 | 182.9 | 308.9 |
| ENSG00000121769.8 | *FABP3* | 2.4 | 2.41E-02 | 11.0 | 25.8 |
| ENSG00000234964.4 | *FABP5P7* | 2.4 | 2.48E-03 | 137.5 | 85.1 |
| ENSG00000164970.15 | *FAM219A* | 2.4 | 1.60E-04 | 128.1 | 195.7 |
| ENSG00000185112.6 | *FAM43A* | 2.4 | 3.51E-03 | 72.8 | 115.5 |
| ENSG00000162458.13 | *FBLIM1* | 2.4 | 7.26E-09 | 284.4 | 596.3 |
| ENSG00000138829.12 | *FBN2* | 2.4 | 3.10E-07 | 978.3 | 1584.9 |
| ENSG00000066468.22 | *FGFR2* | 2.4 | 9.03E-06 | 20.2 | 0.2 |
| ENSG00000160867.15 | *FGFR4* | 2.3 | 3.79E-02 | 49.9 | 26.8 |
| ENSG00000136068.14 | *FLNB* | 2.3 | 7.35E-05 | 5518.3 | 8513.4 |
| ENSG00000102755.12 | *FLT1* | 2.3 | 5.40E-07 | 4.2 | 36.2 |
| ENSG00000115414.19 | *FN1* | 2.3 | 9.31E-13 | 4716.3 | 9091.5 |
| ENSG00000053254.15 | *FOXN3* | 2.3 | 2.74E-02 | 238.8 | 403.4 |
| ENSG00000138759.19 | *FRAS1* | 2.3 | 2.33E-05 | 524.1 | 814.5 |
| ENSG00000157240.4 | *FZD1* | 2.3 | 2.61E-08 | 303.0 | 541.6 |
| ENSG00000174804.4 | *FZD4* | 2.3 | 1.32E-15 | 238.5 | 427.7 |
| ENSG00000268089.3 | *GABRQ* | 2.3 | 1.69E-02 | 68.8 | 38.9 |
| ENSG00000117228.10 | *GBP1* | 2.3 | 7.64E-19 | 131.8 | 423.0 |
| ENSG00000117226.12 | *GBP3* | 2.3 | 3.14E-02 | 76.0 | 149.9 |
| ENSG00000130513.6 | *GDF15* | 2.2 | 1.01E-07 | 38.0 | 161.1 |
| ENSG00000151892.14 | *GFRA1* | 2.2 | 3.27E-17 | 1276.4 | 1991.4 |
| ENSG00000115159.16 | *GPD2* | 2.2 | 2.12E-05 | 1036.3 | 675.8 |
| ENSG00000158292.7 | *GPR153* | 2.2 | 1.13E-03 | 33.3 | 66.3 |
| ENSG00000013588.8 | *GPRC5A* | 2.2 | 1.78E-06 | 492.7 | 818.2 |
| ENSG00000167191.12 | *GPRC5B* | 2.2 | 4.59E-03 | 105.5 | 177.9 |
| ENSG00000213366.13 | *GSTM2* | 2.2 | 3.43E-02 | 46.2 | 72.6 |
| ENSG00000168765.17 | *GSTM4* | 2.2 | 7.15E-24 | 483.3 | 798.6 |
| ENSG00000113070.8 | *HBEGF* | 2.2 | 4.80E-03 | 107.3 | 180.9 |
| ENSG00000099822.3 | *HCN2* | 2.2 | 1.39E-03 | 87.8 | 144.0 |
| ENSG00000173706.14 | *HEG1* | 2.2 | 1.63E-37 | 1329.7 | 2531.6 |
| ENSG00000138646.9 | *HERC5* | 2.2 | 8.05E-17 | 103.0 | 303.2 |
| ENSG00000114315.4 | *HES1* | 2.2 | 6.88E-03 | 29.5 | 62.0 |
| ENSG00000054392.13 | *HHAT* | 2.2 | 1.23E-03 | 44.5 | 93.0 |
| ENSG00000164161.10 | *HHIP* | 2.2 | 2.03E-04 | 230.6 | 105.8 |
| ENSG00000127124.16 | *HIVEP3* | 2.2 | 4.66E-03 | 32.3 | 79.6 |
| ENSG00000234745.11 | *HLA-B* | 2.2 | 9.09E-69 | 3532.0 | 6737.3 |
| ENSG00000204642.14 | *HLA-F* | 2.2 | 2.72E-05 | 26.3 | 68.3 |
| ENSG00000206341.7 | *HLA-H* | 2.2 | 3.83E-05 | 99.7 | 165.8 |
| ENSG00000136630.13 | *HLX* | 2.1 | 2.83E-02 | 120.9 | 183.2 |
| ENSG00000072571.20 | *HMMR* | 2.1 | 4.28E-03 | 1573.8 | 1029.8 |
| ENSG00000158104.11 | *HPD* | 2.1 | 8.98E-03 | 35.0 | 64.5 |
| ENSG00000173083.15 | *HPSE* | 2.1 | 3.14E-02 | 111.3 | 175.2 |
| ENSG00000086696.11 | *HSD17B2* | 2.1 | 2.05E-04 | 19.2 | 58.4 |
| ENSG00000113013.15 | *HSPA9* | 2.1 | 1.35E-04 | 559.2 | 279.6 |
| ENSG00000142798.20 | *HSPG2* | 2.1 | 1.81E-06 | 1668.6 | 2740.3 |
| ENSG00000090339.9 | *ICAM1* | 2.1 | 2.08E-19 | 151.3 | 333.8 |
| ENSG00000115738.10 | *ID2* | 2.1 | 2.42E-02 | 167.4 | 108.9 |
| ENSG00000137331.12 | *IER3* | 2.1 | 4.17E-12 | 312.7 | 591.3 |
| ENSG00000165949.12 | *IFI27* | 2.1 | 3.70E-05 | 3.8 | 43.6 |
| ENSG00000216490.4 | *IFI30* | 2.1 | 1.33E-09 | 253.0 | 414.2 |
| ENSG00000137965.11 | *IFI44* | 2.1 | 3.10E-16 | 415.8 | 724.6 |
| ENSG00000137959.16 | *IFI44L* | 2.1 | 2.60E-03 | 97.3 | 192.0 |
| ENSG00000126709.15 | *IFI6* | 2.1 | 1.99E-18 | 109.4 | 273.4 |
| ENSG00000115267.8 | *IFIH1* | 2.1 | 3.57E-10 | 54.4 | 134.2 |
| ENSG00000185745.10 | *IFIT1* | 2.1 | 4.67E-29 | 568.7 | 1025.2 |
| ENSG00000119917.14 | *IFIT3* | 2.1 | 4.89E-46 | 768.6 | 1710.0 |
| ENSG00000141753.7 | *IGFBP4* | 2.1 | 1.88E-30 | 51.8 | 226.2 |
| ENSG00000115461.5 | *IGFBP5* | 2.1 | 7.68E-31 | 24.7 | 181.8 |
| ENSG00000167779.9 | *IGFBP6* | 2.1 | 1.92E-25 | 204.6 | 545.6 |
| ENSG00000163453.11 | *IGFBP7* | 2.1 | 7.56E-13 | 548.0 | 905.2 |
| ENSG00000268621.6 | *IGFL2-AS1* | 2.1 | 2.31E-02 | 55.1 | 86.7 |
| ENSG00000144730.18 | *IL17RD* | 2.1 | 3.75E-02 | 77.7 | 139.7 |
| ENSG00000196083.10 | *IL1RAP* | 2.0 | 1.81E-04 | 118.5 | 190.7 |
| ENSG00000008517.16 | *IL32* | 2.0 | 7.20E-18 | 96.1 | 255.4 |
| ENSG00000136244.12 | *IL6* | 2.0 | 3.64E-10 | 5.1 | 41.6 |
| ENSG00000122641.11 | *INHBA* | 2.0 | 2.57E-05 | 2.6 | 21.7 |
| ENSG00000125347.14 | *IRF1* | 2.0 | 3.26E-08 | 145.0 | 298.5 |
| ENSG00000185507.21 | *IRF7* | 2.0 | 5.31E-03 | 21.1 | 52.1 |
| ENSG00000187608.10 | *ISG15* | 2.0 | 1.53E-26 | 857.0 | 1777.0 |
| ENSG00000172183.15 | *ISG20* | 2.0 | 2.17E-04 | 11.5 | 38.1 |
| ENSG00000164171.11 | *ITGA2* | 2.0 | 7.15E-03 | 84.7 | 132.7 |
| ENSG00000161638.11 | *ITGA5* | 2.0 | 7.41E-16 | 1064.4 | 1774.4 |
| ENSG00000082781.12 | *ITGB5* | 2.0 | 4.45E-08 | 623.0 | 1232.1 |
| ENSG00000171223.6 | *JUNB* | 2.0 | 6.09E-17 | 224.2 | 433.5 |
| ENSG00000173801.17 | *JUP* | 2.0 | 1.66E-15 | 269.6 | 534.3 |
| ENSG00000120071.14 | *KANSL1* | 1.9 | 8.31E-04 | 249.8 | 382.0 |
| ENSG00000121361.5 | *KCNJ8* | 1.9 | 5.40E-04 | 44.8 | 15.7 |
| ENSG00000104783.14 | *KCNN4* | 1.9 | 1.65E-02 | 42.1 | 73.1 |
| ENSG00000134504.14 | *KCTD1* | 1.9 | 8.86E-05 | 128.2 | 220.7 |
| ENSG00000120549.18 | *KIAA1217* | 1.9 | 6.16E-04 | 81.4 | 140.1 |
| ENSG00000169926.11 | *KLF13* | 1.9 | 1.13E-12 | 330.7 | 596.8 |
| ENSG00000136826.15 | *KLF4* | 1.9 | 2.66E-12 | 185.3 | 431.3 |
| ENSG00000128422.17 | *KRT17* | 1.9 | 8.20E-79 | 273.0 | 1712.3 |
| ENSG00000170477.13 | *KRT4* | 1.9 | 1.72E-04 | 133.6 | 254.3 |
| ENSG00000167767.14 | *KRT80* | 1.9 | 3.95E-10 | 2167.2 | 3411.0 |
| ENSG00000198910.14 | *L1CAM* | 1.9 | 1.20E-21 | 800.0 | 1471.1 |
| ENSG00000112769.20 | *LAMA4* | 1.9 | 1.27E-18 | 787.2 | 1365.9 |
| ENSG00000130702.15 | *LAMA5* | 1.9 | 3.72E-08 | 761.4 | 1475.0 |
| ENSG00000196878.15 | *LAMB3* | 1.9 | 3.49E-08 | 17.3 | 76.1 |
| ENSG00000213626.13 | *LBH* | 1.9 | 7.33E-11 | 89.4 | 199.0 |
| ENSG00000106003.13 | *LFNG* | 1.9 | 3.58E-06 | 73.2 | 164.8 |
| ENSG00000143355.16 | *LHX9* | 1.9 | 3.46E-07 | 35.2 | 104.3 |
| ENSG00000128342.5 | *LIF* | 1.9 | 4.54E-05 | 113.4 | 238.3 |
| ENSG00000072163.19 | *LIMS2* | 1.9 | 1.04E-02 | 17.3 | 71.0 |
| ENSG00000233237.8 | *LINC00472* | 1.9 | 3.05E-02 | 90.7 | 144.3 |
| ENSG00000238266.2 | *LINC00707* | 1.9 | 2.90E-02 | 65.3 | 34.4 |
| ENSG00000071282.12 | *LMCD1* | 1.9 | 1.21E-03 | 33.0 | 81.1 |
| ENSG00000129038.16 | *LOXL1* | 1.9 | 7.72E-12 | 538.9 | 910.6 |
| ENSG00000138131.4 | *LOXL4* | 1.9 | 5.04E-04 | 58.1 | 107.1 |
| ENSG00000123384.14 | *LRP1* | 1.9 | 1.01E-06 | 1871.3 | 2957.2 |
| ENSG00000119681.12 | *LTBP2* | 1.8 | 1.98E-09 | 121.4 | 238.7 |
| ENSG00000147676.14 | *MAL2* | 1.8 | 1.81E-03 | 88.7 | 165.8 |
| ENSG00000069535.14 | *MAOB* | 1.8 | 5.55E-03 | 25.1 | 51.1 |
| ENSG00000078018.19 | *MAP2* | 1.8 | 1.41E-03 | 38.6 | 84.5 |
| ENSG00000180611.7 | *MB21D2* | 1.8 | 9.45E-06 | 245.4 | 385.8 |
| ENSG00000229619.4 | *MBNL1-AS1* | 1.8 | 1.18E-03 | 87.2 | 138.9 |
| ENSG00000076706.17 | *MCAM* | 1.8 | 1.38E-14 | 451.2 | 776.2 |
| ENSG00000068305.17 | *MEF2A* | 1.8 | 4.15E-03 | 318.3 | 510.3 |
| ENSG00000162591.16 | *MEGF6* | 1.8 | 1.68E-03 | 124.0 | 194.6 |
| ENSG00000105419.18 | *MEIS3* | 1.8 | 5.72E-04 | 50.7 | 95.8 |
| ENSG00000181588.16 | *MEX3D* | 1.8 | 8.50E-05 | 95.3 | 173.2 |
| ENSG00000117122.14 | *MFAP2* | 1.8 | 4.90E-12 | 26.2 | 109.6 |
| ENSG00000135596.18 | *MICAL1* | 1.8 | 6.32E-03 | 115.4 | 189.5 |
| ENSG00000099812.9 | *MISP* | 1.8 | 8.49E-05 | 36.5 | 85.6 |
| ENSG00000187098.15 | *MITF* | 1.8 | 4.00E-03 | 40.6 | 6.1 |
| ENSG00000087245.13 | *MMP2* | 1.8 | 2.61E-20 | 162.8 | 415.0 |
| ENSG00000125966.10 | *MMP24* | 1.8 | 1.46E-19 | 117.0 | 393.3 |
| ENSG00000169184.6 | *MN1* | 1.8 | 3.95E-03 | 22.3 | 51.3 |
| ENSG00000008382.15 | *MPND* | 1.8 | 4.11E-02 | 15.2 | 32.0 |
| ENSG00000196588.17 | *MRTFA* | 1.8 | 2.39E-03 | 130.5 | 225.8 |
| ENSG00000110921.14 | *MVK* | 1.8 | 7.21E-05 | 153.6 | 76.7 |
| ENSG00000059728.11 | *MXD1* | 1.8 | 2.46E-03 | 63.4 | 132.9 |
| ENSG00000101825.8 | *MXRA5* | 1.8 | 7.33E-22 | 134.3 | 483.2 |
| ENSG00000136286.16 | *MYO1G* | 1.8 | 5.82E-06 | 3.9 | 28.6 |
| ENSG00000136274.9 | *NACAD* | 1.8 | 6.01E-03 | 14.4 | 38.4 |
| ENSG00000143217.9 | *NECTIN4* | 1.8 | 2.22E-05 | 2.3 | 20.6 |
| ENSG00000111859.17 | *NEDD9* | 1.7 | 9.36E-19 | 297.9 | 660.8 |
| ENSG00000050344.9 | *NFE2L3* | 1.7 | 3.35E-15 | 62.2 | 188.3 |
| ENSG00000077150.20 | *NFKB2* | 1.7 | 4.58E-03 | 339.4 | 571.7 |
| ENSG00000144802.11 | *NFKBIZ* | 1.7 | 3.11E-03 | 109.8 | 181.0 |
| ENSG00000109255.11 | *NMU* | 1.7 | 1.63E-03 | 141.1 | 86.3 |
| ENSG00000166741.7 | *NNMT* | 1.7 | 7.22E-07 | 247.0 | 388.2 |
| ENSG00000113389.16 | *NPR3* | 1.7 | 1.66E-06 | 199.8 | 334.7 |
| ENSG00000175745.14 | *NR2F1* | 1.7 | 3.58E-04 | 78.3 | 146.2 |
| ENSG00000074527.12 | *NTN4* | 1.7 | 1.08E-06 | 328.2 | 558.1 |
| ENSG00000111331.13 | *OAS3* | 1.7 | 1.27E-02 | 372.2 | 585.5 |
| ENSG00000185585.20 | *OLFML2A* | 1.7 | 5.00E-12 | 81.9 | 193.1 |
| ENSG00000173391.9 | *OLR1* | 1.7 | 2.47E-21 | 445.7 | 813.1 |
| ENSG00000090530.10 | *P3H2* | 1.7 | 5.78E-08 | 390.7 | 665.4 |
| ENSG00000072682.18 | *P4HA2* | 1.7 | 4.19E-09 | 365.8 | 583.3 |
| ENSG00000099864.18 | *PALM* | 1.7 | 1.41E-11 | 152.9 | 347.0 |
| ENSG00000115421.13 | *PAPOLG* | 1.7 | 1.48E-02 | 169.5 | 278.8 |
| ENSG00000182752.10 | *PAPPA* | 1.7 | 3.62E-20 | 15.7 | 102.0 |
| ENSG00000178685.14 | *PARP10* | 1.7 | 7.06E-07 | 110.5 | 238.3 |
| ENSG00000173193.15 | *PARP14* | 1.7 | 9.47E-06 | 376.0 | 775.8 |
| ENSG00000138496.16 | *PARP9* | 1.7 | 2.29E-04 | 50.1 | 179.5 |
| ENSG00000169851.15 | *PCDH7* | 1.7 | 7.66E-13 | 62.7 | 214.8 |
| ENSG00000197646.8 | *PDCD1LG2* | 1.7 | 1.77E-04 | 12.1 | 37.3 |
| ENSG00000100311.17 | *PDGFB* | 1.7 | 8.79E-05 | 74.6 | 194.1 |
| ENSG00000163110.15 | *PDLIM5* | 1.7 | 1.17E-06 | 779.3 | 1249.4 |
| ENSG00000164951.16 | *PDP1* | 1.7 | 3.55E-08 | 207.2 | 328.2 |
| ENSG00000133401.16 | *PDZD2* | 1.7 | 3.65E-02 | 240.2 | 382.7 |
| ENSG00000179094.16 | *PER1* | 1.7 | 8.69E-03 | 69.2 | 134.3 |
| ENSG00000144824.21 | *PHLDB2* | 1.7 | 5.38E-37 | 620.1 | 1281.1 |
| ENSG00000164530.15 | *PI16* | 1.7 | 7.43E-11 | 31.7 | 142.7 |
| ENSG00000241878.11 | *PISD* | 1.7 | 5.90E-08 | 89.7 | 185.1 |
| ENSG00000105499.14 | *PLA2G4C* | 1.7 | 4.46E-06 | 28.9 | 86.9 |
| ENSG00000133321.11 | *PLAAT4* | 1.7 | 9.81E-25 | 66.2 | 227.9 |
| ENSG00000122861.16 | *PLAU* | 1.7 | 4.93E-13 | 224.1 | 481.0 |
| ENSG00000011422.12 | *PLAUR* | 1.7 | 1.97E-04 | 152.8 | 235.8 |
| ENSG00000105559.12 | *PLEKHA4* | 1.7 | 4.54E-05 | 40.6 | 133.6 |
| ENSG00000225190.11 | *PLEKHM1* | 1.7 | 5.32E-03 | 140.0 | 225.1 |
| ENSG00000145632.15 | *PLK2* | 1.7 | 1.86E-16 | 2787.3 | 4365.8 |
| ENSG00000173846.13 | *PLK3* | 1.7 | 4.45E-03 | 77.6 | 123.8 |
| ENSG00000076356.7 | *PLXNA2* | 1.7 | 1.45E-10 | 143.3 | 303.3 |
| ENSG00000128567.17 | *PODXL* | 1.7 | 6.19E-22 | 1983.2 | 3329.2 |
| ENSG00000170734.11 | *POLH* | 1.6 | 2.69E-03 | 157.5 | 280.7 |
| ENSG00000133110.15 | *POSTN* | 1.6 | 2.23E-17 | 3941.0 | 7058.0 |
| ENSG00000110841.14 | *PPFIBP1* | 1.6 | 1.09E-08 | 421.0 | 707.3 |
| ENSG00000131238.17 | *PPT1* | 1.6 | 7.12E-06 | 2796.2 | 1562.5 |
| ENSG00000275342.5 | *PRAG1* | 1.6 | 9.46E-08 | 87.5 | 191.0 |
| ENSG00000057657.17 | *PRDM1* | 1.6 | 1.02E-03 | 20.0 | 54.1 |
| ENSG00000163637.13 | *PRICKLE2* | 1.6 | 8.43E-03 | 57.5 | 93.8 |
| ENSG00000148426.13 | *PROSER2* | 1.6 | 1.23E-06 | 180.5 | 293.1 |
| ENSG00000176532.4 | *PRR15* | 1.6 | 4.08E-09 | 10.5 | 55.6 |
| ENSG00000150687.12 | *PRSS23* | 1.6 | 2.63E-13 | 1317.0 | 2028.7 |
| ENSG00000240065.8 | *PSMB9* | 1.6 | 9.71E-10 | 64.5 | 153.0 |
| ENSG00000171522.6 | *PTGER4* | 1.6 | 2.83E-04 | 47.9 | 96.2 |
| ENSG00000095303.17 | *PTGS1* | 1.6 | 3.79E-02 | 34.6 | 17.1 |
| ENSG00000163661.4 | *PTX3* | 1.6 | 3.83E-02 | 26.0 | 47.7 |
| ENSG00000168994.13 | *PXDC1* | 1.6 | 4.74E-03 | 191.3 | 65.6 |
| ENSG00000132329.11 | *RAMP1* | 1.6 | 3.60E-03 | 68.9 | 33.5 |
| ENSG00000106538.10 | *RARRES2* | 1.6 | 1.70E-03 | 76.2 | 164.7 |
| ENSG00000172575.12 | *RASGRP1* | 1.6 | 7.55E-06 | 10.8 | 64.8 |
| ENSG00000112183.15 | *RBM24* | 1.6 | 1.26E-02 | 131.2 | 81.9 |
| ENSG00000104856.14 | *RELB* | 1.6 | 5.89E-04 | 72.9 | 136.6 |
| ENSG00000167550.11 | *RHEBL1* | 1.6 | 2.83E-02 | 39.5 | 67.1 |
| ENSG00000145860.11 | *RNF145* | 1.6 | 2.18E-03 | 338.0 | 153.7 |
| ENSG00000101236.17 | *RNF24* | 1.6 | 3.87E-04 | 266.2 | 474.7 |
| ENSG00000154133.15 | *ROBO4* | 1.6 | 1.24E-03 | 7.2 | 31.0 |
| ENSG00000198208.11 | *RPS6KL1* | 1.6 | 2.59E-02 | 70.1 | 109.9 |
| ENSG00000166592.12 | *RRAD* | 1.6 | 1.26E-02 | 7.0 | 21.0 |
| ENSG00000126458.4 | *RRAS* | 1.6 | 9.79E-06 | 154.7 | 245.2 |
| ENSG00000134321.12 | *RSAD2* | 1.6 | 2.20E-30 | 3.8 | 109.5 |
| ENSG00000196154.12 | *S100A4* | 1.6 | 4.92E-10 | 556.9 | 1083.9 |
| ENSG00000173432.12 | *SAA1* | 1.6 | 1.04E-04 | 6.6 | 30.4 |
| ENSG00000101115.13 | *SALL4* | 1.6 | 3.39E-02 | 8.9 | 23.8 |
| ENSG00000177409.12 | *SAMD9L* | 1.6 | 1.57E-02 | 11.2 | 58.1 |
| ENSG00000130066.16 | *SAT1* | 1.6 | 2.14E-02 | 39.6 | 69.0 |
| ENSG00000091490.11 | *SEL1L3* | 1.6 | 1.09E-04 | 209.7 | 327.0 |
| ENSG00000075213.11 | *SEMA3A* | 1.6 | 8.41E-03 | 107.4 | 197.2 |
| ENSG00000001617.12 | *SEMA3F* | 1.6 | 4.34E-02 | 94.1 | 152.4 |
| ENSG00000185033.14 | *SEMA4B* | 1.6 | 7.62E-03 | 3.4 | 30.9 |
| ENSG00000112902.12 | *SEMA5A* | 1.6 | 5.40E-04 | 102.1 | 216.7 |
| ENSG00000082497.12 | *SERTAD4* | 1.6 | 1.67E-19 | 268.6 | 588.5 |
| ENSG00000168137.16 | *SETD5* | 1.6 | 7.70E-03 | 615.6 | 1004.1 |
| ENSG00000145423.5 | *SFRP2* | 1.6 | 1.39E-02 | 4.6 | 17.1 |
| ENSG00000125089.17 | *SH3TC1* | 1.6 | 4.98E-02 | 4.5 | 20.0 |
| ENSG00000113504.21 | *SLC12A7* | 1.6 | 3.53E-02 | 105.2 | 165.0 |
| ENSG00000221955.10 | *SLC12A8* | 1.6 | 1.00E-03 | 22.0 | 76.9 |
| ENSG00000163393.13 | *SLC22A15* | 1.6 | 2.27E-02 | 7.0 | 23.6 |
| ENSG00000151229.13 | *SLC2A13* | 1.6 | 9.14E-05 | 149.6 | 256.6 |
| ENSG00000059804.16 | *SLC2A3* | 1.6 | 1.07E-02 | 41.4 | 82.8 |
| ENSG00000022567.9 | *SLC45A4* | 1.6 | 4.13E-03 | 22.0 | 88.6 |
| ENSG00000176463.14 | *SLCO3A1* | 1.6 | 2.99E-02 | 64.4 | 115.6 |
| ENSG00000154760.14 | *SLFN13* | 1.6 | 4.77E-04 | 41.6 | 131.4 |
| ENSG00000166002.7 | *SMCO4* | 1.6 | 4.01E-02 | 104.2 | 61.1 |
| ENSG00000143842.15 | *SOX13* | 1.6 | 1.04E-03 | 191.0 | 304.2 |
| ENSG00000125398.8 | *SOX9* | 1.6 | 1.82E-03 | 18.1 | 46.7 |
| ENSG00000170469.10 | *SPATA24* | 1.6 | 1.73E-02 | 47.5 | 21.5 |
| ENSG00000176170.13 | *SPHK1* | 1.6 | 5.13E-03 | 228.8 | 121.2 |
| ENSG00000164056.11 | *SPRY1* | 1.5 | 2.12E-04 | 14.4 | 47.6 |
| ENSG00000136158.12 | *SPRY2* | 1.5 | 9.64E-04 | 32.0 | 66.6 |
| ENSG00000173898.13 | *SPTBN2* | 1.5 | 2.81E-02 | 394.1 | 217.9 |
| ENSG00000137877.10 | *SPTBN5* | 1.5 | 1.28E-03 | 15.9 | 42.7 |
| ENSG00000179954.16 | *SSC5D* | 1.5 | 5.56E-05 | 6.7 | 35.9 |
| ENSG00000115525.18 | *ST3GAL5* | 1.5 | 4.23E-04 | 30.2 | 67.8 |
| ENSG00000197457.10 | *STMN3* | 1.5 | 5.29E-04 | 18.3 | 53.5 |
| ENSG00000001460.18 | *STPG1* | 1.5 | 1.36E-02 | 85.1 | 46.8 |
| ENSG00000137868.19 | *STRA6* | 1.5 | 3.40E-39 | 57.6 | 388.2 |
| ENSG00000197321.14 | *SVIL* | 1.5 | 8.08E-09 | 203.9 | 396.8 |
| ENSG00000173227.14 | *SYT12* | 1.5 | 1.75E-06 | 52.7 | 110.6 |
| ENSG00000139192.12 | *TAPBPL* | 1.5 | 2.91E-02 | 18.0 | 38.6 |
| ENSG00000165929.13 | *TC2N* | 1.5 | 4.55E-02 | 21.8 | 7.2 |
| ENSG00000196116.8 | *TDRD7* | 1.5 | 1.76E-05 | 88.0 | 169.8 |
| ENSG00000092969.12 | *TGFB2* | -1.5 | 4.65E-28 | 293.7 | 716.7 |
| ENSG00000120708.17 | *TGFBI* | -1.5 | 2.53E-12 | 7039.7 | 12988.7 |
| ENSG00000106799.13 | *TGFBR1* | -1.5 | 5.30E-04 | 269.0 | 427.5 |
| ENSG00000092295.12 | *TGM1* | -1.6 | 1.26E-04 | 41.5 | 109.7 |
| ENSG00000198959.12 | *TGM2* | -1.6 | 3.03E-04 | 12.5 | 56.7 |
| ENSG00000137801.11 | *THBS1* | -1.6 | 3.28E-28 | 6676.9 | 17609.8 |
| ENSG00000169231.13 | *THBS3* | -1.6 | 9.81E-03 | 64.5 | 111.5 |
| ENSG00000156299.13 | *TIAM1* | -1.6 | 3.34E-02 | 82.0 | 29.3 |
| ENSG00000006118.14 | *TMEM132A* | -1.6 | 1.38E-04 | 47.8 | 103.3 |
| ENSG00000164180.13 | *TMEM161B* | -1.7 | 2.00E-02 | 172.5 | 96.1 |
| ENSG00000184584.13 | *TMEM173* | -1.8 | 3.33E-16 | 377.1 | 614.9 |
| ENSG00000172738.12 | *TMEM217* | -1.8 | 1.23E-02 | 7.8 | 23.1 |
| ENSG00000154646.9 | *TMPRSS15* | -1.8 | 5.32E-04 | 50.0 | 13.3 |
| ENSG00000164761.9 | *TNFRSF11B* | -1.8 | 4.89E-02 | 17.9 | 35.0 |
| ENSG00000125657.5 | *TNFSF9* | -1.8 | 3.79E-02 | 70.9 | 112.0 |
| ENSG00000114854.8 | *TNNC1* | -1.9 | 6.58E-03 | 14.3 | 36.7 |
| ENSG00000118194.20 | *TNNT2* | -1.9 | 3.92E-03 | 36.7 | 70.3 |
| ENSG00000100354.21 | *TNRC6B* | -1.9 | 1.87E-03 | 295.1 | 453.9 |
| ENSG00000131746.13 | *TNS4* | -1.9 | 2.75E-06 | 1.2 | 22.9 |
| ENSG00000168477.19 | *TNXB* | -2.0 | 4.39E-02 | 57.2 | 89.8 |
| ENSG00000111907.21 | *TPD52L1* | -2.0 | 4.92E-04 | 27.2 | 64.6 |
| ENSG00000056558.11 | *TRAF1* | -2.0 | 1.20E-02 | 12.0 | 34.4 |
| ENSG00000137699.17 | *TRIM29* | -2.0 | 2.16E-19 | 26.1 | 136.7 |
| ENSG00000132481.7 | *TRIM47* | -2.1 | 4.23E-03 | 86.8 | 136.7 |
| ENSG00000182179.13 | *UBA7* | -2.2 | 1.42E-05 | 35.0 | 78.8 |
| ENSG00000156587.16 | *UBE2L6* | -2.2 | 6.60E-05 | 77.2 | 136.9 |
| ENSG00000158062.20 | *UBXN11* | -2.2 | 2.46E-03 | 224.4 | 343.4 |
| ENSG00000107731.12 | *UNC5B* | -2.2 | 9.51E-04 | 14.7 | 38.7 |
| ENSG00000243566.6 | *UPK3B* | -2.3 | 2.68E-06 | 8.7 | 37.2 |
| ENSG00000184979.10 | *USP18* | -2.6 | 5.80E-04 | 39.7 | 82.8 |
| ENSG00000148429.14 | *USP6NL* | -2.7 | 1.95E-02 | 83.3 | 27.6 |
| ENSG00000168140.5 | *VASN* | -2.8 | 1.42E-13 | 688.9 | 1159.9 |
| ENSG00000038427.16 | *VCAN* | -2.8 | 2.50E-05 | 31.2 | 220.2 |
| ENSG00000206538.9 | *VGLL3* | -2.9 | 4.02E-04 | 365.0 | 564.4 |
| ENSG00000114251.14 | *WNT5A* | -2.9 | 6.74E-09 | 257.7 | 553.0 |
| ENSG00000143816.8 | *WNT9A* | -3.0 | 4.45E-02 | 32.3 | 55.1 |
| ENSG00000180667.10 | *YOD1* | -3.0 | 1.68E-08 | 61.4 | 0.0 |
| ENSG00000105939.13 | *ZC3HAV1* | -3.6 | 1.28E-17 | 938.7 | 1672.3 |
| ENSG00000197961.11 | *ZNF121* | -3.6 | 4.05E-02 | 87.7 | 31.1 |
| ENSG00000147118.11 | *ZNF182* | -3.8 | 3.28E-02 | 49.8 | 9.5 |
| ENSG00000167840.13 | *ZNF232* | -4.5 | 1.86E-02 | 38.7 | 10.9 |
| ENSG00000249471.8 | *ZNF324B* | -4.6 | 6.50E-03 | 23.6 | 51.0 |
| ENSG00000144331.20 | *ZNF385B* | -4.7 | 7.51E-04 | 143.2 | 88.6 |
| ENSG00000196705.8 | *ZNF431* | -5.2 | 1.19E-02 | 76.2 | 174.4 |
| ENSG00000188033.10 | *ZNF490* | -6.7 | 4.22E-02 | 16.7 | 46.9 |
| ENSG00000198795.11 | *ZNF521* | -15.2 | 7.62E-03 | 65.9 | 112.6 |
| ENSG00000177873.14 | *ZNF619* | -110.9 | 4.28E-02 | 39.4 | 63.4 |
| ENSG00000251192.7 | *ZNF674* | -5980057.7 | 8.15E-03 | 35.4 | 10.0 |

**Supplementary Table 2. Differentially expressed genes in CGL1^FRA1Act^ cells relative to CGL1^dCas9^ control cells under baseline conditions.**

Genes were considered differentially expressed if they exhibited a fold change (F.C.) >1.5 or <–1.5 with an FDR-adjusted *p*-value (p-adj) < 0.05. This table includes gene names, fold changes, p-adj values, and transcript abundance (TPM, transcripts per million). A total of 216 DEGs were identified under serum-starved, non-irradiated (baseline) conditions, consisting of 6 upregulated and 210 downregulated genes. Genes are listed in alphabetical order.

| **Gene ID** | **Gene Name** | **F.C.** | **p-adj.** | **Control** | **Comparison** |
| --- | --- | --- | --- | --- | --- |
| ENSG00000280347.1 | *AC000123.3* | 2.0 | 2.02E-02 | 22.2 | 45.6 |
| ENSG00000196696.12 | *AC009022.1* | 1.7 | 3.03E-02 | 42.9 | 71.4 |
| ENSG00000230606.11 | *AC092683.1* | 2.4 | 4.38E-04 | 58.2 | 138.5 |
| ENSG00000285943.1 | *AC112128.1* | 1.7 | 4.21E-02 | 37.9 | 66.0 |
| ENSG00000280287.1 | *AC131212.3* | 2.0 | 3.32E-02 | 21.1 | 42.1 |
| ENSG00000277701.5 | *AC159540.2* | 2.2 | 2.41E-02 | 71.7 | 155.6 |
| ENSG00000144476.6 | *ACKR3* | -2.8 | 2.19E-04 | 73.0 | 26.4 |
| ENSG00000184227.8 | *ACOT1* | -1.7 | 1.49E-02 | 115.5 | 68.2 |
| ENSG00000107796.13 | *ACTA2* | -1.8 | 1.26E-02 | 143.0 | 79.4 |
| ENSG00000159251.8 | *ACTC1* | 1.7 | 2.07E-02 | 45.6 | 76.0 |
| ENSG00000092847.12 | *AGO1* | 1.6 | 4.00E-02 | 294.5 | 475.6 |
| ENSG00000116127.18 | *ALMS1* | 1.8 | 8.76E-03 | 278.5 | 507.5 |
| ENSG00000187689.10 | *AMTN* | -2.9 | 7.80E-03 | 136.9 | 47.6 |
| ENSG00000101745.17 | *ANKRD12* | 2.3 | 2.23E-03 | 122.0 | 283.1 |
| ENSG00000174501.14 | *ANKRD36C* | 1.7 | 1.68E-02 | 42.8 | 74.3 |
| ENSG00000065413.20 | *ANKRD44* | 1.9 | 4.85E-02 | 44.7 | 83.9 |
| ENSG00000105643.10 | *ARRDC2* | -1.6 | 1.18E-02 | 248.6 | 156.1 |
| ENSG00000066279.18 | *ASPM* | 2.1 | 1.47E-02 | 2210.7 | 4659.7 |
| ENSG00000085224.22 | *ATRX* | 2.1 | 7.41E-03 | 844.2 | 1775.4 |
| ENSG00000123636.18 | *BAZ2B* | 2.3 | 2.95E-02 | 109.4 | 252.8 |
| ENSG00000145734.19 | *BDP1* | 1.7 | 7.15E-03 | 637.5 | 1100.3 |
| ENSG00000106605.11 | *BLVRA* | -1.8 | 9.75E-07 | 318.2 | 177.0 |
| ENSG00000038219.13 | *BOD1L1* | 1.8 | 2.22E-02 | 369.9 | 676.0 |
| ENSG00000165288.11 | *BRWD3* | 1.6 | 2.07E-03 | 308.0 | 485.5 |
| ENSG00000109743.11 | *BST1* | -1.5 | 2.76E-02 | 140.1 | 91.1 |
| ENSG00000169814.14 | *BTD* | -1.9 | 3.20E-03 | 121.2 | 65.4 |
| ENSG00000122483.17 | *CCDC18* | 2.4 | 3.66E-02 | 71.1 | 170.2 |
| ENSG00000175602.4 | *CCDC85B* | -1.6 | 1.02E-02 | 605.5 | 388.5 |
| ENSG00000115355.17 | *CCDC88A* | 1.7 | 3.20E-03 | 656.7 | 1132.7 |
| ENSG00000271503.6 | *CCL5* | -9.3 | 8.67E-10 | 62.0 | 6.7 |
| ENSG00000272398.6 | *CD24* | -2.1 | 4.55E-03 | 53.5 | 25.6 |
| ENSG00000101224.17 | *CDC25B* | 1.6 | 4.59E-02 | 180.9 | 282.5 |
| ENSG00000070831.17 | *CDC42* | -4.8 | 1.64E-04 | 130.6 | 27.3 |
| ENSG00000221869.5 | *CEBPD* | -1.7 | 8.82E-03 | 119.7 | 71.7 |
| ENSG00000138778.12 | *CENPE* | 1.8 | 5.37E-03 | 162.3 | 299.5 |
| ENSG00000117724.13 | *CENPF* | 2.6 | 1.59E-03 | 2741.1 | 7130.3 |
| ENSG00000103995.14 | *CEP152* | 2.2 | 8.50E-04 | 253.3 | 548.6 |
| ENSG00000166004.15 | *CEP295* | 1.7 | 4.04E-02 | 316.4 | 546.6 |
| ENSG00000135837.16 | *CEP350* | 1.7 | 9.96E-03 | 402.0 | 687.4 |
| ENSG00000171316.12 | *CHD7* | 3.1 | 2.39E-02 | 78.7 | 241.6 |
| ENSG00000177200.17 | *CHD9* | 1.7 | 1.86E-08 | 318.6 | 551.5 |
| ENSG00000119397.16 | *CNTRL* | 6.8 | 3.42E-02 | 3.8 | 25.8 |
| ENSG00000005339.14 | *CREBBP* | 1.5 | 1.09E-04 | 396.5 | 605.5 |
| ENSG00000178971.16 | *CTC1* | 2.0 | 6.39E-04 | 88.5 | 173.4 |
| ENSG00000163510.14 | *CWC22* | 1.8 | 3.72E-03 | 198.1 | 350.0 |
| ENSG00000018610.14 | *CXorf56* | 15.8 | 7.98E-05 | 1.4 | 22.3 |
| ENSG00000090520.11 | *DNAJB11* | -3.9 | 4.18E-02 | 17.9 | 4.6 |
| ENSG00000096696.14 | *DSP* | 3.7 | 6.07E-11 | 29.6 | 111.1 |
| ENSG00000151914.20 | *DST* | 2.0 | 1.83E-02 | 1518.7 | 3033.0 |
| ENSG00000197102.12 | *DYNC1H1* | 1.6 | 8.69E-03 | 922.5 | 1494.1 |
| ENSG00000102189.17 | *EEA1* | 1.5 | 1.32E-02 | 487.2 | 751.9 |
| ENSG00000172638.13 | *EFEMP2* | -1.6 | 4.00E-02 | 104.4 | 64.6 |
| ENSG00000184708.18 | *EIF4ENIF1* | 1.6 | 4.01E-02 | 170.7 | 269.5 |
| ENSG00000116016.14 | *EPAS1* | -2.3 | 2.29E-02 | 76.7 | 33.9 |
| ENSG00000112425.15 | *EPM2A* | -1.7 | 3.92E-02 | 55.7 | 32.6 |
| ENSG00000163161.13 | *ERCC3* | 1.9 | 1.32E-02 | 72.3 | 138.5 |
| ENSG00000225830.13 | *ERCC6* | -1.5 | 3.35E-05 | 253.6 | 164.9 |
| ENSG00000143971.9 | *ETAA1* | 1.7 | 6.60E-06 | 189.3 | 315.8 |
| ENSG00000105755.8 | *ETHE1* | -1.6 | 2.59E-09 | 444.9 | 282.6 |
| ENSG00000067208.14 | *EVI5* | 2.0 | 1.93E-02 | 85.6 | 167.7 |
| ENSG00000248905.9 | *FMN1* | 1.8 | 2.04E-04 | 264.2 | 469.6 |
| ENSG00000115414.19 | *FN1* | -1.8 | 3.99E-02 | 5522.5 | 3018.3 |
| ENSG00000170345.10 | *FOS* | -1.5 | 1.46E-02 | 16.9 | 10.9 |
| ENSG00000175592.9 | *FOSL1* | 2.2 | 9.97E-35 | 966.8 | 2120.4 |
| ENSG00000114861.20 | *FOXP1* | 1.6 | 3.87E-03 | 97.5 | 156.5 |
| ENSG00000117228.10 | *GBP1* | -1.9 | 1.35E-03 | 163.9 | 86.2 |
| ENSG00000115419.12 | *GLS* | 1.7 | 3.31E-02 | 44.7 | 78.2 |
| ENSG00000144591.18 | *GMPPA* | 1.6 | 3.11E-02 | 71.8 | 114.5 |
| ENSG00000127920.6 | *GNG11* | -1.7 | 2.14E-02 | 90.5 | 54.0 |
| ENSG00000175265.17 | *GOLGA8A* | 1.8 | 3.49E-03 | 290.4 | 536.0 |
| ENSG00000215252.11 | *GOLGA8B* | 1.7 | 9.22E-04 | 231.0 | 402.8 |
| ENSG00000116580.18 | *GON4L* | 1.5 | 2.70E-03 | 383.1 | 583.5 |
| ENSG00000068097.14 | *HEATR6* | 1.6 | 2.63E-03 | 144.0 | 230.8 |
| ENSG00000138646.9 | *HERC5* | -2.3 | 2.82E-04 | 123.2 | 54.6 |
| ENSG00000054392.13 | *HHAT* | -2.2 | 6.27E-03 | 55.5 | 24.8 |
| ENSG00000234745.11 | *HLA-B* | -1.8 | 4.82E-40 | 4018.4 | 2286.5 |
| ENSG00000270757.1 | *HSPE1-MOB4* | -148.0 | 2.50E-04 | 27.6 | 0.2 |
| ENSG00000090339.9 | *ICAM1* | -1.5 | 1.68E-03 | 178.4 | 116.4 |
| ENSG00000119632.4 | *IFI27L2* | -1.7 | 5.27E-03 | 109.8 | 62.9 |
| ENSG00000137965.11 | *IFI44* | -1.5 | 2.61E-05 | 453.9 | 296.8 |
| ENSG00000126709.15 | *IFI6* | -1.6 | 1.84E-02 | 127.1 | 81.0 |
| ENSG00000115267.8 | *IFIH1* | -2.2 | 9.57E-04 | 65.3 | 29.3 |
| ENSG00000185745.10 | *IFIT1* | -2.4 | 1.37E-42 | 671.0 | 280.2 |
| ENSG00000119922.10 | *IFIT2* | -6569283.0 | 4.81E-08 | 73.4 | 0.0 |
| ENSG00000119917.14 | *IFIT3* | -2.2 | 8.49E-31 | 921.2 | 411.5 |
| ENSG00000146674.15 | *IGFBP3* | 2.5 | 1.74E-15 | 311.1 | 776.5 |
| ENSG00000141753.7 | *IGFBP4* | -2.2 | 2.01E-03 | 65.8 | 30.2 |
| ENSG00000167779.9 | *IGFBP6* | -1.7 | 5.20E-04 | 240.4 | 142.7 |
| ENSG00000163453.11 | *IGFBP7* | -2.1 | 1.50E-19 | 625.0 | 302.5 |
| ENSG00000150782.12 | *IL18* | -1.8 | 2.24E-21 | 700.0 | 394.2 |
| ENSG00000008517.16 | *IL32* | -2.8 | 1.63E-04 | 119.3 | 43.2 |
| ENSG00000187608.10 | *ISG15* | -2.5 | 4.82E-27 | 1017.4 | 414.8 |
| ENSG00000261796.1 | *ISY1-RAB43* | -1.5 | 1.04E-02 | 251.9 | 166.0 |
| ENSG00000198885.9 | *ITPRIPL1* | 2.7 | 3.96E-03 | 15.8 | 43.4 |
| ENSG00000120071.14 | *KANSL1* | 1.6 | 6.06E-04 | 251.5 | 411.0 |
| ENSG00000118193.12 | *KIF14* | 1.8 | 1.36E-02 | 808.9 | 1425.3 |
| ENSG00000138182.14 | *KIF20B* | 1.9 | 2.04E-04 | 943.8 | 1789.5 |
| ENSG00000128422.17 | *KRT17* | -4.1 | 5.28E-21 | 377.6 | 92.5 |
| ENSG00000205426.10 | *KRT81* | -2.3 | 4.73E-05 | 154.7 | 67.0 |
| ENSG00000126777.18 | *KTN1* | 2.0 | 2.99E-03 | 2857.6 | 5834.4 |
| ENSG00000198910.14 | *L1CAM* | -2.0 | 4.42E-12 | 938.8 | 465.8 |
| ENSG00000188186.10 | *LAMTOR4* | -1.5 | 3.85E-02 | 110.4 | 71.4 |
| ENSG00000167615.16 | *LENG8* | 1.7 | 1.17E-04 | 363.3 | 611.1 |
| ENSG00000072163.19 | *LIMS2* | -12.2 | 5.00E-03 | 32.5 | 2.7 |
| ENSG00000232931.6 | *LINC00342* | 1.8 | 8.74E-03 | 115.9 | 208.6 |
| ENSG00000103227.18 | *LMF1* | -6.5 | 4.62E-02 | 28.4 | 4.4 |
| ENSG00000129038.16 | *LOXL1* | -1.7 | 5.44E-11 | 615.6 | 352.3 |
| ENSG00000198589.13 | *LRBA* | 1.7 | 2.99E-04 | 273.4 | 465.5 |
| ENSG00000150551.11 | *LYPD1* | -2.3 | 5.47E-05 | 88.8 | 38.3 |
| ENSG00000143353.12 | *LYPLAL1* | -1.7 | 1.26E-02 | 198.6 | 114.5 |
| ENSG00000081189.15 | *MEF2C* | 2.6 | 3.85E-02 | 14.0 | 35.7 |
| ENSG00000214548.18 | *MEG3* | 1.6 | 1.27E-07 | 1131.1 | 1801.7 |
| ENSG00000223403.6 | *MEG9* | 2.6 | 1.41E-02 | 12.4 | 32.4 |
| ENSG00000174197.16 | *MGA* | 18.5 | 7.49E-05 | 2.5 | 46.2 |
| ENSG00000262454.4 | *MIR193BHG* | 1.5 | 4.71E-02 | 73.2 | 113.0 |
| ENSG00000148773.14 | *MKI67* | 1.6 | 1.39E-04 | 5968.5 | 9268.4 |
| ENSG00000087245.13 | *MMP2* | -1.8 | 9.00E-05 | 196.3 | 111.4 |
| ENSG00000125966.10 | *MMP24* | -1.9 | 3.87E-03 | 143.8 | 76.8 |
| ENSG00000107186.16 | *MPDZ* | 1.9 | 4.94E-04 | 99.0 | 186.8 |
| ENSG00000196588.17 | *MRTFA* | 1.6 | 4.92E-02 | 129.8 | 201.8 |
| ENSG00000122033.14 | *MTIF3* | 2.6 | 3.85E-02 | 13.4 | 34.8 |
| ENSG00000198695.2 | *MT-ND6* | 1.7 | 8.76E-04 | 407.0 | 679.3 |
| ENSG00000013364.19 | *MVP* | -1.6 | 5.52E-03 | 406.3 | 247.7 |
| ENSG00000132382.14 | *MYBBP1A* | 1.9 | 1.53E-03 | 190.9 | 362.5 |
| ENSG00000172936.14 | *MYD88* | -2.4 | 1.58E-02 | 64.7 | 27.1 |
| ENSG00000124357.13 | *NAGK* | -1.6 | 4.48E-04 | 358.9 | 221.2 |
| ENSG00000128609.15 | *NDUFA5* | 1.6 | 4.69E-02 | 261.3 | 413.5 |
| ENSG00000245532.9 | *NEAT1* | 1.7 | 3.22E-04 | 1180.8 | 2002.6 |
| ENSG00000165525.18 | *NEMF* | 1.7 | 2.98E-04 | 373.4 | 635.3 |
| ENSG00000164190.19 | *NIPBL* | 1.6 | 4.54E-03 | 1354.0 | 2219.4 |
| ENSG00000114857.18 | *NKTR* | 1.7 | 3.80E-04 | 633.7 | 1057.4 |
| ENSG00000166741.7 | *NNMT* | -1.9 | 1.59E-06 | 279.8 | 144.1 |
| ENSG00000119655.10 | *NPC2* | -1.7 | 4.52E-02 | 102.0 | 61.7 |
| ENSG00000243716.10 | *NPIPB5* | 1.6 | 1.45E-03 | 373.3 | 578.7 |
| ENSG00000078618.21 | *NRDC* | 1.9 | 3.50E-03 | 116.4 | 220.4 |
| ENSG00000126653.18 | *NSRP1* | 1.6 | 1.81E-03 | 260.9 | 410.6 |
| ENSG00000135318.12 | *NT5E* | -1.7 | 9.77E-09 | 866.8 | 516.7 |
| ENSG00000154358.21 | *OBSCN* | 1.6 | 4.36E-02 | 140.9 | 220.1 |
| ENSG00000099864.18 | *PALM* | -1.9 | 3.72E-03 | 172.0 | 92.3 |
| ENSG00000182752.10 | *PAPPA* | -3.1 | 2.42E-02 | 21.8 | 7.2 |
| ENSG00000118482.11 | *PHF3* | 1.5 | 3.42E-02 | 723.9 | 1107.3 |
| ENSG00000146247.14 | *PHIP* | 2.2 | 1.57E-03 | 103.1 | 226.6 |
| ENSG00000176485.11 | *PLAAT3* | -3.7 | 3.87E-03 | 78.4 | 21.3 |
| ENSG00000133321.11 | *PLAAT4* | -3.5 | 3.56E-08 | 86.1 | 24.4 |
| ENSG00000122861.16 | *PLAU* | -1.7 | 1.61E-03 | 261.1 | 154.9 |
| ENSG00000107020.10 | *PLGRKT* | -1.5 | 3.62E-02 | 126.5 | 82.0 |
| ENSG00000196313.11 | *POM121* | 1.8 | 3.76E-02 | 34.7 | 60.8 |
| ENSG00000133110.15 | *POSTN* | -1.8 | 3.46E-17 | 4481.7 | 2516.8 |
| ENSG00000101000.6 | *PROCR* | -1.5 | 9.95E-04 | 343.2 | 225.4 |
| ENSG00000240065.8 | *PSMB9* | -1.9 | 1.65E-02 | 69.9 | 36.4 |
| ENSG00000171862.11 | *PTEN* | 1.6 | 9.96E-03 | 322.5 | 516.0 |
| ENSG00000139304.16 | *PTPRQ* | 3.0 | 2.56E-02 | 47.3 | 144.3 |
| ENSG00000136828.19 | *RALGPS1* | 2.1 | 4.25E-02 | 23.6 | 49.7 |
| ENSG00000153201.16 | *RANBP2* | 2.1 | 2.72E-02 | 1306.0 | 2679.0 |
| ENSG00000242389.8 | *RBMY1E* | 2.5 | 5.44E-03 | 76.7 | 189.7 |
| ENSG00000157110.16 | *RBPMS* | -1.6 | 1.76E-03 | 151.0 | 97.4 |
| ENSG00000166831.9 | *RBPMS2* | -2.5 | 2.69E-02 | 26.3 | 10.5 |
| ENSG00000080345.18 | *RIF1* | 1.9 | 1.85E-03 | 784.6 | 1478.7 |
| ENSG00000145860.11 | *RNF145* | -1.9 | 3.93E-02 | 328.6 | 175.3 |
| ENSG00000101236.17 | *RNF24* | -1.5 | 3.23E-02 | 314.2 | 206.4 |
| ENSG00000169855.20 | *ROBO1* | 1.5 | 2.53E-03 | 478.0 | 732.9 |
| ENSG00000067900.8 | *ROCK1* | 1.6 | 1.22E-02 | 601.6 | 939.0 |
| ENSG00000215472.10 | *RPL17-C18orf32* | -1.6 | 3.08E-02 | 188.8 | 118.9 |
| ENSG00000048649.13 | *RSF1* | 2.0 | 5.66E-03 | 211.6 | 424.4 |
| ENSG00000075826.17 | *SEC31B* | 2.0 | 3.55E-02 | 25.8 | 50.5 |
| ENSG00000205572.9 | *SERF1B* | -3.6 | 1.33E-02 | 36.7 | 10.1 |
| ENSG00000080546.13 | *SESN1* | -1.6 | 2.21E-02 | 95.7 | 59.4 |
| ENSG00000163535.18 | *SGO2* | 1.8 | 7.32E-03 | 287.8 | 512.8 |
| ENSG00000130813.18 | *SHFL* | -1.7 | 2.54E-02 | 78.3 | 47.1 |
| ENSG00000138944.8 | *SHISAL1* | -1.7 | 3.80E-02 | 58.8 | 35.1 |
| ENSG00000074621.14 | *SLC24A1* | -2.0 | 1.54E-02 | 126.2 | 64.0 |
| ENSG00000022567.9 | *SLC45A4* | 3.7 | 1.42E-02 | 22.3 | 83.0 |
| ENSG00000154760.14 | *SLFN13* | 2.4 | 4.68E-06 | 45.9 | 110.1 |
| ENSG00000163104.17 | *SMARCAD1* | 1.7 | 4.58E-03 | 241.8 | 404.5 |
| ENSG00000113810.16 | *SMC4* | 1.6 | 6.25E-04 | 4174.9 | 6620.1 |
| ENSG00000172164.15 | *SNTB1* | 2.4 | 6.38E-03 | 19.3 | 47.4 |
| ENSG00000071189.21 | *SNX13* | 1.6 | 4.08E-02 | 307.7 | 483.8 |
| ENSG00000120833.14 | *SOCS2* | -1.5 | 2.49E-02 | 124.0 | 80.1 |
| ENSG00000115904.12 | *SOS1* | 2.1 | 1.86E-02 | 42.4 | 87.5 |
| ENSG00000137767.14 | *SQOR* | -1.6 | 1.20E-02 | 119.8 | 73.7 |
| ENSG00000153914.16 | *SREK1* | 1.5 | 3.17E-02 | 954.5 | 1476.9 |
| ENSG00000266028.7 | *SRGAP2* | 1.5 | 3.52E-03 | 178.1 | 274.7 |
| ENSG00000101972.18 | *STAG2* | 1.7 | 3.37E-04 | 655.0 | 1145.6 |
| ENSG00000137868.19 | *STRA6* | -4.0 | 2.72E-07 | 81.7 | 20.2 |
| ENSG00000196562.14 | *SULF2* | -5.0 | 4.34E-02 | 56.5 | 11.2 |
| ENSG00000054654.16 | *SYNE2* | 1.8 | 8.89E-04 | 303.8 | 557.4 |
| ENSG00000159082.17 | *SYNJ1* | -137.4 | 1.13E-03 | 25.8 | 0.2 |
| ENSG00000149591.17 | *TAGLN* | -2.3 | 1.63E-15 | 453.9 | 194.4 |
| ENSG00000163946.13 | *TASOR* | 1.6 | 5.47E-05 | 523.2 | 822.3 |
| ENSG00000186340.15 | *THBS2* | -14.9 | 4.13E-02 | 29.6 | 2.0 |
| ENSG00000170006.12 | *TMEM154* | 3.9 | 3.96E-03 | 7.2 | 28.4 |
| ENSG00000011638.10 | *TMEM159* | -4.2 | 2.59E-05 | 73.1 | 17.6 |
| ENSG00000154646.9 | *TMPRSS15* | 2.1 | 1.73E-02 | 41.1 | 87.2 |
| ENSG00000047410.14 | *TPR* | 2.4 | 2.14E-03 | 1491.7 | 3509.3 |
| ENSG00000132481.7 | *TRIM47* | -1.7 | 1.14E-02 | 97.0 | 58.4 |
| ENSG00000131002.12 | *TXLNGY* | 1.9 | 3.07E-04 | 112.0 | 208.3 |
| ENSG00000137831.15 | *UACA* | 1.7 | 2.85E-03 | 1021.9 | 1769.4 |
| ENSG00000156587.16 | *UBE2L6* | -1.7 | 3.12E-02 | 86.2 | 51.2 |
| ENSG00000148429.14 | *USP6NL* | -3.5 | 1.31E-02 | 83.6 | 23.7 |
| ENSG00000118640.11 | *VAMP8* | -1.9 | 4.80E-02 | 43.2 | 22.8 |
| ENSG00000004766.17 | *VPS50* | 1.6 | 6.41E-03 | 219.5 | 354.4 |
| ENSG00000133316.15 | *WDR74* | 1.5 | 2.15E-02 | 83.6 | 129.0 |
| ENSG00000105989.10 | *WNT2* | -3.3 | 1.03E-03 | 40.1 | 12.0 |
| ENSG00000180667.10 | *YOD1* | -5510218.1 | 7.50E-08 | 60.8 | 0.0 |
| ENSG00000148516.21 | *ZEB1* | 1.5 | 2.03E-02 | 353.6 | 539.0 |
| ENSG00000005889.15 | *ZFX* | 1.7 | 3.72E-03 | 392.2 | 667.5 |
| ENSG00000043355.12 | *ZIC2* | 1.7 | 3.44E-02 | 92.3 | 158.3 |
| ENSG00000123870.10 | *ZNF137P* | 4.9 | 3.33E-03 | 3.7 | 18.3 |
| ENSG00000158805.12 | *ZNF276* | 1.5 | 1.89E-02 | 211.5 | 323.7 |
| ENSG00000234420.7 | *ZNF37BP* | 1.7 | 4.67E-04 | 116.1 | 192.2 |
| ENSG00000144331.20 | *ZNF385B* | 1.5 | 5.20E-04 | 129.0 | 197.2 |
| ENSG00000213020.10 | *ZNF611* | 2.1 | 2.67E-02 | 20.7 | 42.9 |
| ENSG00000177873.14 | *ZNF619* | 1.8 | 1.11E-02 | 34.8 | 63.4 |
| ENSG00000242779.7 | *ZNF702P* | 2.7 | 2.69E-02 | 12.9 | 34.6 |
| ENSG00000198040.10 | *ZNF84* | 1.6 | 1.41E-02 | 99.8 | 163.0 |
| ENSG00000106479.11 | *ZNF862* | -3.5 | 3.03E-02 | 28.6 | 8.3 |

**Supplementary Table 3. Differentially expressed genes in CGL1 control cells (Cas9 and dCas9) 2 hours after serum stimulation relative to baseline.**

To characterize the early transcriptional response of CGL1 cells to serum stimulation, RNA-sequencing was performed on CGL1^Cas9^ and CGL1^dCas9^ control cells collected 2 hours after media change and compared to their respective baseline (0-hour) condition. As no differentially expressed genes (DEGs) were identified between Cas9 and dCas9 cells at baseline, these samples were pooled for analysis. A total of 639 DEGs were identified using a fold change (F.C.) threshold >1.5 or <–1.5 and an FDR-adjusted *p*-value (p-adj) < 0.05, comprising 337 upregulated and 302 downregulated genes. This dataset reflects the early gene expression program activated by mitogenic signaling in wild-type CGL1 cells. The table includes gene names, fold changes, p-adj values, and transcript abundance (TPM), sorted alphabetically.

| **Gene ID** | **Gene Name** | **F.C.** | **p-adj.** | **Baseline** | **2h Post Serum** |
| --- | --- | --- | --- | --- | --- |
| ENSG00000157426.14 | *AASDH* | -1.5 | 1.26E-04 | 110.8 | 72.2 |
| ENSG00000106077.18 | *ABHD11* | 2.1 | 1.52E-03 | 20.9 | 43.2 |
| ENSG00000143322.21 | *ABL2* | 1.9 | 8.49E-07 | 540.6 | 1019.9 |
| ENSG00000244198.7 | *AC004889.1* | 2.0 | 2.05E-02 | 29.6 | 58.5 |
| ENSG00000229180.7 | *AC006001.3* | 2.1 | 1.22E-17 | 125.5 | 260.5 |
| ENSG00000277534.1 | *AC007996.1* | 1.9 | 1.20E-04 | 37.9 | 72.0 |
| ENSG00000204758.8 | *AC008429.1* | 1.5 | 4.44E-02 | 36.8 | 56.9 |
| ENSG00000285517.1 | *AC010198.2* | 1.7 | 2.47E-03 | 85.6 | 147.3 |
| ENSG00000273184.1 | *AC010655.4* | 2.8 | 3.68E-02 | 10.7 | 29.8 |
| ENSG00000278600.1 | *AC015871.4* | 1.8 | 3.72E-02 | 20.7 | 38.3 |
| ENSG00000226380.9 | *AC016831.1* | 4.7 | 2.72E-09 | 8.1 | 38.6 |
| ENSG00000285106.2 | *AC016831.7* | 3.0 | 3.19E-04 | 10.9 | 32.6 |
| ENSG00000267519.6 | *AC020916.1* | 2.9 | 8.43E-30 | 274.9 | 802.0 |
| ENSG00000230551.4 | *AC021078.1* | 1.5 | 9.20E-05 | 177.6 | 269.9 |
| ENSG00000279092.1 | *AC025678.3* | 2.3 | 1.62E-02 | 9.1 | 20.7 |
| ENSG00000280138.1 | *AC027290.2* | 2.0 | 8.35E-04 | 23.6 | 46.3 |
| ENSG00000253200.1 | *AC037459.2* | 1.6 | 1.34E-02 | 41.1 | 64.2 |
| ENSG00000262003.1 | *AC087392.1* | 3.5 | 1.61E-04 | 5.9 | 20.7 |
| ENSG00000230606.11 | *AC092683.1* | 1.6 | 2.07E-02 | 71.2 | 117.2 |
| ENSG00000270562.1 | *AC097634.1* | 3.0 | 2.73E-03 | 6.7 | 19.8 |
| ENSG00000274383.1 | *AC103691.1* | 2.8 | 2.65E-02 | 5.9 | 16.6 |
| ENSG00000287126.1 | *AC108488.3* | -1.7 | 4.88E-02 | 25.9 | 15.3 |
| ENSG00000271643.2 | *AC112220.2* | 1.7 | 4.45E-03 | 39.1 | 67.2 |
| ENSG00000280407.2 | *AC132872.4* | 3.5 | 6.53E-04 | 4.7 | 16.7 |
| ENSG00000226268.3 | *AC135977.1* | 1.5 | 2.48E-03 | 51.0 | 78.5 |
| ENSG00000277701.5 | *AC159540.2* | 1.7 | 3.60E-02 | 80.6 | 135.1 |
| ENSG00000274471.2 | *AC242376.2* | -2.1 | 2.34E-02 | 27.6 | 13.4 |
| ENSG00000154734.15 | *ADAMTS1* | -2.2 | 1.52E-14 | 336.8 | 155.6 |
| ENSG00000272734.1 | *ADIRF-AS1* | 1.8 | 1.47E-04 | 44.7 | 82.5 |
| ENSG00000148926.10 | *ADM* | 2.4 | 1.83E-47 | 590.7 | 1399.5 |
| ENSG00000128165.9 | *ADM2* | 2.2 | 5.87E-03 | 51.9 | 115.7 |
| ENSG00000181026.15 | *AEN* | 1.6 | 6.94E-20 | 926.1 | 1444.4 |
| ENSG00000131016.17 | *AKAP12* | 3.8 | 1.03E-67 | 2099.1 | 8073.2 |
| ENSG00000287562.1 | *AL109615.4* | -2.0 | 2.13E-02 | 23.0 | 11.6 |
| ENSG00000285796.1 | *AL162458.1* | -1.9 | 9.19E-03 | 37.2 | 20.1 |
| ENSG00000198796.7 | *ALPK2* | 2.2 | 2.54E-33 | 366.4 | 799.1 |
| ENSG00000181754.7 | *AMIGO1* | -1.9 | 5.18E-03 | 66.4 | 34.3 |
| ENSG00000139211.6 | *AMIGO2* | 9.5 | 3.58E-11 | 65.0 | 618.5 |
| ENSG00000114019.14 | *AMOTL2* | 1.5 | 2.33E-15 | 2798.7 | 4330.1 |
| ENSG00000187689.10 | *AMTN* | -1.8 | 3.84E-02 | 122.5 | 69.2 |
| ENSG00000167772.12 | *ANGPTL4* | 8.1 | 4.33E-15 | 24.6 | 199.6 |
| ENSG00000137494.14 | *ANKRD42* | 1.5 | 8.64E-04 | 88.4 | 134.8 |
| ENSG00000272578.5 | *AP000347.1* | 2.9 | 3.37E-02 | 10.0 | 28.6 |
| ENSG00000173727.12 | *AP000769.1* | -1.8 | 3.63E-02 | 30.7 | 17.3 |
| ENSG00000228463.10 | *AP006222.1* | -1.9 | 1.98E-02 | 32.5 | 17.1 |
| ENSG00000198576.4 | *ARC* | 14.6 | 7.02E-18 | 3.6 | 52.4 |
| ENSG00000204959.4 | *ARHGEF34P* | 1.6 | 2.73E-02 | 63.7 | 102.7 |
| ENSG00000137135.18 | *ARHGEF39* | -1.5 | 6.47E-07 | 299.5 | 194.4 |
| ENSG00000116017.11 | *ARID3A* | 2.8 | 9.72E-10 | 82.7 | 233.0 |
| ENSG00000196843.16 | *ARID5A* | 1.9 | 3.84E-07 | 103.6 | 199.2 |
| ENSG00000122644.13 | *ARL4A* | -1.7 | 6.41E-12 | 419.4 | 250.7 |
| ENSG00000188042.8 | *ARL4C* | 2.5 | 6.26E-07 | 29.0 | 71.3 |
| ENSG00000175906.5 | *ARL4D* | -1.7 | 7.53E-05 | 92.9 | 53.4 |
| ENSG00000165997.5 | *ARL5B* | 1.8 | 2.03E-20 | 701.7 | 1268.3 |
| ENSG00000125962.14 | *ARMCX5* | 4.9 | 1.39E-03 | 9.2 | 45.5 |
| ENSG00000183475.12 | *ASB7* | 3.3 | 1.89E-02 | 10.3 | 34.2 |
| ENSG00000162772.17 | *ATF3* | 3.3 | 1.59E-41 | 255.4 | 834.6 |
| ENSG00000123395.14 | *ATG101* | 2.0 | 1.43E-02 | 32.6 | 66.7 |
| ENSG00000170340.11 | *B3GNT2* | 1.5 | 3.28E-06 | 178.4 | 273.9 |
| ENSG00000176597.12 | *B3GNT5* | 2.3 | 6.60E-04 | 22.6 | 51.0 |
| ENSG00000156273.16 | *BACH1* | 2.3 | 1.39E-19 | 458.5 | 1065.4 |
| ENSG00000166170.9 | *BAG5* | 3.1 | 3.78E-02 | 15.8 | 49.4 |
| ENSG00000123636.18 | *BAZ2B* | 1.9 | 1.42E-02 | 122.6 | 236.5 |
| ENSG00000181004.10 | *BBS12* | -2.4 | 4.13E-03 | 28.0 | 11.8 |
| ENSG00000050820.17 | *BCAR1* | 1.9 | 7.36E-40 | 786.9 | 1494.0 |
| ENSG00000137936.18 | *BCAR3* | 1.8 | 1.94E-08 | 400.8 | 729.4 |
| ENSG00000186666.6 | *BCDIN3D* | -1.8 | 5.38E-03 | 61.0 | 33.3 |
| ENSG00000142867.14 | *BCL10* | 2.5 | 1.58E-24 | 215.4 | 536.2 |
| ENSG00000069399.15 | *BCL3* | 3.3 | 5.55E-15 | 70.5 | 230.9 |
| ENSG00000116128.11 | *BCL9* | 2.2 | 1.02E-04 | 68.3 | 153.7 |
| ENSG00000183337.17 | *BCOR* | 2.1 | 5.80E-13 | 493.8 | 1023.6 |
| ENSG00000176697.19 | *BDNF* | 2.2 | 2.38E-55 | 582.9 | 1298.3 |
| ENSG00000183092.16 | *BEGAIN* | 1.9 | 4.76E-02 | 155.1 | 289.7 |
| ENSG00000177951.17 | *BET1L* | 1.9 | 2.45E-03 | 47.3 | 87.8 |
| ENSG00000134107.5 | *BHLHE40* | 18.6 | 1.04E-251 | 133.1 | 2476.8 |
| ENSG00000151746.14 | *BICD1* | 1.8 | 1.81E-12 | 218.8 | 387.4 |
| ENSG00000125378.16 | *BMP4* | -1.7 | 6.57E-06 | 1337.1 | 784.2 |
| ENSG00000196544.8 | *BORCS6* | -1.8 | 3.77E-06 | 93.9 | 53.4 |
| ENSG00000130921.8 | *C12orf65* | -1.8 | 2.25E-06 | 210.7 | 115.6 |
| ENSG00000167173.19 | *C15orf39* | 1.9 | 9.31E-13 | 327.8 | 636.5 |
| ENSG00000108509.21 | *CAMTA2* | 1.7 | 1.16E-05 | 314.5 | 535.1 |
| ENSG00000274070.2 | *CASTOR2* | -1.6 | 2.29E-02 | 155.8 | 98.5 |
| ENSG00000099625.13 | *CBARP* | 3.4 | 2.92E-09 | 29.6 | 102.1 |
| ENSG00000141582.15 | *CBX4* | 1.7 | 2.64E-08 | 275.9 | 470.5 |
| ENSG00000141570.11 | *CBX8* | -1.5 | 1.70E-04 | 146.2 | 94.9 |
| ENSG00000164366.4 | *CCDC127* | -1.6 | 2.31E-13 | 593.7 | 381.4 |
| ENSG00000154781.16 | *CCDC174* | 1.7 | 5.17E-04 | 127.9 | 216.9 |
| ENSG00000141519.15 | *CCDC40* | 2.2 | 9.63E-03 | 12.5 | 27.8 |
| ENSG00000105321.14 | *CCDC9* | 1.8 | 8.82E-17 | 217.7 | 399.5 |
| ENSG00000108691.9 | *CCL2* | 3.1 | 2.32E-07 | 85.8 | 266.2 |
| ENSG00000142871.17 | *CCN1* | 2.4 | 2.27E-126 | 6655.7 | 16147.7 |
| ENSG00000118523.6 | *CCN2* | 10.6 | 7.55E-218 | 1691.9 | 17968.0 |
| ENSG00000175305.17 | *CCNE2* | 2.5 | 4.37E-02 | 9.8 | 24.4 |
| ENSG00000162063.13 | *CCNF* | -1.7 | 4.95E-26 | 856.4 | 500.5 |
| ENSG00000138764.15 | *CCNG2* | -2.2 | 6.76E-07 | 185.8 | 83.1 |
| ENSG00000082258.13 | *CCNT2* | 1.7 | 1.70E-02 | 196.6 | 330.4 |
| ENSG00000120217.14 | *CD274* | 5.5 | 1.50E-07 | 16.4 | 89.9 |
| ENSG00000117877.10 | *CD3EAP* | 1.5 | 1.11E-02 | 244.5 | 376.9 |
| ENSG00000112149.10 | *CD83* | 1.6 | 4.72E-07 | 143.8 | 234.1 |
| ENSG00000070831.17 | *CDC42* | -2.8 | 2.84E-02 | 102.5 | 36.2 |
| ENSG00000149798.5 | *CDC42EP2* | 1.5 | 5.10E-09 | 211.9 | 323.6 |
| ENSG00000197622.13 | *CDC42SE1* | 1.5 | 2.48E-11 | 909.5 | 1399.4 |
| ENSG00000111276.11 | *CDKN1B* | -1.9 | 9.39E-11 | 1003.4 | 533.7 |
| ENSG00000221869.5 | *CEBPD* | 1.6 | 1.55E-04 | 121.5 | 190.0 |
| ENSG00000174799.11 | *CEP135* | 1.8 | 5.36E-04 | 100.7 | 184.2 |
| ENSG00000128965.13 | *CHAC1* | 6.5 | 1.18E-12 | 67.4 | 436.6 |
| ENSG00000177200.17 | *CHD9* | 1.6 | 1.32E-02 | 336.9 | 551.4 |
| ENSG00000131873.7 | *CHSY1* | 1.6 | 3.77E-20 | 607.8 | 1000.3 |
| ENSG00000179862.7 | *CITED4* | 3.3 | 1.07E-11 | 20.3 | 67.9 |
| ENSG00000175505.11 | *CLCF1* | 2.5 | 5.67E-29 | 121.8 | 308.3 |
| ENSG00000163347.6 | *CLDN1* | 2.8 | 3.66E-08 | 20.4 | 56.7 |
| ENSG00000038532.16 | *CLEC16A* | 2.2 | 9.31E-03 | 29.9 | 65.8 |
| ENSG00000187955.12 | *COL14A1* | 2.8 | 3.24E-02 | 8.8 | 25.0 |
| ENSG00000262246.6 | *CORO7* | 1.7 | 1.49E-04 | 131.9 | 227.5 |
| ENSG00000113742.14 | *CPEB4* | 1.6 | 4.28E-02 | 53.3 | 83.8 |
| ENSG00000146592.17 | *CREB5* | 2.0 | 2.94E-07 | 203.1 | 408.2 |
| ENSG00000103196.12 | *CRISPLD2* | 1.9 | 7.69E-04 | 184.0 | 355.7 |
| ENSG00000169826.8 | *CSGALNACT2* | 1.6 | 5.77E-09 | 362.5 | 582.7 |
| ENSG00000204435.13 | *CSNK2B* | 1.9 | 1.96E-06 | 57.1 | 109.3 |
| ENSG00000144655.15 | *CSRNP1* | 5.4 | 3.05E-104 | 157.6 | 854.9 |
| ENSG00000159176.14 | *CSRP1* | 1.7 | 1.25E-31 | 1646.4 | 2816.6 |
| ENSG00000169429.11 | *CXCL8* | 9.7 | 3.49E-13 | 5.7 | 54.9 |
| ENSG00000138061.12 | *CYP1B1* | 1.5 | 2.04E-15 | 1390.8 | 2124.9 |
| ENSG00000100592.15 | *DAAM1* | 2.4 | 6.27E-05 | 47.3 | 115.6 |
| ENSG00000167657.14 | *DAPK3* | 1.5 | 1.98E-20 | 727.9 | 1117.5 |
| ENSG00000188215.10 | *DCUN1D3* | 2.2 | 3.56E-10 | 141.7 | 309.9 |
| ENSG00000175197.12 | *DDIT3* | 2.8 | 4.56E-06 | 52.8 | 148.1 |
| ENSG00000168209.5 | *DDIT4* | 3.2 | 1.37E-06 | 519.7 | 1637.4 |
| ENSG00000181418.8 | *DDN* | -1.6 | 1.45E-02 | 54.4 | 33.8 |
| ENSG00000136267.13 | *DGKB* | 1.5 | 1.70E-02 | 209.2 | 323.5 |
| ENSG00000162496.9 | *DHRS3* | 3.0 | 1.26E-02 | 40.2 | 120.7 |
| ENSG00000140323.6 | *DISP2* | -5.9 | 1.88E-03 | 45.0 | 7.7 |
| ENSG00000164741.15 | *DLC1* | 2.5 | 1.41E-61 | 393.0 | 1001.3 |
| ENSG00000115844.11 | *DLX2* | 4.2 | 5.08E-46 | 97.6 | 405.3 |
| ENSG00000176399.4 | *DMRTA1* | -2.5 | 5.34E-14 | 122.7 | 49.2 |
| ENSG00000135164.18 | *DMTF1* | 1.9 | 1.16E-05 | 229.0 | 436.2 |
| ENSG00000128590.5 | *DNAJB9* | -2.3 | 2.09E-12 | 140.9 | 60.6 |
| ENSG00000230630.5 | *DNM3OS* | 1.9 | 6.03E-06 | 113.3 | 211.4 |
| ENSG00000107554.17 | *DNMBP* | 2.1 | 1.66E-21 | 281.6 | 580.3 |
| ENSG00000125170.11 | *DOK4* | 1.7 | 3.30E-02 | 88.4 | 154.3 |
| ENSG00000096696.14 | *DSP* | 1.9 | 1.42E-02 | 36.6 | 67.9 |
| ENSG00000120129.6 | *DUSP1* | 2.6 | 1.55E-81 | 367.8 | 954.7 |
| ENSG00000158050.5 | *DUSP2* | 5.5 | 8.17E-74 | 98.4 | 544.2 |
| ENSG00000120875.9 | *DUSP4* | 8.4 | 3.23E-114 | 47.8 | 403.1 |
| ENSG00000138166.6 | *DUSP5* | 13.0 | 1.14E-242 | 69.5 | 905.3 |
| ENSG00000139318.8 | *DUSP6* | 5.6 | 1.73E-21 | 38.5 | 216.1 |
| ENSG00000184545.11 | *DUSP8* | 2.5 | 1.48E-08 | 118.8 | 294.6 |
| ENSG00000157540.21 | *DYRK1A* | 1.9 | 9.31E-13 | 433.7 | 824.8 |
| ENSG00000144597.14 | *EAF1* | 1.6 | 1.64E-11 | 380.2 | 600.3 |
| ENSG00000078401.7 | *EDN1* | 4.5 | 1.12E-73 | 162.2 | 729.9 |
| ENSG00000127129.10 | *EDN2* | 2.4 | 2.59E-02 | 8.9 | 21.3 |
| ENSG00000169242.12 | *EFNA1* | -1.6 | 1.62E-02 | 116.1 | 72.1 |
| ENSG00000243364.8 | *EFNA4* | -1.6 | 3.57E-02 | 87.6 | 54.1 |
| ENSG00000120738.8 | *EGR1* | 32.7 | 0.00E+00 | 149.4 | 4879.2 |
| ENSG00000122877.16 | *EGR2* | 68.7 | 1.56E-09 | 1.0 | 66.8 |
| ENSG00000179388.9 | *EGR3* | 146.6 | 1.70E-50 | 2.5 | 370.3 |
| ENSG00000115504.14 | *EHBP1* | 1.7 | 2.74E-03 | 82.4 | 139.0 |
| ENSG00000110047.18 | *EHD1* | 1.5 | 5.34E-06 | 1114.8 | 1715.8 |
| ENSG00000175376.9 | *EIF1AD* | 2.1 | 2.10E-29 | 257.7 | 547.4 |
| ENSG00000105656.13 | *ELL* | 1.7 | 3.15E-09 | 259.1 | 438.2 |
| ENSG00000118985.16 | *ELL2* | 1.6 | 5.73E-16 | 1205.7 | 1932.3 |
| ENSG00000180385.8 | *EMC3-AS1* | 1.8 | 9.68E-03 | 47.9 | 87.7 |
| ENSG00000197774.13 | *EME2* | 3.4 | 3.24E-02 | 21.6 | 72.4 |
| ENSG00000134531.10 | *EMP1* | 1.6 | 8.08E-09 | 1763.1 | 2762.5 |
| ENSG00000171617.14 | *ENC1* | 10.9 | 1.06E-17 | 14.3 | 154.7 |
| ENSG00000198018.7 | *ENTPD7* | 1.6 | 2.07E-06 | 160.8 | 251.1 |
| ENSG00000142627.13 | *EPHA2* | 3.1 | 2.41E-96 | 1897.5 | 5807.5 |
| ENSG00000187266.14 | *EPOR* | -1.8 | 2.69E-05 | 114.9 | 64.1 |
| ENSG00000261150.2 | *EPPK1* | 32.2 | 3.40E-03 | 5.8 | 185.3 |
| ENSG00000163161.13 | *ERCC3* | 1.7 | 6.41E-04 | 82.1 | 138.8 |
| ENSG00000182150.16 | *ERCC6L2* | 2.2 | 1.84E-02 | 49.9 | 107.7 |
| ENSG00000116285.13 | *ERRFI1* | 11.9 | 3.55E-175 | 973.4 | 11541.0 |
| ENSG00000134954.14 | *ETS1* | 2.9 | 5.11E-03 | 18.1 | 52.3 |
| ENSG00000157557.13 | *ETS2* | 1.8 | 1.50E-18 | 647.1 | 1154.2 |
| ENSG00000117036.12 | *ETV3* | 1.6 | 8.06E-20 | 349.8 | 561.5 |
| ENSG00000164002.11 | *EXO5* | 4.0 | 3.04E-02 | 5.7 | 22.9 |
| ENSG00000164251.5 | *F2RL1* | 1.8 | 5.74E-18 | 309.9 | 564.2 |
| ENSG00000117525.14 | *F3* | 7.6 | 2.67E-167 | 1306.8 | 9937.0 |
| ENSG00000167106.12 | *FAM102A* | 1.6 | 8.49E-07 | 190.4 | 303.7 |
| ENSG00000154319.16 | *FAM167A* | 1.7 | 8.72E-03 | 53.4 | 90.5 |
| ENSG00000185112.6 | *FAM43A* | 2.2 | 4.21E-14 | 81.3 | 180.4 |
| ENSG00000126882.13 | *FAM78A* | -2.7 | 1.93E-04 | 28.7 | 10.7 |
| ENSG00000101447.15 | *FAM83D* | -1.7 | 8.61E-35 | 1647.4 | 979.8 |
| ENSG00000188522.15 | *FAM83G* | 2.0 | 1.25E-23 | 507.4 | 1019.5 |
| ENSG00000180921.7 | *FAM83H* | 1.5 | 4.82E-09 | 581.1 | 882.6 |
| ENSG00000173295.7 | *FAM86B3P* | 3.0 | 9.71E-04 | 9.9 | 29.7 |
| ENSG00000158483.16 | *FAM86C1* | 2.4 | 3.72E-02 | 11.1 | 26.5 |
| ENSG00000162458.13 | *FBLIM1* | 1.7 | 8.96E-06 | 334.5 | 562.8 |
| ENSG00000180263.14 | *FGD6* | 1.6 | 4.37E-02 | 63.1 | 100.3 |
| ENSG00000183386.10 | *FHL3* | 1.7 | 3.05E-15 | 427.0 | 737.4 |
| ENSG00000176971.4 | *FIBIN* | 2.5 | 9.44E-03 | 7.6 | 18.9 |
| ENSG00000182263.14 | *FIGN* | -2.1 | 6.40E-04 | 146.4 | 68.7 |
| ENSG00000179431.7 | *FJX1* | 1.6 | 4.59E-17 | 376.8 | 592.5 |
| ENSG00000096060.14 | *FKBP5* | 2.5 | 2.13E-02 | 8.3 | 20.5 |
| ENSG00000204315.4 | *FKBPL* | -1.5 | 2.68E-03 | 139.8 | 90.3 |
| ENSG00000125848.10 | *FLRT3* | -2.5 | 1.34E-04 | 37.7 | 15.0 |
| ENSG00000170345.10 | *FOS* | 9.7 | 9.36E-43 | 15.7 | 152.3 |
| ENSG00000125740.14 | *FOSB* | 24.3 | 5.59E-48 | 18.9 | 460.4 |
| ENSG00000175592.9 | *FOSL1* | 3.9 | 2.89E-89 | 1167.6 | 4556.8 |
| ENSG00000075426.12 | *FOSL2* | 1.9 | 1.89E-30 | 1166.6 | 2256.1 |
| ENSG00000176692.8 | *FOXC2* | 4.7 | 7.99E-13 | 25.5 | 120.7 |
| ENSG00000251493.5 | *FOXD1* | 1.7 | 1.91E-05 | 178.7 | 310.4 |
| ENSG00000150907.9 | *FOXO1* | 1.8 | 6.27E-05 | 72.2 | 132.0 |
| ENSG00000114861.20 | *FOXP1* | 1.7 | 1.69E-04 | 108.1 | 178.5 |
| ENSG00000165879.9 | *FRAT1* | -3.0 | 2.48E-04 | 27.9 | 9.4 |
| ENSG00000181274.7 | *FRAT2* | -2.8 | 6.67E-29 | 223.8 | 79.4 |
| ENSG00000171877.21 | *FRMD5* | 1.6 | 6.55E-03 | 70.2 | 112.3 |
| ENSG00000265817.3 | *FSBP* | 1.9 | 8.78E-04 | 32.8 | 63.8 |
| ENSG00000106701.12 | *FSD1L* | 1.6 | 4.98E-02 | 71.0 | 114.2 |
| ENSG00000145907.15 | *G3BP1* | 1.6 | 1.96E-03 | 836.2 | 1307.1 |
| ENSG00000116717.13 | *GADD45A* | 3.4 | 1.61E-62 | 575.6 | 1969.2 |
| ENSG00000099860.9 | *GADD45B* | 2.1 | 1.15E-108 | 1189.3 | 2532.6 |
| ENSG00000253669.4 | *GASAL1* | 5.4 | 1.69E-13 | 12.3 | 66.6 |
| ENSG00000130513.6 | *GDF15* | 14.5 | 3.80E-49 | 49.1 | 713.1 |
| ENSG00000164949.8 | *GEM* | 2.9 | 1.79E-07 | 18.7 | 54.8 |
| ENSG00000141034.10 | *GID4* | -1.8 | 5.22E-12 | 155.9 | 85.5 |
| ENSG00000188910.8 | *GJB3* | 3.1 | 3.86E-05 | 15.3 | 47.2 |
| ENSG00000115419.12 | *GLS* | 1.7 | 1.77E-02 | 52.0 | 88.4 |
| ENSG00000143457.11 | *GOLPH3L* | -1.6 | 2.24E-05 | 263.8 | 166.6 |
| ENSG00000089916.18 | *GPATCH2L* | 1.8 | 2.60E-09 | 577.1 | 1049.2 |
| ENSG00000125772.13 | *GPCPD1* | 7.0 | 9.97E-04 | 7.8 | 54.0 |
| ENSG00000136235.16 | *GPNMB* | 1.6 | 2.08E-02 | 81.2 | 126.1 |
| ENSG00000169508.7 | *GPR183* | 14.8 | 2.37E-08 | 1.9 | 28.3 |
| ENSG00000181773.7 | *GPR3* | 4.9 | 2.01E-26 | 24.3 | 119.5 |
| ENSG00000100077.15 | *GRK3* | -3.2 | 2.46E-02 | 18.1 | 5.7 |
| ENSG00000164284.15 | *GRPEL2* | 1.8 | 7.79E-11 | 609.6 | 1087.9 |
| ENSG00000179240.11 | *GVQW3* | -1.9 | 2.90E-04 | 67.1 | 35.3 |
| ENSG00000138796.16 | *HADH* | -2.0 | 4.35E-03 | 51.6 | 25.4 |
| ENSG00000113070.8 | *HBEGF* | 5.4 | 2.17E-36 | 123.2 | 667.1 |
| ENSG00000105856.14 | *HBP1* | -2.1 | 3.69E-25 | 461.8 | 225.0 |
| ENSG00000165338.16 | *HECTD2* | 1.8 | 4.68E-02 | 28.9 | 51.7 |
| ENSG00000114315.4 | *HES1* | 20.3 | 4.35E-132 | 36.2 | 735.5 |
| ENSG00000177374.13 | *HIC1* | 2.8 | 3.18E-14 | 51.0 | 144.1 |
| ENSG00000233822.4 | *HIST1H2BN* | 4.7 | 3.36E-02 | 4.4 | 20.6 |
| ENSG00000270882.2 | *HIST2H4A* | -1.9 | 3.11E-02 | 75.0 | 40.4 |
| ENSG00000095951.17 | *HIVEP1* | 1.6 | 1.83E-05 | 379.7 | 610.6 |
| ENSG00000127124.16 | *HIVEP3* | 2.5 | 2.48E-05 | 41.2 | 101.3 |
| ENSG00000149948.13 | *HMGA2* | 2.8 | 6.13E-03 | 11.2 | 31.8 |
| ENSG00000112972.15 | *HMGCS1* | 1.6 | 7.49E-12 | 1218.7 | 1955.4 |
| ENSG00000188620.11 | *HMX3* | 1.7 | 1.76E-03 | 41.7 | 69.2 |
| ENSG00000152413.15 | *HOMER1* | 1.5 | 2.46E-02 | 121.3 | 185.2 |
| ENSG00000106031.9 | *HOXA13* | -2.0 | 2.80E-03 | 76.9 | 39.1 |
| ENSG00000196639.6 | *HRH1* | 3.2 | 1.37E-02 | 11.3 | 36.3 |
| ENSG00000284024.2 | *HSPA14* | 2.3 | 7.38E-08 | 82.8 | 188.7 |
| ENSG00000204389.10 | *HSPA1A* | -1.6 | 5.90E-13 | 3173.6 | 1942.8 |
| ENSG00000204388.7 | *HSPA1B* | -1.8 | 5.99E-13 | 2842.7 | 1618.8 |
| ENSG00000204390.10 | *HSPA1L* | -2.7 | 1.97E-04 | 23.8 | 8.8 |
| ENSG00000251348.1 | *HSPD1P11* | 3.4 | 1.03E-03 | 7.5 | 25.2 |
| ENSG00000090339.9 | *ICAM1* | 1.6 | 7.05E-08 | 168.8 | 265.2 |
| ENSG00000160888.7 | *IER2* | 4.2 | 2.22E-231 | 703.0 | 2970.8 |
| ENSG00000137331.12 | *IER3* | 13.3 | 0.00E+00 | 370.9 | 4921.2 |
| ENSG00000162783.11 | *IER5* | 1.6 | 3.11E-08 | 541.6 | 860.2 |
| ENSG00000188483.8 | *IER5L* | 2.2 | 1.04E-12 | 258.2 | 573.0 |
| ENSG00000169991.11 | *IFFO2* | 3.1 | 1.94E-18 | 368.4 | 1149.9 |
| ENSG00000159128.14 | *IFNGR2* | -1.9 | 4.35E-02 | 51.5 | 27.7 |
| ENSG00000146674.15 | *IGFBP3* | 2.5 | 1.29E-08 | 421.3 | 1051.1 |
| ENSG00000095752.7 | *IL11* | 21.1 | 3.66E-10 | 1.8 | 37.3 |
| ENSG00000163702.20 | *IL17RC* | -1.7 | 2.35E-02 | 101.4 | 57.9 |
| ENSG00000196083.10 | *IL1RAP* | 2.1 | 1.79E-16 | 133.4 | 279.6 |
| ENSG00000136244.12 | *IL6* | 4.9 | 2.87E-07 | 6.8 | 33.6 |
| ENSG00000122641.11 | *INHBA* | 7.4 | 1.42E-05 | 4.1 | 30.4 |
| ENSG00000186480.13 | *INSIG1* | 1.7 | 9.17E-10 | 1072.6 | 1853.3 |
| ENSG00000283154.2 | *IQCJ-SCHIP1* | 1.6 | 1.04E-04 | 188.6 | 294.7 |
| ENSG00000134070.5 | *IRAK2* | 2.1 | 5.40E-03 | 24.1 | 50.1 |
| ENSG00000125347.14 | *IRF1* | 1.8 | 1.69E-05 | 163.1 | 291.3 |
| ENSG00000168264.10 | *IRF2BP2* | 1.6 | 2.05E-08 | 303.2 | 479.3 |
| ENSG00000119669.5 | *IRF2BPL* | 2.9 | 3.98E-132 | 458.7 | 1313.1 |
| ENSG00000159556.10 | *ISL2* | 1.9 | 8.86E-04 | 33.7 | 63.6 |
| ENSG00000005884.18 | *ITGA3* | 1.6 | 2.82E-09 | 1780.3 | 2919.3 |
| ENSG00000161638.11 | *ITGA5* | 2.5 | 1.09E-60 | 1164.1 | 2916.8 |
| ENSG00000086544.3 | *ITPKC* | 1.7 | 2.27E-04 | 74.7 | 123.5 |
| ENSG00000148841.17 | *ITPRIP* | 3.2 | 1.50E-40 | 283.6 | 911.4 |
| ENSG00000101384.12 | *JAG1* | 1.6 | 5.74E-06 | 257.8 | 410.5 |
| ENSG00000177606.7 | *JUN* | 3.8 | 4.81E-57 | 302.9 | 1162.8 |
| ENSG00000171223.6 | *JUNB* | 12.6 | 0.00E+00 | 268.3 | 3371.8 |
| ENSG00000130522.6 | *JUND* | 1.6 | 2.30E-12 | 998.7 | 1622.3 |
| ENSG00000170852.11 | *KBTBD2* | 1.8 | 2.56E-35 | 674.2 | 1242.5 |
| ENSG00000165572.8 | *KBTBD6* | -1.6 | 1.00E-05 | 269.3 | 173.1 |
| ENSG00000120696.9 | *KBTBD7* | -2.2 | 1.71E-10 | 104.7 | 47.1 |
| ENSG00000184185.10 | *KCNJ12* | 2.8 | 1.03E-07 | 35.1 | 96.6 |
| ENSG00000121361.5 | *KCNJ8* | -2.0 | 3.01E-02 | 39.6 | 20.2 |
| ENSG00000164626.9 | *KCNK5* | 1.7 | 1.37E-06 | 101.0 | 171.0 |
| ENSG00000188997.8 | *KCTD21* | -2.0 | 1.18E-06 | 74.0 | 36.8 |
| ENSG00000107077.18 | *KDM4C* | 1.7 | 3.02E-02 | 33.3 | 56.7 |
| ENSG00000132510.10 | *KDM6B* | 13.8 | 9.24E-06 | 34.8 | 481.9 |
| ENSG00000150477.15 | *KIAA1328* | 2.1 | 2.14E-03 | 25.4 | 52.2 |
| ENSG00000054523.17 | *KIF1B* | 2.1 | 2.42E-02 | 143.1 | 302.9 |
| ENSG00000066735.14 | *KIF26A* | 3488435.9 | 4.47E-11 | 0.0 | 46.2 |
| ENSG00000155090.15 | *KLF10* | 8.3 | 3.74E-228 | 438.3 | 3657.9 |
| ENSG00000172059.11 | *KLF11* | 1.7 | 5.33E-12 | 273.9 | 467.9 |
| ENSG00000169926.11 | *KLF13* | 1.8 | 2.95E-18 | 374.0 | 679.4 |
| ENSG00000129911.9 | *KLF16* | 1.6 | 1.39E-10 | 642.2 | 1047.6 |
| ENSG00000109787.13 | *KLF3* | 1.6 | 2.51E-07 | 264.0 | 432.2 |
| ENSG00000136826.15 | *KLF4* | 2.8 | 1.70E-12 | 215.7 | 598.6 |
| ENSG00000102554.14 | *KLF5* | 1.6 | 3.49E-08 | 328.1 | 524.1 |
| ENSG00000067082.15 | *KLF6* | 2.0 | 6.80E-13 | 1304.7 | 2654.4 |
| ENSG00000118263.15 | *KLF7* | 1.7 | 1.30E-06 | 319.9 | 555.7 |
| ENSG00000167487.12 | *KLHL26* | 1.7 | 8.49E-07 | 139.1 | 242.3 |
| ENSG00000133247.14 | *KMT5C* | -1.7 | 5.88E-03 | 66.9 | 39.8 |
| ENSG00000212724.3 | *KRTAP2-3* | 6.1 | 3.70E-30 | 21.1 | 129.3 |
| ENSG00000174720.16 | *LARP7* | -2.2 | 1.81E-02 | 154.1 | 69.1 |
| ENSG00000150457.9 | *LATS2* | 1.7 | 2.02E-15 | 294.8 | 492.7 |
| ENSG00000213626.13 | *LBH* | 3.5 | 3.87E-19 | 102.8 | 364.4 |
| ENSG00000130164.13 | *LDLR* | 6.9 | 1.71E-04 | 31.2 | 214.4 |
| ENSG00000167615.16 | *LENG8* | 1.7 | 4.05E-06 | 398.3 | 668.5 |
| ENSG00000106003.13 | *LFNG* | 5.2 | 5.98E-60 | 84.6 | 439.1 |
| ENSG00000128342.5 | *LIF* | 13.7 | 2.68E-157 | 135.5 | 1860.3 |
| ENSG00000267796.8 | *LIN37* | -1.6 | 3.38E-02 | 63.2 | 38.4 |
| ENSG00000233237.8 | *LINC00472* | 1.9 | 6.52E-06 | 99.4 | 189.8 |
| ENSG00000175611.12 | *LINC00476* | -1.5 | 2.93E-03 | 97.2 | 62.9 |
| ENSG00000245060.8 | *LINC00847* | -1.8 | 1.15E-03 | 61.7 | 34.8 |
| ENSG00000228794.9 | *LINC01128* | 1.6 | 1.17E-02 | 127.2 | 204.0 |
| ENSG00000272419.6 | *LINC01145* | -4.9 | 3.61E-03 | 118.3 | 24.3 |
| ENSG00000261326.4 | *LINC01355* | -2.3 | 2.10E-04 | 64.7 | 27.7 |
| ENSG00000226053.1 | *LINC01776* | 2.7 | 4.46E-07 | 20.6 | 55.4 |
| ENSG00000204362.6 | *LINC02783* | 3.1 | 1.02E-02 | 7.2 | 22.1 |
| ENSG00000198589.13 | *LRBA* | 1.6 | 2.72E-03 | 314.9 | 508.4 |
| ENSG00000248323.7 | *LUCAT1* | 2.6 | 3.15E-02 | 6.4 | 16.9 |
| ENSG00000169641.13 | *LUZP1* | 2.1 | 1.80E-03 | 37.7 | 78.3 |
| ENSG00000185022.12 | *MAFF* | 7.7 | 2.33E-153 | 84.6 | 651.2 |
| ENSG00000198517.10 | *MAFK* | 2.3 | 5.28E-66 | 561.9 | 1305.3 |
| ENSG00000112893.9 | *MAN2A1* | 3.2 | 1.47E-02 | 11.4 | 36.8 |
| ENSG00000078018.19 | *MAP2* | 2.1 | 1.23E-02 | 44.3 | 93.5 |
| ENSG00000034152.19 | *MAP2K3* | 2.5 | 2.32E-33 | 633.6 | 1559.9 |
| ENSG00000095015.6 | *MAP3K1* | 1.5 | 1.09E-03 | 525.3 | 801.1 |
| ENSG00000006062.17 | *MAP3K14* | 3.0 | 1.61E-78 | 504.7 | 1493.9 |
| ENSG00000006432.15 | *MAP3K9* | 3.1 | 2.90E-02 | 5.6 | 17.3 |
| ENSG00000069956.12 | *MAPK6* | 1.5 | 2.33E-10 | 789.4 | 1198.8 |
| ENSG00000162889.10 | *MAPKAPK2* | 2.1 | 1.35E-02 | 27.0 | 57.1 |
| ENSG00000180611.7 | *MB21D2* | 3.1 | 3.32E-68 | 277.7 | 856.1 |
| ENSG00000204406.13 | *MBD5* | 2.0 | 8.16E-03 | 33.6 | 67.4 |
| ENSG00000229619.4 | *MBNL1-AS1* | 2.0 | 1.10E-04 | 94.4 | 187.5 |
| ENSG00000143384.13 | *MCL1* | 2.6 | 3.00E-88 | 3086.2 | 7948.8 |
| ENSG00000065328.16 | *MCM10* | 1.5 | 1.54E-02 | 268.2 | 411.2 |
| ENSG00000198625.13 | *MDM4* | 1.6 | 3.85E-06 | 327.5 | 528.2 |
| ENSG00000116604.18 | *MEF2D* | 1.6 | 1.79E-14 | 447.8 | 718.3 |
| ENSG00000168389.17 | *MFSD2A* | 4.8 | 1.41E-03 | 6.4 | 30.8 |
| ENSG00000174197.16 | *MGA* | 13.9 | 8.08E-09 | 3.6 | 49.6 |
| ENSG00000148343.18 | *MIGA2* | -1.9 | 2.07E-02 | 139.2 | 71.7 |
| ENSG00000204682.8 | *MIR1915HG* | -1.6 | 3.29E-03 | 96.8 | 62.4 |
| ENSG00000223749.10 | *MIR503HG* | 1.7 | 9.15E-05 | 66.7 | 114.2 |
| ENSG00000149968.12 | *MMP3* | 2.8 | 1.40E-03 | 11.4 | 32.2 |
| ENSG00000070444.15 | *MNT* | 1.7 | 7.91E-19 | 311.8 | 542.6 |
| ENSG00000130675.15 | *MNX1* | -2.0 | 3.54E-03 | 45.4 | 22.3 |
| ENSG00000165943.5 | *MOAP1* | 1.5 | 2.26E-10 | 229.1 | 347.4 |
| ENSG00000142961.14 | *MOB3C* | 2.4 | 7.76E-03 | 15.2 | 36.9 |
| ENSG00000185716.12 | *MOSMO* | 1.6 | 6.08E-05 | 106.4 | 172.9 |
| ENSG00000278619.5 | *MRM1* | -1.8 | 3.11E-08 | 119.0 | 66.1 |
| ENSG00000204839.9 | *MROH6* | -2.7 | 8.47E-04 | 26.1 | 9.5 |
| ENSG00000066697.14 | *MSANTD3* | 1.5 | 8.67E-03 | 269.0 | 410.5 |
| ENSG00000116062.15 | *MSH6* | 6.9 | 5.26E-08 | 6.6 | 45.4 |
| ENSG00000188786.10 | *MTF1* | 1.6 | 1.14E-03 | 304.9 | 500.5 |
| ENSG00000172732.12 | *MUS81* | 1.5 | 7.02E-07 | 314.9 | 486.2 |
| ENSG00000179820.16 | *MYADM* | 3.0 | 1.62E-37 | 914.2 | 2707.5 |
| ENSG00000172927.8 | *MYEOV* | 1.7 | 3.59E-02 | 30.9 | 52.3 |
| ENSG00000007944.15 | *MYLIP* | -2.0 | 1.43E-06 | 72.7 | 36.6 |
| ENSG00000099331.13 | *MYO9B* | 1.6 | 1.64E-11 | 529.0 | 826.1 |
| ENSG00000078177.14 | *N4BP2* | 1.9 | 2.01E-02 | 92.7 | 178.0 |
| ENSG00000145911.6 | *N4BP3* | 2.9 | 4.46E-04 | 13.3 | 38.8 |
| ENSG00000138386.17 | *NAB1* | 1.9 | 1.17E-12 | 247.6 | 463.3 |
| ENSG00000166886.13 | *NAB2* | 7.4 | 0.00E+00 | 245.2 | 1807.5 |
| ENSG00000161048.11 | *NAPEPLD* | -2.3 | 1.48E-05 | 60.9 | 26.0 |
| ENSG00000144959.10 | *NCEH1* | 1.7 | 4.00E-02 | 140.7 | 235.1 |
| ENSG00000115053.16 | *NCL* | 6.4 | 2.29E-02 | 19.7 | 125.3 |
| ENSG00000266412.5 | *NCOA4* | -1.5 | 6.78E-03 | 469.8 | 308.0 |
| ENSG00000166579.15 | *NDEL1* | 1.7 | 1.01E-08 | 186.4 | 311.1 |
| ENSG00000111859.17 | *NEDD9* | 4.2 | 4.51E-82 | 353.3 | 1469.6 |
| ENSG00000101096.20 | *NFATC2* | 2.5 | 1.66E-02 | 7.6 | 18.6 |
| ENSG00000001167.14 | *NFYA* | 1.7 | 1.71E-03 | 91.2 | 156.4 |
| ENSG00000163293.12 | *NIPAL1* | 2.6 | 4.40E-09 | 29.2 | 76.5 |
| ENSG00000167034.10 | *NKX3-1* | 1.6 | 4.22E-03 | 54.4 | 86.6 |
| ENSG00000011052.21 | *NME1-NME2* | -1.5 | 9.69E-05 | 207.0 | 135.0 |
| ENSG00000151014.6 | *NOCT* | 2.3 | 1.15E-33 | 162.1 | 373.1 |
| ENSG00000249673.7 | *NOP14-AS1* | 1.8 | 1.90E-04 | 54.8 | 99.1 |
| ENSG00000141458.13 | *NPC1* | 1.5 | 1.90E-10 | 418.5 | 635.1 |
| ENSG00000281183.1 | *NPTN-IT1* | 2.2 | 6.55E-03 | 14.7 | 32.6 |
| ENSG00000123358.20 | *NR4A1* | 7.1 | 9.82E-38 | 121.5 | 865.2 |
| ENSG00000153234.14 | *NR4A2* | 3.0 | 1.48E-03 | 10.7 | 31.9 |
| ENSG00000119508.18 | *NR4A3* | 10.1 | 4.46E-06 | 3.2 | 32.6 |
| ENSG00000180530.11 | *NRIP1* | 1.7 | 1.99E-04 | 278.5 | 487.3 |
| ENSG00000111696.12 | *NT5DC3* | 2.2 | 2.54E-29 | 256.0 | 553.0 |
| ENSG00000163545.10 | *NUAK2* | 5.5 | 9.59E-28 | 55.9 | 309.9 |
| ENSG00000108256.9 | *NUFIP2* | 1.6 | 3.07E-06 | 1230.0 | 1944.6 |
| ENSG00000235636.1 | *NUS1P1* | 2.5 | 1.79E-13 | 75.3 | 186.5 |
| ENSG00000144227.5 | *NXPH2* | -2.1 | 9.40E-08 | 113.4 | 54.9 |
| ENSG00000164920.9 | *OSR2* | -3.1 | 1.41E-05 | 68.1 | 22.0 |
| ENSG00000142623.11 | *PADI1* | 3.4 | 4.34E-03 | 9.5 | 32.4 |
| ENSG00000110218.9 | *PANX1* | 1.6 | 4.86E-11 | 361.9 | 565.8 |
| ENSG00000239883.8 | *PARGP1* | 1.9 | 4.45E-02 | 13.9 | 26.8 |
| ENSG00000162396.6 | *PARS2* | -2.1 | 1.08E-05 | 54.2 | 26.2 |
| ENSG00000132849.20 | *PATJ* | 1.7 | 3.21E-04 | 173.9 | 299.7 |
| ENSG00000189184.11 | *PCDH18* | -1.8 | 5.59E-03 | 221.5 | 125.5 |
| ENSG00000169851.15 | *PCDH7* | 4.4 | 1.57E-33 | 78.1 | 347.2 |
| ENSG00000197646.8 | *PDCD1LG2* | 2.0 | 9.60E-03 | 14.6 | 28.9 |
| ENSG00000100311.17 | *PDGFB* | 4.7 | 2.01E-21 | 83.7 | 391.9 |
| ENSG00000004799.8 | *PDK4* | -1.9 | 4.73E-04 | 71.2 | 36.9 |
| ENSG00000163110.15 | *PDLIM5* | 1.7 | 4.61E-09 | 793.2 | 1353.4 |
| ENSG00000164951.16 | *PDP1* | 2.5 | 1.06E-42 | 238.5 | 598.7 |
| ENSG00000197329.12 | *PELI1* | 1.9 | 1.52E-05 | 82.2 | 154.2 |
| ENSG00000179094.16 | *PER1* | 3.2 | 1.02E-15 | 89.3 | 283.2 |
| ENSG00000108733.10 | *PEX12* | -1.6 | 3.51E-04 | 83.0 | 50.5 |
| ENSG00000112357.13 | *PEX7* | -1.8 | 3.29E-02 | 32.8 | 18.7 |
| ENSG00000170525.21 | *PFKFB3* | 1.9 | 8.32E-29 | 933.3 | 1806.7 |
| ENSG00000134686.18 | *PHC2* | 1.8 | 4.94E-17 | 930.2 | 1642.2 |
| ENSG00000109118.14 | *PHF12* | -1.7 | 2.24E-07 | 433.3 | 261.8 |
| ENSG00000116273.6 | *PHF13* | 1.7 | 8.64E-14 | 445.2 | 747.2 |
| ENSG00000139289.13 | *PHLDA1* | 8.5 | 1.81E-121 | 235.0 | 2004.4 |
| ENSG00000181649.8 | *PHLDA2* | 1.7 | 2.26E-09 | 532.4 | 885.5 |
| ENSG00000144824.21 | *PHLDB2* | 2.3 | 2.82E-27 | 703.8 | 1635.0 |
| ENSG00000140451.13 | *PIF1* | -1.8 | 1.70E-08 | 285.7 | 159.6 |
| ENSG00000137193.14 | *PIM1* | 1.7 | 8.30E-17 | 777.8 | 1350.8 |
| ENSG00000198355.5 | *PIM3* | 1.6 | 7.39E-11 | 670.9 | 1094.4 |
| ENSG00000241878.11 | *PISD* | 2.0 | 9.02E-07 | 109.4 | 216.5 |
| ENSG00000164093.17 | *PITX2* | -2.3 | 5.18E-07 | 77.2 | 33.6 |
| ENSG00000122861.16 | *PLAU* | 3.7 | 4.80E-67 | 267.2 | 993.0 |
| ENSG00000011422.12 | *PLAUR* | 2.5 | 9.26E-13 | 171.3 | 420.0 |
| ENSG00000179598.6 | *PLD6* | 2.0 | 3.12E-13 | 86.7 | 169.6 |
| ENSG00000178209.15 | *PLEC* | 1.6 | 6.87E-12 | 4281.8 | 6872.8 |
| ENSG00000105559.12 | *PLEKHA4* | 1.6 | 3.37E-02 | 45.3 | 74.1 |
| ENSG00000090924.15 | *PLEKHG2* | 2.6 | 3.38E-03 | 101.9 | 263.8 |
| ENSG00000068137.15 | *PLEKHH3* | 1.6 | 2.20E-08 | 187.8 | 294.0 |
| ENSG00000241839.10 | *PLEKHO2* | 1.6 | 1.93E-07 | 128.5 | 209.4 |
| ENSG00000173846.13 | *PLK3* | 5.8 | 9.86E-115 | 88.6 | 515.5 |
| ENSG00000124225.16 | *PMEPA1* | 1.9 | 1.01E-19 | 435.3 | 814.7 |
| ENSG00000064933.17 | *PMS1* | -1.8 | 1.68E-02 | 89.8 | 49.5 |
| ENSG00000146278.11 | *PNRC1* | -2.6 | 2.87E-30 | 359.7 | 140.2 |
| ENSG00000186866.16 | *POFUT2* | 2.0 | 1.23E-03 | 79.1 | 155.8 |
| ENSG00000196313.11 | *POM121* | 1.8 | 2.22E-04 | 39.6 | 70.5 |
| ENSG00000087074.8 | *PPP1R15A* | 2.3 | 1.43E-15 | 678.7 | 1553.9 |
| ENSG00000146112.12 | *PPP1R18* | 1.6 | 9.70E-14 | 811.2 | 1324.1 |
| ENSG00000173281.5 | *PPP1R3B* | 3.0 | 1.24E-09 | 29.2 | 87.5 |
| ENSG00000119938.9 | *PPP1R3C* | -1.6 | 1.93E-04 | 81.3 | 50.1 |
| ENSG00000275342.5 | *PRAG1* | 4.2 | 2.48E-37 | 103.9 | 431.6 |
| ENSG00000106617.14 | *PRKAG2* | 1.6 | 1.13E-06 | 143.0 | 228.4 |
| ENSG00000228672.4 | *PROB1* | 1.6 | 4.33E-06 | 89.6 | 145.8 |
| ENSG00000171522.6 | *PTGER4* | 2.0 | 2.36E-07 | 54.1 | 108.5 |
| ENSG00000122420.10 | *PTGFR* | -1.7 | 1.43E-08 | 155.7 | 93.7 |
| ENSG00000073756.12 | *PTGS2* | 31.4 | 3.06E-13 | 1.5 | 47.9 |
| ENSG00000127329.15 | *PTPRB* | 1.8 | 5.95E-05 | 142.6 | 255.7 |
| ENSG00000055917.15 | *PUM2* | -1.6 | 2.45E-02 | 603.6 | 388.3 |
| ENSG00000146676.10 | *PURB* | 1.8 | 1.63E-29 | 1259.1 | 2249.1 |
| ENSG00000168994.13 | *PXDC1* | 2.1 | 1.72E-06 | 201.0 | 413.7 |
| ENSG00000204764.14 | *RANBP17* | 1.9 | 4.19E-02 | 29.0 | 53.6 |
| ENSG00000109756.9 | *RAPGEF2* | 2.2 | 5.54E-05 | 200.3 | 439.5 |
| ENSG00000173166.18 | *RAPH1* | 1.6 | 3.52E-03 | 407.9 | 670.6 |
| ENSG00000131759.18 | *RARA* | 2.7 | 1.20E-49 | 231.8 | 621.8 |
| ENSG00000198774.5 | *RASSF9* | -2.3 | 9.36E-06 | 48.3 | 20.9 |
| ENSG00000146587.18 | *RBAK* | 1.6 | 2.09E-03 | 248.1 | 408.5 |
| ENSG00000104856.14 | *RELB* | 2.2 | 2.37E-08 | 82.5 | 179.8 |
| ENSG00000181826.10 | *RELL1* | 1.7 | 1.13E-05 | 66.8 | 116.6 |
| ENSG00000054967.13 | *RELT* | 2.1 | 1.10E-31 | 224.3 | 471.1 |
| ENSG00000182175.14 | *RGMA* | -2.4 | 4.21E-02 | 18.1 | 7.7 |
| ENSG00000143333.7 | *RGS16* | 2.4 | 5.31E-06 | 45.2 | 110.3 |
| ENSG00000116741.8 | *RGS2* | 3.0 | 1.70E-15 | 71.7 | 212.1 |
| ENSG00000138835.22 | *RGS3* | 2.0 | 1.02E-14 | 157.3 | 318.6 |
| ENSG00000117152.13 | *RGS4* | 7.9 | 1.56E-02 | 3.8 | 30.4 |
| ENSG00000143878.10 | *RHOB* | 2.4 | 1.44E-45 | 567.6 | 1342.7 |
| ENSG00000104889.6 | *RNASEH2A* | -1.6 | 7.18E-04 | 72.4 | 44.1 |
| ENSG00000115963.13 | *RND3* | 1.8 | 5.72E-03 | 311.8 | 556.9 |
| ENSG00000145860.11 | *RNF145* | -3.2 | 2.74E-02 | 298.4 | 94.3 |
| ENSG00000118518.15 | *RNF146* | -1.7 | 2.63E-03 | 140.5 | 84.4 |
| ENSG00000139266.6 | *RNF179* | -1.7 | 5.53E-03 | 57.4 | 34.0 |
| ENSG00000146373.16 | *RNF217* | 2.4 | 3.13E-05 | 60.1 | 142.4 |
| ENSG00000223396.4 | *RPS10P7* | 2.5 | 1.67E-02 | 8.1 | 20.0 |
| ENSG00000166592.12 | *RRAD* | 3.0 | 1.89E-04 | 8.5 | 25.5 |
| ENSG00000081019.13 | *RSBN1* | -1.7 | 2.10E-07 | 251.8 | 147.2 |
| ENSG00000215012.9 | *RTL10* | 1.7 | 1.12E-11 | 334.1 | 561.2 |
| ENSG00000159216.18 | *RUNX1* | 2.0 | 4.41E-15 | 471.7 | 933.1 |
| ENSG00000198853.12 | *RUSC2* | 1.7 | 1.41E-29 | 751.4 | 1284.5 |
| ENSG00000204231.10 | *RXRB* | -1.6 | 3.82E-04 | 371.1 | 231.6 |
| ENSG00000267534.4 | *S1PR2* | 1.9 | 2.35E-06 | 41.9 | 81.3 |
| ENSG00000180739.14 | *S1PR5* | -2.3 | 5.29E-03 | 27.1 | 11.7 |
| ENSG00000211456.12 | *SACM1L* | -1.6 | 3.32E-02 | 492.4 | 305.4 |
| ENSG00000101115.13 | *SALL4* | 3.0 | 4.06E-02 | 8.7 | 25.8 |
| ENSG00000187634.12 | *SAMD11* | 2.2 | 3.36E-04 | 65.2 | 142.0 |
| ENSG00000130066.16 | *SAT1* | 3.4 | 7.79E-17 | 45.0 | 150.9 |
| ENSG00000213079.9 | *SCAF8* | 288.4 | 3.67E-14 | 0.2 | 66.9 |
| ENSG00000124145.6 | *SDC4* | 2.2 | 7.81E-62 | 1285.5 | 2818.6 |
| ENSG00000107651.13 | *SEC23IP* | 2.2 | 3.96E-02 | 23.8 | 53.3 |
| ENSG00000138623.10 | *SEMA7A* | 2.8 | 8.54E-40 | 275.9 | 781.4 |
| ENSG00000119231.11 | *SENP5* | 1.6 | 9.69E-08 | 495.6 | 786.3 |
| ENSG00000166401.15 | *SERPINB8* | 1.7 | 1.03E-05 | 111.1 | 183.7 |
| ENSG00000197019.5 | *SERTAD1* | 4.5 | 1.38E-78 | 101.2 | 453.2 |
| ENSG00000179833.4 | *SERTAD2* | 2.0 | 1.65E-18 | 411.2 | 825.5 |
| ENSG00000082497.12 | *SERTAD4* | 1.6 | 1.78E-05 | 290.0 | 469.0 |
| ENSG00000080546.13 | *SESN1* | -1.7 | 2.60E-04 | 92.6 | 53.7 |
| ENSG00000130766.5 | *SESN2* | 2.5 | 2.14E-04 | 215.7 | 532.8 |
| ENSG00000139718.10 | *SETD1B* | 164.1 | 1.46E-02 | 0.2 | 38.0 |
| ENSG00000168137.16 | *SETD5* | 1.6 | 2.96E-04 | 659.2 | 1041.1 |
| ENSG00000145391.13 | *SETD7* | 1.7 | 2.22E-02 | 30.4 | 51.3 |
| ENSG00000107290.14 | *SETX* | 1.6 | 4.68E-03 | 900.6 | 1443.3 |
| ENSG00000118515.11 | *SGK1* | 3.4 | 3.28E-28 | 138.3 | 467.9 |
| ENSG00000154447.15 | *SH3RF1* | 2.2 | 1.46E-10 | 177.2 | 391.6 |
| ENSG00000107338.10 | *SHB* | 1.7 | 1.51E-18 | 282.7 | 491.5 |
| ENSG00000142178.9 | *SIK1* | 2.3 | 2.16E-15 | 179.9 | 411.9 |
| ENSG00000180592.17 | *SKIDA1* | -5.5 | 7.20E-10 | 35.9 | 6.5 |
| ENSG00000136603.14 | *SKIL* | 2.9 | 1.22E-27 | 494.0 | 1435.7 |
| ENSG00000141526.16 | *SLC16A3* | 1.7 | 9.47E-04 | 279.6 | 466.3 |
| ENSG00000144136.11 | *SLC20A1* | 2.0 | 1.68E-40 | 1038.4 | 2028.4 |
| ENSG00000163393.13 | *SLC22A15* | 3.2 | 8.99E-03 | 7.4 | 23.8 |
| ENSG00000148339.12 | *SLC25A25* | 2.2 | 2.11E-25 | 337.7 | 743.1 |
| ENSG00000117394.23 | *SLC2A1* | 1.5 | 1.36E-09 | 475.0 | 724.4 |
| ENSG00000059804.16 | *SLC2A3* | 2.8 | 2.60E-15 | 49.8 | 138.1 |
| ENSG00000142583.18 | *SLC2A5* | 3.4 | 3.63E-03 | 10.1 | 34.5 |
| ENSG00000170385.10 | *SLC30A1* | 2.8 | 7.88E-71 | 471.5 | 1316.2 |
| ENSG00000215790.7 | *SLC35E2A* | -1.7 | 1.68E-02 | 46.6 | 28.2 |
| ENSG00000022567.9 | *SLC45A4* | 4.7 | 1.26E-06 | 21.3 | 100.0 |
| ENSG00000154760.14 | *SLFN13* | 2.2 | 7.33E-03 | 48.8 | 106.1 |
| ENSG00000166750.10 | *SLFN5* | 1.6 | 1.22E-08 | 159.4 | 256.2 |
| ENSG00000281207.1 | *SLFNL1-AS1* | 2.2 | 4.93E-04 | 15.6 | 35.1 |
| ENSG00000165300.7 | *SLITRK5* | -1.8 | 8.27E-03 | 161.6 | 90.5 |
| ENSG00000101665.9 | *SMAD7* | 2.5 | 1.05E-05 | 63.7 | 159.2 |
| ENSG00000261556.10 | *SMG1P7* | -2.2 | 3.27E-04 | 57.1 | 25.5 |
| ENSG00000088826.18 | *SMOX* | 1.7 | 2.50E-03 | 50.9 | 88.7 |
| ENSG00000198742.9 | *SMURF1* | 2.5 | 4.52E-02 | 6.9 | 17.2 |
| ENSG00000124216.4 | *SNAI1* | 5.2 | 2.24E-67 | 71.8 | 370.6 |
| ENSG00000019549.13 | *SNAI2* | 1.7 | 8.41E-09 | 110.6 | 186.4 |
| ENSG00000178996.14 | *SNX18* | 2.5 | 5.75E-26 | 202.0 | 495.0 |
| ENSG00000184557.4 | *SOCS3* | 1.5 | 2.00E-04 | 81.5 | 126.0 |
| ENSG00000170677.6 | *SOCS6* | 1.6 | 1.64E-06 | 170.6 | 269.8 |
| ENSG00000142168.14 | *SOD1* | -2.9 | 3.33E-03 | 16.4 | 5.7 |
| ENSG00000198142.5 | *SOWAHC* | 4.2 | 4.24E-60 | 338.0 | 1417.4 |
| ENSG00000125398.8 | *SOX9* | 4.3 | 3.17E-18 | 22.3 | 95.9 |
| ENSG00000158792.16 | *SPATA2L* | 2.9 | 1.09E-11 | 51.6 | 148.0 |
| ENSG00000176170.13 | *SPHK1* | 1.8 | 2.14E-04 | 222.3 | 407.0 |
| ENSG00000134668.12 | *SPOCD1* | 2.8 | 1.79E-13 | 75.3 | 213.5 |
| ENSG00000166068.13 | *SPRED1* | 1.6 | 3.19E-09 | 333.1 | 542.2 |
| ENSG00000198369.10 | *SPRED2* | 1.8 | 1.29E-11 | 242.9 | 437.0 |
| ENSG00000136158.12 | *SPRY2* | 4.8 | 1.44E-45 | 38.1 | 181.6 |
| ENSG00000187678.9 | *SPRY4* | 257.3 | 5.40E-14 | 0.2 | 59.7 |
| ENSG00000137877.10 | *SPTBN5* | 2.1 | 6.68E-03 | 17.8 | 36.6 |
| ENSG00000172296.13 | *SPTLC3* | 2.7 | 3.78E-02 | 7.4 | 19.7 |
| ENSG00000112658.8 | *SRF* | 1.9 | 1.96E-50 | 1428.3 | 2706.0 |
| ENSG00000146700.9 | *SSC4D* | 2.3 | 1.58E-02 | 10.8 | 25.3 |
| ENSG00000084112.15 | *SSH1* | 1.5 | 1.14E-05 | 659.0 | 1013.3 |
| ENSG00000008513.16 | *ST3GAL1* | 1.6 | 2.81E-03 | 190.5 | 296.0 |
| ENSG00000115525.18 | *ST3GAL5* | 2.1 | 1.16E-04 | 33.7 | 71.0 |
| ENSG00000227199.1 | *ST7-AS1* | 1.8 | 3.41E-02 | 22.7 | 40.1 |
| ENSG00000101972.18 | *STAG2* | 1.5 | 7.21E-04 | 746.5 | 1140.3 |
| ENSG00000164211.13 | *STARD4* | 1.9 | 4.51E-13 | 330.7 | 624.4 |
| ENSG00000159167.12 | *STC1* | 2.0 | 2.33E-02 | 13.2 | 26.0 |
| ENSG00000113739.10 | *STC2* | 2.7 | 4.22E-05 | 63.9 | 172.2 |
| ENSG00000211455.8 | *STK38L* | 1.8 | 4.41E-15 | 285.6 | 506.8 |
| ENSG00000137868.19 | *STRA6* | 1.9 | 1.29E-02 | 68.2 | 131.1 |
| ENSG00000106089.12 | *STX1A* | 1.7 | 1.87E-02 | 64.9 | 111.5 |
| ENSG00000116668.13 | *SWT1* | -2.1 | 3.26E-04 | 46.7 | 22.1 |
| ENSG00000097096.9 | *SYDE2* | 2.2 | 3.76E-05 | 60.5 | 134.0 |
| ENSG00000078269.15 | *SYNJ2* | 1.6 | 4.47E-11 | 430.7 | 695.6 |
| ENSG00000173227.14 | *SYT12* | 1.6 | 3.48E-03 | 66.4 | 108.3 |
| ENSG00000135801.9 | *TAF5L* | 2.5 | 6.08E-20 | 131.7 | 335.3 |
| ENSG00000149591.17 | *TAGLN* | 2.0 | 6.94E-11 | 481.9 | 983.3 |
| ENSG00000274512.5 | *TBC1D3L* | -1.7 | 1.45E-02 | 31.5 | 18.5 |
| ENSG00000081059.20 | *TCF7* | 2.1 | 4.95E-04 | 69.7 | 144.0 |
| ENSG00000112773.16 | *TENT5A* | -1.7 | 7.94E-17 | 363.3 | 211.2 |
| ENSG00000173451.7 | *THAP2* | -1.6 | 4.52E-02 | 43.0 | 27.3 |
| ENSG00000041988.15 | *THAP3* | -1.7 | 4.71E-02 | 67.0 | 39.5 |
| ENSG00000137801.11 | *THBS1* | 3.4 | 5.33E-74 | 7997.6 | 27470.3 |
| ENSG00000140534.14 | *TICRR* | -1.7 | 7.13E-10 | 672.1 | 394.0 |
| ENSG00000204152.11 | *TIMM23B* | 1.6 | 3.12E-02 | 63.4 | 99.5 |
| ENSG00000163659.13 | *TIPARP* | 2.2 | 2.01E-32 | 1254.9 | 2821.3 |
| ENSG00000140406.4 | *TLNRD1* | 1.6 | 1.37E-16 | 334.3 | 547.8 |
| ENSG00000149809.15 | *TM7SF2* | -2.9 | 1.37E-03 | 19.7 | 6.9 |
| ENSG00000006118.14 | *TMEM132A* | 2.0 | 2.96E-04 | 55.8 | 113.8 |
| ENSG00000172738.12 | *TMEM217* | 3.3 | 1.13E-05 | 9.5 | 31.0 |
| ENSG00000070269.14 | *TMEM260* | -1.5 | 3.40E-02 | 115.3 | 75.0 |
| ENSG00000157693.15 | *TMEM268* | 1.6 | 1.79E-11 | 219.9 | 355.4 |
| ENSG00000166471.11 | *TMEM41B* | 1.5 | 3.87E-08 | 436.7 | 670.0 |
| ENSG00000104689.10 | *TNFRSF10A* | 2.1 | 1.30E-16 | 156.8 | 332.6 |
| ENSG00000120889.13 | *TNFRSF10B* | 1.5 | 1.31E-08 | 888.6 | 1352.5 |
| ENSG00000006327.13 | *TNFRSF12A* | 2.0 | 1.29E-07 | 163.7 | 319.5 |
| ENSG00000181634.8 | *TNFSF15* | 8.6 | 6.21E-05 | 2.4 | 20.9 |
| ENSG00000125657.5 | *TNFSF9* | 1.7 | 1.31E-05 | 79.1 | 134.8 |
| ENSG00000241015.2 | *TPM3P9* | 1.9 | 6.39E-05 | 41.5 | 78.4 |
| ENSG00000076604.15 | *TRAF4* | 2.0 | 3.45E-37 | 495.5 | 1007.2 |
| ENSG00000173334.4 | *TRIB1* | 2.2 | 1.81E-12 | 133.7 | 299.4 |
| ENSG00000154370.16 | *TRIM11* | 1.5 | 1.02E-10 | 466.1 | 714.7 |
| ENSG00000204977.10 | *TRIM13* | 2.1 | 3.90E-16 | 96.7 | 203.0 |
| ENSG00000157514.16 | *TSC22D3* | 2.1 | 1.30E-06 | 77.0 | 158.9 |
| ENSG00000121297.8 | *TSHZ3* | 1.7 | 4.23E-02 | 40.4 | 68.6 |
| ENSG00000143367.16 | *TUFT1* | 2.6 | 3.48E-122 | 976.4 | 2522.4 |
| ENSG00000265972.6 | *TXNIP* | -1.7 | 3.07E-06 | 581.1 | 334.3 |
| ENSG00000117143.13 | *UAP1* | 2.6 | 1.74E-04 | 14.7 | 38.6 |
| ENSG00000183696.14 | *UPP1* | 1.6 | 4.27E-06 | 225.0 | 360.4 |
| ENSG00000273820.2 | *USP27X* | 2.1 | 7.73E-04 | 24.2 | 51.0 |
| ENSG00000055483.19 | *USP36* | 2.2 | 3.67E-06 | 64.6 | 139.0 |
| ENSG00000139190.16 | *VAMP1* | 1.8 | 3.55E-02 | 21.6 | 38.1 |
| ENSG00000168140.5 | *VASN* | 2.9 | 3.32E-87 | 782.4 | 2233.1 |
| ENSG00000125753.14 | *VASP* | 1.6 | 4.21E-09 | 1013.6 | 1659.9 |
| ENSG00000035403.17 | *VCL* | 1.6 | 4.53E-10 | 1482.5 | 2320.2 |
| ENSG00000112715.22 | *VEGFA* | 1.9 | 4.15E-09 | 506.5 | 977.9 |
| ENSG00000128564.7 | *VGF* | 3.0 | 3.02E-03 | 6.1 | 18.1 |
| ENSG00000206538.9 | *VGLL3* | 1.9 | 7.67E-07 | 406.6 | 779.2 |
| ENSG00000062716.13 | *VMP1* | 1.7 | 5.41E-16 | 2058.6 | 3565.0 |
| ENSG00000139722.7 | *VPS37B* | 1.7 | 8.50E-26 | 373.7 | 643.7 |
| ENSG00000163026.12 | *WDCP* | -1.7 | 8.37E-04 | 332.1 | 193.0 |
| ENSG00000133316.15 | *WDR74* | 1.7 | 6.81E-06 | 92.6 | 157.4 |
| ENSG00000167716.18 | *WDR81* | -2.2 | 3.73E-02 | 35.1 | 16.2 |
| ENSG00000198373.12 | *WWP2* | 1.8 | 9.19E-03 | 59.0 | 105.2 |
| ENSG00000134684.10 | *YARS* | 2.0 | 3.47E-04 | 74.6 | 148.7 |
| ENSG00000180667.10 | *YOD1* | -10148955.9 | 7.55E-13 | 45.5 | 0.0 |
| ENSG00000196449.4 | *YRDC* | 1.8 | 1.14E-18 | 424.8 | 773.3 |
| ENSG00000221886.4 | *ZBED8* | -2.6 | 5.46E-10 | 71.3 | 27.7 |
| ENSG00000126804.13 | *ZBTB1* | 2.0 | 1.16E-02 | 112.7 | 229.4 |
| ENSG00000173276.14 | *ZBTB21* | 1.9 | 2.94E-06 | 354.9 | 669.6 |
| ENSG00000163874.11 | *ZC3H12A* | 3.3 | 3.96E-31 | 98.4 | 323.8 |
| ENSG00000149289.11 | *ZC3H12C* | 1.9 | 3.91E-05 | 161.2 | 308.1 |
| ENSG00000133858.16 | *ZFC3H1* | 1.9 | 1.29E-02 | 86.5 | 162.6 |
| ENSG00000128016.7 | *ZFP36* | 3.6 | 2.80E-25 | 73.0 | 260.8 |
| ENSG00000185650.9 | *ZFP36L1* | 11.6 | 1.36E-06 | 6.1 | 70.6 |
| ENSG00000152518.8 | *ZFP36L2* | 3.0 | 2.77E-92 | 557.5 | 1674.6 |
| ENSG00000189298.14 | *ZKSCAN3* | -2.1 | 1.07E-02 | 40.0 | 19.2 |
| ENSG00000170949.17 | *ZNF160* | 1.8 | 8.45E-03 | 52.0 | 92.3 |
| ENSG00000147118.11 | *ZNF182* | -2.3 | 4.34E-02 | 42.0 | 18.5 |
| ENSG00000198393.8 | *ZNF26* | 1.7 | 3.05E-03 | 69.5 | 121.4 |
| ENSG00000185947.15 | *ZNF267* | 1.7 | 1.53E-02 | 81.5 | 137.9 |
| ENSG00000162702.8 | *ZNF281* | 1.9 | 1.98E-31 | 712.0 | 1359.4 |
| ENSG00000083812.12 | *ZNF324* | 1.6 | 2.53E-04 | 115.0 | 179.8 |
| ENSG00000088876.12 | *ZNF343* | -1.5 | 7.90E-04 | 214.0 | 138.1 |
| ENSG00000175213.3 | *ZNF408* | 1.5 | 5.13E-06 | 117.5 | 179.8 |
| ENSG00000125945.15 | *ZNF436* | -1.7 | 2.17E-07 | 211.0 | 122.0 |
| ENSG00000180035.12 | *ZNF48* | 1.9 | 8.22E-03 | 104.5 | 194.3 |
| ENSG00000265763.4 | *ZNF488* | -3.1 | 5.12E-03 | 23.7 | 7.5 |
| ENSG00000269343.8 | *ZNF587B* | 1.8 | 3.89E-02 | 36.3 | 66.8 |
| ENSG00000213020.10 | *ZNF611* | 2.1 | 1.30E-02 | 26.5 | 55.3 |
| ENSG00000167528.12 | *ZNF641* | 1.7 | 2.89E-02 | 52.6 | 91.9 |
| ENSG00000251192.7 | *ZNF674* | -2.3 | 1.99E-02 | 30.8 | 13.5 |
| ENSG00000230844.3 | *ZNF674-AS1* | 2.2 | 6.80E-13 | 64.1 | 138.5 |
| ENSG00000143067.5 | *ZNF697* | 3.3 | 8.29E-13 | 76.4 | 253.6 |
| ENSG00000196110.8 | *ZNF699* | 1.9 | 1.24E-03 | 71.5 | 133.1 |
| ENSG00000183779.7 | *ZNF703* | 1.7 | 4.04E-08 | 131.3 | 223.1 |
| ENSG00000196391.11 | *ZNF774* | -2.8 | 4.15E-02 | 44.2 | 15.7 |
| ENSG00000196456.13 | *ZNF775* | -2.1 | 9.08E-04 | 60.5 | 28.9 |
| ENSG00000170396.8 | *ZNF804A* | 1.5 | 7.79E-03 | 85.3 | 129.9 |
| ENSG00000198040.10 | *ZNF84* | 1.6 | 2.39E-04 | 101.7 | 164.9 |
| ENSG00000184635.16 | *ZNF93* | 1.5 | 3.80E-02 | 33.8 | 52.0 |
| ENSG00000137185.12 | *ZSCAN9* | 1.8 | 3.54E-03 | 61.6 | 112.4 |
| ENSG00000132801.7 | *ZSWIM3* | -1.9 | 1.68E-02 | 25.0 | 13.0 |
| ENSG00000130449.6 | *ZSWIM6* | 1.8 | 1.51E-09 | 234.5 | 422.3 |
| ENSG00000159840.16 | *ZYX* | 1.5 | 5.27E-29 | 3715.4 | 5680.8 |

**Supplementary Table 4. Differentially expressed genes in CGL1^FRA1KO^ cells 2 hours after serum stimulation relative to baseline.**

To characterize the early transcriptional response to serum stimulation in the absence of FRA1, RNA-sequencing was performed on CGL1^FRA1KO^ cells collected 2 hours after media change and compared to baseline (0-hour) conditions. DEGs were identified using a fold change (F.C.) threshold >1.5 or <–1.5 and an FDR-adjusted p-value (p-adj) < 0.05. A total of 480 DEGs were identified, including 364 upregulated and 116 downregulated genes. This dataset represents the serum-induced gene expression program in FRA1-deficient CGL1 cells. The table includes gene names, fold changes, p-adj values, and transcript abundance (TPM), sorted alphabetically.

| **Gene ID** | **Gene Name** | **F.C.** | **p-adj.** | **Baseline** | **2h Post Serum** |
| --- | --- | --- | --- | --- | --- |
| ENSG00000124574.15 | *ABCC10* | -1.6 | 5.48E-04 | 335.9 | 205.7 |
| ENSG00000268812.3 | *AC004264.1* | 7.1 | 7.91E-04 | 4.6 | 32.5 |
| ENSG00000205325.3 | *AC005863.1* | -2.0 | 1.49E-05 | 208.3 | 102.8 |
| ENSG00000267519.6 | *AC020916.1* | 2.8 | 1.76E-53 | 402.4 | 1124.9 |
| ENSG00000259820.1 | *AC083843.3* | 2.2 | 6.52E-03 | 35.3 | 79.1 |
| ENSG00000111271.14 | *ACAD10* | -2.2 | 1.18E-02 | 114.3 | 52.3 |
| ENSG00000117054.13 | *ACADM* | 1.7 | 1.99E-02 | 135.5 | 226.1 |
| ENSG00000107796.13 | *ACTA2* | -2.9 | 9.62E-11 | 461.9 | 159.9 |
| ENSG00000154734.15 | *ADAMTS1* | -3.0 | 2.57E-18 | 546.3 | 182.7 |
| ENSG00000148926.10 | *ADM* | 1.8 | 1.65E-29 | 857.6 | 1542.2 |
| ENSG00000135541.21 | *AHI1* | 1.6 | 3.93E-03 | 183.6 | 293.1 |
| ENSG00000131016.17 | *AKAP12* | 2.4 | ######## | 2860.8 | 6800.7 |
| ENSG00000278139.1 | *AL358075.4* | -2906680.6 | 1.58E-06 | 30.1 | 0.0 |
| ENSG00000285130.2 | *AL358113.1* | -8.8 | 1.02E-04 | 40.3 | 4.6 |
| ENSG00000217801.10 | *AL390719.1* | 5.6 | 3.09E-02 | 4.6 | 25.9 |
| ENSG00000119711.13 | *ALDH6A1* | 1.6 | 2.18E-02 | 140.9 | 221.5 |
| ENSG00000198796.7 | *ALPK2* | 1.7 | 3.36E-10 | 554.2 | 920.2 |
| ENSG00000003393.15 | *ALS2* | 2.2 | 6.92E-03 | 46.0 | 101.5 |
| ENSG00000139211.6 | *AMIGO2* | 3.3 | 2.09E-05 | 146.6 | 487.5 |
| ENSG00000167772.12 | *ANGPTL4* | 2.3 | 2.16E-06 | 79.9 | 184.6 |
| ENSG00000168876.9 | *ANKRD49* | -1.6 | 2.08E-02 | 209.0 | 131.3 |
| ENSG00000120868.13 | *APAF1* | -2.0 | 2.49E-02 | 119.0 | 59.1 |
| ENSG00000198576.4 | *ARC* | 6.5 | 6.45E-06 | 8.5 | 54.8 |
| ENSG00000146376.11 | *ARHGAP18* | 1.5 | 1.10E-07 | 419.0 | 642.8 |
| ENSG00000150347.16 | *ARID5B* | -1.5 | 1.00E-16 | 1633.0 | 1072.3 |
| ENSG00000188042.8 | *ARL4C* | 1.7 | 4.44E-03 | 89.3 | 154.4 |
| ENSG00000165997.5 | *ARL5B* | 1.8 | 4.37E-22 | 762.5 | 1406.4 |
| ENSG00000113369.9 | *ARRDC3* | 1.5 | 8.41E-04 | 442.9 | 676.3 |
| ENSG00000204147.10 | *ASAH2B* | 2.4 | 1.43E-02 | 75.2 | 178.7 |
| ENSG00000066279.18 | *ASPM* | 1.6 | 2.16E-02 | 2753.2 | 4321.8 |
| ENSG00000176208.9 | *ATAD5* | 1.5 | 5.46E-04 | 262.8 | 402.8 |
| ENSG00000162772.17 | *ATF3* | 2.8 | 3.75E-29 | 450.0 | 1281.2 |
| ENSG00000168874.13 | *ATOH8* | -1.9 | 4.97E-04 | 214.9 | 115.4 |
| ENSG00000171953.16 | *ATPAF2* | 1.7 | 2.25E-04 | 143.9 | 243.5 |
| ENSG00000085224.22 | *ATRX* | 1.9 | 2.40E-11 | 1031.4 | 1982.0 |
| ENSG00000135454.14 | *B4GALNT1* | -1.7 | 3.06E-02 | 118.3 | 70.3 |
| ENSG00000156273.16 | *BACH1* | 2.1 | 2.18E-34 | 571.1 | 1179.5 |
| ENSG00000166170.9 | *BAG5* | 146.1 | 3.15E-05 | 0.2 | 26.6 |
| ENSG00000137936.18 | *BCAR3* | 1.6 | 1.52E-07 | 446.8 | 724.9 |
| ENSG00000142867.14 | *BCL10* | 2.1 | 2.92E-15 | 275.9 | 577.8 |
| ENSG00000069399.15 | *BCL3* | 3.0 | 3.81E-23 | 150.9 | 450.8 |
| ENSG00000186174.12 | *BCL9L* | 2.0 | 2.66E-03 | 91.8 | 181.4 |
| ENSG00000183337.17 | *BCOR* | 2.1 | 9.26E-08 | 526.1 | 1092.0 |
| ENSG00000176697.19 | *BDNF* | 1.9 | 7.41E-28 | 781.0 | 1454.9 |
| ENSG00000134107.5 | *BHLHE40* | 10.2 | 0.00E+00 | 332.9 | 3407.4 |
| ENSG00000151746.14 | *BICD1* | 1.8 | 1.78E-07 | 251.4 | 440.0 |
| ENSG00000197299.12 | *BLM* | 2.0 | 9.02E-07 | 146.6 | 291.8 |
| ENSG00000125378.16 | *BMP4* | -1.6 | 4.29E-11 | 1673.1 | 1039.4 |
| ENSG00000196544.8 | *BORCS6* | -1.7 | 2.41E-02 | 117.9 | 70.2 |
| ENSG00000167173.19 | *C15orf39* | 1.5 | 3.64E-07 | 500.6 | 766.0 |
| ENSG00000155330.10 | *C16orf87* | 1.8 | 3.86E-03 | 143.8 | 263.3 |
| ENSG00000137752.24 | *CASP1* | -6.2 | 1.98E-02 | 32.4 | 5.3 |
| ENSG00000078699.21 | *CBFA2T2* | -4.7 | 2.69E-02 | 125.6 | 26.8 |
| ENSG00000141582.15 | *CBX4* | 1.7 | 8.09E-10 | 360.8 | 614.7 |
| ENSG00000115355.17 | *CCDC88A* | 1.8 | 8.58E-07 | 753.5 | 1348.8 |
| ENSG00000188549.12 | *CCDC9B* | -6.9 | 3.94E-02 | 196.0 | 28.3 |
| ENSG00000108691.9 | *CCL2* | 2.0 | 6.02E-12 | 293.7 | 588.7 |
| ENSG00000142871.17 | *CCN1* | 1.9 | ######## | 11184.8 | 21606.5 |
| ENSG00000118523.6 | *CCN2* | 6.6 | 0.00E+00 | 3030.4 | 19936.0 |
| ENSG00000110092.3 | *CCND1* | 1.8 | 3.99E-02 | 75.4 | 135.2 |
| ENSG00000162063.13 | *CCNF* | -1.9 | 1.31E-20 | 1118.3 | 604.4 |
| ENSG00000138764.15 | *CCNG2* | -1.7 | 6.70E-03 | 223.0 | 130.0 |
| ENSG00000163660.11 | *CCNL1* | 1.6 | 3.77E-08 | 802.6 | 1248.5 |
| ENSG00000082258.13 | *CCNT2* | 1.8 | 9.65E-03 | 231.3 | 426.1 |
| ENSG00000120217.14 | *CD274* | 3.9 | 9.50E-09 | 38.0 | 147.4 |
| ENSG00000112149.10 | *CD83* | 1.9 | 9.16E-10 | 196.5 | 379.6 |
| ENSG00000167797.7 | *CDK2AP2* | -1.5 | 4.63E-02 | 173.2 | 112.2 |
| ENSG00000111276.11 | *CDKN1B* | -1.7 | 8.28E-16 | 1214.2 | 732.8 |
| ENSG00000115816.15 | *CEBPZ* | 1.5 | 6.80E-09 | 950.6 | 1466.3 |
| ENSG00000138778.12 | *CENPE* | 1.9 | 1.84E-02 | 195.8 | 370.6 |
| ENSG00000117724.13 | *CENPF* | 1.7 | 1.09E-10 | 3306.6 | 5560.7 |
| ENSG00000100629.17 | *CEP128* | 1.9 | 1.15E-03 | 209.6 | 387.9 |
| ENSG00000174799.11 | *CEP135* | 1.7 | 4.70E-02 | 109.5 | 189.1 |
| ENSG00000172292.14 | *CERS6* | 5.6 | 4.83E-02 | 6.9 | 38.7 |
| ENSG00000206530.11 | *CFAP44* | 2.0 | 1.48E-02 | 55.8 | 113.4 |
| ENSG00000128965.13 | *CHAC1* | 3.1 | 1.42E-16 | 88.8 | 277.3 |
| ENSG00000177200.17 | *CHD9* | 1.6 | 9.58E-04 | 442.6 | 689.4 |
| ENSG00000179862.7 | *CITED4* | 2.4 | 4.49E-04 | 54.2 | 127.8 |
| ENSG00000175505.11 | *CLCF1* | 2.0 | 2.22E-05 | 139.7 | 272.4 |
| ENSG00000169583.13 | *CLIC3* | -1.7 | 8.17E-08 | 500.1 | 299.4 |
| ENSG00000162368.13 | *CMPK1* | 6.2 | 5.33E-03 | 8.6 | 53.4 |
| ENSG00000162852.14 | *CNST* | 1.5 | 5.32E-06 | 488.6 | 743.3 |
| ENSG00000044459.15 | *CNTLN* | 1.6 | 8.94E-06 | 311.0 | 489.8 |
| ENSG00000108797.12 | *CNTNAP1* | -1.6 | 8.99E-07 | 620.1 | 396.2 |
| ENSG00000082438.17 | *COBLL1* | 1.6 | 4.35E-02 | 189.4 | 312.0 |
| ENSG00000182871.16 | *COL18A1* | -1.5 | 1.55E-04 | 833.2 | 541.4 |
| ENSG00000187498.16 | *COL4A1* | -1.5 | 9.99E-13 | 3186.5 | 2085.7 |
| ENSG00000158270.12 | *COLEC12* | 1.8 | 3.72E-02 | 53.9 | 97.9 |
| ENSG00000166200.15 | *COPS2* | 1.5 | 5.95E-07 | 1147.4 | 1752.4 |
| ENSG00000110880.10 | *CORO1C* | -1.6 | 4.51E-02 | 184.4 | 114.2 |
| ENSG00000163751.4 | *CPA3* | 1.9 | 2.81E-02 | 53.9 | 101.4 |
| ENSG00000113742.14 | *CPEB4* | 2.0 | 2.29E-02 | 68.8 | 137.3 |
| ENSG00000197603.14 | *CPLANE1* | 1.6 | 4.01E-02 | 267.1 | 428.8 |
| ENSG00000080947.15 | *CROCCP3* | -3.6 | 4.79E-02 | 48.0 | 13.4 |
| ENSG00000121671.12 | *CRY2* | 1.6 | 8.50E-03 | 102.2 | 166.2 |
| ENSG00000109846.9 | *CRYAB* | -2.3 | 2.28E-02 | 58.4 | 25.7 |
| ENSG00000144655.15 | *CSRNP1* | 5.3 | 1.30E-74 | 178.8 | 939.1 |
| ENSG00000108094.14 | *CUL2* | 1.7 | 1.64E-03 | 166.2 | 278.5 |
| ENSG00000163510.14 | *CWC22* | 1.6 | 2.59E-04 | 251.7 | 405.0 |
| ENSG00000169429.11 | *CXCL8* | 7.3 | 2.07E-09 | 14.2 | 103.7 |
| ENSG00000138061.12 | *CYP1B1* | 1.8 | 7.61E-48 | 2547.8 | 4534.9 |
| ENSG00000180902.18 | *D2HGDH* | -1.7 | 1.17E-02 | 131.0 | 75.0 |
| ENSG00000188215.10 | *DCUN1D3* | 1.8 | 2.44E-07 | 232.1 | 425.0 |
| ENSG00000168209.5 | *DDIT4* | 2.2 | 1.72E-17 | 595.7 | 1294.3 |
| ENSG00000181381.13 | *DDX60L* | 1.7 | 4.86E-02 | 110.5 | 187.0 |
| ENSG00000062282.15 | *DGAT2* | -1.6 | 2.09E-02 | 165.7 | 101.7 |
| ENSG00000065357.20 | *DGKA* | -1.6 | 3.36E-02 | 261.6 | 165.1 |
| ENSG00000162496.9 | *DHRS3* | 1.9 | 7.58E-04 | 100.8 | 194.4 |
| ENSG00000164741.15 | *DLC1* | 1.6 | 1.34E-05 | 583.9 | 956.1 |
| ENSG00000115844.11 | *DLX2* | 4.3 | 1.08E-37 | 102.5 | 439.2 |
| ENSG00000135164.18 | *DMTF1* | 1.6 | 7.58E-03 | 307.6 | 502.8 |
| ENSG00000135924.15 | *DNAJB2* | -1.8 | 1.00E-04 | 216.3 | 120.3 |
| ENSG00000128590.5 | *DNAJB9* | -2.0 | 1.60E-04 | 172.9 | 87.3 |
| ENSG00000107554.17 | *DNMBP* | 1.7 | 1.26E-06 | 356.7 | 619.2 |
| ENSG00000120129.6 | *DUSP1* | 1.8 | 3.66E-14 | 593.0 | 1049.2 |
| ENSG00000158050.5 | *DUSP2* | 3.8 | 4.26E-28 | 109.7 | 421.0 |
| ENSG00000120875.9 | *DUSP4* | 3.4 | 4.88E-80 | 367.9 | 1234.1 |
| ENSG00000138166.6 | *DUSP5* | 10.6 | 1.10E-95 | 98.6 | 1045.7 |
| ENSG00000139318.8 | *DUSP6* | 3.5 | 3.54E-12 | 75.6 | 261.5 |
| ENSG00000184545.11 | *DUSP8* | 1.7 | 2.29E-02 | 243.7 | 422.4 |
| ENSG00000187240.16 | *DYNC2H1* | 1.7 | 1.08E-02 | 367.4 | 636.3 |
| ENSG00000078401.7 | *EDN1* | 2.9 | 3.48E-61 | 407.2 | 1190.0 |
| ENSG00000243364.8 | *EFNA4* | -1.6 | 4.41E-02 | 122.0 | 75.8 |
| ENSG00000120738.8 | *EGR1* | 12.0 | 0.00E+00 | 353.0 | 4230.7 |
| ENSG00000122877.16 | *EGR2* | 47.1 | 2.33E-09 | 1.6 | 73.2 |
| ENSG00000179388.9 | *EGR3* | 52.1 | 1.60E-49 | 6.3 | 326.0 |
| ENSG00000115504.14 | *EHBP1* | 1.7 | 3.66E-02 | 96.8 | 162.8 |
| ENSG00000175376.9 | *EIF1AD* | 1.5 | 1.50E-05 | 322.2 | 497.8 |
| ENSG00000198692.10 | *EIF1AY* | 1.6 | 1.85E-03 | 228.8 | 365.5 |
| ENSG00000151247.12 | *EIF4E* | 2.0 | 1.21E-02 | 79.0 | 160.1 |
| ENSG00000158417.11 | *EIF5B* | 1.6 | 3.29E-09 | 1574.6 | 2497.3 |
| ENSG00000158711.13 | *ELK4* | 2.4 | 3.31E-03 | 34.6 | 83.0 |
| ENSG00000066629.17 | *EML1* | 5.8 | 1.48E-02 | 8.5 | 48.6 |
| ENSG00000171617.14 | *ENC1* | 4.4 | 1.40E-06 | 37.3 | 163.7 |
| ENSG00000142627.13 | *EPHA2* | 2.1 | ######## | 3056.8 | 6380.0 |
| ENSG00000182150.16 | *ERCC6L2* | 3.3 | 1.40E-03 | 27.4 | 89.6 |
| ENSG00000116285.13 | *ERRFI1* | 6.8 | 0.00E+00 | 1406.4 | 9600.7 |
| ENSG00000157557.13 | *ETS2* | 1.9 | 9.50E-27 | 687.2 | 1282.1 |
| ENSG00000164251.5 | *F2RL1* | 1.5 | 2.52E-07 | 399.2 | 612.2 |
| ENSG00000117525.14 | *F3* | 4.7 | 0.00E+00 | 1993.7 | 9283.2 |
| ENSG00000156504.16 | *FAM122B* | 1.7 | 2.21E-05 | 409.1 | 687.5 |
| ENSG00000154319.16 | *FAM167A* | 2.6 | 7.21E-07 | 57.4 | 147.5 |
| ENSG00000185112.6 | *FAM43A* | 2.0 | 5.61E-07 | 152.2 | 303.5 |
| ENSG00000188522.15 | *FAM83G* | 1.5 | 1.52E-07 | 602.0 | 923.9 |
| ENSG00000173295.7 | *FAM86B3P* | 3.6 | 3.12E-02 | 10.3 | 37.6 |
| ENSG00000181544.15 | *FANCB* | 3.2 | 4.66E-03 | 38.5 | 124.1 |
| ENSG00000078098.14 | *FAP* | 1.6 | 6.43E-03 | 160.6 | 259.7 |
| ENSG00000179431.7 | *FJX1* | 1.6 | 2.98E-08 | 416.5 | 659.9 |
| ENSG00000204315.4 | *FKBPL* | -1.6 | 2.16E-02 | 179.1 | 114.6 |
| ENSG00000125848.10 | *FLRT3* | -2.5 | 7.30E-03 | 72.1 | 28.6 |
| ENSG00000102755.12 | *FLT1* | -4.6 | 1.57E-03 | 52.0 | 11.2 |
| ENSG00000248905.9 | *FMN1* | 1.7 | 4.09E-05 | 405.3 | 683.5 |
| ENSG00000170345.10 | *FOS* | 3.5 | 4.30E-09 | 40.6 | 141.1 |
| ENSG00000125740.14 | *FOSB* | 29.7 | 1.21E-77 | 16.9 | 501.4 |
| ENSG00000175592.9 | *FOSL1* | 3.5 | ######## | 1063.5 | 3697.1 |
| ENSG00000075426.12 | *FOSL2* | 1.6 | 1.79E-29 | 1938.4 | 3018.8 |
| ENSG00000176692.8 | *FOXC2* | 3.4 | 8.26E-10 | 47.8 | 161.1 |
| ENSG00000170802.16 | *FOXN2* | 1.5 | 3.47E-04 | 283.3 | 433.8 |
| ENSG00000181274.7 | *FRAT2* | -2.5 | 9.00E-11 | 259.5 | 104.4 |
| ENSG00000265817.3 | *FSBP* | 2.0 | 3.11E-02 | 36.5 | 74.0 |
| ENSG00000116717.13 | *GADD45A* | 2.2 | 3.37E-56 | 858.6 | 1907.4 |
| ENSG00000099860.9 | *GADD45B* | 1.9 | 5.22E-37 | 1546.1 | 2944.2 |
| ENSG00000253669.4 | *GASAL1* | 3.2 | 2.42E-05 | 24.8 | 80.6 |
| ENSG00000130513.6 | *GDF15* | 5.3 | ######## | 237.6 | 1264.6 |
| ENSG00000074047.21 | *GLI2* | -3.6 | 1.74E-02 | 36.4 | 10.1 |
| ENSG00000127920.6 | *GNG11* | 1.6 | 3.83E-03 | 143.5 | 235.2 |
| ENSG00000164850.15 | *GPER1* | -1.8 | 1.48E-03 | 190.0 | 107.1 |
| ENSG00000173264.14 | *GPR137* | -1.7 | 6.43E-03 | 190.9 | 114.3 |
| ENSG00000169508.7 | *GPR183* | 10.8 | 1.21E-04 | 3.3 | 36.1 |
| ENSG00000181773.7 | *GPR3* | 3.2 | 6.25E-07 | 37.1 | 119.1 |
| ENSG00000167191.12 | *GPRC5B* | -1.8 | 7.12E-05 | 226.6 | 126.2 |
| ENSG00000164284.15 | *GRPEL2* | 1.7 | 4.90E-17 | 641.3 | 1103.5 |
| ENSG00000134202.11 | *GSTM3* | -1.6 | 2.27E-16 | 1698.8 | 1054.1 |
| ENSG00000168765.17 | *GSTM4* | -1.6 | 7.52E-15 | 1040.4 | 631.7 |
| ENSG00000113070.8 | *HBEGF* | 2.3 | 2.55E-16 | 237.0 | 538.4 |
| ENSG00000180229.12 | *HERC2P3* | -1.7 | 5.33E-03 | 235.4 | 142.1 |
| ENSG00000138642.14 | *HERC6* | -6.6 | 2.20E-02 | 31.5 | 4.8 |
| ENSG00000114315.4 | *HES1* | 14.4 | ######## | 84.6 | 1216.2 |
| ENSG00000188290.10 | *HES4* | 1.6 | 1.69E-02 | 137.6 | 218.0 |
| ENSG00000177374.13 | *HIC1* | 2.2 | 7.12E-05 | 62.2 | 139.7 |
| ENSG00000095951.17 | *HIVEP1* | 1.5 | 1.32E-06 | 525.6 | 813.3 |
| ENSG00000136630.13 | *HLX* | -3.9 | 1.67E-14 | 246.4 | 62.7 |
| ENSG00000112972.15 | *HMGCS1* | 1.6 | 2.99E-18 | 1344.0 | 2143.8 |
| ENSG00000108511.10 | *HOXB6* | -2.0 | 1.62E-02 | 86.3 | 43.5 |
| ENSG00000284024.2 | *HSPA14* | 2.1 | 1.35E-04 | 78.5 | 168.6 |
| ENSG00000204389.10 | *HSPA1A* | -1.6 | 1.22E-32 | 4586.4 | 2957.9 |
| ENSG00000125968.9 | *ID1* | 1.8 | 7.12E-49 | 1619.6 | 2953.0 |
| ENSG00000119912.17 | *IDE* | -1.5 | 3.09E-02 | 380.1 | 246.6 |
| ENSG00000067064.11 | *IDI1* | 2.1 | 8.92E-03 | 74.2 | 154.7 |
| ENSG00000160888.7 | *IER2* | 3.2 | ######## | 1057.5 | 3349.4 |
| ENSG00000137331.12 | *IER3* | 8.8 | 0.00E+00 | 795.8 | 6995.2 |
| ENSG00000188483.8 | *IER5L* | 2.1 | 3.31E-21 | 411.6 | 848.9 |
| ENSG00000169991.11 | *IFFO2* | 2.2 | 5.95E-41 | 638.5 | 1388.8 |
| ENSG00000137959.16 | *IFI44L* | -1.6 | 3.91E-02 | 277.8 | 170.4 |
| ENSG00000159128.14 | *IFNGR2* | 2.1 | 2.56E-02 | 32.6 | 69.9 |
| ENSG00000096872.16 | *IFT74* | 1.7 | 4.81E-02 | 67.9 | 114.2 |
| ENSG00000115461.5 | *IGFBP5* | -1.6 | 5.30E-04 | 276.1 | 173.1 |
| ENSG00000008517.16 | *IL32* | -2.5 | 1.10E-09 | 343.8 | 137.4 |
| ENSG00000136244.12 | *IL6* | 3.8 | 1.25E-20 | 67.9 | 257.6 |
| ENSG00000168556.7 | *ING2* | 1.6 | 3.82E-02 | 83.4 | 133.8 |
| ENSG00000168395.15 | *ING5* | 1.7 | 9.96E-03 | 139.9 | 232.8 |
| ENSG00000186480.13 | *INSIG1* | 1.9 | 1.32E-37 | 1148.4 | 2167.5 |
| ENSG00000164066.13 | *INTU* | -1.8 | 3.35E-02 | 134.8 | 76.0 |
| ENSG00000106012.18 | *IQCE* | 1.7 | 5.00E-04 | 214.0 | 367.6 |
| ENSG00000125347.14 | *IRF1* | 1.7 | 5.41E-08 | 399.8 | 667.0 |
| ENSG00000119669.5 | *IRF2BPL* | 2.2 | 1.77E-54 | 747.0 | 1660.3 |
| ENSG00000213949.10 | *ITGA1* | 1.7 | 2.80E-02 | 134.5 | 226.7 |
| ENSG00000132470.14 | *ITGB4* | 1.7 | 4.55E-02 | 88.1 | 152.3 |
| ENSG00000198542.14 | *ITGBL1* | 1.6 | 1.07E-12 | 705.7 | 1126.7 |
| ENSG00000086544.3 | *ITPKC* | 1.8 | 4.23E-02 | 81.4 | 142.5 |
| ENSG00000148841.17 | *ITPRIP* | 1.7 | 3.17E-12 | 419.0 | 731.1 |
| ENSG00000171988.19 | *JMJD1C* | 1.6 | 7.63E-04 | 655.9 | 1068.2 |
| ENSG00000177606.7 | *JUN* | 2.6 | 2.96E-39 | 511.2 | 1318.0 |
| ENSG00000171223.6 | *JUNB* | 7.5 | 0.00E+00 | 592.2 | 4465.0 |
| ENSG00000170852.11 | *KBTBD2* | 1.7 | 1.83E-15 | 818.3 | 1377.1 |
| ENSG00000120696.9 | *KBTBD7* | -1.7 | 3.05E-02 | 132.9 | 78.7 |
| ENSG00000184185.10 | *KCNJ12* | 2.5 | 8.47E-07 | 67.2 | 168.7 |
| ENSG00000164626.9 | *KCNK5* | 1.6 | 5.70E-03 | 118.9 | 189.2 |
| ENSG00000156113.23 | *KCNMA1* | 1.8 | 7.42E-03 | 89.6 | 165.1 |
| ENSG00000132510.10 | *KDM6B* | 14.7 | 1.46E-26 | 42.1 | 620.3 |
| ENSG00000118193.12 | *KIF14* | 1.5 | 9.07E-07 | 864.0 | 1310.0 |
| ENSG00000138182.14 | *KIF20B* | 1.6 | 2.22E-04 | 979.7 | 1561.0 |
| ENSG00000155090.15 | *KLF10* | 7.3 | 0.00E+00 | 618.6 | 4491.3 |
| ENSG00000172059.11 | *KLF11* | 1.6 | 1.90E-05 | 382.6 | 605.0 |
| ENSG00000129911.9 | *KLF16* | 1.5 | 9.56E-06 | 732.1 | 1112.7 |
| ENSG00000136826.15 | *KLF4* | 1.7 | 9.13E-15 | 598.6 | 1003.8 |
| ENSG00000212724.3 | *KRTAP2-3* | 4.0 | 1.67E-06 | 20.1 | 81.3 |
| ENSG00000126777.18 | *KTN1* | 1.5 | 2.80E-07 | 3483.2 | 5379.5 |
| ENSG00000130164.13 | *LDLR* | 2.0 | 3.44E-04 | 97.2 | 198.1 |
| ENSG00000106003.13 | *LFNG* | 2.1 | 1.26E-09 | 218.5 | 454.9 |
| ENSG00000128342.5 | *LIF* | 7.0 | ######## | 309.4 | 2161.5 |
| ENSG00000233237.8 | *LINC00472* | 1.7 | 1.44E-02 | 176.7 | 297.7 |
| ENSG00000249628.3 | *LINC00942* | -1.7 | 6.86E-06 | 419.7 | 244.1 |
| ENSG00000226053.1 | *LINC01776* | 3.4 | 7.39E-04 | 19.2 | 66.0 |
| ENSG00000185621.11 | *LMLN* | 3.5 | 2.04E-02 | 18.2 | 64.5 |
| ENSG00000198121.13 | *LPAR1* | 2.7 | 1.38E-02 | 18.5 | 50.2 |
| ENSG00000107816.17 | *LZTS2* | -1.6 | 1.92E-03 | 251.1 | 154.0 |
| ENSG00000185022.12 | *MAFF* | 5.8 | 1.78E-64 | 130.6 | 762.2 |
| ENSG00000198517.10 | *MAFK* | 2.0 | 2.54E-39 | 721.3 | 1441.2 |
| ENSG00000034152.19 | *MAP2K3* | 2.0 | 9.86E-25 | 753.3 | 1520.3 |
| ENSG00000006062.17 | *MAP3K14* | 2.1 | 8.24E-47 | 818.1 | 1744.3 |
| ENSG00000069956.12 | *MAPK6* | 1.5 | 9.90E-07 | 966.2 | 1489.8 |
| ENSG00000180611.7 | *MB21D2* | 2.0 | 4.46E-25 | 505.1 | 1027.7 |
| ENSG00000143384.13 | *MCL1* | 1.9 | 2.59E-92 | 4530.1 | 8416.8 |
| ENSG00000166508.17 | *MCM7* | 2.1 | 3.86E-03 | 72.2 | 154.2 |
| ENSG00000135679.25 | *MDM2* | 1.8 | 2.22E-02 | 60.4 | 107.9 |
| ENSG00000116604.18 | *MEF2D* | 1.5 | 8.67E-10 | 598.9 | 926.8 |
| ENSG00000162591.16 | *MEGF6* | -2.8 | 6.26E-08 | 282.2 | 99.4 |
| ENSG00000254726.3 | *MEX3A* | -1.6 | 2.78E-07 | 584.6 | 359.5 |
| ENSG00000168389.17 | *MFSD2A* | 5.0 | 3.59E-02 | 7.1 | 35.7 |
| ENSG00000135596.18 | *MICAL1* | -1.6 | 3.01E-03 | 256.3 | 159.1 |
| ENSG00000108788.11 | *MLX* | 2.0 | 1.43E-04 | 111.5 | 217.8 |
| ENSG00000146263.11 | *MMS22L* | 1.7 | 5.44E-03 | 265.8 | 460.0 |
| ENSG00000165943.5 | *MOAP1* | 1.7 | 3.07E-07 | 228.9 | 396.6 |
| ENSG00000196588.17 | *MRTFA* | -1.6 | 1.40E-02 | 322.8 | 202.8 |
| ENSG00000116062.15 | *MSH6* | 5.2 | 4.04E-03 | 14.1 | 72.7 |
| ENSG00000110921.14 | *MVK* | 1.8 | 2.88E-02 | 92.0 | 166.3 |
| ENSG00000101825.8 | *MXRA5* | -1.8 | 2.25E-12 | 697.7 | 393.1 |
| ENSG00000179820.16 | *MYADM* | 2.0 | 9.18E-30 | 1501.5 | 2956.9 |
| ENSG00000132382.14 | *MYBBP1A* | 1.7 | 2.26E-04 | 241.2 | 398.6 |
| ENSG00000172927.8 | *MYEOV* | 1.8 | 5.88E-03 | 77.3 | 138.1 |
| ENSG00000101335.10 | *MYL9* | -1.7 | 6.73E-26 | 2464.2 | 1429.7 |
| ENSG00000162601.11 | *MYSM1* | 2.0 | 1.09E-04 | 131.2 | 261.0 |
| ENSG00000156239.12 | *N6AMT1* | -1.6 | 3.35E-02 | 215.7 | 132.3 |
| ENSG00000138386.17 | *NAB1* | 1.7 | 2.46E-05 | 316.9 | 547.7 |
| ENSG00000166886.13 | *NAB2* | 4.6 | ######## | 356.9 | 1648.1 |
| ENSG00000111912.20 | *NCOA7* | 1.6 | 6.00E-09 | 581.9 | 945.2 |
| ENSG00000143217.9 | *NECTIN4* | -4.2 | 1.08E-02 | 28.9 | 6.9 |
| ENSG00000111859.17 | *NEDD9* | 2.1 | 7.01E-49 | 893.4 | 1862.9 |
| ENSG00000100906.10 | *NFKBIA* | 3.3 | 2.52E-03 | 20.6 | 67.6 |
| ENSG00000151014.6 | *NOCT* | 2.0 | 9.62E-11 | 242.5 | 492.4 |
| ENSG00000113389.16 | *NPR3* | -1.7 | 4.39E-06 | 426.0 | 247.5 |
| ENSG00000123358.20 | *NR4A1* | 7.7 | ######## | 155.0 | 1193.4 |
| ENSG00000119508.18 | *NR4A3* | 14.2 | 1.57E-11 | 5.0 | 71.2 |
| ENSG00000180530.11 | *NRIP1* | 1.8 | 1.32E-04 | 251.4 | 453.2 |
| ENSG00000111696.12 | *NT5DC3* | 2.1 | 9.31E-14 | 275.1 | 574.0 |
| ENSG00000163545.10 | *NUAK2* | 6.1 | 3.09E-56 | 98.6 | 597.2 |
| ENSG00000235636.1 | *NUS1P1* | 1.8 | 1.23E-03 | 99.8 | 178.2 |
| ENSG00000144227.5 | *NXPH2* | -1.9 | 2.93E-03 | 117.8 | 61.5 |
| ENSG00000173391.9 | *OLR1* | -1.6 | 3.48E-11 | 1086.1 | 698.7 |
| ENSG00000054277.14 | *OPN3* | -1.6 | 3.92E-05 | 387.8 | 244.9 |
| ENSG00000183444.10 | *OR7E38P* | -2.3 | 3.15E-02 | 51.5 | 22.5 |
| ENSG00000164823.11 | *OSGIN2* | 1.8 | 8.42E-11 | 275.2 | 507.5 |
| ENSG00000117385.15 | *P3H1* | -1.7 | 1.99E-02 | 336.9 | 203.6 |
| ENSG00000142623.11 | *PADI1* | 2.4 | 3.75E-03 | 33.2 | 80.2 |
| ENSG00000182752.10 | *PAPPA* | -1.6 | 2.90E-02 | 153.7 | 98.0 |
| ENSG00000178685.14 | *PARP10* | -1.6 | 1.94E-02 | 292.3 | 184.5 |
| ENSG00000132849.20 | *PATJ* | 1.6 | 1.46E-02 | 286.5 | 460.1 |
| ENSG00000138650.9 | *PCDH10* | -1.6 | 3.05E-02 | 151.6 | 93.3 |
| ENSG00000169851.15 | *PCDH7* | 1.9 | 1.94E-11 | 304.9 | 582.0 |
| ENSG00000160613.13 | *PCSK7* | 2.4 | 2.57E-04 | 129.2 | 307.0 |
| ENSG00000100311.17 | *PDGFB* | 4.4 | 2.59E-48 | 258.3 | 1133.0 |
| ENSG00000152256.13 | *PDK1* | 1.8 | 1.13E-02 | 64.3 | 115.8 |
| ENSG00000164951.16 | *PDP1* | 1.9 | 1.63E-15 | 426.0 | 801.5 |
| ENSG00000090857.13 | *PDPR* | 1.9 | 2.20E-02 | 60.2 | 113.6 |
| ENSG00000139289.13 | *PHLDA1* | 5.7 | ######## | 339.4 | 1927.9 |
| ENSG00000144824.21 | *PHLDB2* | 1.6 | 2.52E-19 | 1656.8 | 2602.0 |
| ENSG00000164530.15 | *PI16* | -1.7 | 5.44E-03 | 201.3 | 116.6 |
| ENSG00000140451.13 | *PIF1* | -2.0 | 6.55E-08 | 364.1 | 183.3 |
| ENSG00000011405.13 | *PIK3C2A* | 4.1 | 1.79E-07 | 35.6 | 145.0 |
| ENSG00000137193.14 | *PIM1* | 1.6 | 8.64E-15 | 860.2 | 1359.0 |
| ENSG00000198355.5 | *PIM3* | 1.6 | 6.70E-16 | 769.5 | 1257.3 |
| ENSG00000186111.9 | *PIP5K1C* | -1.9 | 3.50E-02 | 85.0 | 44.1 |
| ENSG00000164093.17 | *PITX2* | -2.1 | 7.49E-03 | 80.6 | 37.6 |
| ENSG00000244257.5 | *PKD1P1* | 2.4 | 3.85E-02 | 56.9 | 136.2 |
| ENSG00000105499.14 | *PLA2G4C* | -2.4 | 8.23E-04 | 114.1 | 47.4 |
| ENSG00000122861.16 | *PLAU* | 2.3 | 2.27E-42 | 654.6 | 1490.6 |
| ENSG00000011422.12 | *PLAUR* | 2.1 | 2.92E-15 | 304.0 | 646.2 |
| ENSG00000179598.6 | *PLD6* | 2.7 | 2.92E-09 | 72.8 | 196.1 |
| ENSG00000052126.14 | *PLEKHA5* | 1.5 | 2.54E-03 | 196.6 | 301.5 |
| ENSG00000173846.13 | *PLK3* | 3.7 | 2.25E-36 | 157.1 | 584.5 |
| ENSG00000130827.6 | *PLXNA3* | -1.8 | 9.90E-03 | 320.1 | 173.0 |
| ENSG00000004399.12 | *PLXND1* | -1.9 | 8.53E-03 | 127.1 | 66.1 |
| ENSG00000122512.16 | *PMS2* | 1.8 | 3.33E-03 | 126.4 | 232.2 |
| ENSG00000100941.9 | *PNN* | 1.8 | 3.49E-58 | 2201.5 | 4068.2 |
| ENSG00000146278.11 | *PNRC1* | -2.5 | 1.84E-18 | 533.7 | 212.0 |
| ENSG00000131626.18 | *PPFIA1* | 1.6 | 1.41E-02 | 112.9 | 178.5 |
| ENSG00000087074.8 | *PPP1R15A* | 1.9 | 1.16E-26 | 731.1 | 1361.1 |
| ENSG00000225361.4 | *PPP1R26-AS1* | -2.9 | 1.06E-02 | 42.8 | 14.6 |
| ENSG00000275342.5 | *PRAG1* | 2.4 | 1.45E-24 | 261.2 | 639.4 |
| ENSG00000106617.14 | *PRKAG2* | 1.8 | 9.83E-05 | 148.0 | 269.0 |
| ENSG00000204941.14 | *PSG5* | -1.5 | 4.74E-03 | 296.1 | 191.4 |
| ENSG00000185920.15 | *PTCH1* | -1.9 | 1.22E-02 | 284.1 | 153.3 |
| ENSG00000171522.6 | *PTGER4* | 1.6 | 3.62E-02 | 119.4 | 192.4 |
| ENSG00000073756.12 | *PTGS2* | 345.7 | 1.17E-09 | 0.2 | 63.2 |
| ENSG00000152894.14 | *PTPRK* | 2.0 | 2.37E-04 | 186.7 | 369.2 |
| ENSG00000146676.10 | *PURB* | 1.6 | 7.73E-16 | 1605.1 | 2491.9 |
| ENSG00000168994.13 | *PXDC1* | 4.7 | 8.02E-06 | 69.0 | 322.0 |
| ENSG00000171016.12 | *PYGO1* | 1.8 | 4.73E-02 | 74.4 | 133.2 |
| ENSG00000121350.16 | *PYROXD1* | 1.6 | 1.60E-02 | 192.0 | 311.7 |
| ENSG00000099246.16 | *RAB18* | -103.0 | 4.61E-05 | 41.0 | 0.4 |
| ENSG00000041353.10 | *RAB27B* | 2.3 | 3.09E-02 | 26.9 | 62.9 |
| ENSG00000188060.7 | *RAB42* | -148.5 | 2.09E-05 | 27.6 | 0.2 |
| ENSG00000113522.14 | *RAD50* | 1.5 | 1.53E-06 | 948.7 | 1455.0 |
| ENSG00000111247.14 | *RAD51AP1* | 1.7 | 5.95E-03 | 151.8 | 257.6 |
| ENSG00000153201.16 | *RANBP2* | 1.8 | 6.31E-09 | 1673.8 | 3083.8 |
| ENSG00000131759.18 | *RARA* | 2.1 | 3.05E-24 | 343.2 | 737.8 |
| ENSG00000123094.15 | *RASSF8* | 1.5 | 8.78E-03 | 383.6 | 581.7 |
| ENSG00000146587.18 | *RBAK* | 1.7 | 7.75E-07 | 281.8 | 491.5 |
| ENSG00000112183.15 | *RBM24* | 1.7 | 1.22E-02 | 96.5 | 161.5 |
| ENSG00000121039.10 | *RDH10* | -1.5 | 3.88E-08 | 2752.1 | 1778.0 |
| ENSG00000054967.13 | *RELT* | 1.7 | 6.67E-06 | 269.0 | 448.4 |
| ENSG00000182175.14 | *RGMA* | -3.9 | 2.30E-02 | 32.4 | 8.3 |
| ENSG00000143333.7 | *RGS16* | 2.1 | 1.58E-03 | 54.0 | 110.9 |
| ENSG00000116741.8 | *RGS2* | 1.7 | 3.17E-03 | 87.1 | 152.3 |
| ENSG00000138835.22 | *RGS3* | 2.3 | 2.62E-12 | 184.1 | 416.3 |
| ENSG00000143878.10 | *RHOB* | 2.0 | 2.36E-35 | 785.1 | 1542.5 |
| ENSG00000080345.18 | *RIF1* | 1.6 | 5.84E-06 | 1050.3 | 1629.0 |
| ENSG00000115963.13 | *RND3* | 1.6 | 2.00E-04 | 465.0 | 765.2 |
| ENSG00000179859.9 | *RNF227* | 13.7 | 1.66E-02 | 2.5 | 33.7 |
| ENSG00000127870.17 | *RNF6* | 1.7 | 4.86E-05 | 323.2 | 536.0 |
| ENSG00000166592.12 | *RRAD* | 2.7 | 1.14E-03 | 29.8 | 79.1 |
| ENSG00000048649.13 | *RSF1* | 1.7 | 5.73E-04 | 229.5 | 400.6 |
| ENSG00000159216.18 | *RUNX1* | 1.6 | 1.66E-05 | 530.0 | 839.6 |
| ENSG00000124813.23 | *RUNX2* | 1.5 | 2.52E-03 | 162.4 | 251.3 |
| ENSG00000213694.5 | *S1PR3* | -2.0 | 3.65E-03 | 106.8 | 53.3 |
| ENSG00000151835.16 | *SACS* | 1.6 | 1.35E-05 | 1499.6 | 2429.0 |
| ENSG00000205413.8 | *SAMD9* | 1.6 | 1.76E-03 | 345.7 | 544.7 |
| ENSG00000136715.18 | *SAP130* | 1.6 | 3.99E-02 | 341.9 | 555.5 |
| ENSG00000130066.16 | *SAT1* | 2.1 | 7.48E-06 | 92.3 | 192.6 |
| ENSG00000151967.18 | *SCHIP1* | 7.0 | 3.95E-06 | 22.2 | 154.8 |
| ENSG00000124145.6 | *SDC4* | 2.1 | ######## | 2173.7 | 4459.1 |
| ENSG00000091490.11 | *SEL1L3* | -1.7 | 3.85E-05 | 427.9 | 258.3 |
| ENSG00000138623.10 | *SEMA7A* | 2.1 | 3.59E-11 | 245.9 | 515.7 |
| ENSG00000122545.20 | *SEPTIN7* | 2.0 | 1.65E-03 | 322.3 | 633.5 |
| ENSG00000197019.5 | *SERTAD1* | 3.2 | 6.77E-19 | 130.9 | 414.5 |
| ENSG00000179833.4 | *SERTAD2* | 1.7 | 1.29E-13 | 563.7 | 950.7 |
| ENSG00000118515.11 | *SGK1* | 4.0 | 7.06E-29 | 137.1 | 554.6 |
| ENSG00000104205.15 | *SGK3* | 1.7 | 4.49E-02 | 90.4 | 152.7 |
| ENSG00000142178.9 | *SIK1* | 2.3 | 4.14E-16 | 230.3 | 535.9 |
| ENSG00000198053.11 | *SIRPA* | -1.5 | 4.10E-04 | 373.3 | 243.1 |
| ENSG00000180592.17 | *SKIDA1* | -2.7 | 1.16E-02 | 46.5 | 17.5 |
| ENSG00000136603.14 | *SKIL* | 2.4 | 3.68E-17 | 750.6 | 1824.6 |
| ENSG00000144136.11 | *SLC20A1* | 1.6 | 6.83E-15 | 1191.9 | 1870.4 |
| ENSG00000122912.15 | *SLC25A16* | 1.7 | 2.08E-03 | 133.5 | 221.6 |
| ENSG00000148339.12 | *SLC25A25* | 1.9 | 2.55E-14 | 425.9 | 793.5 |
| ENSG00000059804.16 | *SLC2A3* | 1.9 | 1.25E-03 | 118.8 | 227.5 |
| ENSG00000170385.10 | *SLC30A1* | 3.0 | 3.30E-58 | 564.2 | 1668.4 |
| ENSG00000184347.14 | *SLIT3* | -2.3 | 1.67E-03 | 116.3 | 50.7 |
| ENSG00000213599.10 | *SLX1A-SULT1A3* | -2.3 | 2.24E-04 | 146.9 | 64.0 |
| ENSG00000101665.9 | *SMAD7* | 3.2 | 2.70E-06 | 81.3 | 256.3 |
| ENSG00000088826.18 | *SMOX* | 1.8 | 5.01E-03 | 90.9 | 159.3 |
| ENSG00000183963.18 | *SMTN* | 1.7 | 3.69E-02 | 69.3 | 121.1 |
| ENSG00000124216.4 | *SNAI1* | 3.6 | 1.36E-15 | 87.2 | 311.2 |
| ENSG00000019549.13 | *SNAI2* | 1.9 | 1.49E-06 | 147.6 | 284.7 |
| ENSG00000232956.9 | *SNHG15* | 1.8 | 2.04E-02 | 185.6 | 329.8 |
| ENSG00000178996.14 | *SNX18* | 2.2 | 1.65E-18 | 260.5 | 578.3 |
| ENSG00000198142.5 | *SOWAHC* | 3.0 | 2.36E-59 | 429.0 | 1281.7 |
| ENSG00000124766.7 | *SOX4* | -1.6 | 7.76E-10 | 1934.7 | 1222.0 |
| ENSG00000125398.8 | *SOX9* | 3.1 | 2.71E-10 | 65.6 | 200.9 |
| ENSG00000104450.12 | *SPAG1* | 156.6 | 4.09E-05 | 0.2 | 27.8 |
| ENSG00000158792.16 | *SPATA2L* | 1.6 | 3.59E-02 | 86.8 | 137.4 |
| ENSG00000152582.14 | *SPEF2* | 1.9 | 2.49E-04 | 114.0 | 219.8 |
| ENSG00000176170.13 | *SPHK1* | 2.0 | 1.32E-04 | 141.6 | 285.0 |
| ENSG00000136158.12 | *SPRY2* | 2.7 | 7.45E-10 | 90.5 | 246.5 |
| ENSG00000187678.9 | *SPRY4* | 32.4 | 3.30E-06 | 1.7 | 55.0 |
| ENSG00000112658.8 | *SRF* | 1.7 | 4.34E-34 | 1799.5 | 3006.4 |
| ENSG00000100650.16 | *SRSF5* | 1.6 | 3.68E-17 | 3194.3 | 5125.4 |
| ENSG00000164211.13 | *STARD4* | 1.8 | 2.78E-06 | 383.7 | 679.9 |
| ENSG00000113739.10 | *STC2* | 2.3 | 9.25E-04 | 55.1 | 125.2 |
| ENSG00000094975.14 | *SUCO* | 1.6 | 9.25E-04 | 345.1 | 556.4 |
| ENSG00000097096.9 | *SYDE2* | 2.0 | 6.01E-04 | 115.1 | 226.2 |
| ENSG00000135801.9 | *TAF5L* | 1.8 | 9.03E-04 | 175.1 | 316.6 |
| ENSG00000133142.17 | *TCEAL4* | 1.6 | 1.62E-02 | 227.1 | 355.3 |
| ENSG00000113649.11 | *TCERG1* | 1.7 | 2.11E-07 | 464.5 | 769.1 |
| ENSG00000112773.16 | *TENT5A* | -1.5 | 6.68E-05 | 491.6 | 320.5 |
| ENSG00000105825.14 | *TFPI2* | 2.1 | 4.97E-03 | 43.6 | 92.0 |
| ENSG00000137801.11 | *THBS1* | 1.7 | 1.95E-77 | 24183.5 | 40484.6 |
| ENSG00000140534.14 | *TICRR* | -1.6 | 2.44E-14 | 933.2 | 567.1 |
| ENSG00000140993.11 | *TIGD7* | 2.2 | 2.14E-02 | 39.4 | 85.9 |
| ENSG00000163659.13 | *TIPARP* | 2.7 | ######## | 1474.9 | 4017.2 |
| ENSG00000249992.2 | *TMEM158* | 1.8 | 1.41E-07 | 246.4 | 432.6 |
| ENSG00000164484.11 | *TMEM200A* | 3.7 | 1.79E-05 | 58.2 | 213.9 |
| ENSG00000103978.16 | *TMEM87A* | 1.6 | 9.22E-03 | 273.9 | 450.8 |
| ENSG00000167920.10 | *TMEM99* | -1.7 | 3.56E-02 | 138.6 | 80.5 |
| ENSG00000118503.15 | *TNFAIP3* | 2.2 | 2.07E-04 | 70.6 | 157.0 |
| ENSG00000104689.10 | *TNFRSF10A* | 1.8 | 3.19E-05 | 241.1 | 434.0 |
| ENSG00000161955.16 | *TNFSF13* | -2.7 | 6.36E-03 | 54.2 | 19.9 |
| ENSG00000131746.13 | *TNS4* | 2.1 | 6.29E-03 | 44.8 | 93.7 |
| ENSG00000186815.12 | *TPCN1* | -1.7 | 4.53E-03 | 337.0 | 202.7 |
| ENSG00000047410.14 | *TPR* | 1.5 | 2.42E-08 | 2126.1 | 3281.4 |
| ENSG00000131323.14 | *TRAF3* | 1.7 | 3.91E-02 | 94.3 | 161.7 |
| ENSG00000076604.15 | *TRAF4* | 1.8 | 2.38E-16 | 675.5 | 1183.2 |
| ENSG00000173334.4 | *TRIB1* | 1.7 | 4.55E-05 | 176.1 | 306.3 |
| ENSG00000204977.10 | *TRIM13* | 1.7 | 3.17E-03 | 121.4 | 202.0 |
| ENSG00000108448.21 | *TRIM16L* | -2.1 | 2.00E-17 | 709.5 | 339.0 |
| ENSG00000153827.13 | *TRIP12* | 3.8 | 1.60E-06 | 40.5 | 153.6 |
| ENSG00000106025.9 | *TSPAN12* | -1.6 | 4.58E-02 | 136.2 | 86.0 |
| ENSG00000108423.15 | *TUBD1* | -2.2 | 4.10E-05 | 158.3 | 72.9 |
| ENSG00000143367.16 | *TUFT1* | 1.9 | 1.67E-40 | 1558.0 | 2977.1 |
| ENSG00000131002.12 | *TXLNGY* | 2.3 | 8.47E-04 | 120.1 | 276.4 |
| ENSG00000265972.6 | *TXNIP* | -1.7 | 4.14E-10 | 619.9 | 371.3 |
| ENSG00000137831.15 | *UACA* | 1.9 | 1.41E-10 | 1094.8 | 2122.8 |
| ENSG00000177169.10 | *ULK1* | -1.6 | 9.96E-06 | 574.4 | 360.0 |
| ENSG00000058056.9 | *USP13* | -1.7 | 1.88E-02 | 902.7 | 527.1 |
| ENSG00000170185.10 | *USP38* | 2.4 | 2.12E-02 | 53.5 | 126.0 |
| ENSG00000123552.17 | *USP45* | 1.9 | 2.25E-02 | 52.7 | 97.9 |
| ENSG00000145390.11 | *USP53* | 5.9 | 1.51E-08 | 28.8 | 168.9 |
| ENSG00000166348.18 | *USP54* | -1.7 | 1.28E-02 | 206.0 | 118.8 |
| ENSG00000168140.5 | *VASN* | 1.7 | 1.20E-43 | 1548.9 | 2700.0 |
| ENSG00000062716.13 | *VMP1* | 1.5 | 1.05E-29 | 2737.8 | 4168.9 |
| ENSG00000197969.14 | *VPS13A* | 1.7 | 6.20E-05 | 674.3 | 1166.8 |
| ENSG00000167716.18 | *WDR81* | -6.6 | 3.19E-02 | 32.2 | 4.9 |
| ENSG00000105875.14 | *WDR91* | -2.2 | 2.29E-02 | 99.9 | 45.2 |
| ENSG00000111186.13 | *WNT5B* | -1.7 | 2.01E-05 | 314.2 | 185.6 |
| ENSG00000134684.10 | *YARS* | 1.8 | 9.22E-03 | 84.8 | 156.0 |
| ENSG00000136758.18 | *YME1L1* | 1.8 | 1.48E-02 | 432.8 | 777.9 |
| ENSG00000196449.4 | *YRDC* | 1.8 | 2.19E-10 | 477.6 | 860.5 |
| ENSG00000221886.4 | *ZBED8* | -2.1 | 2.56E-02 | 98.7 | 47.7 |
| ENSG00000173276.14 | *ZBTB21* | 1.6 | 2.75E-10 | 472.3 | 770.0 |
| ENSG00000163874.11 | *ZC3H12A* | 4.1 | 2.76E-28 | 116.6 | 483.0 |
| ENSG00000149289.11 | *ZC3H12C* | 1.7 | 9.25E-04 | 236.7 | 408.3 |
| ENSG00000123200.16 | *ZC3H13* | 1.5 | 1.92E-06 | 662.5 | 1016.3 |
| ENSG00000128016.7 | *ZFP36* | 4.0 | 1.06E-25 | 97.2 | 386.4 |
| ENSG00000185650.9 | *ZFP36L1* | 5.9 | 1.73E-02 | 10.4 | 61.0 |
| ENSG00000152518.8 | *ZFP36L2* | 2.6 | 5.19E-70 | 782.4 | 2065.6 |
| ENSG00000165861.14 | *ZFYVE1* | -1.8 | 1.78E-02 | 225.2 | 124.1 |
| ENSG00000163867.17 | *ZMYM6* | 1.6 | 1.83E-03 | 181.9 | 298.7 |
| ENSG00000164631.19 | *ZNF12* | 1.7 | 3.63E-05 | 372.3 | 633.9 |
| ENSG00000197961.11 | *ZNF121* | 5.0 | 6.58E-07 | 51.1 | 253.6 |
| ENSG00000196418.12 | *ZNF124* | 1.9 | 2.26E-02 | 57.5 | 109.7 |
| ENSG00000213762.12 | *ZNF134* | 1.6 | 9.83E-03 | 149.0 | 237.9 |
| ENSG00000196646.12 | *ZNF136* | 2.1 | 8.07E-03 | 44.5 | 92.9 |
| ENSG00000170949.17 | *ZNF160* | 2.2 | 2.16E-02 | 82.3 | 183.5 |
| ENSG00000198105.14 | *ZNF248* | 2.1 | 4.40E-02 | 36.2 | 76.2 |
| ENSG00000185947.15 | *ZNF267* | 2.1 | 2.61E-05 | 91.1 | 193.9 |
| ENSG00000162702.8 | *ZNF281* | 1.7 | 1.72E-16 | 868.0 | 1438.1 |
| ENSG00000197044.11 | *ZNF441* | 2.4 | 9.37E-03 | 32.2 | 77.2 |
| ENSG00000198464.14 | *ZNF480* | 1.9 | 2.28E-02 | 114.3 | 217.4 |
| ENSG00000127081.14 | *ZNF484* | 1.7 | 3.34E-02 | 71.0 | 118.9 |
| ENSG00000188785.12 | *ZNF548* | 2.2 | 2.53E-04 | 66.4 | 144.5 |
| ENSG00000167981.7 | *ZNF597* | 1.8 | 5.95E-03 | 81.3 | 147.9 |
| ENSG00000166704.11 | *ZNF606* | -3.1 | 3.12E-02 | 29.3 | 9.4 |
| ENSG00000122482.21 | *ZNF644* | 1.5 | 4.68E-06 | 664.1 | 1007.7 |
| ENSG00000230844.3 | *ZNF674-AS1* | 2.2 | 2.78E-03 | 53.7 | 118.5 |
| ENSG00000171163.15 | *ZNF692* | -1.7 | 2.61E-03 | 294.9 | 177.6 |
| ENSG00000143067.5 | *ZNF697* | 2.0 | 5.06E-06 | 102.7 | 206.4 |
| ENSG00000196110.8 | *ZNF699* | 1.8 | 1.14E-02 | 66.8 | 122.9 |
| ENSG00000185252.19 | *ZNF74* | -1.6 | 4.15E-02 | 143.4 | 92.2 |
| ENSG00000196214.11 | *ZNF766* | 1.8 | 1.31E-02 | 136.5 | 241.7 |

**Supplementary Table 5. Differentially expressed genes in CGL1^FRA1Act^ cells 2 hours after serum stimulation relative to baseline.**

To characterize the early transcriptional response to serum stimulation in the context of FRA1 overexpression, RNA-sequencing was performed on CGL1^FRA1Act^ cells collected 2 hours after media change and compared to baseline (0-hour) conditions. DEGs were identified using a fold change (F.C.) threshold >1.5 or <–1.5 and an FDR-adjusted p-value (p-adj) < 0.05. A total of 550 DEGs were identified, including 352 upregulated and 198 downregulated genes. This dataset reflects the transcriptional program triggered by mitogenic signaling in FRA1-overexpressing CGL1 cells. The table includes gene names, fold changes, p-adj values, and transcript abundance (TPM), sorted alphabetically.

| **Gene ID** | **Gene Name** | **F.C.** | **p-adj.** | **Baseline** | **2h Post Serum** |
| --- | --- | --- | --- | --- | --- |
| ENSG00000285238.2 | *AC006064.6* | 3.6 | 6.45E-03 | 148.4 | 531.7 |
| ENSG00000260597.1 | *AC012531.1* | -4.4 | 4.51E-03 | 64.3 | 14.7 |
| ENSG00000226380.9 | *AC016831.1* | 4.5 | 2.71E-03 | 11.8 | 52.7 |
| ENSG00000267519.6 | *AC020916.1* | 2.8 | 1.70E-06 | 212.9 | 602.9 |
| ENSG00000280138.1 | *AC027290.2* | 3.5 | 1.21E-02 | 17.2 | 59.3 |
| ENSG00000249773.3 | *AC092647.5* | -97.3 | 1.45E-02 | 25.7 | 0.3 |
| ENSG00000282057.1 | *AC092807.3* | 3.6 | 4.42E-02 | 28.2 | 100.8 |
| ENSG00000226268.3 | *AC135977.1* | 2.5 | 4.18E-02 | 46.8 | 117.8 |
| ENSG00000183889.12 | *AC138969.1* | -1.6 | 1.91E-02 | 516.2 | 324.1 |
| ENSG00000130402.12 | *ACTN4* | -1.6 | 2.31E-02 | 7075.9 | 4431.7 |
| ENSG00000154736.6 | *ADAMTS5* | -2.5 | 3.48E-03 | 141.0 | 57.2 |
| ENSG00000148926.10 | *ADM* | 2.9 | 1.06E-05 | 512.5 | 1486.5 |
| ENSG00000128165.9 | *ADM2* | 2.3 | 4.10E-02 | 47.9 | 111.4 |
| ENSG00000092847.12 | *AGO1* | -2.0 | 2.82E-02 | 549.3 | 268.7 |
| ENSG00000131016.17 | *AKAP12* | 2.6 | 2.02E-30 | 2003.9 | 5282.5 |
| ENSG00000053371.12 | *AKR7A2* | 13.9 | 1.12E-02 | 4.3 | 59.3 |
| ENSG00000142208.16 | *AKT1* | 3.9 | 1.71E-02 | 205.0 | 806.3 |
| ENSG00000185839.3 | *AL035411.1* | 3.6 | 2.50E-02 | 169.2 | 611.7 |
| ENSG00000172339.10 | *ALG14* | 2.0 | 2.30E-03 | 138.6 | 282.3 |
| ENSG00000139211.6 | *AMIGO2* | 5.1 | 9.42E-04 | 51.9 | 263.8 |
| ENSG00000167772.12 | *ANGPTL4* | 15.1 | 4.21E-08 | 8.5 | 128.2 |
| ENSG00000164236.12 | *ANKRD33B* | -1.7 | 8.33E-04 | 799.4 | 476.6 |
| ENSG00000163516.14 | *ANKZF1* | -1.8 | 2.25E-02 | 283.2 | 157.1 |
| ENSG00000042753.11 | *AP2S1* | 5.0 | 1.22E-02 | 98.7 | 494.5 |
| ENSG00000198576.4 | *ARC* | 7.1 | 2.71E-02 | 3.7 | 26.2 |
| ENSG00000132254.12 | *ARFIP2* | 1.6 | 3.59E-02 | 164.0 | 265.9 |
| ENSG00000137135.18 | *ARHGEF39* | -1.9 | 3.90E-03 | 339.5 | 175.4 |
| ENSG00000049618.23 | *ARID1B* | -1.7 | 3.03E-02 | 557.6 | 332.3 |
| ENSG00000116017.11 | *ARID3A* | 2.9 | 1.13E-02 | 48.4 | 139.4 |
| ENSG00000054267.22 | *ARID4B* | -1.6 | 3.48E-03 | 790.5 | 498.8 |
| ENSG00000150347.16 | *ARID5B* | -2.5 | 3.36E-07 | 1483.4 | 603.7 |
| ENSG00000122644.13 | *ARL4A* | -1.9 | 2.88E-02 | 608.1 | 326.0 |
| ENSG00000140691.16 | *ARMC5* | 3.6 | 1.97E-02 | 37.1 | 133.4 |
| ENSG00000136950.13 | *ARPC5L* | 1.7 | 1.62E-02 | 580.3 | 1009.7 |
| ENSG00000088280.19 | *ASAP3* | -1.7 | 3.15E-02 | 311.5 | 178.4 |
| ENSG00000070669.17 | *ASNS* | 1.6 | 4.77E-06 | 1791.9 | 2950.0 |
| ENSG00000034533.11 | *ASTE1* | 4.5 | 2.95E-02 | 8.4 | 37.2 |
| ENSG00000162772.17 | *ATF3* | 3.5 | 3.20E-13 | 164.7 | 570.7 |
| ENSG00000123395.14 | *ATG101* | 5.0 | 1.26E-05 | 24.3 | 121.2 |
| ENSG00000168874.13 | *ATOH8* | -2.4 | 1.95E-03 | 240.1 | 101.8 |
| ENSG00000156273.16 | *BACH1* | 1.7 | 1.76E-02 | 540.7 | 915.8 |
| ENSG00000204463.12 | *BAG6* | -1.5 | 1.04E-02 | 2344.7 | 1532.3 |
| ENSG00000050820.17 | *BCAR1* | 1.7 | 1.41E-05 | 829.6 | 1388.8 |
| ENSG00000137936.18 | *BCAR3* | 1.8 | 1.30E-04 | 385.8 | 709.9 |
| ENSG00000142867.14 | *BCL10* | 2.2 | 2.69E-05 | 193.8 | 432.9 |
| ENSG00000153094.23 | *BCL2L11* | -2.6 | 3.80E-04 | 188.9 | 71.9 |
| ENSG00000069399.15 | *BCL3* | 2.0 | 4.91E-02 | 56.9 | 114.7 |
| ENSG00000236824.2 | *BCYRN1* | 1.9 | 4.07E-02 | 679.0 | 1265.0 |
| ENSG00000176697.19 | *BDNF* | 1.6 | 1.02E-03 | 571.1 | 931.5 |
| ENSG00000133169.6 | *BEX1* | 1.6 | 4.69E-02 | 1175.5 | 1924.7 |
| ENSG00000134107.5 | *BHLHE40* | 14.0 | 4.15E-75 | 75.4 | 1059.3 |
| ENSG00000063169.10 | *BICRA* | 8.3 | 2.30E-02 | 9.7 | 80.1 |
| ENSG00000104081.14 | *BMF* | -5.5 | 4.52E-05 | 106.6 | 19.3 |
| ENSG00000125845.7 | *BMP2* | -2.6 | 4.91E-02 | 80.8 | 31.5 |
| ENSG00000125378.16 | *BMP4* | -2.9 | 1.72E-23 | 1505.7 | 528.0 |
| ENSG00000171634.18 | *BPTF* | -1.6 | 1.61E-02 | 1689.8 | 1054.6 |
| ENSG00000169925.17 | *BRD3* | -1.7 | 7.22E-03 | 880.1 | 526.8 |
| ENSG00000112983.17 | *BRD8* | -2.0 | 3.03E-06 | 1473.5 | 748.8 |
| ENSG00000133243.10 | *BTBD2* | -1.6 | 1.05E-02 | 1238.6 | 779.2 |
| ENSG00000132640.15 | *BTBD3* | 1.9 | 3.40E-02 | 699.1 | 1340.7 |
| ENSG00000106245.11 | *BUD31* | 1.6 | 1.83E-02 | 956.6 | 1566.7 |
| ENSG00000082153.18 | *BZW1* | 1.9 | 3.17E-04 | 2156.7 | 4002.8 |
| ENSG00000160298.17 | *C21orf58* | -2.2 | 2.94E-03 | 180.4 | 82.2 |
| ENSG00000110619.17 | *CARS* | 1.6 | 4.03E-03 | 1072.4 | 1681.7 |
| ENSG00000274070.2 | *CASTOR2* | -2.0 | 3.25E-02 | 150.0 | 75.3 |
| ENSG00000147996.16 | *CBWD5* | 1.6 | 2.59E-03 | 433.6 | 707.5 |
| ENSG00000159873.10 | *CCDC117* | -1.6 | 2.26E-02 | 426.5 | 260.7 |
| ENSG00000154781.16 | *CCDC174* | 2.2 | 3.16E-02 | 129.9 | 290.7 |
| ENSG00000105321.14 | *CCDC9* | 1.8 | 1.05E-02 | 218.3 | 390.0 |
| ENSG00000118523.6 | *CCN2* | 5.9 | 2.28E-69 | 1371.9 | 8085.3 |
| ENSG00000162063.13 | *CCNF* | -1.9 | 6.09E-04 | 1044.5 | 548.8 |
| ENSG00000138764.15 | *CCNG2* | -2.1 | 4.91E-02 | 173.8 | 83.9 |
| ENSG00000146574.15 | *CCZ1B* | 6.6 | 2.88E-02 | 22.6 | 150.0 |
| ENSG00000112149.10 | *CD83* | 1.8 | 1.88E-02 | 117.9 | 214.4 |
| ENSG00000197622.13 | *CDC42SE1* | 1.6 | 2.33E-03 | 865.2 | 1426.3 |
| ENSG00000134690.11 | *CDCA8* | -1.7 | 3.62E-03 | 1698.7 | 996.4 |
| ENSG00000134058.12 | *CDK7* | 1.7 | 6.29E-04 | 427.1 | 736.8 |
| ENSG00000111276.11 | *CDKN1B* | -1.9 | 6.39E-06 | 1054.6 | 561.0 |
| ENSG00000147883.12 | *CDKN2B* | -1.6 | 4.91E-02 | 461.6 | 289.4 |
| ENSG00000172216.6 | *CEBPB* | 1.8 | 1.05E-02 | 385.1 | 693.2 |
| ENSG00000153879.9 | *CEBPG* | 1.6 | 5.24E-03 | 671.7 | 1055.8 |
| ENSG00000143126.8 | *CELSR2* | -1.8 | 1.73E-02 | 272.8 | 151.1 |
| ENSG00000115163.15 | *CENPA* | -1.8 | 9.34E-03 | 439.7 | 242.5 |
| ENSG00000166451.13 | *CENPN* | 1.8 | 1.12E-02 | 557.7 | 1031.0 |
| ENSG00000198707.16 | *CEP290* | -5.3 | 3.36E-02 | 105.3 | 20.0 |
| ENSG00000003402.20 | *CFLAR* | 2.0 | 4.87E-03 | 200.5 | 401.1 |
| ENSG00000164430.16 | *CGAS* | 1.6 | 3.78E-03 | 415.8 | 663.9 |
| ENSG00000128965.13 | *CHAC1* | 10.8 | 1.43E-39 | 47.2 | 511.3 |
| ENSG00000109220.11 | *CHIC2* | 2.1 | 2.31E-02 | 77.0 | 161.5 |
| ENSG00000122966.16 | *CIT* | -1.6 | 1.87E-02 | 1048.9 | 659.1 |
| ENSG00000179862.7 | *CITED4* | 4.2 | 1.33E-02 | 11.2 | 47.4 |
| ENSG00000115295.20 | *CLIP4* | -422.6 | 2.93E-06 | 112.2 | 0.3 |
| ENSG00000183723.12 | *CMTM4* | -2.6 | 3.43E-02 | 267.2 | 103.9 |
| ENSG00000153721.19 | *CNKSR3* | -1.8 | 5.60E-06 | 894.0 | 497.8 |
| ENSG00000114270.17 | *COL7A1* | 2.4 | 7.20E-03 | 144.8 | 351.4 |
| ENSG00000129083.12 | *COPB1* | 2.0 | 4.05E-02 | 237.6 | 465.7 |
| ENSG00000021826.16 | *CPS1* | 5.1 | 6.36E-03 | 10.9 | 55.6 |
| ENSG00000146592.17 | *CREB5* | 1.7 | 9.65E-03 | 215.7 | 369.2 |
| ENSG00000160741.17 | *CRTC2* | -1.9 | 3.12E-04 | 484.0 | 256.9 |
| ENSG00000121671.12 | *CRY2* | 1.9 | 4.64E-02 | 76.3 | 147.6 |
| ENSG00000144655.15 | *CSRNP1* | 4.8 | 4.98E-12 | 129.6 | 624.1 |
| ENSG00000159176.14 | *CSRP1* | 2.1 | 3.45E-09 | 1011.4 | 2080.8 |
| ENSG00000178971.16 | *CTC1* | -3.2 | 4.31E-05 | 211.7 | 65.9 |
| ENSG00000275496.4 | *CU633906.1* | -6.1 | 7.68E-04 | 116.3 | 19.2 |
| ENSG00000107562.16 | *CXCL12* | -4.1 | 1.75E-02 | 73.3 | 18.0 |
| ENSG00000169429.11 | *CXCL8* | 12.9 | 1.71E-02 | 1.6 | 21.2 |
| ENSG00000008256.16 | *CYTH3* | -1.5 | 3.68E-03 | 1848.9 | 1199.7 |
| ENSG00000222041.11 | *CYTOR* | 2.0 | 2.93E-06 | 492.2 | 973.2 |
| ENSG00000137992.14 | *DBT* | -1.9 | 1.07E-02 | 403.2 | 212.9 |
| ENSG00000129187.14 | *DCTD* | 1.6 | 1.75E-02 | 333.0 | 548.5 |
| ENSG00000188215.10 | *DCUN1D3* | 1.7 | 4.40E-02 | 114.0 | 196.3 |
| ENSG00000175197.12 | *DDIT3* | 3.4 | 2.93E-04 | 52.6 | 180.8 |
| ENSG00000168209.5 | *DDIT4* | 5.2 | 2.87E-32 | 382.8 | 1978.5 |
| ENSG00000108654.15 | *DDX5* | 1.6 | 1.24E-02 | 7503.8 | 11729.0 |
| ENSG00000107984.10 | *DKK1* | 1.8 | 3.06E-02 | 1532.2 | 2772.7 |
| ENSG00000164741.15 | *DLC1* | 2.4 | 3.76E-03 | 339.9 | 799.9 |
| ENSG00000115844.11 | *DLX2* | 2.2 | 5.44E-04 | 103.1 | 231.9 |
| ENSG00000176399.4 | *DMRTA1* | -2.6 | 1.96E-03 | 153.8 | 60.0 |
| ENSG00000128590.5 | *DNAJB9* | -2.4 | 4.32E-03 | 137.7 | 57.5 |
| ENSG00000088305.18 | *DNMT3B* | -2.6 | 1.43E-02 | 158.3 | 61.9 |
| ENSG00000133059.17 | *DSTYK* | -1.5 | 1.74E-02 | 586.9 | 379.7 |
| ENSG00000120129.6 | *DUSP1* | 1.7 | 1.47E-03 | 304.2 | 513.6 |
| ENSG00000158050.5 | *DUSP2* | 3.1 | 7.11E-09 | 93.3 | 291.9 |
| ENSG00000120875.9 | *DUSP4* | 7.0 | 8.52E-12 | 21.6 | 150.0 |
| ENSG00000138166.6 | *DUSP5* | 10.6 | 3.67E-32 | 36.8 | 388.6 |
| ENSG00000139318.8 | *DUSP6* | 4.9 | 6.38E-09 | 29.9 | 147.6 |
| ENSG00000271672.1 | *DUXAP8* | -75.1 | 1.05E-02 | 19.7 | 0.3 |
| ENSG00000116406.19 | *EDEM3* | -992.9 | 7.27E-08 | 264.3 | 0.3 |
| ENSG00000078401.7 | *EDN1* | 3.0 | 1.21E-12 | 155.0 | 468.7 |
| ENSG00000120738.8 | *EGR1* | 16.9 | 6.52E-84 | 104.4 | 1768.2 |
| ENSG00000179388.9 | *EGR3* | 46.2 | 1.09E-10 | 2.5 | 116.7 |
| ENSG00000204371.11 | *EHMT2* | -2.0 | 2.35E-02 | 619.5 | 313.7 |
| ENSG00000128692.8 | *EIF2S2P4* | 2.2 | 4.94E-02 | 140.2 | 305.9 |
| ENSG00000149100.13 | *EIF3M* | 1.6 | 7.47E-03 | 2661.6 | 4270.7 |
| ENSG00000126749.16 | *EMG1* | 1.7 | 1.25E-02 | 882.5 | 1494.9 |
| ENSG00000171617.14 | *ENC1* | 4.7 | 1.15E-04 | 17.7 | 83.8 |
| ENSG00000152223.15 | *EPG5* | -111.0 | 3.27E-03 | 30.1 | 0.3 |
| ENSG00000142627.13 | *EPHA2* | 2.4 | 4.61E-17 | 1841.1 | 4499.1 |
| ENSG00000261150.2 | *EPPK1* | 20368719.4 | 2.93E-06 | 0.0 | 277.8 |
| ENSG00000116285.13 | *ERRFI1* | 11.4 | 3.97E-70 | 846.6 | 9651.4 |
| ENSG00000105379.9 | *ETFB* | 6.6 | 7.92E-03 | 15.3 | 101.5 |
| ENSG00000107371.13 | *EXOSC3* | 1.9 | 1.16E-02 | 549.5 | 1028.0 |
| ENSG00000123737.12 | *EXOSC9* | 2.4 | 3.44E-03 | 203.1 | 478.6 |
| ENSG00000110723.12 | *EXPH5* | -2.1 | 3.05E-02 | 128.4 | 61.3 |
| ENSG00000164251.5 | *F2RL1* | 2.2 | 3.24E-07 | 303.4 | 655.1 |
| ENSG00000117525.14 | *F3* | 7.1 | 1.85E-87 | 907.7 | 6429.8 |
| ENSG00000035141.8 | *FAM136A* | 2.9 | 6.57E-04 | 55.5 | 161.7 |
| ENSG00000165669.14 | *FAM204A* | 1.6 | 4.00E-02 | 296.6 | 483.1 |
| ENSG00000196227.11 | *FAM217B* | -2.0 | 5.86E-03 | 264.8 | 135.7 |
| ENSG00000101447.15 | *FAM83D* | -1.8 | 7.53E-07 | 1832.8 | 1025.4 |
| ENSG00000188522.15 | *FAM83G* | 1.7 | 1.60E-04 | 555.1 | 916.5 |
| ENSG00000181544.15 | *FANCB* | 2.6 | 2.85E-02 | 46.7 | 120.2 |
| ENSG00000162458.13 | *FBLIM1* | 2.1 | 1.98E-02 | 206.4 | 424.8 |
| ENSG00000115641.19 | *FHL2* | 1.6 | 4.75E-04 | 2130.3 | 3310.9 |
| ENSG00000183386.10 | *FHL3* | 1.6 | 1.27E-02 | 385.1 | 605.3 |
| ENSG00000182263.14 | *FIGN* | -3.3 | 1.58E-03 | 228.9 | 69.7 |
| ENSG00000198468.9 | *FLVCR1-DT* | -56.3 | 3.19E-02 | 26.4 | 0.5 |
| ENSG00000170345.10 | *FOS* | 8.1 | 2.27E-05 | 6.5 | 52.9 |
| ENSG00000125740.14 | *FOSB* | 10.9 | 7.42E-14 | 11.5 | 125.6 |
| ENSG00000175592.9 | *FOSL1* | 2.5 | 1.71E-23 | 2149.3 | 5343.2 |
| ENSG00000054598.9 | *FOXC1* | -2.0 | 4.87E-04 | 602.7 | 304.4 |
| ENSG00000176692.8 | *FOXC2* | 4.9 | 5.74E-03 | 10.4 | 51.5 |
| ENSG00000251493.5 | *FOXD1* | 1.7 | 8.68E-03 | 185.6 | 316.8 |
| ENSG00000181274.7 | *FRAT2* | -2.2 | 3.48E-03 | 256.8 | 118.7 |
| ENSG00000139926.15 | *FRMD6* | 1.7 | 2.71E-02 | 450.2 | 763.9 |
| ENSG00000265817.3 | *FSBP* | 2.4 | 1.61E-02 | 39.2 | 93.1 |
| ENSG00000134363.12 | *FST* | 1.8 | 2.01E-02 | 606.2 | 1098.9 |
| ENSG00000070404.10 | *FSTL3* | 2.0 | 4.42E-02 | 1549.7 | 3123.4 |
| ENSG00000180340.7 | *FZD2* | -2.2 | 2.06E-05 | 1314.8 | 600.1 |
| ENSG00000116717.13 | *GADD45A* | 4.7 | 2.44E-14 | 476.0 | 2225.4 |
| ENSG00000099860.9 | *GADD45B* | 2.9 | 1.32E-08 | 968.2 | 2817.4 |
| ENSG00000180447.7 | *GAS1* | -2.4 | 1.80E-02 | 119.1 | 49.7 |
| ENSG00000143614.10 | *GATAD2B* | -1.6 | 6.97E-03 | 738.6 | 472.6 |
| ENSG00000130513.6 | *GDF15* | 17.8 | 3.25E-24 | 22.8 | 406.9 |
| ENSG00000164949.8 | *GEM* | 3.4 | 1.52E-03 | 30.8 | 104.4 |
| ENSG00000159921.16 | *GNE* | 4.1 | 4.53E-03 | 29.1 | 119.1 |
| ENSG00000092978.11 | *GPATCH2* | 1.7 | 4.72E-03 | 231.7 | 391.6 |
| ENSG00000089916.18 | *GPATCH2L* | 2.1 | 4.14E-04 | 582.0 | 1198.8 |
| ENSG00000164850.15 | *GPER1* | -1.8 | 2.30E-02 | 212.2 | 115.4 |
| ENSG00000181773.7 | *GPR3* | 4.1 | 1.08E-03 | 19.5 | 79.8 |
| ENSG00000164418.20 | *GRIK2* | -294.9 | 1.33E-05 | 78.4 | 0.3 |
| ENSG00000164284.15 | *GRPEL2* | 2.6 | 1.14E-11 | 476.1 | 1232.7 |
| ENSG00000165417.12 | *GTF2A1* | -1.6 | 2.26E-02 | 865.3 | 537.2 |
| ENSG00000107937.19 | *GTPBP4* | 1.7 | 3.39E-04 | 2195.0 | 3746.7 |
| ENSG00000075218.19 | *GTSE1* | -1.8 | 5.60E-06 | 932.6 | 518.7 |
| ENSG00000113070.8 | *HBEGF* | 5.1 | 8.87E-12 | 77.0 | 395.2 |
| ENSG00000105856.14 | *HBP1* | -1.9 | 1.54E-02 | 437.3 | 230.7 |
| ENSG00000276550.4 | *HERC2P2* | -1.6 | 1.78E-02 | 528.2 | 321.1 |
| ENSG00000180229.12 | *HERC2P3* | -2.1 | 3.77E-02 | 237.6 | 112.2 |
| ENSG00000122557.10 | *HERPUD2* | -2.0 | 2.36E-02 | 331.3 | 167.6 |
| ENSG00000114315.4 | *HES1* | 14.3 | 1.13E-33 | 21.7 | 310.8 |
| ENSG00000188290.10 | *HES4* | 3.5 | 1.45E-02 | 67.2 | 232.1 |
| ENSG00000177374.13 | *HIC1* | 2.4 | 3.85E-02 | 48.6 | 118.8 |
| ENSG00000156515.23 | *HK1* | 27.8 | 3.80E-02 | 12.1 | 337.3 |
| ENSG00000136630.13 | *HLX* | -2.2 | 3.57E-02 | 194.7 | 90.5 |
| ENSG00000149948.13 | *HMGA2* | 7.0 | 1.21E-03 | 8.3 | 58.2 |
| ENSG00000112972.15 | *HMGCS1* | 1.5 | 2.05E-02 | 1272.9 | 1968.8 |
| ENSG00000215492.6 | *HNRNPA1P7* | 2.0 | 4.75E-02 | 2920.6 | 5957.7 |
| ENSG00000214753.4 | *HNRNPUL2* | -1.5 | 6.06E-03 | 2079.5 | 1357.4 |
| ENSG00000106031.9 | *HOXA13* | -3.2 | 6.03E-04 | 113.5 | 35.5 |
| ENSG00000123388.4 | *HOXC11* | -1.7 | 1.98E-02 | 523.1 | 314.7 |
| ENSG00000123407.4 | *HOXC12* | -2.0 | 3.76E-03 | 282.4 | 141.4 |
| ENSG00000123364.5 | *HOXC13* | -1.8 | 9.40E-04 | 425.4 | 232.2 |
| ENSG00000284024.2 | *HSPA14* | 3.1 | 1.46E-05 | 65.0 | 199.2 |
| ENSG00000204389.10 | *HSPA1A* | -2.0 | 3.45E-07 | 4105.2 | 2101.9 |
| ENSG00000204388.7 | *HSPA1B* | -2.2 | 2.61E-12 | 3706.0 | 1685.0 |
| ENSG00000004776.13 | *HSPB6* | -1.8 | 3.62E-02 | 458.5 | 260.4 |
| ENSG00000086758.16 | *HUWE1* | -1.6 | 4.45E-05 | 6483.5 | 3969.9 |
| ENSG00000115738.10 | *ID2* | -2.3 | 3.76E-03 | 197.8 | 87.2 |
| ENSG00000160888.7 | *IER2* | 4.8 | 2.74E-13 | 561.5 | 2689.3 |
| ENSG00000137331.12 | *IER3* | 7.4 | 5.82E-63 | 215.3 | 1602.2 |
| ENSG00000162783.11 | *IER5* | 2.1 | 1.74E-02 | 436.6 | 900.7 |
| ENSG00000188483.8 | *IER5L* | 1.8 | 1.27E-03 | 232.8 | 421.0 |
| ENSG00000169991.11 | *IFFO2* | 4.1 | 5.37E-08 | 235.2 | 973.5 |
| ENSG00000006652.14 | *IFRD1* | 1.8 | 2.95E-05 | 493.5 | 881.0 |
| ENSG00000214706.10 | *IFRD2* | 3.7 | 1.92E-02 | 178.6 | 654.0 |
| ENSG00000073792.15 | *IGF2BP2* | -1.7 | 1.07E-02 | 1197.4 | 725.2 |
| ENSG00000095752.7 | *IL11* | 48.7 | 1.63E-04 | 0.8 | 39.5 |
| ENSG00000150782.12 | *IL18* | 1.6 | 3.04E-03 | 383.1 | 606.5 |
| ENSG00000149503.13 | *INCENP* | -1.6 | 3.07E-03 | 1397.5 | 887.2 |
| ENSG00000122641.11 | *INHBA* | 10.2 | 2.53E-02 | 1.9 | 19.9 |
| ENSG00000186480.13 | *INSIG1* | 1.7 | 1.60E-05 | 987.9 | 1630.2 |
| ENSG00000176095.12 | *IP6K1* | -1.6 | 3.88E-02 | 954.3 | 594.3 |
| ENSG00000183856.11 | *IQGAP3* | -1.5 | 2.21E-03 | 1844.5 | 1201.7 |
| ENSG00000119669.5 | *IRF2BPL* | 2.1 | 1.01E-06 | 433.1 | 911.1 |
| ENSG00000185950.9 | *IRS2* | -1.8 | 3.62E-03 | 456.1 | 258.4 |
| ENSG00000078747.15 | *ITCH* | -3.0 | 3.03E-02 | 112.4 | 37.0 |
| ENSG00000005884.18 | *ITGA3* | 2.6 | 1.02E-02 | 1345.9 | 3463.4 |
| ENSG00000161638.11 | *ITGA5* | 2.1 | 3.23E-09 | 868.7 | 1787.3 |
| ENSG00000148841.17 | *ITPRIP* | 2.2 | 1.56E-06 | 276.3 | 617.4 |
| ENSG00000205726.15 | *ITSN1* | -1.6 | 5.18E-03 | 807.6 | 495.9 |
| ENSG00000152409.9 | *JMY* | -2.0 | 1.50E-03 | 543.9 | 278.0 |
| ENSG00000225470.8 | *JPX* | 1.7 | 3.84E-02 | 223.2 | 382.6 |
| ENSG00000177606.7 | *JUN* | 3.0 | 6.12E-11 | 299.8 | 886.2 |
| ENSG00000171223.6 | *JUNB* | 9.0 | 1.46E-74 | 191.5 | 1714.6 |
| ENSG00000130522.6 | *JUND* | 1.5 | 1.24E-02 | 953.3 | 1447.1 |
| ENSG00000114982.19 | *KANSL3* | -1.5 | 6.05E-03 | 752.4 | 488.9 |
| ENSG00000156650.14 | *KAT6B* | -1.6 | 4.08E-02 | 721.7 | 447.0 |
| ENSG00000170852.11 | *KBTBD2* | 1.6 | 1.72E-03 | 719.1 | 1114.7 |
| ENSG00000120696.9 | *KBTBD7* | -2.5 | 1.67E-02 | 128.8 | 51.2 |
| ENSG00000156113.23 | *KCNMA1* | 2.9 | 4.94E-02 | 56.4 | 165.9 |
| ENSG00000153885.14 | *KCTD15* | 1.9 | 4.42E-02 | 85.6 | 163.5 |
| ENSG00000188997.8 | *KCTD21* | -3.9 | 6.05E-03 | 87.6 | 22.3 |
| ENSG00000126012.11 | *KDM5C* | -1.6 | 1.09E-03 | 1432.1 | 923.0 |
| ENSG00000162522.11 | *KIAA1522* | -1.6 | 2.07E-02 | 722.1 | 464.2 |
| ENSG00000186185.14 | *KIF18B* | -1.6 | 3.24E-03 | 1056.4 | 677.8 |
| ENSG00000155090.15 | *KLF10* | 4.5 | 7.26E-20 | 410.8 | 1860.5 |
| ENSG00000129911.9 | *KLF16* | 1.8 | 2.49E-02 | 619.6 | 1095.2 |
| ENSG00000136826.15 | *KLF4* | 3.4 | 5.72E-17 | 156.3 | 533.1 |
| ENSG00000067082.15 | *KLF6* | 2.6 | 4.19E-03 | 1100.8 | 2808.7 |
| ENSG00000099910.17 | *KLHL22* | -2.8 | 2.42E-03 | 113.7 | 40.7 |
| ENSG00000114796.16 | *KLHL24* | -2.2 | 2.94E-02 | 267.0 | 123.1 |
| ENSG00000212724.3 | *KRTAP2-3* | 6.1 | 1.63E-03 | 9.5 | 57.8 |
| ENSG00000198841.4 | *KTI12* | 1.6 | 4.43E-02 | 251.1 | 413.3 |
| ENSG00000188186.10 | *LAMTOR4* | 2.7 | 9.34E-03 | 68.9 | 184.0 |
| ENSG00000213626.13 | *LBH* | 2.1 | 2.30E-03 | 103.2 | 211.5 |
| ENSG00000106003.13 | *LFNG* | 4.1 | 3.37E-10 | 57.0 | 235.2 |
| ENSG00000139292.13 | *LGR5* | -2.7 | 3.70E-02 | 67.6 | 25.3 |
| ENSG00000128342.5 | *LIF* | 11.2 | 5.37E-52 | 84.6 | 950.3 |
| ENSG00000169756.16 | *LIMS1* | 24.4 | 1.32E-02 | 3.1 | 75.2 |
| ENSG00000072163.19 | *LIMS2* | 12.3 | 9.97E-03 | 1.8 | 22.4 |
| ENSG00000183814.15 | *LIN9* | -2.6 | 6.92E-04 | 157.9 | 60.5 |
| ENSG00000228794.9 | *LINC01128* | 1.9 | 1.51E-02 | 138.0 | 268.2 |
| ENSG00000226053.1 | *LINC01776* | 3.2 | 3.06E-02 | 19.3 | 62.6 |
| ENSG00000185621.11 | *LMLN* | 3.3 | 4.80E-02 | 23.4 | 76.6 |
| ENSG00000136153.20 | *LMO7* | -1.5 | 1.38E-02 | 1795.5 | 1180.1 |
| ENSG00000154359.13 | *LONRF1* | 2.1 | 2.30E-04 | 133.5 | 281.7 |
| ENSG00000160959.8 | *LRRC14* | -1.6 | 1.76E-02 | 706.4 | 439.3 |
| ENSG00000136802.11 | *LRRC8A* | 8.8 | 3.53E-02 | 24.4 | 214.2 |
| ENSG00000143429.10 | *LSP1P4* | -6.2 | 2.99E-02 | 30.2 | 4.8 |
| ENSG00000185022.12 | *MAFF* | 7.7 | 1.80E-20 | 57.4 | 441.7 |
| ENSG00000198517.10 | *MAFK* | 2.1 | 1.12E-04 | 575.8 | 1228.9 |
| ENSG00000198042.11 | *MAK16* | 1.6 | 1.63E-03 | 559.8 | 900.2 |
| ENSG00000013619.14 | *MAMLD1* | 4.8 | 3.93E-02 | 20.2 | 97.0 |
| ENSG00000034152.19 | *MAP2K3* | 2.3 | 1.05E-10 | 632.3 | 1464.4 |
| ENSG00000006062.17 | *MAP3K14* | 2.1 | 4.20E-09 | 470.4 | 1000.3 |
| ENSG00000047849.21 | *MAP4* | -1.7 | 3.60E-02 | 3521.4 | 2084.1 |
| ENSG00000180611.7 | *MB21D2* | 3.5 | 1.39E-20 | 194.1 | 671.7 |
| ENSG00000143384.13 | *MCL1* | 2.9 | 1.14E-11 | 2653.3 | 7703.0 |
| ENSG00000133398.4 | *MED10* | 1.9 | 9.68E-03 | 675.9 | 1263.1 |
| ENSG00000254726.3 | *MEX3A* | -2.0 | 3.80E-05 | 451.8 | 231.1 |
| ENSG00000174197.16 | *MGA* | -17.6 | 1.03E-05 | 74.1 | 4.2 |
| ENSG00000074416.14 | *MGLL* | 109.9 | 1.67E-04 | 0.5 | 58.2 |
| ENSG00000172965.16 | *MIR4435-2HG* | 2.2 | 6.44E-04 | 535.1 | 1151.6 |
| ENSG00000148773.14 | *MKI67* | -2.2 | 4.47E-02 | 11186.7 | 5176.2 |
| ENSG00000076242.14 | *MLH1* | -83.7 | 1.24E-02 | 22.1 | 0.3 |
| ENSG00000122687.18 | *MRM2* | 3.1 | 1.21E-06 | 244.7 | 750.7 |
| ENSG00000188895.12 | *MSL1* | -1.6 | 3.03E-04 | 1252.5 | 783.0 |
| ENSG00000052802.13 | *MSMO1* | 1.5 | 3.68E-03 | 579.6 | 889.1 |
| ENSG00000169715.15 | *MT1E* | 1.6 | 9.12E-04 | 1235.4 | 1967.3 |
| ENSG00000187193.9 | *MT1X* | 2.3 | 4.78E-03 | 116.5 | 263.0 |
| ENSG00000125148.7 | *MT2A* | 1.8 | 7.40E-09 | 1273.0 | 2298.9 |
| ENSG00000168502.17 | *MTCL1* | 3.2 | 4.25E-02 | 67.3 | 215.8 |
| ENSG00000065911.12 | *MTHFD2* | 1.6 | 2.54E-03 | 974.4 | 1511.7 |
| ENSG00000198695.2 | *MT-ND6* | -1.8 | 1.54E-04 | 828.7 | 462.2 |
| ENSG00000179820.16 | *MYADM* | 2.0 | 6.76E-06 | 781.7 | 1548.7 |
| ENSG00000172936.14 | *MYD88* | 6.7 | 8.43E-03 | 19.1 | 127.3 |
| ENSG00000101608.12 | *MYL12A* | 2.6 | 7.42E-04 | 83.7 | 220.7 |
| ENSG00000118680.13 | *MYL12B* | 18.8 | 3.71E-06 | 14.6 | 273.2 |
| ENSG00000092841.18 | *MYL6* | 1.6 | 3.50E-02 | 3665.8 | 5796.3 |
| ENSG00000007944.15 | *MYLIP* | -3.2 | 2.93E-03 | 91.2 | 28.2 |
| ENSG00000176182.6 | *MYPOP* | -2.4 | 2.73E-02 | 91.8 | 38.5 |
| ENSG00000164134.13 | *NAA15* | 2.4 | 5.80E-04 | 344.7 | 813.2 |
| ENSG00000138386.17 | *NAB1* | 1.6 | 3.14E-02 | 280.9 | 452.7 |
| ENSG00000166886.13 | *NAB2* | 7.6 | 4.12E-63 | 190.1 | 1449.0 |
| ENSG00000160796.17 | *NBEAL2* | -1.6 | 4.42E-02 | 381.5 | 243.3 |
| ENSG00000115053.16 | *NCL* | 92.2 | 7.09E-03 | 19.4 | 1792.1 |
| ENSG00000140396.13 | *NCOA2* | -1.8 | 2.76E-02 | 367.4 | 199.0 |
| ENSG00000198646.14 | *NCOA6* | -1.8 | 1.72E-03 | 904.2 | 510.4 |
| ENSG00000125356.7 | *NDUFA1* | 1.5 | 4.96E-02 | 578.2 | 890.2 |
| ENSG00000186010.19 | *NDUFA13* | 1.6 | 3.36E-02 | 721.9 | 1181.2 |
| ENSG00000189043.10 | *NDUFA4* | 2.1 | 3.01E-02 | 776.5 | 1594.0 |
| ENSG00000151366.13 | *NDUFC2* | 1.9 | 4.86E-02 | 902.2 | 1716.7 |
| ENSG00000111859.17 | *NEDD9* | 2.7 | 6.10E-05 | 281.5 | 764.7 |
| ENSG00000214357.9 | *NEURL1B* | -1.6 | 4.03E-03 | 476.7 | 291.4 |
| ENSG00000141905.19 | *NFIC* | -1.6 | 4.14E-04 | 3189.6 | 2014.2 |
| ENSG00000008441.16 | *NFIX* | -1.7 | 5.04E-06 | 1200.6 | 692.2 |
| ENSG00000169599.12 | *NFU1* | 12.7 | 1.05E-02 | 5.8 | 74.0 |
| ENSG00000132603.15 | *NIP7* | 1.6 | 5.74E-03 | 1353.8 | 2222.7 |
| ENSG00000136448.12 | *NMT1* | -1.6 | 1.31E-03 | 1127.4 | 712.8 |
| ENSG00000166741.7 | *NNMT* | 2.0 | 3.96E-02 | 138.7 | 271.3 |
| ENSG00000264343.6 | *NOTCH2NLA* | -3.5 | 3.13E-02 | 79.4 | 22.8 |
| ENSG00000249353.2 | *NPM1P27* | 3.2 | 8.80E-03 | 3121.1 | 10091.0 |
| ENSG00000184162.14 | *NR2C2AP* | 2.4 | 4.15E-02 | 53.7 | 130.7 |
| ENSG00000123358.20 | *NR4A1* | 3.9 | 5.91E-14 | 98.1 | 384.8 |
| ENSG00000111696.12 | *NT5DC3* | 1.6 | 4.18E-02 | 299.5 | 483.7 |
| ENSG00000135318.12 | *NT5E* | 1.6 | 1.72E-03 | 453.1 | 744.5 |
| ENSG00000163545.10 | *NUAK2* | 3.5 | 1.63E-04 | 39.2 | 138.7 |
| ENSG00000132182.12 | *NUP210* | -1.5 | 2.95E-02 | 2754.5 | 1803.8 |
| ENSG00000110713.17 | *NUP98* | -3.1 | 7.68E-04 | 2090.4 | 672.0 |
| ENSG00000235636.1 | *NUS1P1* | 2.6 | 3.56E-05 | 87.8 | 230.5 |
| ENSG00000157181.16 | *ODR4* | 2.2 | 3.15E-02 | 195.5 | 429.3 |
| ENSG00000164920.9 | *OSR2* | -9.5 | 2.39E-03 | 72.5 | 7.6 |
| ENSG00000142623.11 | *PADI1* | 7.6 | 7.74E-03 | 4.1 | 30.8 |
| ENSG00000189184.11 | *PCDH18* | -3.3 | 6.44E-06 | 275.6 | 82.9 |
| ENSG00000169851.15 | *PCDH7* | 3.1 | 4.76E-07 | 76.1 | 238.4 |
| ENSG00000135749.19 | *PCNX2* | -1.8 | 2.10E-02 | 219.1 | 119.4 |
| ENSG00000100311.17 | *PDGFB* | 4.3 | 7.94E-07 | 44.5 | 191.9 |
| ENSG00000113721.14 | *PDGFRB* | -1.7 | 2.95E-03 | 783.7 | 456.8 |
| ENSG00000004799.8 | *PDK4* | -3.3 | 1.46E-02 | 82.2 | 24.7 |
| ENSG00000164951.16 | *PDP1* | 2.2 | 7.54E-05 | 191.0 | 428.4 |
| ENSG00000143256.5 | *PFDN2* | 1.6 | 1.69E-03 | 1038.8 | 1627.4 |
| ENSG00000170525.21 | *PFKFB3* | 1.9 | 2.47E-03 | 994.0 | 1875.7 |
| ENSG00000137338.5 | *PGBD1* | -10.2 | 1.18E-03 | 84.5 | 8.3 |
| ENSG00000204138.13 | *PHACTR4* | -1.6 | 3.76E-03 | 1167.8 | 746.0 |
| ENSG00000134686.18 | *PHC2* | 1.6 | 1.65E-03 | 812.3 | 1293.0 |
| ENSG00000092621.12 | *PHGDH* | 3.1 | 4.15E-02 | 407.5 | 1275.6 |
| ENSG00000139289.13 | *PHLDA1* | 7.0 | 1.17E-49 | 223.6 | 1561.1 |
| ENSG00000181649.8 | *PHLDA2* | 1.5 | 7.58E-03 | 524.4 | 800.7 |
| ENSG00000144824.21 | *PHLDB2* | 2.1 | 2.01E-08 | 504.0 | 1054.1 |
| ENSG00000033800.13 | *PIAS1* | -2.8 | 1.46E-03 | 275.5 | 99.0 |
| ENSG00000131788.16 | *PIAS3* | -1.6 | 1.69E-02 | 491.9 | 298.3 |
| ENSG00000140451.13 | *PIF1* | -2.1 | 3.06E-02 | 374.5 | 182.5 |
| ENSG00000090975.12 | *PITPNM2* | -13.2 | 1.42E-02 | 49.5 | 3.7 |
| ENSG00000122861.16 | *PLAU* | 3.5 | 4.56E-12 | 140.9 | 495.8 |
| ENSG00000011422.12 | *PLAUR* | 3.6 | 7.28E-05 | 120.2 | 436.1 |
| ENSG00000068137.15 | *PLEKHH3* | 1.8 | 1.12E-02 | 186.7 | 335.7 |
| ENSG00000173846.13 | *PLK3* | 5.6 | 2.23E-13 | 66.2 | 371.5 |
| ENSG00000105520.10 | *PLPPR2* | -2.6 | 2.55E-02 | 100.6 | 39.3 |
| ENSG00000004399.12 | *PLXND1* | -2.7 | 2.99E-02 | 64.4 | 23.6 |
| ENSG00000124225.16 | *PMEPA1* | 1.8 | 2.76E-03 | 453.4 | 809.2 |
| ENSG00000138035.15 | *PNPT1* | 1.8 | 3.35E-02 | 839.3 | 1544.9 |
| ENSG00000146278.11 | *PNRC1* | -3.0 | 2.10E-09 | 412.5 | 137.0 |
| ENSG00000101868.11 | *POLA1* | -8.2 | 1.39E-03 | 424.1 | 51.8 |
| ENSG00000171453.20 | *POLR1C* | 1.9 | 2.76E-03 | 507.8 | 973.3 |
| ENSG00000155846.17 | *PPARGC1B* | -1.6 | 3.27E-02 | 355.6 | 227.8 |
| ENSG00000198618.5 | *PPIAP22* | 2.1 | 3.43E-02 | 642.6 | 1327.5 |
| ENSG00000087074.8 | *PPP1R15A* | 2.3 | 5.11E-04 | 703.2 | 1625.9 |
| ENSG00000146112.12 | *PPP1R18* | 1.5 | 1.74E-02 | 726.9 | 1116.6 |
| ENSG00000196422.11 | *PPP1R26* | -1.6 | 7.74E-03 | 491.3 | 312.0 |
| ENSG00000173281.5 | *PPP1R3B* | 2.6 | 3.55E-02 | 26.8 | 70.3 |
| ENSG00000275342.5 | *PRAG1* | 3.0 | 2.92E-05 | 84.4 | 249.3 |
| ENSG00000139174.12 | *PRICKLE1* | -3.1 | 4.31E-05 | 277.4 | 88.5 |
| ENSG00000126464.14 | *PRR12* | -1.7 | 2.85E-04 | 713.4 | 421.9 |
| ENSG00000204469.13 | *PRRC2A* | -1.5 | 1.89E-02 | 6725.6 | 4341.9 |
| ENSG00000095261.14 | *PSMD5* | 1.7 | 5.85E-06 | 761.0 | 1325.8 |
| ENSG00000185920.15 | *PTCH1* | -2.4 | 3.07E-02 | 326.3 | 138.6 |
| ENSG00000187514.16 | *PTMA* | 21.4 | 4.67E-02 | 196.3 | 4194.3 |
| ENSG00000134644.15 | *PUM1* | -1.8 | 2.04E-03 | 1264.9 | 693.7 |
| ENSG00000080608.10 | *PUM3* | 1.6 | 8.27E-05 | 1113.8 | 1732.7 |
| ENSG00000099246.16 | *RAB18* | -150.8 | 1.21E-02 | 39.8 | 0.3 |
| ENSG00000111540.16 | *RAB5B* | -2.0 | 1.57E-04 | 1381.2 | 705.3 |
| ENSG00000132341.12 | *RAN* | 1.7 | 2.95E-02 | 8547.7 | 14592.4 |
| ENSG00000204764.14 | *RANBP17* | 3.6 | 2.14E-03 | 21.4 | 77.7 |
| ENSG00000109756.9 | *RAPGEF2* | 2.0 | 7.92E-03 | 149.6 | 291.9 |
| ENSG00000131759.18 | *RARA* | 2.1 | 4.77E-06 | 221.1 | 463.0 |
| ENSG00000265241.6 | *RBM8A* | 1.9 | 4.91E-02 | 1598.1 | 3053.3 |
| ENSG00000104856.14 | *RELB* | 2.3 | 1.71E-02 | 65.8 | 149.2 |
| ENSG00000054967.13 | *RELT* | 1.8 | 1.12E-02 | 228.9 | 409.1 |
| ENSG00000144567.11 | *RETREG2* | -1.6 | 5.56E-03 | 742.0 | 475.3 |
| ENSG00000141699.11 | *RETREG3* | -1.7 | 4.91E-02 | 313.1 | 188.8 |
| ENSG00000009413.15 | *REV3L* | -5.8 | 3.13E-02 | 189.5 | 32.8 |
| ENSG00000163918.10 | *RFC4* | 1.9 | 1.24E-02 | 196.7 | 368.6 |
| ENSG00000168411.14 | *RFWD3* | 1.6 | 2.71E-02 | 1680.9 | 2669.4 |
| ENSG00000015568.13 | *RGPD5* | 2.6 | 6.34E-05 | 109.1 | 286.3 |
| ENSG00000143333.7 | *RGS16* | 2.5 | 7.63E-03 | 55.0 | 136.3 |
| ENSG00000116741.8 | *RGS2* | 4.1 | 2.88E-13 | 61.6 | 255.3 |
| ENSG00000138835.22 | *RGS3* | 1.7 | 4.89E-02 | 144.5 | 249.3 |
| ENSG00000143878.10 | *RHOB* | 1.6 | 1.60E-03 | 575.9 | 932.2 |
| ENSG00000172922.9 | *RNASEH2C* | 1.8 | 4.62E-02 | 350.9 | 622.6 |
| ENSG00000146083.12 | *RNF44* | -1.5 | 2.21E-03 | 828.1 | 540.8 |
| ENSG00000125995.16 | *ROMO1* | 1.9 | 3.93E-02 | 416.8 | 783.8 |
| ENSG00000265681.7 | *RPL17* | 2.6 | 3.07E-02 | 1035.3 | 2675.2 |
| ENSG00000232573.1 | *RPL3P4* | 2.9 | 3.45E-02 | 493.6 | 1410.4 |
| ENSG00000229638.1 | *RPL4P4* | 5.0 | 2.92E-02 | 18.9 | 93.5 |
| ENSG00000164587.13 | *RPS14* | 1.8 | 3.48E-02 | 13946.1 | 25188.0 |
| ENSG00000008988.9 | *RPS20* | 1.7 | 3.98E-02 | 121.5 | 205.0 |
| ENSG00000186468.13 | *RPS23* | 1.6 | 5.16E-03 | 11018.4 | 17199.6 |
| ENSG00000212994.5 | *RPS26P6* | 11.2 | 3.55E-02 | 2.3 | 25.5 |
| ENSG00000214389.2 | *RPS3AP26* | 2.1 | 1.91E-02 | 150.7 | 322.6 |
| ENSG00000081019.13 | *RSBN1* | -1.6 | 3.38E-02 | 265.4 | 162.6 |
| ENSG00000215012.9 | *RTL10* | 1.7 | 4.70E-03 | 339.8 | 563.5 |
| ENSG00000177409.12 | *SAMD9L* | -95.0 | 3.67E-03 | 25.0 | 0.3 |
| ENSG00000130066.16 | *SAT1* | 3.5 | 3.85E-05 | 41.8 | 145.0 |
| ENSG00000133028.12 | *SCO1* | 2.1 | 3.45E-02 | 958.1 | 2049.1 |
| ENSG00000124145.6 | *SDC4* | 3.0 | 2.43E-10 | 799.9 | 2393.1 |
| ENSG00000107651.13 | *SEC23IP* | 2.9 | 4.99E-02 | 30.6 | 89.2 |
| ENSG00000211450.10 | *SELENOH* | 2.2 | 4.69E-02 | 543.8 | 1217.8 |
| ENSG00000162430.17 | *SELENON* | -1.6 | 2.92E-02 | 1650.5 | 1009.6 |
| ENSG00000138623.10 | *SEMA7A* | 2.9 | 3.08E-07 | 233.1 | 678.1 |
| ENSG00000140264.19 | *SERF2* | 1.7 | 4.80E-02 | 3304.8 | 5552.9 |
| ENSG00000166401.15 | *SERPINB8* | 2.6 | 3.65E-04 | 78.3 | 205.1 |
| ENSG00000135919.13 | *SERPINE2* | 4.9 | 1.03E-02 | 11.3 | 55.2 |
| ENSG00000197019.5 | *SERTAD1* | 6.1 | 3.23E-14 | 70.9 | 431.4 |
| ENSG00000130766.5 | *SESN2* | 3.7 | 3.30E-21 | 194.7 | 727.2 |
| ENSG00000198879.11 | *SFMBT2* | -3805312.9 | 6.90E-05 | 81.2 | 0.0 |
| ENSG00000118515.11 | *SGK1* | 4.0 | 9.89E-03 | 147.7 | 592.6 |
| ENSG00000154447.15 | *SH3RF1* | 1.9 | 1.31E-03 | 202.1 | 385.7 |
| ENSG00000125089.17 | *SH3TC1* | 30.3 | 4.60E-03 | 0.7 | 19.9 |
| ENSG00000107338.10 | *SHB* | 1.6 | 1.71E-02 | 322.8 | 502.3 |
| ENSG00000116991.10 | *SIPA1L2* | -2048917.8 | 1.68E-04 | 42.0 | 0.0 |
| ENSG00000180592.17 | *SKIDA1* | -15.0 | 8.99E-06 | 63.2 | 4.2 |
| ENSG00000136603.14 | *SKIL* | 1.9 | 5.30E-04 | 570.4 | 1075.6 |
| ENSG00000141526.16 | *SLC16A3* | 2.1 | 1.05E-02 | 205.7 | 423.4 |
| ENSG00000144136.11 | *SLC20A1* | 2.0 | 7.05E-09 | 1030.1 | 2025.2 |
| ENSG00000148339.12 | *SLC25A25* | 1.8 | 2.96E-04 | 342.2 | 630.9 |
| ENSG00000164933.12 | *SLC25A32* | 1.9 | 5.75E-03 | 330.0 | 631.8 |
| ENSG00000170385.10 | *SLC30A1* | 2.3 | 8.33E-15 | 530.1 | 1221.6 |
| ENSG00000189339.12 | *SLC35E2B* | -1.7 | 4.31E-05 | 1000.8 | 580.9 |
| ENSG00000198743.7 | *SLC5A3* | -1.9 | 2.91E-05 | 1383.4 | 720.1 |
| ENSG00000089060.11 | *SLC8B1* | 4.5 | 3.51E-02 | 21.5 | 97.3 |
| ENSG00000166750.10 | *SLFN5* | 1.8 | 2.25E-02 | 129.9 | 238.9 |
| ENSG00000165300.7 | *SLITRK5* | -2.9 | 2.93E-03 | 281.0 | 96.5 |
| ENSG00000138375.12 | *SMARCAL1* | -3.5 | 1.14E-02 | 157.7 | 45.5 |
| ENSG00000139613.12 | *SMARCC2* | -1.5 | 2.16E-02 | 1740.7 | 1142.0 |
| ENSG00000183963.18 | *SMTN* | 5.1 | 6.29E-03 | 50.3 | 257.9 |
| ENSG00000124216.4 | *SNAI1* | 3.3 | 7.68E-09 | 74.2 | 244.6 |
| ENSG00000163597.15 | *SNHG16* | 5.2 | 2.93E-03 | 45.4 | 236.0 |
| ENSG00000242125.3 | *SNHG3* | 1.5 | 5.04E-03 | 1042.5 | 1584.5 |
| ENSG00000267321.3 | *SNHG30* | 2.2 | 2.57E-02 | 53.9 | 117.5 |
| ENSG00000203875.12 | *SNHG5* | 1.7 | 5.18E-03 | 861.9 | 1470.3 |
| ENSG00000125743.10 | *SNRPD2* | 30.9 | 3.08E-02 | 48.6 | 1498.2 |
| ENSG00000178996.14 | *SNX18* | 2.2 | 1.41E-05 | 162.7 | 350.9 |
| ENSG00000149639.15 | *SOGA1* | -2.1 | 4.05E-12 | 2057.9 | 974.3 |
| ENSG00000159140.20 | *SON* | -1.7 | 4.53E-03 | 4174.8 | 2523.8 |
| ENSG00000115904.12 | *SOS1* | -12.8 | 1.50E-02 | 72.8 | 5.7 |
| ENSG00000198142.5 | *SOWAHC* | 3.3 | 4.44E-24 | 336.6 | 1115.9 |
| ENSG00000125398.8 | *SOX9* | 3.1 | 1.98E-02 | 19.0 | 59.2 |
| ENSG00000158792.16 | *SPATA2L* | 3.4 | 4.78E-03 | 45.1 | 152.5 |
| ENSG00000152253.9 | *SPC25* | 2.0 | 1.32E-02 | 249.7 | 508.0 |
| ENSG00000176170.13 | *SPHK1* | 2.2 | 7.09E-04 | 255.0 | 570.4 |
| ENSG00000134668.12 | *SPOCD1* | 3.7 | 4.32E-08 | 51.7 | 193.1 |
| ENSG00000157837.16 | *SPPL3* | -2.0 | 3.35E-02 | 487.4 | 247.4 |
| ENSG00000166068.13 | *SPRED1* | 1.7 | 1.61E-02 | 334.6 | 558.1 |
| ENSG00000136158.12 | *SPRY2* | 2.7 | 7.22E-03 | 33.6 | 91.0 |
| ENSG00000187678.9 | *SPRY4* | 208.6 | 7.68E-04 | 0.1 | 26.7 |
| ENSG00000112081.17 | *SRSF3* | 1.8 | 1.58E-02 | 5654.1 | 10177.4 |
| ENSG00000100650.16 | *SRSF5* | 1.7 | 7.94E-05 | 2882.7 | 5007.6 |
| ENSG00000115875.19 | *SRSF7* | 1.7 | 5.67E-04 | 2945.7 | 4971.3 |
| ENSG00000138134.12 | *STAMBPL1* | 1.7 | 2.15E-02 | 291.9 | 483.2 |
| ENSG00000164211.13 | *STARD4* | 1.9 | 4.53E-03 | 353.9 | 689.5 |
| ENSG00000113739.10 | *STC2* | 2.7 | 1.36E-02 | 70.1 | 191.0 |
| ENSG00000168439.16 | *STIP1* | 5.0 | 1.33E-02 | 88.2 | 440.6 |
| ENSG00000078269.15 | *SYNJ2* | 1.8 | 2.99E-05 | 407.9 | 740.6 |
| ENSG00000182253.15 | *SYNM* | -1.6 | 3.17E-02 | 421.1 | 263.9 |
| ENSG00000171992.13 | *SYNPO* | -2.0 | 3.45E-05 | 532.8 | 266.9 |
| ENSG00000162298.18 | *SYVN1* | -1.7 | 2.08E-03 | 530.7 | 305.2 |
| ENSG00000148835.11 | *TAF5* | -3.2 | 2.93E-03 | 187.8 | 58.6 |
| ENSG00000149591.17 | *TAGLN* | 4.1 | 5.83E-23 | 168.1 | 681.9 |
| ENSG00000158710.14 | *TAGLN2* | 32.3 | 2.63E-02 | 34.9 | 1125.7 |
| ENSG00000168394.11 | *TAP1* | -19.9 | 3.65E-02 | 74.1 | 3.7 |
| ENSG00000113407.13 | *TARS* | 4.1 | 6.41E-03 | 166.1 | 677.8 |
| ENSG00000112773.16 | *TENT5A* | -1.8 | 7.07E-04 | 441.7 | 242.3 |
| ENSG00000158246.8 | *TENT5B* | -1.9 | 4.73E-03 | 246.4 | 131.5 |
| ENSG00000090447.12 | *TFAP4* | -1.6 | 1.21E-02 | 550.9 | 341.6 |
| ENSG00000137801.11 | *THBS1* | 5.7 | 7.29E-10 | 4759.5 | 27177.9 |
| ENSG00000206573.9 | *THUMPD3-AS1* | 2.1 | 4.78E-03 | 130.2 | 275.7 |
| ENSG00000140534.14 | *TICRR* | -2.3 | 3.94E-12 | 984.4 | 426.8 |
| ENSG00000078237.7 | *TIGAR* | 2.1 | 4.84E-02 | 234.4 | 488.0 |
| ENSG00000232112.3 | *TMA7* | 1.9 | 3.47E-02 | 1290.6 | 2469.0 |
| ENSG00000075568.17 | *TMEM131* | -1.9 | 3.29E-02 | 483.6 | 250.6 |
| ENSG00000165152.9 | *TMEM246* | -1.6 | 1.42E-04 | 1216.1 | 758.2 |
| ENSG00000166471.11 | *TMEM41B* | 1.6 | 4.29E-02 | 428.1 | 680.2 |
| ENSG00000153214.11 | *TMEM87B* | 2.0 | 1.33E-02 | 124.4 | 249.7 |
| ENSG00000104689.10 | *TNFRSF10A* | 2.8 | 1.37E-03 | 129.6 | 363.6 |
| ENSG00000120889.13 | *TNFRSF10B* | 1.6 | 1.21E-04 | 797.4 | 1310.5 |
| ENSG00000067182.7 | *TNFRSF1A* | 15.7 | 3.43E-02 | 8.7 | 135.7 |
| ENSG00000136205.17 | *TNS3* | -1.6 | 3.82E-03 | 2933.5 | 1875.8 |
| ENSG00000198113.3 | *TOR4A* | 1.7 | 4.47E-02 | 447.2 | 752.6 |
| ENSG00000140416.21 | *TPM1* | 1.6 | 3.93E-02 | 3603.4 | 5789.1 |
| ENSG00000076604.15 | *TRAF4* | 1.7 | 1.08E-03 | 480.1 | 825.4 |
| ENSG00000204977.10 | *TRIM13* | 2.6 | 1.85E-05 | 81.9 | 210.1 |
| ENSG00000104907.12 | *TRMT1* | 2.9 | 3.40E-02 | 232.6 | 669.9 |
| ENSG00000121486.12 | *TRMT1L* | -1.9 | 2.64E-02 | 366.6 | 189.3 |
| ENSG00000157514.16 | *TSC22D3* | 2.7 | 1.11E-04 | 74.4 | 197.3 |
| ENSG00000136295.15 | *TTYH3* | -1.6 | 8.43E-03 | 1759.4 | 1071.5 |
| ENSG00000143367.16 | *TUFT1* | 2.4 | 8.71E-17 | 840.7 | 2034.2 |
| ENSG00000122691.13 | *TWIST1* | -1.8 | 7.12E-05 | 881.7 | 478.2 |
| ENSG00000185262.9 | *UBALD2* | -1.8 | 4.70E-03 | 476.5 | 270.0 |
| ENSG00000154127.10 | *UBASH3B* | 2.0 | 4.80E-02 | 354.3 | 709.4 |
| ENSG00000103275.20 | *UBE2I* | -145.0 | 9.50E-04 | 38.3 | 0.3 |
| ENSG00000157741.15 | *UBN2* | -1.8 | 2.18E-02 | 341.2 | 185.9 |
| ENSG00000160803.8 | *UBQLN4* | -1.6 | 2.02E-03 | 1515.6 | 956.4 |
| ENSG00000005007.12 | *UPF1* | -2.5 | 1.92E-02 | 224.6 | 88.2 |
| ENSG00000183696.14 | *UPP1* | 2.7 | 2.17E-04 | 165.5 | 440.6 |
| ENSG00000164405.11 | *UQCRQ* | 1.5 | 4.42E-02 | 1080.6 | 1673.1 |
| ENSG00000132467.4 | *UTP3* | 1.7 | 5.03E-03 | 369.3 | 610.3 |
| ENSG00000168140.5 | *VASN* | 2.8 | 2.92E-14 | 620.9 | 1751.6 |
| ENSG00000125753.14 | *VASP* | 2.1 | 3.92E-02 | 798.6 | 1659.8 |
| ENSG00000035403.17 | *VCL* | 2.4 | 3.32E-02 | 1309.8 | 3120.7 |
| ENSG00000062716.13 | *VMP1* | 2.1 | 7.86E-07 | 1571.6 | 3265.3 |
| ENSG00000122958.15 | *VPS26A* | 1.7 | 2.17E-02 | 839.0 | 1413.6 |
| ENSG00000115368.10 | *WDR75* | 2.3 | 1.12E-02 | 737.3 | 1715.4 |
| ENSG00000100219.16 | *XBP1* | 2.0 | 2.49E-05 | 779.1 | 1531.4 |
| ENSG00000154767.14 | *XPC* | 3.6 | 4.80E-02 | 13.2 | 47.2 |
| ENSG00000114127.10 | *XRN1* | 3.3 | 4.77E-06 | 68.1 | 226.4 |
| ENSG00000015153.14 | *YAF2* | 2.0 | 3.71E-02 | 78.0 | 156.7 |
| ENSG00000134684.10 | *YARS* | 2.0 | 3.32E-02 | 89.1 | 181.9 |
| ENSG00000196449.4 | *YRDC* | 1.9 | 1.84E-04 | 374.4 | 726.9 |
| ENSG00000179456.10 | *ZBTB18* | -1.6 | 4.43E-02 | 446.9 | 285.2 |
| ENSG00000174282.12 | *ZBTB4* | -1.6 | 1.07E-02 | 476.2 | 300.8 |
| ENSG00000184677.18 | *ZBTB40* | -1.8 | 2.81E-04 | 807.7 | 454.5 |
| ENSG00000168795.5 | *ZBTB5* | -1.8 | 2.94E-03 | 504.6 | 288.1 |
| ENSG00000163874.11 | *ZC3H12A* | 2.1 | 1.01E-02 | 78.2 | 165.5 |
| ENSG00000144161.13 | *ZC3H8* | 1.6 | 4.75E-02 | 179.1 | 287.5 |
| ENSG00000140948.12 | *ZCCHC14* | -1.7 | 4.80E-02 | 464.6 | 273.3 |
| ENSG00000204160.12 | *ZDHHC18* | -1.6 | 1.04E-02 | 577.9 | 350.3 |
| ENSG00000091656.19 | *ZFHX4* | -2.4 | 1.19E-02 | 380.4 | 160.2 |
| ENSG00000185650.9 | *ZFP36L1* | 77.9 | 4.21E-03 | 8.4 | 655.6 |
| ENSG00000152518.8 | *ZFP36L2* | 2.0 | 3.83E-09 | 561.9 | 1103.9 |
| ENSG00000108175.17 | *ZMIZ1* | -1.7 | 4.34E-03 | 1536.6 | 920.5 |
| ENSG00000147130.14 | *ZMYM3* | -1.6 | 2.69E-02 | 775.9 | 474.9 |
| ENSG00000198393.8 | *ZNF26* | 2.3 | 3.38E-03 | 71.6 | 164.9 |
| ENSG00000171467.16 | *ZNF318* | -1.8 | 2.30E-03 | 558.1 | 317.8 |
| ENSG00000109445.11 | *ZNF330* | 2.0 | 2.59E-02 | 438.9 | 877.3 |
| ENSG00000173545.5 | *ZNF622* | 1.7 | 3.99E-02 | 867.7 | 1479.9 |
| ENSG00000156853.13 | *ZNF689* | -1.7 | 4.57E-02 | 221.5 | 126.8 |
| ENSG00000143067.5 | *ZNF697* | 3.2 | 1.26E-06 | 68.8 | 220.6 |
| ENSG00000128000.16 | *ZNF780B* | -2.4 | 1.93E-02 | 127.7 | 53.3 |
| ENSG00000132003.9 | *ZSWIM4* | 319.7 | 9.91E-07 | 0.1 | 41.3 |

**Supplementary Table 6. Differentially expressed genes in CGL1^FRA1KO^ cells compared to CGL1^Cas9^ cells 2 hours after serum stimulation.**

RNA-sequencing was performed to identify DEGs between CGL1^FRA1KO^ and CGL1^Cas9^ cells 2 hours after serum stimulation. DEGs were defined by a fold change (F.C.) >1.5 or <–1.5 and FDR-adjusted *p*-value (p-adj) < 0.05. A total of 223 DEGs were identified, indicating that loss of FRA1 markedly alters the gene expression response to early serum stimulation. Of these, 172 genes were upregulated and 51 were downregulated relative to control cells. Gene names, fold changes, p-adj values, and transcript abundance (TPM) are provided in alphabetical order.

| **Gene ID** | **Gene Name** | **F.C.** | **p-adj.** | **CGL1^Cas9^** | **CGL1^FRA1KO^** |
| --- | --- | --- | --- | --- | --- |
| ENSG00000159251.8 | *ACTC1* | -2.6 | 1.41E-02 | 83.1 | 32.1 |
| ENSG00000178031.17 | *ADAMTSL1* | -1.9 | 2.13E-03 | 245.7 | 127.2 |
| ENSG00000117114.19 | *ADGRL2* | 440.8 | 9.91E-10 | 0.2 | 84.6 |
| ENSG00000163295.5 | *ALPI* | 68.3 | 8.84E-04 | 0.3 | 23.8 |
| ENSG00000178038.17 | *ALS2CL* | 5.9 | 1.88E-02 | 4.5 | 26.6 |
| ENSG00000076513.17 | *ANKRD13A* | -1.5 | 1.03E-04 | 1343.0 | 868.6 |
| ENSG00000166825.14 | *ANPEP* | 1.8 | 6.23E-08 | 434.3 | 795.7 |
| ENSG00000128335.14 | *APOL2* | 1.7 | 9.99E-06 | 232.8 | 397.5 |
| ENSG00000221963.6 | *APOL6* | 2.2 | 8.84E-13 | 244.6 | 533.3 |
| ENSG00000165272.16 | *AQP3* | 3.9 | 1.80E-12 | 50.2 | 196.1 |
| ENSG00000188042.8 | *ARL4C* | 1.7 | 4.42E-02 | 88.0 | 147.8 |
| ENSG00000085224.22 | *ATRX* | 1.6 | 5.34E-03 | 1144.2 | 1866.3 |
| ENSG00000166710.20 | *B2M* | 1.6 | 3.79E-02 | 168.6 | 274.3 |
| ENSG00000183092.16 | *BEGAIN* | -2.6 | 6.23E-09 | 417.8 | 160.0 |
| ENSG00000159388.6 | *BTG2* | 1.6 | 1.03E-02 | 138.1 | 224.7 |
| ENSG00000182326.15 | *C1S* | 10.7 | 4.27E-02 | 3.5 | 37.6 |
| ENSG00000172375.13 | *C2CD2L* | -2.2 | 6.25E-03 | 97.1 | 43.4 |
| ENSG00000125730.17 | *C3* | 8.5 | 9.01E-03 | 8.1 | 69.2 |
| ENSG00000244731.8 | *C4A* | 5.2 | 3.72E-02 | 5.4 | 28.0 |
| ENSG00000224389.9 | *C4B* | 7.0 | 1.34E-02 | 5.3 | 37.1 |
| ENSG00000168497.5 | *CAVIN2* | 2.2 | 3.52E-09 | 150.8 | 333.0 |
| ENSG00000078699.21 | *CBFA2T2* | -4.8 | 4.27E-02 | 78.6 | 16.4 |
| ENSG00000271503.6 | *CCL5* | 16.2 | 7.92E-51 | 24.3 | 392.0 |
| ENSG00000135837.16 | *CEP350* | 1.7 | 3.17E-03 | 290.5 | 494.8 |
| ENSG00000163347.6 | *CLDN1* | 2.9 | 6.37E-08 | 61.1 | 175.8 |
| ENSG00000100473.18 | *COCH* | -2.8 | 4.52E-03 | 78.4 | 28.5 |
| ENSG00000163359.15 | *COL6A3* | 1.7 | 1.62E-07 | 430.5 | 723.5 |
| ENSG00000158270.12 | *COLEC12* | 5.3 | 7.28E-07 | 17.8 | 95.0 |
| ENSG00000128510.12 | *CPA4* | 1.7 | 2.69E-14 | 2191.1 | 3732.4 |
| ENSG00000143320.9 | *CRABP2* | 1.6 | 1.61E-05 | 708.9 | 1124.7 |
| ENSG00000213145.10 | *CRIP1* | 2.2 | 1.62E-07 | 130.8 | 285.8 |
| ENSG00000172346.15 | *CSDC2* | -1.7 | 4.93E-03 | 255.9 | 149.7 |
| ENSG00000184371.14 | *CSF1* | 1.6 | 1.14E-05 | 281.1 | 459.7 |
| ENSG00000169429.11 | *CXCL8* | 2.3 | 5.93E-03 | 45.3 | 102.4 |
| ENSG00000138061.12 | *CYP1B1* | 1.7 | 9.55E-22 | 2536.0 | 4326.8 |
| ENSG00000197191.6 | *CYSRT1* | 4.2 | 4.48E-02 | 6.5 | 27.6 |
| ENSG00000107201.10 | *DDX58* | 5.7 | 1.04E-03 | 98.1 | 557.4 |
| ENSG00000137628.17 | *DDX60* | 3.6 | 7.17E-28 | 256.6 | 910.9 |
| ENSG00000108771.13 | *DHX58* | 4.7 | 1.41E-05 | 16.1 | 74.9 |
| ENSG00000187957.8 | *DNER* | 4.2 | 1.04E-03 | 11.8 | 49.2 |
| ENSG00000106976.20 | *DNM1* | -1.7 | 1.00E-03 | 272.7 | 157.0 |
| ENSG00000147251.15 | *DOCK11* | 1.8 | 1.53E-02 | 315.4 | 557.6 |
| ENSG00000136048.14 | *DRAM1* | 1.7 | 1.61E-10 | 438.3 | 739.2 |
| ENSG00000163840.10 | *DTX3L* | 1.9 | 1.09E-09 | 292.7 | 541.6 |
| ENSG00000158050.5 | *DUSP2* | -1.5 | 3.61E-03 | 608.9 | 393.1 |
| ENSG00000120875.9 | *DUSP4* | 2.7 | 9.09E-49 | 428.3 | 1169.5 |
| ENSG00000115380.20 | *EFEMP1* | 1.7 | 6.93E-38 | 3793.3 | 6417.9 |
| ENSG00000163435.16 | *ELF3* | 4.7 | 6.05E-04 | 17.2 | 81.0 |
| ENSG00000116016.14 | *EPAS1* | 3.0 | 8.40E-04 | 83.6 | 250.8 |
| ENSG00000261150.2 | *EPPK1* | 2.9 | 3.04E-04 | 142.2 | 415.9 |
| ENSG00000141736.13 | *ERBB2* | -1.8 | 3.33E-04 | 493.4 | 276.0 |
| ENSG00000087502.18 | *ERGIC2* | 1.6 | 6.05E-04 | 560.8 | 889.5 |
| ENSG00000173153.14 | *ESRRA* | -2.2 | 1.92E-02 | 207.0 | 93.6 |
| ENSG00000244405.8 | *ETV5* | 1.7 | 4.11E-03 | 227.0 | 385.2 |
| ENSG00000140285.10 | *FGF7* | -4.6 | 4.23E-04 | 48.9 | 10.6 |
| ENSG00000176692.8 | *FOXC2* | 1.6 | 4.61E-02 | 93.7 | 152.8 |
| ENSG00000157240.4 | *FZD1* | 1.5 | 2.26E-02 | 324.1 | 500.0 |
| ENSG00000174804.4 | *FZD4* | 2.2 | 5.20E-12 | 218.6 | 474.4 |
| ENSG00000033327.13 | *GAB2* | -1.6 | 2.72E-04 | 449.5 | 281.7 |
| ENSG00000268089.3 | *GABRQ* | -2.2 | 1.40E-02 | 110.6 | 49.9 |
| ENSG00000272695.2 | *GAS6-DT* | 1.8 | 4.38E-02 | 59.1 | 108.3 |
| ENSG00000117228.10 | *GBP1* | 3.2 | 2.45E-32 | 202.5 | 639.4 |
| ENSG00000117226.12 | *GBP3* | 1.9 | 2.51E-03 | 116.4 | 224.0 |
| ENSG00000130513.6 | *GDF15* | 1.9 | 6.18E-10 | 628.2 | 1198.9 |
| ENSG00000127920.6 | *GNG11* | 1.6 | 8.54E-03 | 137.4 | 224.8 |
| ENSG00000168243.11 | *GNG4* | -2.9 | 1.33E-02 | 59.8 | 20.9 |
| ENSG00000138271.6 | *GPR87* | -1.8 | 4.66E-02 | 171.0 | 96.6 |
| ENSG00000155324.10 | *GRAMD2B* | 1.7 | 1.79E-08 | 457.8 | 761.6 |
| ENSG00000173706.14 | *HEG1* | 1.7 | 3.68E-17 | 1918.2 | 3221.6 |
| ENSG00000103657.14 | *HERC1* | 1.5 | 1.17E-02 | 957.8 | 1480.7 |
| ENSG00000138646.9 | *HERC5* | 5.4 | 1.89E-37 | 84.7 | 453.7 |
| ENSG00000270276.2 | *HIST2H4B* | -301.8 | 1.57E-07 | 53.0 | 0.2 |
| ENSG00000234745.11 | *HLA-B* | 1.9 | 1.83E-45 | 3008.7 | 5809.3 |
| ENSG00000204642.14 | *HLA-F* | 2.9 | 7.99E-03 | 20.5 | 60.0 |
| ENSG00000164120.14 | *HPGD* | -4.3 | 2.33E-02 | 29.6 | 6.9 |
| ENSG00000142149.9 | *HUNK* | -2.8 | 7.57E-05 | 125.4 | 45.1 |
| ENSG00000090339.9 | *ICAM1* | 1.7 | 1.15E-06 | 282.9 | 481.6 |
| ENSG00000115738.10 | *ID2* | -2.0 | 7.85E-04 | 164.4 | 82.1 |
| ENSG00000216490.4 | *IFI30* | 1.7 | 3.63E-04 | 241.9 | 399.8 |
| ENSG00000137965.11 | *IFI44* | 2.2 | 4.74E-16 | 421.4 | 921.5 |
| ENSG00000126709.15 | *IFI6* | 2.1 | 4.17E-07 | 128.3 | 270.9 |
| ENSG00000115267.8 | *IFIH1* | 6.5 | 1.63E-15 | 28.0 | 182.4 |
| ENSG00000185745.10 | *IFIT1* | 3.3 | 1.82E-38 | 383.1 | 1273.7 |
| ENSG00000119922.10 | *IFIT2* | 2005.6 | 7.64E-17 | 0.2 | 386.2 |
| ENSG00000119917.14 | *IFIT3* | 3.7 | ######## | 640.2 | 2394.9 |
| ENSG00000152778.9 | *IFIT5* | 1.6 | 9.01E-03 | 162.1 | 253.6 |
| ENSG00000185885.16 | *IFITM1* | 1.6 | 8.24E-04 | 271.1 | 423.8 |
| ENSG00000142166.13 | *IFNAR1* | 1.8 | 1.63E-04 | 200.0 | 368.9 |
| ENSG00000141753.7 | *IGFBP4* | 5.5 | 3.49E-38 | 75.1 | 415.0 |
| ENSG00000115461.5 | *IGFBP5* | 3.9 | 6.29E-12 | 42.4 | 164.8 |
| ENSG00000167779.9 | *IGFBP6* | 2.5 | 2.52E-15 | 284.7 | 719.6 |
| ENSG00000163453.11 | *IGFBP7* | 1.8 | 1.17E-08 | 484.1 | 863.7 |
| ENSG00000030419.16 | *IKZF2* | -6.8 | 3.05E-02 | 33.9 | 5.0 |
| ENSG00000136244.12 | *IL6* | 8.0 | 8.17E-28 | 30.1 | 242.1 |
| ENSG00000134352.20 | *IL6ST* | 5.9 | 4.14E-03 | 26.6 | 155.7 |
| ENSG00000125347.14 | *IRF1* | 1.7 | 2.33E-05 | 377.0 | 626.1 |
| ENSG00000187608.10 | *ISG15* | 2.3 | 1.26E-22 | 908.5 | 2125.8 |
| ENSG00000172183.15 | *ISG20* | 4.4 | 9.32E-03 | 11.0 | 48.3 |
| ENSG00000164171.11 | *ITGA2* | 1.8 | 3.42E-02 | 104.6 | 191.2 |
| ENSG00000091409.15 | *ITGA6* | 7.7 | 8.95E-07 | 10.9 | 84.4 |
| ENSG00000132470.14 | *ITGB4* | 3.2 | 1.17E-05 | 43.3 | 138.2 |
| ENSG00000082781.12 | *ITGB5* | 1.8 | 5.52E-07 | 849.2 | 1527.8 |
| ENSG00000123104.12 | *ITPR2* | 1.7 | 1.17E-03 | 405.3 | 687.4 |
| ENSG00000184185.10 | *KCNJ12* | 1.8 | 4.42E-02 | 83.9 | 148.4 |
| ENSG00000121361.5 | *KCNJ8* | -3.9 | 2.10E-02 | 33.3 | 8.6 |
| ENSG00000138688.15 | *KIAA1109* | 2.1 | 1.62E-02 | 108.6 | 223.7 |
| ENSG00000120549.18 | *KIAA1217* | 1.8 | 3.25E-02 | 100.0 | 177.6 |
| ENSG00000136826.15 | *KLF4* | 1.9 | 5.06E-15 | 509.2 | 947.4 |
| ENSG00000114796.16 | *KLHL24* | -1.9 | 1.22E-04 | 324.3 | 174.7 |
| ENSG00000128422.17 | *KRT17* | 4.4 | ######## | 400.6 | 1773.1 |
| ENSG00000198910.14 | *L1CAM* | 1.9 | 4.30E-06 | 770.4 | 1467.3 |
| ENSG00000213626.13 | *LBH* | -1.6 | 1.56E-02 | 468.4 | 291.5 |
| ENSG00000143355.16 | *LHX9* | 3.5 | 1.19E-07 | 34.6 | 121.2 |
| ENSG00000073350.13 | *LLGL2* | -1.8 | 1.53E-02 | 191.7 | 108.7 |
| ENSG00000147676.14 | *MAL2* | 4.5 | 1.16E-21 | 60.9 | 275.9 |
| ENSG00000078018.19 | *MAP2* | -2.1 | 2.72E-04 | 145.5 | 67.7 |
| ENSG00000162591.16 | *MEGF6* | -2.1 | 4.45E-02 | 219.1 | 105.5 |
| ENSG00000099812.9 | *MISP* | 2.3 | 2.13E-03 | 49.9 | 114.6 |
| ENSG00000087245.13 | *MMP2* | 1.6 | 2.86E-06 | 419.6 | 670.2 |
| ENSG00000125966.10 | *MMP24* | 2.3 | 5.32E-09 | 193.8 | 436.8 |
| ENSG00000261971.8 | *MMP25-AS1* | 3.1 | 1.05E-02 | 17.5 | 54.2 |
| ENSG00000196588.17 | *MRTFA* | -1.8 | 1.91E-03 | 350.8 | 190.0 |
| ENSG00000129422.14 | *MTUS1* | 1.8 | 1.33E-02 | 178.0 | 313.5 |
| ENSG00000185499.16 | *MUC1* | 1.9 | 3.05E-02 | 80.5 | 150.8 |
| ENSG00000013364.19 | *MVP* | 1.7 | 6.04E-03 | 353.7 | 591.7 |
| ENSG00000101825.8 | *MXRA5* | 1.7 | 1.34E-04 | 220.4 | 374.6 |
| ENSG00000172927.8 | *MYEOV* | 1.9 | 1.76E-02 | 67.1 | 127.2 |
| ENSG00000176658.17 | *MYO1D* | -2.1 | 1.49E-02 | 127.1 | 60.1 |
| ENSG00000157483.9 | *MYO1E* | 1.6 | 3.16E-02 | 414.8 | 677.6 |
| ENSG00000136286.16 | *MYO1G* | 6.3 | 1.63E-02 | 4.2 | 26.3 |
| ENSG00000111912.20 | *NCOA7* | 2.0 | 6.53E-17 | 456.8 | 920.4 |
| ENSG00000172260.15 | *NEGR1* | 1.6 | 1.11E-02 | 193.2 | 318.2 |
| ENSG00000151414.15 | *NEK7* | 1.6 | 2.65E-13 | 2312.6 | 3648.3 |
| ENSG00000050344.9 | *NFE2L3* | 2.2 | 2.53E-03 | 78.9 | 173.7 |
| ENSG00000100906.10 | *NFKBIA* | 3.9 | 1.12E-03 | 17.9 | 69.0 |
| ENSG00000135540.11 | *NHSL1* | 10.5 | 8.32E-03 | 3.5 | 36.5 |
| ENSG00000204131.9 | *NHSL2* | -10.8 | 2.17E-02 | 18.6 | 1.7 |
| ENSG00000087303.18 | *NID2* | 5.7 | 6.28E-03 | 6.1 | 35.1 |
| ENSG00000123609.11 | *NMI* | 1.7 | 2.65E-02 | 97.3 | 163.9 |
| ENSG00000166741.7 | *NNMT* | 1.6 | 5.08E-04 | 311.1 | 507.1 |
| ENSG00000135318.12 | *NT5E* | 1.6 | 7.10E-08 | 739.5 | 1186.9 |
| ENSG00000074527.12 | *NTN4* | 1.6 | 1.63E-04 | 321.6 | 516.5 |
| ENSG00000111331.13 | *OAS3* | 1.6 | 7.99E-03 | 409.1 | 659.4 |
| ENSG00000154358.21 | *OBSCN* | -1.8 | 3.95E-03 | 340.3 | 185.5 |
| ENSG00000185585.20 | *OLFML2A* | 1.9 | 2.04E-05 | 129.1 | 247.9 |
| ENSG00000135473.15 | *PAN2* | 2.0 | 1.25E-02 | 122.5 | 239.0 |
| ENSG00000182752.10 | *PAPPA* | 4.1 | 7.19E-07 | 22.9 | 93.3 |
| ENSG00000173193.15 | *PARP14* | 2.1 | 2.22E-13 | 445.3 | 928.2 |
| ENSG00000138496.16 | *PARP9* | 2.0 | 3.78E-02 | 72.7 | 143.9 |
| ENSG00000138650.9 | *PCDH10* | -1.8 | 1.33E-02 | 163.1 | 88.2 |
| ENSG00000197646.8 | *PDCD1LG2* | 2.3 | 4.49E-02 | 27.8 | 64.5 |
| ENSG00000100311.17 | *PDGFB* | 2.1 | 1.03E-14 | 480.0 | 1007.3 |
| ENSG00000132326.12 | *PER2* | 32.9 | 2.07E-02 | 0.8 | 25.0 |
| ENSG00000255200.1 | *PGAM1P8* | 7.3 | 4.48E-02 | 2.6 | 18.7 |
| ENSG00000144824.21 | *PHLDB2* | 1.6 | 1.41E-11 | 1528.6 | 2446.6 |
| ENSG00000164530.15 | *PI16* | 2.2 | 4.48E-02 | 51.6 | 115.5 |
| ENSG00000133321.11 | *PLAAT4* | 4.8 | 3.21E-18 | 54.8 | 263.1 |
| ENSG00000011422.12 | *PLAUR* | 1.7 | 2.67E-04 | 372.4 | 617.3 |
| ENSG00000240694.9 | *PNMA2* | 2.2 | 4.66E-10 | 165.7 | 364.8 |
| ENSG00000128567.17 | *PODXL* | 1.5 | 1.80E-17 | 2557.0 | 3879.5 |
| ENSG00000133110.15 | *POSTN* | 1.6 | 5.62E-05 | 4631.7 | 7325.4 |
| ENSG00000156475.18 | *PPP2R2B* | -1.5 | 4.14E-03 | 392.0 | 257.6 |
| ENSG00000124126.14 | *PREX1* | -1.7 | 2.67E-02 | 338.9 | 197.7 |
| ENSG00000101000.6 | *PROCR* | 1.6 | 2.97E-04 | 349.3 | 560.9 |
| ENSG00000176532.4 | *PRR15* | 3.7 | 6.82E-04 | 21.4 | 78.6 |
| ENSG00000150687.12 | *PRSS23* | 1.5 | 8.30E-22 | 1998.4 | 3091.5 |
| ENSG00000240065.8 | *PSMB9* | 3.2 | 2.25E-09 | 52.9 | 168.8 |
| ENSG00000163661.4 | *PTX3* | 3.4 | 2.60E-04 | 21.1 | 71.2 |
| ENSG00000099246.16 | *RAB18* | -112.1 | 1.26E-03 | 40.6 | 0.4 |
| ENSG00000041353.10 | *RAB27B* | 2.8 | 2.32E-02 | 21.5 | 61.1 |
| ENSG00000183688.4 | *RFLNB* | -1.6 | 4.83E-04 | 433.3 | 268.0 |
| ENSG00000101236.17 | *RNF24* | 1.6 | 3.28E-07 | 381.1 | 609.4 |
| ENSG00000166592.12 | *RRAD* | 3.0 | 1.41E-03 | 25.3 | 74.9 |
| ENSG00000134321.12 | *RSAD2* | 68.6 | 1.51E-15 | 2.0 | 138.5 |
| ENSG00000124813.23 | *RUNX2* | 1.5 | 3.05E-02 | 156.3 | 240.5 |
| ENSG00000196154.12 | *S100A4* | 1.7 | 1.87E-17 | 857.9 | 1435.1 |
| ENSG00000173432.12 | *SAA1* | 7.8 | 1.02E-02 | 3.2 | 24.8 |
| ENSG00000187634.12 | *SAMD11* | -2.5 | 1.99E-04 | 197.4 | 78.9 |
| ENSG00000205413.8 | *SAMD9* | 3.3 | 1.55E-10 | 158.9 | 528.2 |
| ENSG00000177409.12 | *SAMD9L* | 4.0 | 1.44E-03 | 22.4 | 90.7 |
| ENSG00000151967.18 | *SCHIP1* | 6.2 | 2.97E-04 | 24.1 | 149.3 |
| ENSG00000169247.12 | *SH3TC2* | 2.6 | 3.52E-03 | 41.2 | 108.8 |
| ENSG00000221955.10 | *SLC12A8* | 2.7 | 4.52E-03 | 48.0 | 128.1 |
| ENSG00000197375.12 | *SLC22A5* | 8.1 | 1.47E-02 | 9.9 | 80.6 |
| ENSG00000151229.13 | *SLC2A13* | 1.8 | 1.22E-04 | 187.6 | 332.7 |
| ENSG00000131389.17 | *SLC6A6* | -2.0 | 8.15E-03 | 680.1 | 338.9 |
| ENSG00000176463.14 | *SLCO3A1* | 2.1 | 3.43E-02 | 71.3 | 146.6 |
| ENSG00000130340.16 | *SNX9* | -16.4 | 1.51E-02 | 37.8 | 2.3 |
| ENSG00000125398.8 | *SOX9* | 1.9 | 1.59E-03 | 102.9 | 192.1 |
| ENSG00000134668.12 | *SPOCD1* | -2.3 | 2.40E-05 | 227.6 | 100.4 |
| ENSG00000137767.14 | *SQOR* | 1.9 | 3.80E-03 | 100.0 | 193.5 |
| ENSG00000179954.16 | *SSC5D* | 5.0 | 7.14E-04 | 8.9 | 44.7 |
| ENSG00000117069.15 | *ST6GALNAC5* | -2.2 | 3.17E-03 | 116.5 | 54.1 |
| ENSG00000137868.19 | *STRA6* | 3.2 | 2.41E-15 | 199.6 | 640.3 |
| ENSG00000197321.14 | *SVIL* | 1.8 | 1.97E-04 | 244.1 | 447.4 |
| ENSG00000176907.5 | *TCIM* | 23.3 | 1.91E-03 | 1.1 | 24.7 |
| ENSG00000196116.8 | *TDRD7* | 2.3 | 7.29E-06 | 85.4 | 196.7 |
| ENSG00000164081.12 | *TEX264* | -3.6 | 5.53E-03 | 173.8 | 47.8 |
| ENSG00000092969.12 | *TGFB2* | 1.6 | 1.24E-06 | 623.9 | 988.8 |
| ENSG00000198959.12 | *TGM2* | 4.7 | 1.38E-04 | 22.1 | 103.6 |
| ENSG00000271092.5 | *TLCD4-RWDD3* | -5.2 | 3.06E-02 | 26.9 | 5.2 |
| ENSG00000164484.11 | *TMEM200A* | 1.7 | 1.87E-02 | 122.1 | 205.1 |
| ENSG00000041982.16 | *TNC* | 3.0 | 2.50E-04 | 35.0 | 103.4 |
| ENSG00000185215.9 | *TNFAIP2* | 2.3 | 3.04E-04 | 90.3 | 206.2 |
| ENSG00000079308.19 | *TNS1* | -2.0 | 3.19E-02 | 231.0 | 116.8 |
| ENSG00000131746.13 | *TNS4* | 5.1 | 7.50E-05 | 12.8 | 65.5 |
| ENSG00000168477.19 | *TNXB* | 2.5 | 2.33E-02 | 45.5 | 114.0 |
| ENSG00000111907.21 | *TPD52L1* | 2.4 | 1.74E-02 | 43.1 | 104.3 |
| ENSG00000132274.16 | *TRIM22* | 3.6 | 1.08E-02 | 24.6 | 89.3 |
| ENSG00000137699.17 | *TRIM29* | 3.2 | 3.61E-06 | 40.6 | 128.1 |
| ENSG00000132481.7 | *TRIM47* | 1.7 | 1.49E-02 | 137.5 | 238.0 |
| ENSG00000067445.21 | *TRO* | -3.4 | 1.58E-04 | 74.6 | 21.7 |
| ENSG00000137672.13 | *TRPC6* | -2.3 | 1.96E-04 | 228.8 | 98.2 |
| ENSG00000130338.13 | *TULP4* | -1.5 | 4.55E-03 | 510.5 | 333.3 |
| ENSG00000156587.16 | *UBE2L6* | 1.9 | 7.30E-03 | 98.9 | 184.8 |
| ENSG00000184979.10 | *USP18* | 2.1 | 1.26E-02 | 51.0 | 108.5 |
| ENSG00000166348.18 | *USP54* | -2.4 | 1.75E-04 | 228.6 | 97.0 |
| ENSG00000038427.16 | *VCAN* | 6.3 | 1.99E-27 | 59.6 | 377.0 |
| ENSG00000105939.13 | *ZC3HAV1* | 2.0 | 5.77E-28 | 1122.3 | 2209.5 |
| ENSG00000062370.16 | *ZNF112* | 19.7 | 5.19E-03 | 1.2 | 23.0 |
| ENSG00000144331.20 | *ZNF385B* | -1.7 | 2.47E-02 | 178.0 | 103.2 |
| ENSG00000197608.11 | *ZNF841* | -2.1 | 2.67E-02 | 83.1 | 40.2 |
| ENSG00000124201.15 | *ZNFX1* | 1.6 | 3.70E-13 | 1022.5 | 1605.7 |

**Supplementary Table 7. Differentially expressed genes in CGL1^FRA1Act^ cells compared to CGL1^dCas9^ cells 2 hours after serum stimulation.**

RNA-sequencing was performed to identify differentially expressed genes (DEGs) between CGL1^FRA1Act^ and CGL1^dCas9^ cells 2 hours after serum stimulation. DEGs were defined by a fold change (F.C.) >1.5 or <–1.5 and FDR-adjusted *p*-value (p-adj) < 0.05. A total of 107 DEGs were identified, indicating that FRA1 overexpression substantially reprograms the early transcriptional response to serum. Of these, 75 genes exhibited attenuated expression (suppressed response), while 32 were further upregulated relative to control cells. Gene names, fold changes, p-adj values, and transcript abundance (TPM) are provided in alphabetical order.

| **Gene ID** | **Gene Name** | **F.C.** | **p-adj.** | **CGL1^dCas9^** | **CGL1^FRA1Act^** |
| --- | --- | --- | --- | --- | --- |
| ENSG00000287263.1 | *AC008875.3* | -97.4 | 1.48E-02 | 25.6 | 0.3 |
| ENSG00000283515.1 | *AC020915.4* | -83.5 | 2.39E-02 | 21.9 | 0.3 |
| ENSG00000259316.11 | *AC087632.2* | 2.2 | 2.18E-02 | 307.3 | 689.2 |
| ENSG00000249773.3 | *AC092647.5* | -80.2 | 3.38E-02 | 21.0 | 0.3 |
| ENSG00000154736.6 | *ADAMTS5* | -2.8 | 6.94E-03 | 154.6 | 55.0 |
| ENSG00000065413.20 | *ANKRD44* | 3.9 | 4.74E-05 | 31.5 | 122.1 |
| ENSG00000166825.14 | *ANPEP* | -2.2 | 1.28E-02 | 566.5 | 262.3 |
| ENSG00000122359.18 | *ANXA11* | -1.5 | 3.95E-02 | 1869.8 | 1207.4 |
| ENSG00000221963.6 | *APOL6* | -2.0 | 1.40E-02 | 355.8 | 180.2 |
| ENSG00000153317.15 | *ASAP1* | -1.6 | 6.21E-03 | 1655.9 | 1062.7 |
| ENSG00000163635.18 | *ATXN7* | -2.8 | 2.72E-02 | 148.2 | 53.6 |
| ENSG00000134107.5 | *BHLHE40* | -1.9 | 1.03E-06 | 2637.3 | 1354.4 |
| ENSG00000271503.6 | *CCL5* | -7.2 | 2.13E-03 | 67.9 | 9.4 |
| ENSG00000142871.17 | *CCN1* | -1.8 | 3.51E-05 | 17357.4 | 9414.9 |
| ENSG00000118523.6 | *CCN2* | -1.7 | 1.55E-03 | 16729.7 | 9934.9 |
| ENSG00000173207.13 | *CKS1B* | 1.7 | 2.27E-02 | 1816.4 | 3084.0 |
| ENSG00000115295.20 | *CLIP4* | -492.2 | 3.08E-06 | 130.3 | 0.3 |
| ENSG00000129083.12 | *COPB1* | 2.7 | 2.04E-02 | 161.4 | 439.3 |
| ENSG00000123815.12 | *COQ8B* | 25.3 | 4.71E-05 | 1.9 | 47.0 |
| ENSG00000128510.12 | *CPA4* | -1.7 | 1.75E-04 | 2683.9 | 1551.1 |
| ENSG00000275496.4 | *CU633906.1* | -4.2 | 1.14E-02 | 76.9 | 18.2 |
| ENSG00000277067.4 | *CU634019.1* | 57.2 | 2.40E-03 | 0.5 | 27.9 |
| ENSG00000138061.12 | *CYP1B1* | -1.5 | 3.59E-03 | 2121.5 | 1375.3 |
| ENSG00000100201.21 | *DDX17* | 3.4 | 2.34E-02 | 486.1 | 1666.7 |
| ENSG00000173852.14 | *DPY19L1* | 165.2 | 2.13E-03 | 0.1 | 21.7 |
| ENSG00000120129.6 | *DUSP1* | -1.8 | 3.51E-05 | 1060.1 | 574.7 |
| ENSG00000120875.9 | *DUSP4* | -2.2 | 9.74E-05 | 420.8 | 187.4 |
| ENSG00000138166.6 | *DUSP5* | -1.9 | 7.69E-05 | 949.4 | 495.4 |
| ENSG00000116406.19 | *EDEM3* | -1267.2 | 1.04E-08 | 335.6 | 0.3 |
| ENSG00000120738.8 | *EGR1* | -2.3 | 1.03E-09 | 5370.4 | 2324.0 |
| ENSG00000179388.9 | *EGR3* | -2.1 | 6.21E-03 | 343.9 | 160.8 |
| ENSG00000075151.20 | *EIF4G3* | -1.9 | 9.95E-03 | 571.9 | 306.1 |
| ENSG00000182150.16 | *ERCC6L2* | -4.2 | 2.42E-02 | 115.7 | 27.7 |
| ENSG00000035141.8 | *FAM136A* | 2.6 | 1.03E-02 | 65.4 | 167.5 |
| ENSG00000115414.19 | *FN1* | -1.8 | 8.52E-07 | 6853.9 | 3733.5 |
| ENSG00000170345.10 | *FOS* | -2.4 | 7.03E-03 | 159.3 | 66.0 |
| ENSG00000125740.14 | *FOSB* | -2.3 | 1.64E-02 | 360.6 | 155.8 |
| ENSG00000176692.8 | *FOXC2* | -2.7 | 1.24E-03 | 180.2 | 65.9 |
| ENSG00000134202.11 | *GSTM3* | 1.8 | 3.97E-02 | 881.2 | 1590.2 |
| ENSG00000092148.13 | *HECTD1* | 1.7 | 3.69E-02 | 663.2 | 1126.6 |
| ENSG00000138646.9 | *HERC5* | -2.2 | 3.10E-02 | 143.6 | 63.8 |
| ENSG00000114315.4 | *HES1* | -1.7 | 5.37E-03 | 676.7 | 397.1 |
| ENSG00000234745.11 | *HLA-B* | -1.7 | 2.93E-06 | 3951.6 | 2357.2 |
| ENSG00000090339.9 | *ICAM1* | -2.1 | 1.40E-02 | 301.3 | 141.0 |
| ENSG00000137331.12 | *IER3* | -2.4 | 1.81E-14 | 4949.9 | 2045.0 |
| ENSG00000185745.10 | *IFIT1* | -2.0 | 1.76E-05 | 591.5 | 290.9 |
| ENSG00000119917.14 | *IFIT3* | -3.0 | 3.95E-02 | 1120.4 | 370.7 |
| ENSG00000214706.10 | *IFRD2* | 3.9 | 3.69E-02 | 160.3 | 631.0 |
| ENSG00000140443.15 | *IGF1R* | -1.6 | 2.53E-02 | 1292.3 | 806.3 |
| ENSG00000163453.11 | *IGFBP7* | -1.8 | 1.02E-03 | 738.3 | 402.3 |
| ENSG00000136244.12 | *IL6* | -5.8 | 2.76E-02 | 45.1 | 7.8 |
| ENSG00000261796.1 | *ISY1-RAB43* | -1.8 | 6.71E-03 | 379.5 | 205.5 |
| ENSG00000161638.11 | *ITGA5* | -1.6 | 9.81E-04 | 3119.7 | 1978.9 |
| ENSG00000171223.6 | *JUNB* | -1.7 | 9.23E-05 | 3692.5 | 2169.3 |
| ENSG00000089177.18 | *KIF16B* | -120.7 | 5.37E-03 | 31.7 | 0.3 |
| ENSG00000066735.14 | *KIF26A* | -2363341.3 | 6.95E-04 | 48.7 | 0.0 |
| ENSG00000128422.17 | *KRT17* | -4.4 | 7.70E-24 | 707.6 | 159.5 |
| ENSG00000198910.14 | *L1CAM* | -2.8 | 1.51E-06 | 957.8 | 340.8 |
| ENSG00000106003.13 | *LFNG* | -1.7 | 3.97E-02 | 452.4 | 264.7 |
| ENSG00000129038.16 | *LOXL1* | -2.4 | 8.62E-07 | 746.2 | 315.7 |
| ENSG00000280987.4 | *MATR3* | 2.0 | 1.01E-02 | 1182.8 | 2379.1 |
| ENSG00000143995.20 | *MEIS1* | -2.1 | 3.21E-02 | 177.1 | 86.2 |
| ENSG00000174197.16 | *MGA* | -14.0 | 4.39E-04 | 58.5 | 4.2 |
| ENSG00000013364.19 | *MVP* | -2.0 | 4.06E-03 | 469.0 | 230.6 |
| ENSG00000179820.16 | *MYADM* | -1.6 | 4.01E-03 | 2867.0 | 1777.9 |
| ENSG00000118680.13 | *MYL12B* | 7.1 | 2.42E-02 | 38.2 | 272.4 |
| ENSG00000164134.13 | *NAA15* | 2.4 | 3.00E-05 | 349.8 | 845.5 |
| ENSG00000008441.16 | *NFIX* | -1.6 | 2.38E-03 | 1140.1 | 714.4 |
| ENSG00000011052.21 | *NME1-NME2* | 3.7 | 1.09E-02 | 130.2 | 487.9 |
| ENSG00000074527.12 | *NTN4* | -1.9 | 2.13E-03 | 455.7 | 241.5 |
| ENSG00000119900.9 | *OGFRL1* | -1.6 | 3.20E-02 | 1075.1 | 652.4 |
| ENSG00000182752.10 | *PAPPA* | -12.4 | 3.11E-03 | 49.1 | 4.0 |
| ENSG00000176485.11 | *PLAAT3* | -4.0 | 1.55E-03 | 90.2 | 22.6 |
| ENSG00000122861.16 | *PLAU* | -1.7 | 4.28E-04 | 1012.0 | 591.3 |
| ENSG00000122512.16 | *PMS2* | 12.6 | 3.85E-02 | 7.9 | 99.5 |
| ENSG00000170734.11 | *POLH* | 3.7 | 3.97E-02 | 75.5 | 280.4 |
| ENSG00000095261.14 | *PSMD5* | 1.7 | 1.31E-02 | 743.3 | 1295.6 |
| ENSG00000168994.13 | *PXDC1* | -2.2 | 5.37E-05 | 470.4 | 209.9 |
| ENSG00000188060.7 | *RAB42* | -78.5 | 4.91E-02 | 20.6 | 0.3 |
| ENSG00000204764.14 | *RANBP17* | 3.3 | 2.85E-02 | 21.0 | 69.3 |
| ENSG00000244395.6 | *RBMY1D* | 2.0 | 1.80E-02 | 571.6 | 1116.3 |
| ENSG00000015568.13 | *RGPD5* | 2.0 | 4.80E-02 | 145.1 | 290.6 |
| ENSG00000265681.7 | *RPL17* | 2.8 | 4.26E-02 | 954.0 | 2697.5 |
| ENSG00000241343.9 | *RPL36A* | 1.7 | 7.91E-03 | 1028.5 | 1700.2 |
| ENSG00000117616.18 | *RSRP1* | 1.7 | 7.59E-03 | 386.3 | 655.4 |
| ENSG00000213694.5 | *S1PR3* | -2.6 | 4.26E-02 | 108.9 | 41.4 |
| ENSG00000177409.12 | *SAMD9L* | -123.6 | 5.37E-03 | 32.6 | 0.3 |
| ENSG00000164023.14 | *SGMS2* | -3.5 | 1.03E-02 | 136.9 | 39.3 |
| ENSG00000163597.15 | *SNHG16* | 5.7 | 6.21E-03 | 40.0 | 229.0 |
| ENSG00000159140.20 | *SON* | -1.9 | 9.74E-05 | 4681.4 | 2496.5 |
| ENSG00000115306.16 | *SPTBN1* | -1.6 | 6.21E-03 | 5422.3 | 3404.8 |
| ENSG00000124356.16 | *STAMBP* | -1758666.2 | 1.02E-03 | 39.8 | 0.0 |
| ENSG00000168610.14 | *STAT3* | -10.4 | 2.81E-02 | 63.2 | 6.1 |
| ENSG00000168439.16 | *STIP1* | 6.4 | 5.71E-03 | 88.3 | 566.0 |
| ENSG00000197321.14 | *SVIL* | -1.9 | 3.90E-02 | 261.6 | 137.8 |
| ENSG00000147041.12 | *SYTL5* | 2.1 | 5.71E-03 | 139.0 | 290.7 |
| ENSG00000168394.11 | *TAP1* | -25.4 | 5.89E-06 | 113.6 | 4.5 |
| ENSG00000113407.13 | *TARS* | 4.0 | 2.95E-02 | 171.2 | 678.6 |
| ENSG00000168016.14 | *TRANK1* | -7.1 | 4.16E-05 | 72.6 | 10.2 |
| ENSG00000132274.16 | *TRIM22* | -94.9 | 2.00E-02 | 25.1 | 0.3 |
| ENSG00000109103.11 | *UNC119* | 4.6 | 7.91E-03 | 12.7 | 58.3 |
| ENSG00000174282.12 | *ZBTB4* | -1.6 | 2.18E-02 | 490.4 | 300.5 |
| ENSG00000105939.13 | *ZC3HAV1* | -1.5 | 9.47E-03 | 1328.3 | 867.3 |
| ENSG00000152518.8 | *ZFP36L2* | -1.5 | 7.91E-03 | 1920.1 | 1243.2 |
| ENSG00000088876.12 | *ZNF343* | 2.0 | 1.31E-02 | 102.6 | 207.9 |
| ENSG00000185869.14 | *ZNF829* | 161.0 | 2.15E-03 | 0.1 | 20.8 |
| ENSG00000124201.15 | *ZNFX1* | -1.6 | 2.51E-02 | 1244.4 | 786.4 |
