# Supplementary material for: FRA1 (FOSL1) suppresses neoplastic transformation and modulates radiation responses via transcriptional control of mitogenic and stress-responsive networks
Source: Front Cell Dev Biol. 2025 Sep 22;13:1659986. doi: 10.3389/fcell.2025.1659986 (PMC12497744; doi:10.3389/fcell.2025.1659986)
Supplement: Supplementary file 2 [file DataSheet1.pdf]

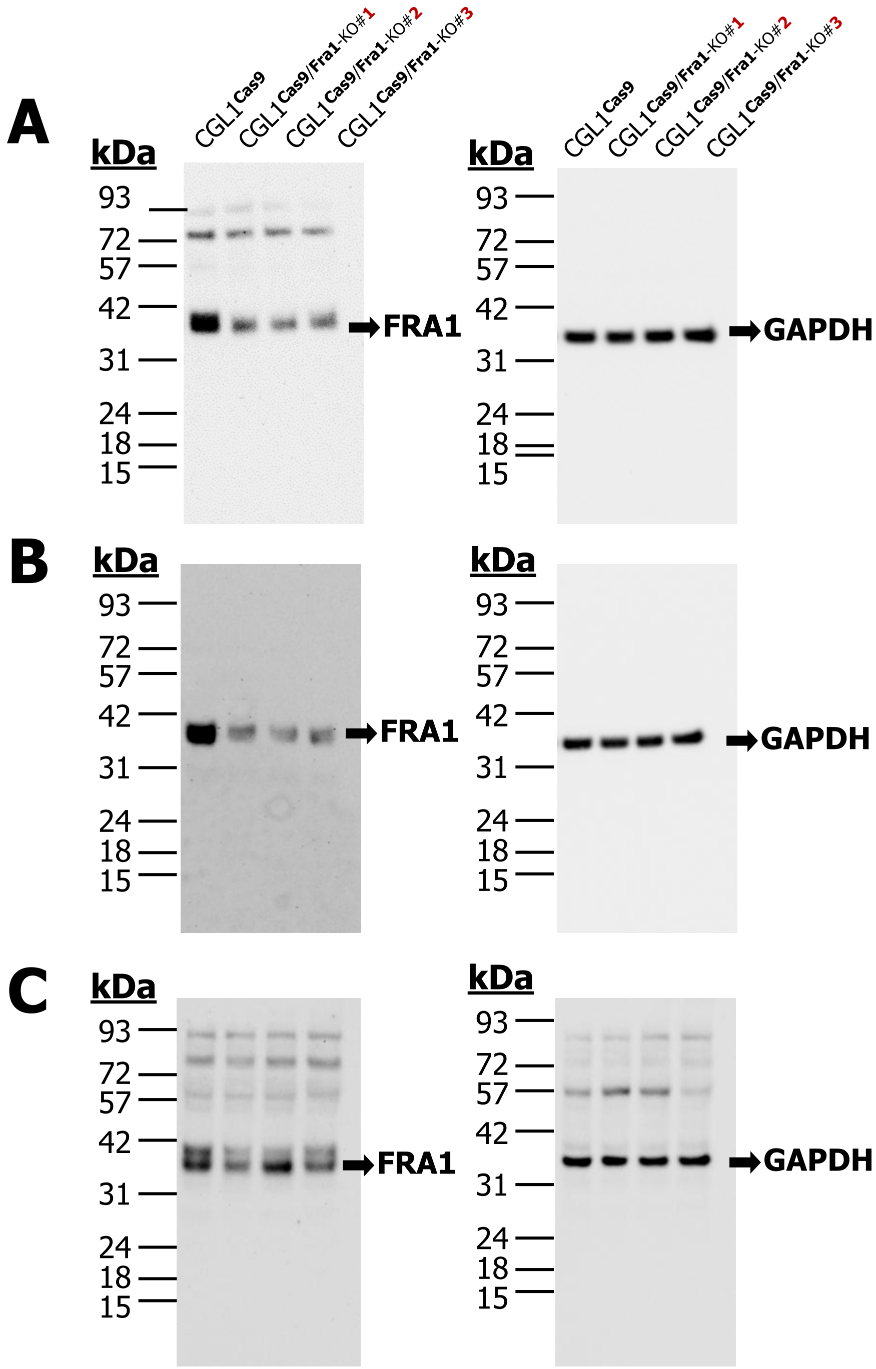

Supplementary Figure 1. Raw immunoblot images for Figure 1A. Unprocessed Western blot images showing FRA1 protein expression in $CGL1^{Cas9}$ control cells and three independently generated FRA1 knockout clones ($CGL1^{FRA1KO\#1–3}$). (A–C) Three independent biological replicates of FRA1 immunoblots are shown. Corresponding GAPDH blots are provided as loading controls. Molecular weight markers (kDa) are indicated on the left side of each blot; FRA1 protein is detected at ~40 kDa and GAPDH at ~37 kDa. These replicate blots were used for quantification presented in Figure 1A of the main manuscript.

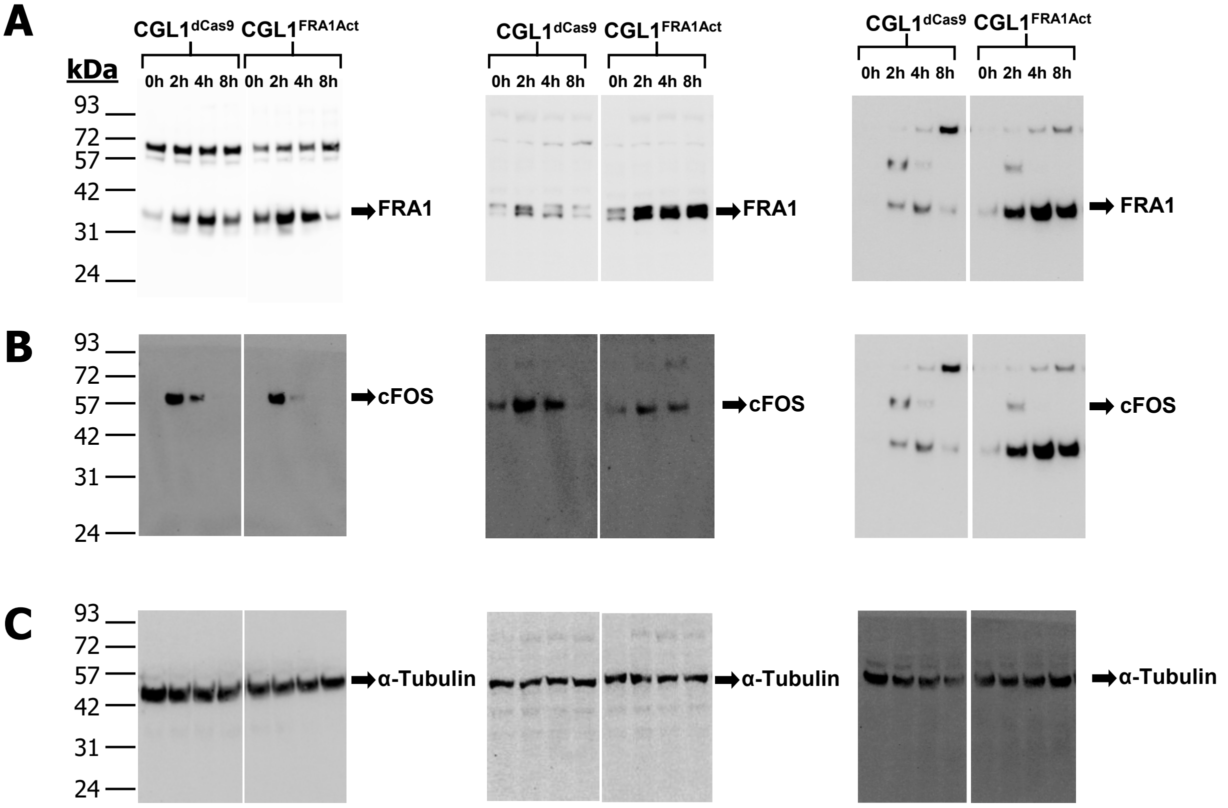

Supplementary Figure 2. Immunoblot images for Figure 8A. (A–C) Blot images for FRA1 and cFOS protein expression in CGL1^{FRA1Act} cells and their matched control (CGL1^{dCas9}) at 0, 2, 4, and 8 hours following serum stimulation. Each panel represents one of three independent biological replicates used for quantification in Figure 8C and 8E of the main manuscript. α-Tubulin was used as the loading control. Molecular weight markers (kDa) are indicated; FRA1 protein is detected at ~40 kDa, cFOS protein is detected at ~62 kDa, and α-Tubulin at ~52 kDa.

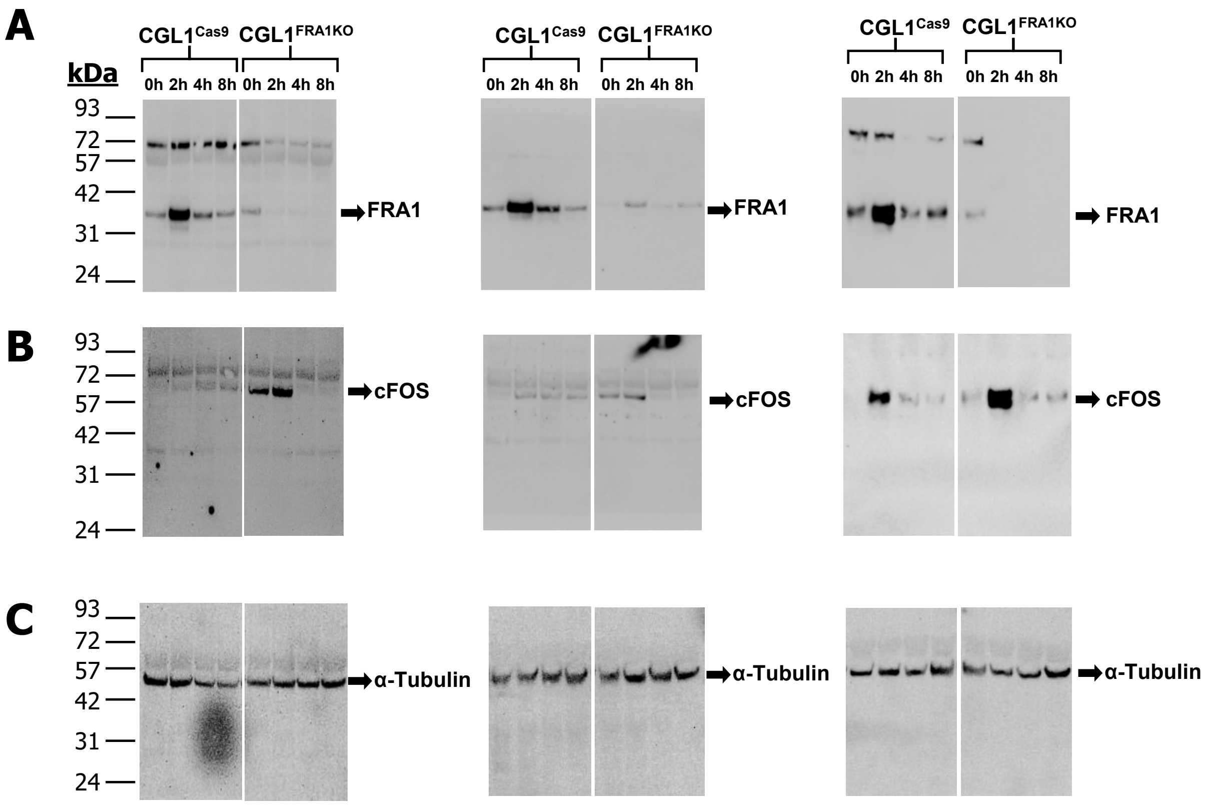

Supplementary Figure 3. Immunoblot images for Figure 8B. (A–C) Blot images for FRA1 and cFOS protein expression in $CGL1^{FRA1KO}$ cells and their matched control ($CGL1^{Cas9}$) at 0, 2, 4, and 8 hours following serum stimulation. Each panel represents one of three independent biological replicates used for quantification in Figure 8D and 8F of the main manuscript. α-Tubulin was used as the loading control. Molecular weight markers (kDa) are indicated; FRA1 protein is detected at ~40 kDa, cFOS protein is detected at ~62 kDa, and α-Tubulin at ~52 kDa. These blots confirm FRA1 and cFOS protein expression patterns observed in Figure 8.
